# Whole Transcriptome Analysis of Pre-invasive and Invasive Early Squamous Lung Carcinoma in Archival Laser Microdissected Samples

**DOI:** 10.1186/s12931-016-0496-3

**Published:** 2017-01-10

**Authors:** Andre Koper, Leo A. H. Zeef, Leena Joseph, Keith Kerr, John Gosney, Mark A. Lindsay, Richard Booton

**Affiliations:** 1 Qiagen GmbH, Hilden, 40724 Germany; 2 Faculty of Life Science, University of Manchester, Manchester, England M13 9PT UK; 3 Department of Pathology, University Hospital of South Manchester, Manchester, England M23 9LT UK; 4 Department of Pathology, University of Aberdeen, Aberdeen, Scotland AB25 2ZD UK; 5 Department of Pathology, Royal Liverpool University Hospital, Liverpool, England L7 8XP UK; 6 Department of Pharmacy and Pharmacology, University of Bath, Bath, England BA 7AY UK; 7 Manchester Thoracic Oncology Centre, University Hospital of South Manchester, Manchester, England M23 9LT UK

**Keywords:** Preinvasive squamous cell cancer, Invasive squamous cell cancer, Exon arrays, Gene expression profiling, Microdissection

## Abstract

**Background:**

Preinvasive squamous cell cancer (PSCC) are local transformations of bronchial epithelia that are frequently observed in current or former smokers. Their different grades and sizes suggest a continuum of dysplastic change with increasing severity, which may culminate in invasive squamous cell carcinoma (ISCC). As a consequence of the difficulty in isolating cancerous cells from biopsies, the molecular pathology that underlies their histological variability remains largely unknown.

**Method:**

To address this issue, we have employed microdissection to isolate normal bronchial epithelia and cancerous cells from low- and high-grade PSCC and ISCC, from paraffin embedded (FFPE) biopsies and determined gene expression using Affymetric Human Exon 1.0 ST arrays. Tests for differential gene expression were performed using the Bioconductor package limma followed by functional analyses of differentially expressed genes in IPA.

**Results:**

Examination of differential gene expression showed small differences between low- and high-grade PSCC but substantial changes between PSCC and ISCC samples (184 vs 1200 p-value <0.05, fc ±1.75). However, the majority of the differentially expressed PSCC genes (142 genes: 77%) were shared with those in ISCC samples. Pathway analysis showed that these shared genes are associated with DNA damage response, DNA/RNA metabolism and inflammation as major biological themes. Cluster analysis identified 12 distinct patterns of gene expression including progressive up or down-regulation across PSCC and ISCC. Pathway analysis of incrementally up-regulated genes revealed again significant enrichment of terms related to DNA damage response, DNA/RNA metabolism, inflammation, survival and proliferation. Altered expression of selected genes was confirmed using RT-PCR, as well as immunohistochemistry in an independent set of 45 ISCCs.

**Conclusions:**

Gene expression profiles in PSCC and ISCC differ greatly in terms of numbers of genes with altered transcriptional activity. However, altered gene expression in PSCC affects canonical pathways and cellular and biological processes, such as inflammation and DNA damage response, which are highly consistent with hallmarks of cancer.

**Electronic supplementary material:**

The online version of this article (doi:10.1186/s12931-016-0496-3) contains supplementary material, which is available to authorized users.

## Background

Lung cancer has been the most common fatal cancer worldwide in the last 30 years. Its 5-year survival rate is poor at less than 15% and has not improved significantly since the 1970’s. However, the prospect of survival in treated stage I lung cancer are greater than from stage II lung cancer or worse. Hence, it is suggested that an early diagnosis is pivotal for successful treatment, and advancing the means for early diagnosis will reduce mortality of this disease [1, 2].

About 20% of all lung cancers are ISCC that arise from epithelia that line the upper airways [3]. Patients at high risk for ISCC, such as smokers, are susceptible to focal squamous changes within the upper airway epithelia that are detectable by sensitive autofluorescence bronchoscopy techniques [4, 5]. The lesions of PSCC are usually small, do not disrupt the basement membrane and show a diverse histological spectrum that suggests a gradual morphological transformation of bronchial epithelia into low- and high-grade PSCC that eventually may progress into ISCC [6]. High-grade lesions in sputum or bronchial biopsies indicate a higher risk for lung cancer within the airway and at remote parenchymal sites and are therefore regarded as important clinical indicators [6, 7]. However, the prognostic benefit of PSCC is impaired by a challenging histopathological classification and uncertainty about their individual malignant potential, which may impair their clinical relevance [8, 9].

Squamous cancers accumulate structural chromosomal damage that increase in number and size at well-recognised genomic positions in high-grade PSCC and ISCC [10–12]. Moreover, amplifications of the distal part of chromosome 3q may correlate with progression of high-grade PSCC to ISCC, and amplifications of this particular chromosomal area are a significant feature of ISCC in the lung and the esophagus, which is postulated to lead to an up-regulation of gene expression of known oncogenic potential, such as *SOX2* or *PIK3CA* [13, 14]. These findings strongly suggest distinct gene expression changes that underpin both PSCC and ISCC and which may offer insights into the mechanism of progression from a pre-invasive to an invasive tumour and potentially aid the phenotypic classification of PSCC according to their malignant potential. However, this has been hindered by the scarcity of fresh and longitudinally harvested material and the experimental challenges associated with analysis of the widely available FFPE biopsies.

In this report, we have examined the changes in gene expression in PSCC and ISCC using RNA from microdissected archival biopsies obtained from the University Hospital of South Manchester. To generate genome-wide gene expression profiles across a reliable histological classification of these samples, expert pathological review of lesions was agreed prior to analysis with Human Exon 1.0 ST arrays, which demonstrate greater accuracy of gene expression estimation using genomic material obtained from FFPE biopsies [15, 16].

## Methods

### Laser capture microscopy (LCM) and RNA extractions

All FFPE biopsies were selected by a specialist thoracic pathologist, sectioned using a Leica RM2125 microtome and stained with Hematoxylin and Eosin. PSCC, ISCC cell clusters and normal epithelia were microdissected as follows: each FFPE biopsy was used to produce a series of a single 5μm section, transferred to a standard glass slide for diagnostic evaluation by specialist thoracic pathologists, and on average ten 10μm sections were each placed on 1mm PEN membrane slides (Carl Zeiss, Germany) for LCM using a Leica LMD6000 (Additional file 1: Figure S1). RNA was isolated using the Ambion Recover All Kit. Isolated RNA was quantified using a Qubit fluorometer (Qubit RNA assay kit) and RNA integrity was examined using an Agilent Bioanalyser. Samples with RIN values in the range 2–3 were employed for microarray analysis. All donors gave written informed consent and the conducted research was approved by the South Manchester Ethics Committee

### Library preparations and array hybridization

Gene expression was analysed as previously described [16]. 50ng of total RNA was used for amplification and reverse transcription of individual samples using the Nugen Ovation FFPE WTA Kit, followed by biotin labelling and library fragmentation via the Nugen Encore Biotin Kit. Affymetrix Human Exon 1.0 ST array hybridisation, washing, staining and scanning was performed at the Molecular Biology Core Facility of the CRUK Manchester Institute.

### Real-time PCR (RT-PCR)

Comparative RT-PCR was performed to validate expression changes of candidate genes between ISCC and paired normal biopsies by using the delta delta Ct method [17]. All primers were designed using Primer-BLAST (see Additional file 2: Table S1). PCR reactions were setup using Applied Biosystem’s Fast SYBR green master mix and were run in triplicates on an Applied Biosystems 7900HT Real-Time PCR system. Primers targeting 28S were used for the endogenous control in all assays [18].

### Bioinformatics

Exon array quality control and outlier detection was performed using dChip (www.dchip.org, [19]). For normalization and expression analysis of the array data, the implementation of the RMA algorithm in Partek GS 6.6 (Copyright 2010, Partek Inc., St. Charles, MO, USA) using core probesets was used. Differential expression tests were performed with the Bioconductor package limma using paired designs [20]. The Bioconductor package QVALUE was used to calculate the corresponding q-values [21]. Principal component analysis (PCA) of normalized expression data from the included exon arrays was performed using the R package FactoMineR (https://cran.r-project.org/web/packages/FactoMineR/). Gene Set Enrichment Analysis was used to calculate positional enrichment of abnormal transcription using pre-ranked list of aberrantly expressed genes in all ISCC or all PSCC and positional gene sets available from MSigDB (http://www.broadinstitute.org/gsea/msigdb) according to previously published procedures [22]. RCircos was used to create the circus plot [23]. For clustering analysis, 1914 differentially expressed genes from the three contrasts, i.e., all ISCC versus control samples, all PSCC samples versus control samples and PSCC high-grade versus PSCC low-grade, were selected and filtered for *p*-value <0.05 and fold change greater than +/−1.75. For the clustering analysis, 4 data points were used; average controls, average PSCC low-grade, average PSCC high-grade and average ISCC. Averages are calculated in log base 2 and were standardised (standard deviation normalised to 1 and mean to 0). Genes were clustered according to these standardised expression levels by k-means into 12 clusters followed by ranking by hierarchical clustering using maxdView software "Super Grouper" plugin (available from http://bioinf.man.ac.uk/microarray/maxd/). The functional analyses of differentially expressed genes were generated through the use of QIAGEN’s Ingenuity Pathway Analysis (IPA®, QIAGEN Redwood City, https://www.qiagenbioinformatics.com/products/ingenuity-pathway-analysis/). Microarray data has been uploaded to the ArrayExpress database (www.ebi.ac.uk/arrayexpress) under accession number E-MTAB-3950.

### Immunohistochemistry

Immunohistochemistry on FFPE lung biopsies was performed on the Leica Bond platform, which included standard procedures for the removal of paraffin wax, section rehydration, epitope retrieval (Leica Bond Epitope Retrieval Solution 2, 20 min, Leica Biosystems, Germany), blocking of endogenous peroxidases for 5 min and blocking with 10% casein for 10 min. The primary antibody (Anti-PTTG1, Sigma-Aldrich HPA008890, produced in rabbit) was used at 5 μg/ml for 15 min followed by treatment with the Leica Bond Refine Kit (8 min, Leica Biosystems, Germany), Leica Bond DAB (10 mins, Leica Biosystems, Germany) and a counterstaining with hematoxylin prior to rehydration, clearing and coverslipping. Images were taken on a Leica SCN 400.

## Results

### Classification of the biopsy samples

**Fig. 1:**
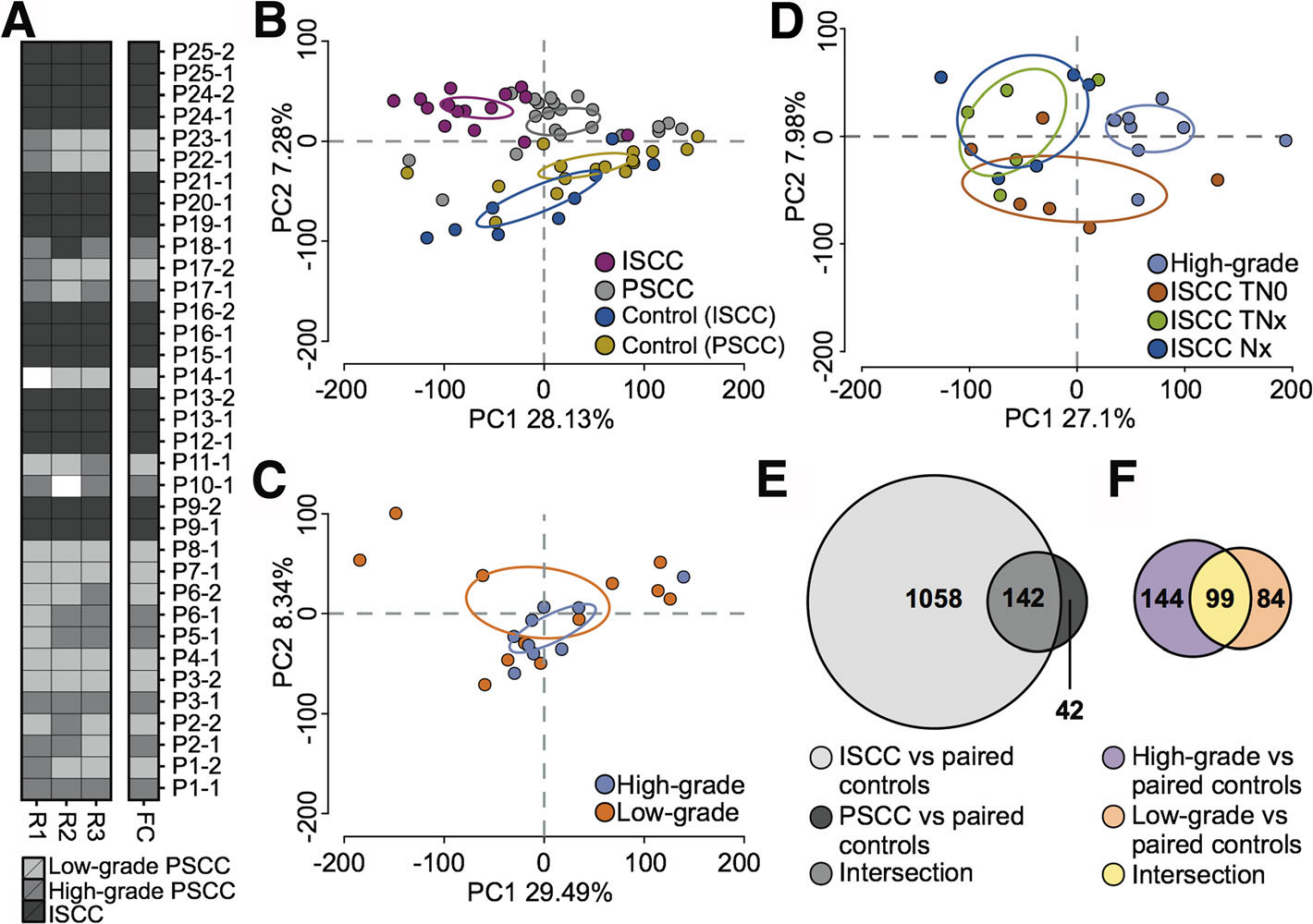
Sample classification, general variability of normalized gene expression and differential gene expression in the selected FFPE PSCC and ISCC samples. **a** Sample classification according to independent reviews by three expert histopathologists. Each of the first three columns in the heatmap corresponds to one of the three involved pathologists (R1, R2 and R3) and rows to the selected samples. The color of each heatmap cell indicates the corresponding sample classification (see legend, white = no review for technical reasons). The FC column represents the final classification based on two concordant reviews. **b** Plot shows the result of a Principal component analysis (PCA) of normalized gene expression data obtained from all the included samples. ISCC and PSCC samples are clearly separated. Ellipses delineate 95% confidence level for each sample class respectively. **c** PCA plot of normalized gene expression data from low- and high-grade PSCC. Samples are widespread and show no clear separation. **d** PCA plot of normalized gene expression data from ISCC and high-grade PSCC. TN0, TNx and Nx ISCC samples are widespread and do not show recognizable differences. High-grade PSCC are clearly separated from ISCC samples. **e** Venn diagram illustrate the numbers of differentially expressed genes in ISCC and PSCC, including the intersection between both. **f** The Venn diagram represents the numbers of differentially expressed genes in high-grade and low-grade PSCC, including the intersection between both

FFPE biopsies used in this study originate from the Manchester Cancer Research Centre Biobank and sample interpretation was undertaken by three specialist thoracic pathologists who classified areas of normal bronchial epithelium, PSCC and ISCC. Pathologists also subdivided PSCC into low- or high-grade lesions, according to mild and moderate squamous dysplasia (low grade dysplasia) and severe squamous dysplasia and carcinoma in-situ (CIS) (high grade dysplasia). Final sample classifications were based on two concordant reviews; those without a majority agreement or technically uninterpretable were excluded. Using this approach, we obtained 15 ISCC regions (from surgical specimens) and 20 PSCC regions (from bronchial biopsies) from 25 patients (Fig. 1a, Additional file 2: Table S2). ISCC samples include 10 primary tumours (5 lymph node-negative (TN0), 5 lymph node-positive (TNx)) and 5 paired lymph node metastases (Nx) to the primary TNx samples. PSCC samples were composed of 8 high-grade and 12 low-grade lesions. The median age of the 17 male (68%) and 8 female (32%) donors at the time of sampling was 65years (range 45-79years). 72% (18/25) where treated for ISCC, 8% (2/25) for head and neck squamous cell carcinoma and another 8% (2/25) for CIS. Three patients (12%) had no treatment. A history of smoking was recorded in 52% (13/25) of the donors.

### PCA of expression data and assessment of differential RNA expression in pre-invasive and invasive squamous carcinomas

RNA libraries isolated from 15 ISCC (and 10 paired samples of control normal bronchial epithelia) and 20 PSCC (and 15 paired samples of control normal bronchial epithelia) using micro-dissection were amplified and converted into cDNA for hybridisation using Exon 1.0 ST arrays. Principal Component Analysis (PCA) was performed in order to visualize variability of the entire normalized expression data from all arrays included in this study (Fig. 1b). In this PCA, ISCC and PSCC data points show a clear tendency of being separated from each other by mainly the first principal component (explains 28.13% of the observed variability in gene expression), which suggests that the general gene expression in ISCC and PSCC is strikingly different (Fig. 1b and 95% confidence intervals therein). Controls paired to ISCC or PSCC were widely distributed across the PCA plot but did not show any significant clustering according to sample origin (surgical or bronchial biopsies) or their various locations (see Additional file 2: Table S2) (Fig. 1b). However, a PCA of low-grade and high-grade PSCC showed widely spread low-grade PSCC data points that overlap with high-grade PSCC data points (Fig. 1c), and similarly, no clear clusters of TN0, TNx or Nx ISCC data points were observed in a PCA (Fig. 1d), which suggests that the observed variability of gene expression changes in these samples do not differentiate these distinct histological categories. Furthermore, the observed gene expression was clearly different between ISCC and high-grade PSCC, which were previously reported with similar changes to chromosomal structures and which were suggested to be more prone to malignant transformations (Fig. 1d) [13, 24, 25].

Following comparison with matched normal epithelium, 1200 RNAs were differentially expressed in ISCC samples (*p* < 0.05 and fold change ±1.75, Additional file 2: Table S3). As might be expected, a considerably smaller number of 184 RNAs were differentially expressed in PSCC samples (Additional file 2: Table S4). However, 142 (77%) of these RNAs were abnormally expressed in both PSCC and ISCC (Fig. 1e, Additional file 2: Table S5). Further comparative analysis of the high-grade and low-grade preinvasive samples and their controls confirmed differential expression of 243 (Additional file 2: Table S6) and 183 RNAs (Additional file 2: Table S7) respectively, of which 99 RNAs (40% and 54% respectively) were differentially expressed in both low-grade and high-grade groups (Fig. 1e, Additional file 2: Table S8). Hence, the obvious difference in the amount of abnormal gene expression between ISCC and PSCC suggests a substantial molecular difference between these two conditions, but no such dramatic changes were found between low-grade and high-grade PSCC, which in the case of PSCC reasons against a process of gradually added modifications to gene transcription. Nevertheless our analysis identified abnormal gene expression shared between ISCC and PSCC that supports the assumption of a common ancestry and the possibility of molecular events that could drive the progression of squamous lesions.

### Genome-wide assessment of coordinately regulated chromosomal regions in ISCC and PSCC

**Fig. 2:**
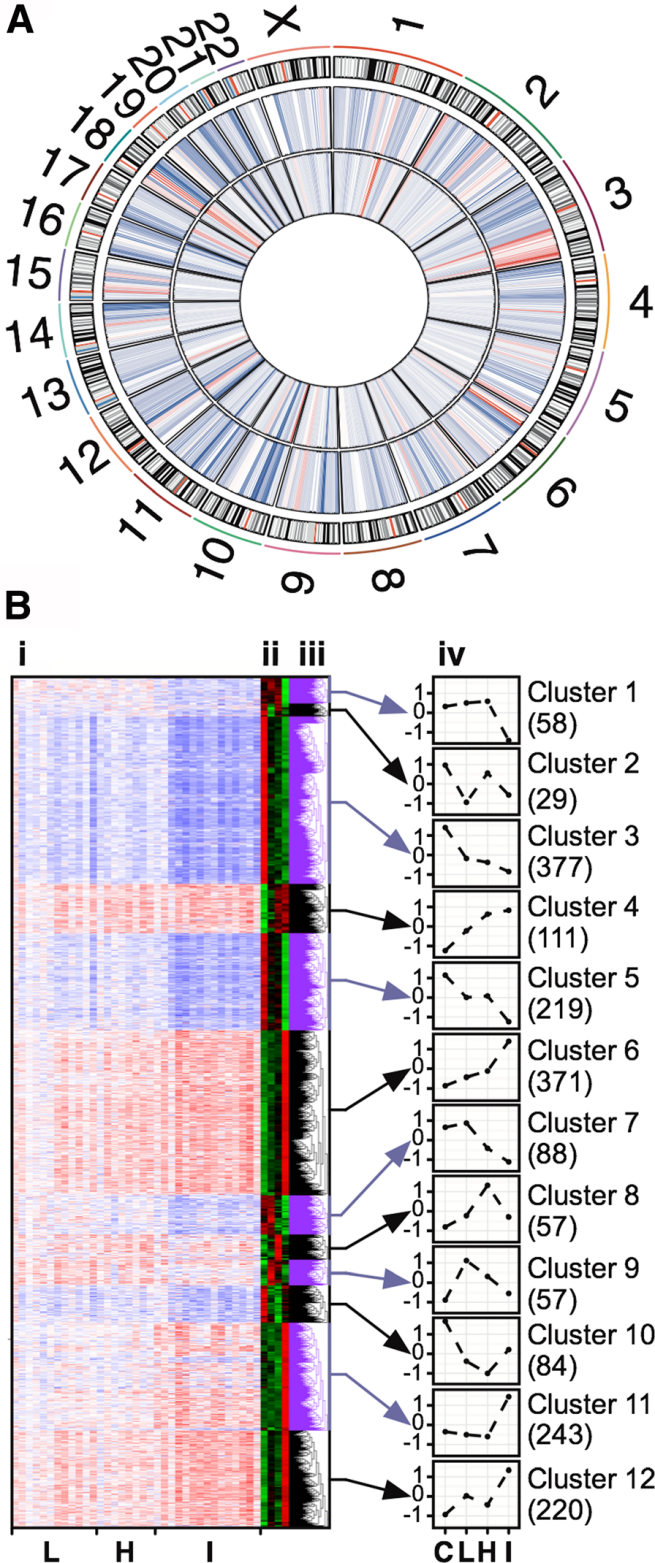
Positional enrichment and cluster analysis differentially expressed genes in PSCC and ISCC. **a** Genome wide positional enrichment of aberrant transcription in PSCC and ISCC. Tracks of the circos plot from outside to inside: chromosome ideogram (except Y chromosome), in which red lines demarcates the centromere; heatmap of enriched aberrant transcription in ISCC; heatmap of enriched aberrant transcription in ISCC. In both heatmaps gene up-regulation is coded in red and gene down-regulation in blue. **b** Cluster analysis of genes differentially expressed in low-grade PSCC, high-grade PSCC or ISCC; (i) heatmap of standardized gene expression; (ii) z-scores and (iii) histograms of standardized expression of the selected genes across normal bronchial epithelia (C), low-grade PSCC (L), high-grade PSCC (H) and ISCC (I); (iv) profiles of aberrant gene expression for the 12 identified clusters. The value in brackets represents the number of genes assigned to each of the 12 clusters

We used Gene Set Enrichment Analyses (GSEA) with pre-ranked lists of differently expressed genes derived from all ISCC or PSCC samples versus paired controls using the positional gene sets provided by MSigDB in order to correlate aberrant transcription to chromosomal positions as previously reported for PSCC and ISCC [22, 24]. Significantly enriched gene up-regulation in ISCC was found for chromosomes 2p25, 3q21, 3q24, 3q26-28, 6p22, 15q11 and 18q12 (Fig. 2a). Similarly, gene down-regulation in ISCC was significantly enriched at 2q35, 9p13, 10q11, 11p14, 11q12, 12q14 and 18q21 (Fig. 2a). Interestingly, significant positional gene expression changes at the positions 2q35, 10q11, 11q12, 12q14 (down-regulation) as well as 3q21 and 18q12 (up-regulation) were detected in both ISCC and PSCC (Fig. 2a). As reported previously, this analysis confirms elevated levels of gene expression in ISCC and, to a lesser extent, PSCC at the distal part of chromosome 3q (Fig. 2a). Conversely, chromosome 3p confirmed a moderate degree of down-regulation, most evident in ISCC (Fig. 2a). This data supports and extends previous observations of structural changes to chromosomes in PSCC that are thought be important in the phenotypic transformation to invasive malignancies and further suggests the propagation of aberrant transcriptional activity during squamous carcinogenesis [11, 13, 22].

### Pathway analysis of differentially expressed mRNA in pre-invasive and invasive squamous carcinomas confirms a malignant phenotype

**Fig. 3:**
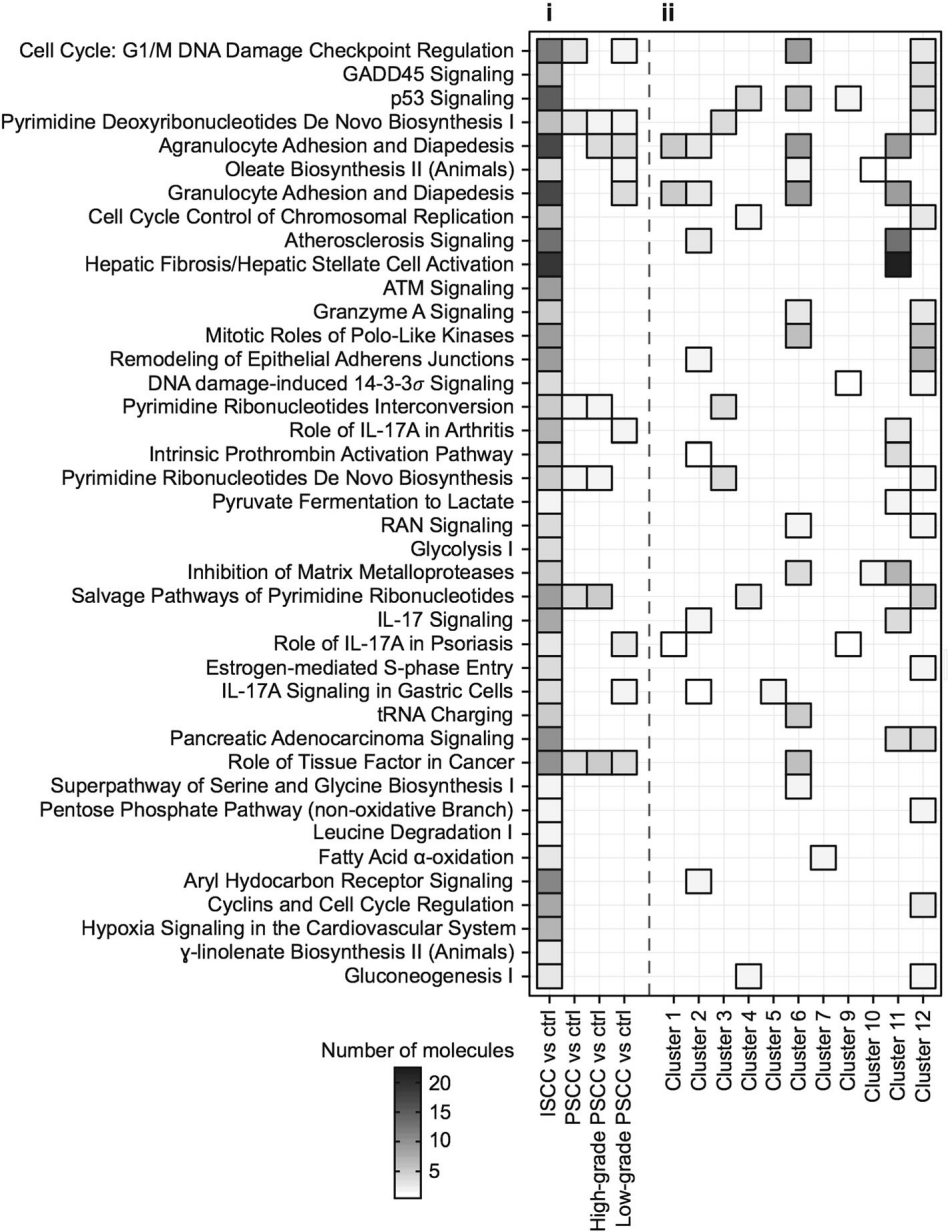
Ingenuity pathway analysis (IPA) of aberrant transcription in PSCC and ISCC. (**i**) The first heatmap column shows all IPA pathways that are significantly affected in ISCC (*p*-value < =0.05). The remaining 3 columns indicate the association of these IPA pathways with differentially expressed genes in all PSCC, high-grade PSCC or low-grade PSCC. (**ii**) This heatmap summarise the connection between IPA pathways significantly related to gene expression changes in ISCC (*p*-value < =0.05) and genes sorted by cluster analysis. Note that genes from cluster 8 were not linked to any IPA pathways associated with ISCC

Ingenuity pathway analysis (IPA) was employed to identify canonical pathways linked to genes that were differentially expressed in ISCC, total PSCC, high-grade PSCC and low grade PSCC (Fig. 3 and Additional file 2: Table S9). The widespread changes in genes expression in ISCC samples were associated with 40 significantly affected pathways (*P*: <=0.05) including those characteristic for invasive malignancies such as ‘Cell Cycle: G2/M DNA Checkpoint Regulation’ (*p*-value: 2.18e-6, z-score: −1.414), ‘ATM signaling’ (*p*-value: 0.00173, z-score: −1.134), ‘p53 Signaling’ (*p*-value: 5.75e-5, z-score: −0.905) and ‘GADD45 Signaling’ (*p*-value: 9.12e-6) [25–27]. IPA further suggested ‘Cell Cycle: G2/M DNA Checkpoint Regulation’, pathways concerned with DNA and RNA metabolism and inflammation-related processes to be affected in all contrasts, which is line with the finding of differential gene expression shared between ISCC and PSCC. Overall, this pathway analysis further supports the existence of shared aberrant gene expression in the analysed samples with potential to affect cellular functions that may drive squamous carcinogenesis across pre-invasive and invasive stages.

### Cluster analysis refines gene expression profiles across PSCC and ISCC

A cluster analysis was performed using 1914 genes that are differentially expressed in either of the low-grade PSCC, high-grade PSCC or ISCC samples in order to identify genes that share a particular expression pattern across these samples. Among the 12 identified clusters, three (clusters 4, 6 and 12) showed a gradual increase of expression of 702 genes (36.7% of 1914 genes) across controls, PSCC and ISCC (Fig. 2b). Signalling pathways associated with these genes by IPA cover functional themes of DNA damage response and cell cycle regulation, including ‘cell cycle: G2/M DNA checkpoint regulation’ (z-score: −0.447), ‘p53 signaling’, ‘GADD45 signaling’ and ‘mitotic roles of Polo-like kinases’, and other pathways associated with tumor development, such as ‘Rac signaling’ (z-score: 2.449), ‘RhoA signaling’, ‘ERK/MAPK signaling’ (z-score: 2.236) and ‘PI3K/AKT signaling’ (z-score: 2.449) (Fig. 2b and Fig. 3, see Additional file 2: Table S10 for full details) [28]. Two clusters (3 and 5) of 596 genes (31.1% of 1914 genes) were found gradually down regulated from control to PSCC and then ISCC. These included several cytochrome P450 genes (*CYP4B1*, *CYP2B6*, *CYP4Z1*) that indicates a reduced metabolism of potential carcinogens (see Additional file 2: Table S10). In addition, we identified 243 RNAs in cluster 11 (12.7% of 1914 genes) that demonstrated an increase in expression levels only in ISCC that were linked to changes in the extracellular matrix and an increase in inflammation-related processes (‘IL-17 signaling’, ‘IL-8 signaling’, ‘agranulocyte/granulocyte adhesion and diapedesis’, see Additional file 2: Table S10). Hence, using cluster analysis and IPA we were able to sort genes with similar expression profiles across PSCC and ISCC (see Additional file 2: Table S11) and to identify biological themes associated with these profiles, which refines our understanding of the molecular pathology that underlies squamous carcinogenesis. As summarised in Fig. 3, IPA pathways associated with ISCC are mainly, but not exclusively, linked to a gene up-regulation.

### Validation of gene expression data in ISCC samples

**Fig. 4:**
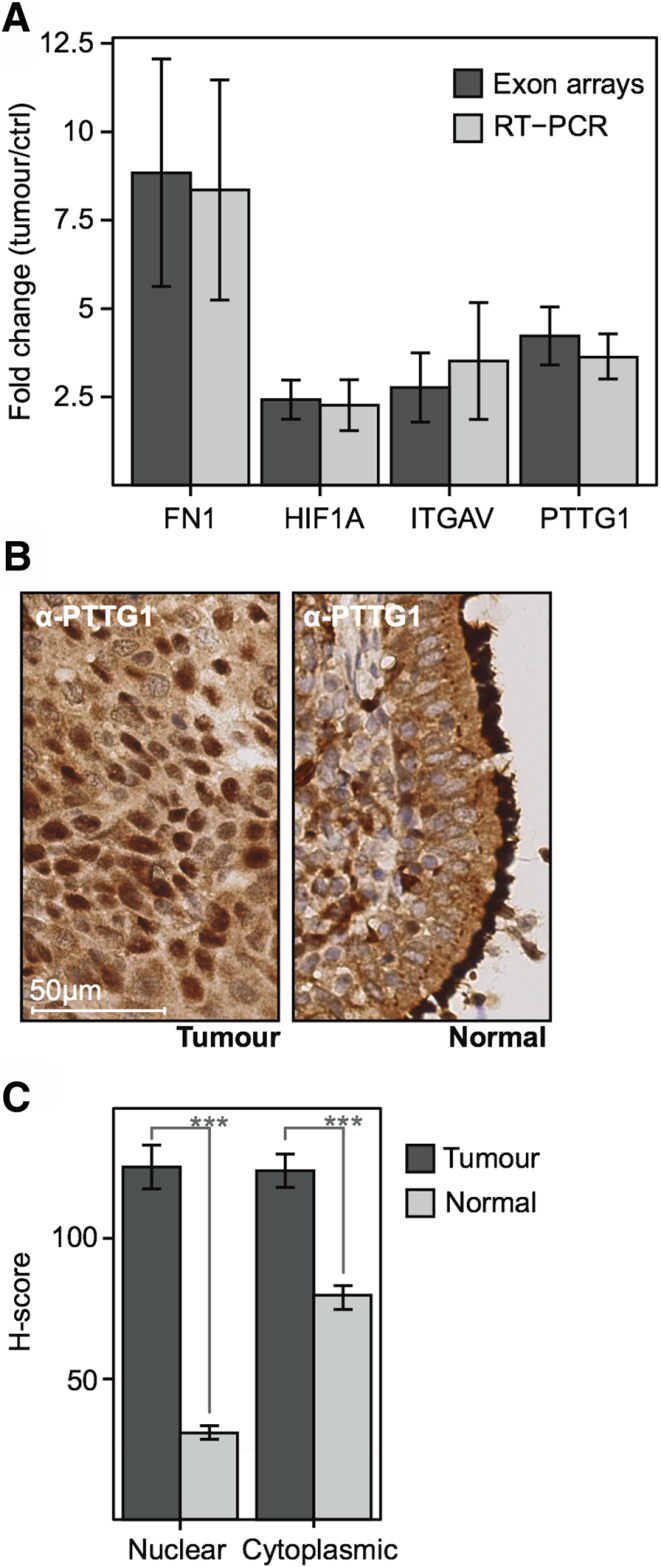
Validation of the array data obtained from FFPE PSCC and ISCC samples. **a** The bar plot compares the fold changes of mRNA levels for four individual genes in ISCC tumours and paired controls obtained either by Human Exon arrays or RT-PCR. **b** Anti-PTTG1 staining in FFPE ISCC (Tumour) and paired bronchial mucosa (Normal). In ISCC anti-PTTG1 appears moderately in the cytoplasm and strongly in about ~70% of nuclei. The used ISCC is unrelated to the samples for the gene expression study. **c** H-scores of anti-PTTG1 immunostaining in a set of 45 independent ISCC (Tumour) and paired bronchial epithelia (Normal). Cytoplasmic and nuclear PTTG1 levels are significantly raised in ISCC and therefore cross-validate the gene expression data. Nuclear staining is increased 4x in ISCC whereas the cytoplasmic was raised 1.5x

To validate the microarray data, we undertook RT-PCR to confirm changes in gene expression of four candidate genes, PTTG1, FN1, HIF1alpha and ITGAV that have been reported to drive tumorigenesis. As a result of the availability of independent samples, this could only be performed in ISCC samples. As with the microarray data, RNA expression of these four genes was found to gradually increase across the tested histology and to peak in ISCC samples. Thus, the observed upregulation of these four genes was confirmed by RT-PCR: PTTG1 (P: 0.0015), FN1 (P: 0.0011), HIF1α (P: 0.0017) and ITGAV (P: 0.0082) in ISCC samples with fold changes comparable to the results obtained from the Human Exon arrays (Fig. 4a). In addition, we performed cross-validation of PTTG1 up-regulation using FFPE tissue microarrays of 45 unrelated ISCC and paired normal bronchial epithelia by immunohistochemistry. The obtained antibody stainings were quantified by an experienced pathologist using the H-score system, which revealed a significant up-regulation of PTTG1 in the cytoplasmic (mean in tumor: 124.1, mean in control: 79.78, P: 3.97e-08) and nuclear (mean in tumor: 125.4, mean in control: 30.66, P: 4.35e-16) compartments of tumor cells in comparison to cells in paired pseudo stratified epithelia that were used as controls. As suggested by the H-scores, a prevalent nuclear localization of PTTG1 protein was noted in ISCC (Fig. 4b and c). Hence, we detected a significant up-regulation of PTTG1 in malignant squamous cells from an independent set of ISCC that confirms the obtained array data and is in line with previously published PTTG1 expression in lung and other cancers [29].

## Discussion

Understanding the molecular alteration that underpins malignant squamous transformations within the airway epithelia is pivotal for the development of novel means of early detection or treatments of squamous cell carcinomas at a non-lethal stage [30, 31]. Recently published studies utilized different grades of FFPE PSCC samples that revealed incremental increase of chromosomal rearrangements to occur during the proposed model of squamous carcinogenesis, and although these findings imply changing transcriptional profiles, the available knowledge of genes or pathways with altered transcriptional profiles in these conditions remains sparse [6, 32]. Only a few studies so far have successfully addressed this shortcoming and uncovered a number of genes, e.g., *SOX2*, *CEACAM5* or *SLC2A1*, with the potential of inducing malignant traits in PSCC by amplification or up-regulation in fresh samples of PSCC and ISCC [13, 22, 33].

Surprisingly, the results from the array data presented here suggest a massive surge of significantly altered transcription in ISCC in comparison to PSCC, while levels between low-grade and high-grade PSCC or the different stages of ISCC, i.e., TN0, TNx and Nx ISCC, remain rather indistinguishable. This seems to point to a great leap of significantly changed gene expression at or after the transition of PSCC to ISCC rather than a gradual change in gene expression that would match the previously described stepwise accumulation of chromosome instability in PSCC and ISCC. Nevertheless, despite these obvious molecular differences found between PSCC and ISCC, we were able to identify abnormal gene expression shared between both. Moreover, cluster analysis of this shared abnormal gene expression revealed that the majority of these genes are either incrementally changed across PSCC and ISCC or exclusively altered in ISCC.

The pathways identified by IPA for genes gradually up regulated across PSCC and ISCC encompass changes to biological functions that are reminiscent of malignant cells. ‘G2/M cell cycle checkpoint regulation’, ‘p53 signaling’, ‘GADD signaling’ or ‘Polo-like kinase signaling’ can be associated with DNA damage and stress reactions in response to a chronic exposure to tobacco fumes or other volatile carcinogens [25, 27, 34–36]. The observed activations of ‘ERK/MAPK Signaling’ (z-score: 2.236) or ‘PI3K/AKT Signaling’ (z-score: 2.449) are common in many different cancers and may gradually enhance survival and proliferation of preinvasive and invasive cells in the included samples [28]. In addition, the incremental down regulation of the cytochrome P450 genes *CYP2B6* and *CYP4B1* in PSCC and ISCC suggest a reduced catabolism of inhaled carcinogens, such as those from tobacco fumes, which might in turn accelerate their detrimental effects in bronchial epithelia and preinvasive squamous tumors [37]. Hence, the combination of increased genetic injury, modifications of the G2/M checkpoint regulation and early changes of signalling cascades that underpin cell survival and proliferation could provide a possible explanation for the occurrence and propagation of chromosomal rearrangements in PSCC. *PTTG1*, which is a known proto-oncogene that under normal conditions acts as a securin to regulate chromatid separation during mitosis, has been found up-regulated in colorectal, thyroid and skin cancer where it is suggested to cause genetic instability and an increase in aneuploidy [29]. Hence, the observed increase of *PTTG1* on the mRNA and protein level suggests a similar function of this protein in invasive squamous carcinomas. Moreover, the observed shift to a pronounced nuclear localization in ISCC confirms a previous observation in aggressive-invasive subtypes of pituitary tumors (prolactin) that suggest a crucial role for PTTG1 during squamous carcinogenesis [38].

Genes up regulated in ISCC (cluster 11 in Fig. 2b) are enriched with a subset of IPA pathways, e.g., ‘leukocyte extravasation signaling’ (z-score: 1.265), ‘granulocyte adhesion and diapedesis’, or ‘IL-8 Signaling’ (z-score: 1) that suggest inflammation-related, microenvironmental processes such as the infiltration of immune cells, rearrangements of the connective tissue by metalloproteases or chemokine signaling in invasive squamous tumors (Fig. 3 and Additional file 2: Table S10). It is understood that such a local inflammation may contribute to tumor growth by the initiation of tumor vascularization, the local supply of growth stimuli, modifications of the immune response towards tumor cells and might eventually facilitate tumor metastasis [39, 40]. Interestingly, abnormal up regulation of chemokine genes such as *CXCL1*, *CXCL8*, *CXCL9* or *CXCL10* is also frequently detected in pre-invasive tumors, which might be indicative of a response to microbiological infections or an earlier onset of the inflammation-related processes observed in ISCC samples.

## Conclusions

Despite the known challenges to molecular studies that come with the use of FFPE material, we believe that our transcriptome analysis of preinvasive and invasive squamous FFPE tumor samples from the lower airways provides valuable insights into the genomic changes that occur during squamous carcinogenesis. The analysis of our Human Exon 1.0 ST array data confirms the previously published prevalence of segmental amplifications on chromosome 3q through enrichment analysis of abnormal transcription in PSCC and ISCC, and extends this analysis to all chromosomes (apart Y), which suggests further changes to chromosomal structures and consequently changes to gene expression being present in PSCC and ISCC. In addition, the functional analysis of this array data by IPA revealed alterations of biological functions in PSCC that confirm and extend recent findings [11, 22, 33]. The included PSCC and ISCC samples were not longitudinally harvested from the same bronchial lesion and are therefore unlikely to be clonally related. Nevertheless, we were able to identify common patterns of aberrant transcription during squamous carcinogenesis by sorting genes according to their abnormal expression profiles in PSCC and ISCC and associate these genes with biological functions related to developing malignancies. Consequently, we can propose alterations to cell cycle checkpoint regulation, DNA damage response and, among other signal transduction cascades, PI3K/AKT signaling as early events during squamous carcinogenesis, and suggest the up-regulations and nuclear localization of PTTG1 as a novel biomarker for ISCC.

## Additional files

Additional file 1:
**Figure S1.** Laser microdissection of FFPE PSCC and ISCC. (DOC 2651 kb)
Additional file 2:
**Table S1.** RT-PCR primer. **Table S2.** Patient and sample information. **Table S3.** Differential RNA expression in ISCC versus paired controls (normal histology). **Table S4.** Differential RNA expression in PSCC versus paired controls. **Table S5.** Differential RNA expression in PSCC and ISCC. **Table S6.** Differential RNA expression in high-grade PSCC versus paired controls. **Table S7.** Differential RNA expression in low-grade PSCC versus paired controls. **Table S8.** Differential RNA expression in high-grade PSCC and low-grade PSCC. **Table S9.** Ingenuity Pathway Analysis. **Table S10.** IPA results for genes associated with Clusters 1-12. **Table S11.** RNA expression profiles across PSCC and ISCC. (DOC 5260 kb)

## Abbreviations

cDNA: Complementary DNA
CIS: Carcinoma in-situ
DNA: Deoxyribonucleic acid
FFPE: Formalin fixed and paraffin embedded
GSEA: Gene set enrichment analysis
IPA: Ingenuity pathways analysis
ISCC: Invasive squamous cell carcinoma
LCM: Laser capture microscopy
lncRNA: Long non-coding RNA
Nx: Lymph node metastasis
PCA: Principal component analysis
PSCC: Preinvasive squamous cell carcinoma
RNA: Ribonucleic acid
RT-PCR: Real-time PCR
TN0: Lymph node-negative
TNx: Lymph node-positive
