# Supplementary material for: Whole Transcriptome Analysis of Pre-invasive and Invasive Early Squamous Lung Carcinoma in Archival Laser Microdissected Samples
Source: Respir Res. 2017 Jan 10;18:12. doi: 10.1186/s12931-016-0496-3 (PMC5223343; doi:10.1186/s12931-016-0496-3)
Supplement: Additional file 1: — Figure S1. Laser microdissection of FFPE PSCC and ISCC. (DOC 2651 kb) [file 12931_2016_496_MOESM1_ESM.doc]

**Supplemental Figure 1.** Laser microdissection of FFPE PSCC and ISCC.


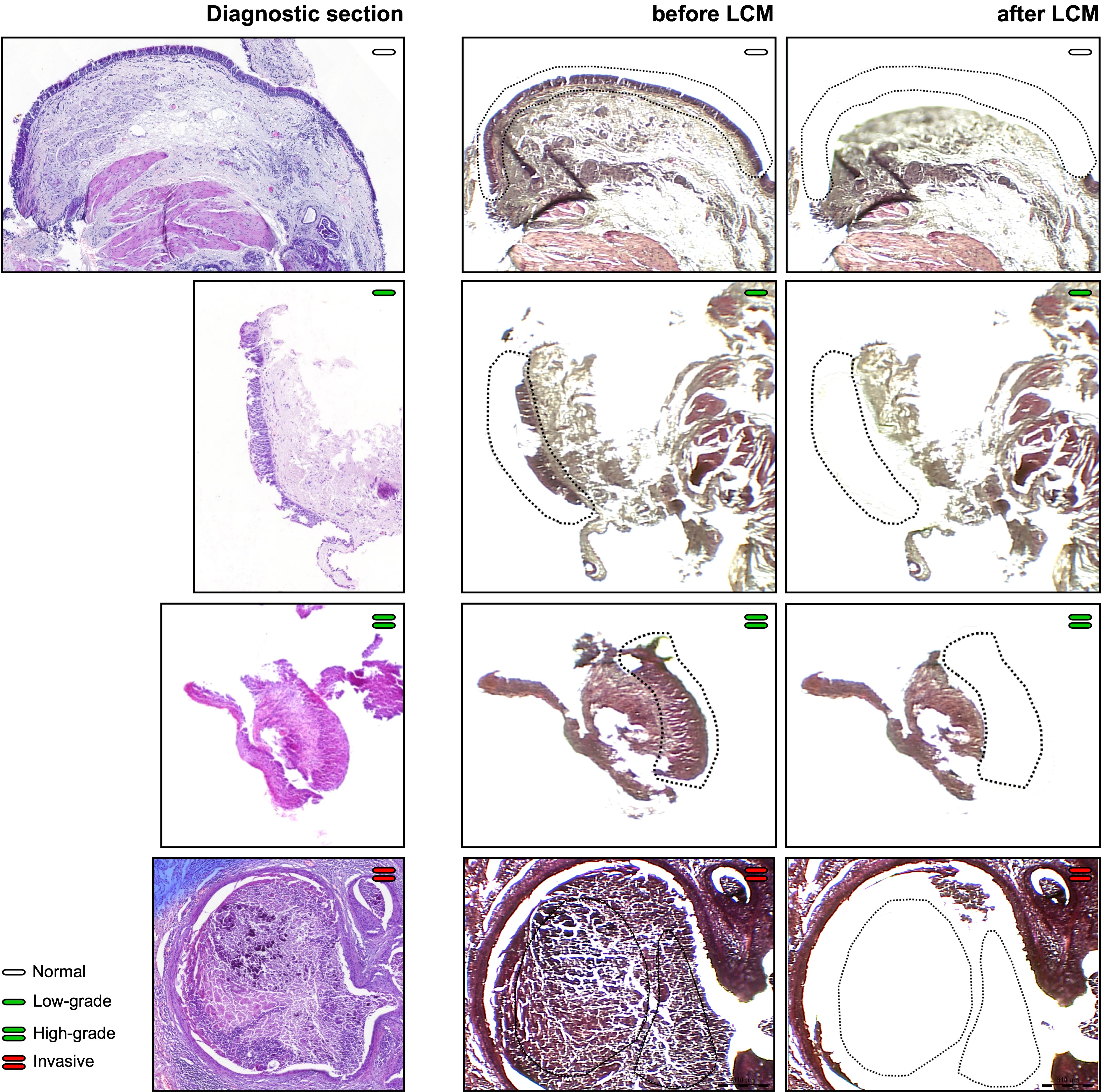


**Suppl. Fig.1.** Overview of the microdissection procedure. Image rows display examples of normal lung mucosa, low-grade PSCC, high-grade PSCC and ISCC. Image columns represent from left to right examples of diagnostic sections and a corresponding section that underwent microdissections. Diagnostic sections were stained with Hematoxylin and Eosin and independently reviewed by three specialist thoracic pathologists who designated areas of interest.
