# Supplementary material for: Whole Transcriptome Analysis of Pre-invasive and Invasive Early Squamous Lung Carcinoma in Archival Laser Microdissected Samples
Source: Respir Res. 2017 Jan 10;18:12. doi: 10.1186/s12931-016-0496-3 (PMC5223343; doi:10.1186/s12931-016-0496-3)
Supplement: Additional file 2: — Table S1. RT-PCR primer. Table S2. Patient and sample information. Table S3. Differential RNA expression in ISCC versus paired controls (normal histology). Table S4. Differential RNA expression in PSCC versus paired controls. Table S5. Differential RNA expression in PSCC and ISCC. Table S6. Differential RNA expression in high-grade PSCC versus paired controls. Table S7. Differential RNA expression in low-grade PSCC versus paired controls. Table S8. Differential RNA expression in high-grade PSCC and low-grade PSCC. Table S9. Ingenuity Pathway Analysis. Table S10. IPA results for genes associated with Clusters 1-12. Table S11. RNA expression profiles across PSCC and ISCC. (DOC 5260 kb) [file 12931_2016_496_MOESM2_ESM.doc]

Additional file 1. Table S1. RT-PCR primer.

| **Name** | **Sequence (5’>3’)** | **Template strand** | **Product length** | **Target** |
| --- | --- | --- | --- | --- |
| FN1-Forward | AGCAAGCCCGGTTGTTATGA | Plus | 185 | Fibronectin 1 (FN1), transcript variant 1 |
| FN1-Reverse | CCCACTCGGTAAGTGTTCCC | Minus |
| HIF-Forward | TCAAGCAGTAGGAATTGGAACA | Plus | 178 | Hypoxia inducible factor 1, alpha subunit (HIF1A), transcript variant 1 |
| HIF-Reverse | ATCCATTGATTGCCCCAGCA | Minus |
| ITG-Forward | ATGCTCCATGTAGATCACAAGAT | Plus | 114 | Integrin, alpha V (ITGAV), transcript variant 1 |
| ITG-Reverse | AGCTACCAGGACCACCAAGA | Minus |
| PTT-Forward | CCAAGGGACCCCTCAAACAA | Plus | 148 | Pituitary tumor-transforming 1 (PTTG1), transcript variant 1 |
| PTT-Reverse | TCAAAGTCTAGAGGATTGAAGGGA | Minus |
| 28S-Forward | CGGTACACCTGTCAAACGGTAA | Plus | 75 | 28S ribosomal 1 (RN28S1) |
| 28S-Reverse | TCTGCTCCACGGGAGGTT | Minus |

Additional file 2. Table S2. Patient and sample information.

| **Patient ID** | **Age at sampling** | **Gender** | **Control samples (normal histology)** | **PSCC low-grade samples** | **PSCC high-grade samples** | **Lymph node negative ISCC samples (TN0)** | **Lymph node positive ISCC samples (TNx)** | **Lymph node invasion samples (Nx)** |
| --- | --- | --- | --- | --- | --- | --- | --- | --- |
| P1 | 61 | M | RMB 2006 (P1-N) | subcarina lingular 2006 (P1-2) | Lingula 2006 (P1-1) | - | - | - |
| P2 | 60 | M | LMB 2005 (P2-N) | RMB 2005 (P2-2) | RMB 2005 (P2-1) | - | - | - |
| P3 | 67 | M | RUL 2004 (P3-N) | LUL 2003 (P3-2) | LUL 2004 (P3-1) | - | - | - |
| P4 | 57 | M | left basal spur 2003 (P4-N) | right apical lower spur 2003 (P4-1) | - | - | - | - |
| P5 | 67 | M | left apial lower bifurcation 2003 (P5-N) | - | left basal spur 2003 (P5-1) | - | - | - |
| P6 | 60 | M | LSC 2000 (P6-N) | LUL 2000 (P6-2) | LS 2000 (P6-1) | - | - | - |
| P7 | 67 | M | RUL 2012 (P7-N) | RUL 2012 (P7-1) | - | - | - | - |
| P8 | 45 | F | RUL 2013 (P8-N) | LSC 2013 (P8-1) | - | - | - | - |
| P9 | 65 | M | RUL 2012 (P9-N) | - | - | - | RUL 2012 (P9-1) | R4 2012 (P9-2) |
| P10 | 69 | M | LUL 2006 (P10-N) | - | RML 2006 (P10-1) | - | - | - |
| P11 | 60 | M | ML spur 2006 (P11-N) | Main carina 2006 (P11-1) | - | - | - | - |
| P12 | 71 | F | LLL 2012 (P12-N) | - | - | LLL 2012 (P12-1) | - | - |
| P13 | 65 | M | RUL 2012 (P13-N) | - | - | - | RUL 2012 (P13-1) | R4 2012 (P13-2) |
| P14 | 68 | F | main carina, 2004 (P14-N) | LMB 2005 (P14-1) | - | - | - | - |
| P15 | 76 | F | LUL 2012 (P15-N) | - | - | LUL 2012 (P15-1) | - | - |
| P16 | 65 | M | RML 2012 (P16-N) | - | - | - | RML 2012 (P16-1) | R4 2012 (P16-2) |
| P17 | 60 | M | RUL 1999 (P17-N) | Right apical lower 1999 (P17-2) | lateral wall Br 2000 (P17-1) | - | - | - |
| P18 | 61 | M | RUL 2000 (P18-N) | - | RMB 2000 (P18-1) | - | - | - |
| P19 | 79 | M | RLL 2012 (P19-N) | - | - | RLL 2012 (P19-1) | - | - |
| P20 | 57 | M | LUL 2011 (P20-N) | - | - | LUL 2011 (P20-1) | - | - |
| P21 | 77 | F | LUL 2011 (P21-N) | - | - | LUL 2011 (P21-1) | - | - |
| P22 | 51 | F | LUL 2013 (P22-N) | RUL 2013 (P22-1) | - | - | - | - |
| P23 | 74 | F | roof of left upper divison 2002 (P23-N) | RUL 2002 (P23-1) | - | - | - | - |
| P24 | 61 | F | RUL 2012 (P24-N) | - | - | - | RUL 2012 (P24-1) | R4 2012 (P24-2) |
| P25 | 72 | M | LUL 2012 (P25-N) | - | - | - | LUL 2012 (P25-1) | R7 2012 (P25-2) |

Additional file 3. Table S3. Differential RNA expression in ISCC versus paired controls (normal histology).

| **Gene symbol** | **RefSeq** | **Mean ISCC** | **Mean control** | **Ratio** | **Fold change** | **P-value** | **Q-value** |
| --- | --- | --- | --- | --- | --- | --- | --- |
| SPP1 | NM_001251830 | 276.9611865 | 23.27388261 | 11.9 | 11.9 | 5.06E-07 | 5.08E-05 |
| SPRR2A | NM_005988 | 100.522873 | 14.77247856 | 6.80474 | 6.80474 | 0.03037113 | 0.06566052 |
| SCD | NM_005063 | 224.3955102 | 36.83384246 | 6.09213 | 6.09213 | 3.93E-05 | 0.000646791 |
| HIST1H3C | NM_003531 | 152.1984652 | 25.30955085 | 6.01347 | 6.01347 | 0.000911773 | 0.005788771 |
| RRM2 | ENST00000360566 | 190.7236578 | 33.17236577 | 5.74948 | 5.74948 | 1.23E-06 | 7.02E-05 |
| FN1 | ENST00000354785 | 325.630043 | 57.32966724 | 5.67995 | 5.67995 | 0.001447589 | 0.007945185 |
| HIST2H2AB | NM_175065 | 277.8938314 | 49.3435634 | 5.63181 | 5.63181 | 1.04E-06 | 6.49E-05 |
| HIST1H3F | NM_021018 | 92.78887902 | 19.12436579 | 4.85185 | 4.85185 | 1.30E-05 | 0.000301181 |
| GPNMB | NM_001005340 | 254.9640446 | 54.34781013 | 4.69134 | 4.69134 | 7.78E-06 | 0.000215959 |
| TOP2A | NM_001067 | 45.22661137 | 9.921030627 | 4.55863 | 4.55863 | 2.91E-06 | 0.000117446 |
| COL1A1 | ENST00000225964 | 307.013865 | 67.50956417 | 4.54771 | 4.54771 | 0.003716993 | 0.01542966 |
| MAD2L1 | ENST00000296509 | 66.04465941 | 14.82284017 | 4.45559 | 4.45559 | 0.000200449 | 0.002027895 |
| HIST1H4B | NM_003544 | 50.48104953 | 11.82550591 | 4.26883 | 4.26883 | 2.94E-09 | 3.36E-05 |
| CKS2 | NM_001827 | 68.80111307 | 16.16240517 | 4.25687 | 4.25687 | 0.000150599 | 0.001650765 |
| GREM1 | ENST00000300177 | 112.1604319 | 26.35838367 | 4.25519 | 4.25519 | 0.002458133 | 0.01153495 |
| PTTG1 | ENST00000352433 | 181.7485444 | 43.82007353 | 4.14761 | 4.14761 | 2.05E-05 | 0.000407209 |
| LUM | ENST00000266718 | 158.1719537 | 38.73591732 | 4.08336 | 4.08336 | 0.009210789 | 0.02936168 |
| SPARC | NM_003118 | 445.3266067 | 113.4199016 | 3.92636 | 3.92636 | 0.003498946 | 0.01473629 |
| GJA1 | NM_000165 | 314.6490987 | 81.89934451 | 3.8419 | 3.8419 | 0.000186271 | 0.001920155 |
| TPX2 | NM_012112 | 50.85437538 | 13.94194562 | 3.64759 | 3.64759 | 2.72E-06 | 0.000112971 |
| COL3A1 | ENST00000304636 | 199.3983968 | 55.60104142 | 3.58621 | 3.58621 | 0.009076203 | 0.02904614 |
| SLC16A1 | NM_001166496 | 55.39677173 | 15.50756213 | 3.57225 | 3.57225 | 0.000101604 | 0.001262775 |
| SULF1 | NM_001128205 | 69.26141708 | 19.55492368 | 3.54187 | 3.54187 | 0.001022389 | 0.006277106 |
| MKI67 | ENST00000368654 | 41.55326689 | 11.7928458 | 3.52361 | 3.52361 | 1.93E-06 | 8.96E-05 |
| SMC4 | NM_005496 | 66.33093697 | 18.96569059 | 3.49742 | 3.49742 | 1.38E-06 | 7.38E-05 |
| STMN1 | NM_203401 | 80.32348724 | 23.0553554 | 3.48394 | 3.48394 | 5.74E-06 | 0.000176238 |
| RPL35 | CR456877 | 68.99213495 | 19.87220422 | 3.47178 | 3.47178 | 0.002491906 | 0.01161706 |
| PRC1 | ENST00000361188 | 38.68842251 | 11.41342593 | 3.38972 | 3.38972 | 2.43E-06 | 0.00010395 |
| KIAA0101 | ENST00000380258 | 20.55731585 | 6.124661029 | 3.35648 | 3.35648 | 1.74E-05 | 0.000365411 |
| RCN1 | NM_002901 | 64.87811508 | 19.42536767 | 3.33988 | 3.33988 | 0.0472636 | 0.08753734 |
| CDKN3 | NM_005192 | 38.60323909 | 11.59089448 | 3.3305 | 3.3305 | 6.65E-06 | 0.000194257 |
| ARL6IP1 | ENST00000304414 | 187.1174116 | 56.3037848 | 3.32336 | 3.32336 | 0.000225005 | 0.002181613 |
| GJB6 | NM_001110219 | 56.17592227 | 16.94467428 | 3.31525 | 3.31525 | 0.008173845 | 0.02700585 |
| COL1A2 | NM_000089 | 224.2477967 | 67.65525068 | 3.31457 | 3.31457 | 0.01251927 | 0.0362738 |
| UCHL1 | NM_004181 | 67.07810373 | 20.4481724 | 3.2804 | 3.2804 | 0.000406876 | 0.003307627 |
| VCAN | NM_004385 | 86.19187662 | 26.52976516 | 3.24887 | 3.24887 | 0.001085353 | 0.006497769 |
| THY1 | ENST00000284240 | 137.540244 | 42.37231362 | 3.24601 | 3.24601 | 0.004233715 | 0.01689006 |
| COL12A1 | NM_004370 | 50.95917443 | 15.8751685 | 3.20997 | 3.20997 | 0.04442906 | 0.08414119 |
| H2AFZ | ENST00000296417 | 92.51337171 | 29.26369421 | 3.16138 | 3.16138 | 6.19E-05 | 0.000892681 |
| FXR1 | NM_001013439 | 123.3710428 | 39.24263469 | 3.1438 | 3.1438 | 4.01E-05 | 0.000656175 |
| COL6A3 | NM_004369 | 159.2103151 | 50.72728352 | 3.13855 | 3.13855 | 0.009715436 | 0.03033538 |
| CENPF | NM_016343 | 30.58240586 | 9.751607101 | 3.13612 | 3.13612 | 3.31E-06 | 0.000126019 |
| TBL1XR1 | ENST00000430069 | 142.8700793 | 45.8344355 | 3.11711 | 3.11711 | 2.23E-05 | 0.000431702 |
| DSC2 | NM_024422 | 51.05640313 | 16.41132708 | 3.11106 | 3.11106 | 1.39E-06 | 7.38E-05 |
| CTHRC1 | NM_138455 | 43.71510624 | 14.0810206 | 3.10456 | 3.10456 | 0.000252251 | 0.002351948 |
| GPI | NM_000175 | 212.5119266 | 68.53932126 | 3.10059 | 3.10059 | 1.80E-06 | 8.47E-05 |
| PIGX | NM_001166304 | 39.58412346 | 12.8757985 | 3.0743 | 3.0743 | 0.000683907 | 0.004742794 |
| TUBB | ENST00000421473 | 641.2841768 | 208.9957797 | 3.0684 | 3.0684 | 3.79E-06 | 0.0001357 |
| DSG3 | NM_001944 | 53.64121462 | 17.78772376 | 3.01562 | 3.01562 | 0.002054971 | 0.01023159 |
| PGK1 | NM_000291 | 402.6437139 | 134.4242861 | 2.99532 | 2.99532 | 1.26E-07 | 4.84E-05 |
| ACTL6A | NM_178042 | 66.27349048 | 22.25997396 | 2.97726 | 2.97726 | 3.94E-06 | 0.000138894 |
| DSG2 | NM_001943 | 80.78692987 | 27.2746731 | 2.96197 | 2.96197 | 0.000451021 | 0.003538068 |
| ANLN | NM_018685 | 34.90592408 | 11.86606759 | 2.94166 | 2.94166 | 9.90E-05 | 0.001246576 |
| CALU | NM_001199671 | 66.13719709 | 22.49184337 | 2.94049 | 2.94049 | 1.04E-05 | 0.000260495 |
| HIST1H4C | NM_003542 | 212.9070629 | 72.61773364 | 2.9319 | 2.9319 | 9.52E-05 | 0.001212203 |
| UBE2T | ENST00000367274 | 44.23419 | 15.13272608 | 2.92308 | 2.92308 | 1.88E-05 | 0.000385508 |
| MRPL51 | ENST00000229238 | 198.6644607 | 68.01966521 | 2.92067 | 2.92067 | 0.001010917 | 0.006227828 |
| PLOD2 | NM_182943 | 39.15053182 | 13.49459711 | 2.9012 | 2.9012 | 1.23E-05 | 0.000293292 |
| FOXM1 | NM_202002 | 62.31985623 | 21.50037623 | 2.89856 | 2.89856 | 4.06E-06 | 0.000141378 |
| ODC1 | ENST00000234111 | 170.0281724 | 59.27163708 | 2.86864 | 2.86864 | 0.000455056 | 0.003562381 |
| NDRG1 | ENST00000414097 | 211.4041664 | 73.74891912 | 2.86654 | 2.86654 | 0.000276665 | 0.002517916 |
| HIST1H2BM | NM_003521 | 84.85689589 | 29.6499959 | 2.86194 | 2.86194 | 0.000160549 | 0.001731422 |
| DTL | ENST00000366991 | 26.76322374 | 9.367213839 | 2.85713 | 2.85713 | 8.16E-07 | 6.07E-05 |
| SLC44A1 | NM_080546 | 61.09151022 | 21.39675335 | 2.85519 | 2.85519 | 5.42E-05 | 0.000813477 |
| GINS1 | NM_021067 | 28.27862366 | 9.924263219 | 2.84944 | 2.84944 | 4.45E-07 | 5.08E-05 |
| PRR11 | NM_018304 | 25.02867478 | 8.814324436 | 2.83956 | 2.83956 | 3.52E-05 | 0.000599171 |
| DCUN1D1 | ENST00000292782 | 288.8786954 | 102.1729863 | 2.82734 | 2.82734 | 0.000301055 | 0.002659959 |
| RBP1 | ENST00000483943 | 89.42956789 | 31.64224376 | 2.82626 | 2.82626 | 0.00136716 | 0.007651488 |
| NUSAP1 | NM_016359 | 30.07035511 | 10.68400018 | 2.81452 | 2.81452 | 1.42E-07 | 4.84E-05 |
| HIST1H3I | NM_003533 | 30.61061234 | 10.9041847 | 2.80723 | 2.80723 | 0.004067643 | 0.01642252 |
| ECT2 | NM_001258316 | 23.31473295 | 8.396685779 | 2.77665 | 2.77665 | 0.00010904 | 0.001332003 |
| FSCN1 | NM_003088 | 84.3712751 | 30.49942206 | 2.76634 | 2.76634 | 0.000268452 | 0.002465747 |
| DNAJB11 | ENST00000439351 | 100.8613758 | 36.49229551 | 2.7639 | 2.7639 | 3.67E-05 | 0.000615531 |
| CDC20 | ENST00000372462 | 39.61733691 | 14.42610576 | 2.74621 | 2.74621 | 0.001015616 | 0.006250034 |
| ARHGAP11A | ENST00000361627 | 15.30933806 | 5.578433305 | 2.74438 | 2.74438 | 9.50E-08 | 4.84E-05 |
| CCT5 | ENST00000280326 | 210.4582129 | 76.8796573 | 2.7375 | 2.7375 | 0.000127203 | 0.001472011 |
| NTRK2 | NM_006180 | 46.13372883 | 17.0440939 | 2.70673 | 2.70673 | 0.000358644 | 0.003016725 |
| ENO1 | NM_001428 | 295.4581531 | 109.3652049 | 2.70156 | 2.70156 | 2.37E-05 | 0.000453424 |
| HIST1H2AE | NM_021052 | 138.4258912 | 51.69167333 | 2.6779 | 2.6779 | 0.004162159 | 0.01667331 |
| THBS2 | NM_003247 | 96.79093282 | 36.2600749 | 2.66936 | 2.66936 | 0.004030358 | 0.01633657 |
| PA2G4 | NM_006191 | 705.1908767 | 265.597913 | 2.65511 | 2.65511 | 9.40E-06 | 0.000244009 |
| CKAP2 | NM_018204 | 33.74215005 | 12.75543328 | 2.64531 | 2.64531 | 1.12E-05 | 0.000273925 |
| MMP12 | ENST00000532855 | 29.75169586 | 11.32956057 | 2.62602 | 2.62602 | 6.76E-05 | 0.000945292 |
| CDC6 | ENST00000209728 | 17.85269535 | 6.801344648 | 2.62489 | 2.62489 | 6.73E-07 | 5.61E-05 |
| HMGCS1 | NM_001098272 | 69.8641288 | 26.64513061 | 2.62202 | 2.62202 | 0.008058864 | 0.02676354 |
| TFRC | NM_003234 | 92.24315798 | 35.19430277 | 2.62097 | 2.62097 | 0.000123018 | 0.001444065 |
| GOLIM4 | ENST00000470487 | 72.22968632 | 27.80610953 | 2.59763 | 2.59763 | 2.69E-05 | 0.000498772 |
| YEATS2 | NM_018023 | 71.75562373 | 27.64180651 | 2.5959 | 2.5959 | 1.02E-05 | 0.000257746 |
| PKM | NM_001206796 | 286.3428957 | 110.4944426 | 2.59146 | 2.59146 | 4.94E-05 | 0.000752309 |
| RPL39L | ENST00000296277 | 97.15996061 | 37.61874983 | 2.58277 | 2.58277 | 0.000459336 | 0.003587297 |
| FKBP4 | ENST00000001008 | 111.7228135 | 43.3653245 | 2.57631 | 2.57631 | 0.000278175 | 0.002521613 |
| C8orf59 | ENST00000518786 | 173.408403 | 67.7045084 | 2.56126 | 2.56126 | 0.000310175 | 0.002711959 |
| ASPM | NM_018136 | 18.35854732 | 7.201862223 | 2.54915 | 2.54915 | 5.18E-07 | 5.08E-05 |
| MTHFD2 | NR_027405 | 93.36112238 | 36.84099191 | 2.53417 | 2.53417 | 0.000143203 | 0.001592513 |
| ITGA6 | NM_000210 | 69.68083466 | 27.55438458 | 2.52884 | 2.52884 | 0.00269239 | 0.01221759 |
| HIST1H4L | NM_003546 | 23.60924498 | 9.377608174 | 2.51761 | 2.51761 | 0.000623032 | 0.004447554 |
| DLGAP5 | NM_001146015 | 16.13006112 | 6.409980642 | 2.51639 | 2.51639 | 5.40E-06 | 0.00016991 |
| ADAM23 | ENST00000264377 | 31.35774694 | 12.49726072 | 2.50917 | 2.50917 | 0.001317763 | 0.007475164 |
| ANAPC13 | NM_001242374 | 62.62730966 | 24.97287473 | 2.50781 | 2.50781 | 0.001762364 | 0.009174656 |
| HIST1H2BK | NM_080593 | 164.6946636 | 65.94676568 | 2.49738 | 2.49738 | 0.03382975 | 0.070484 |
| DLX5 | ENST00000222598 | 37.57627104 | 15.10767763 | 2.48723 | 2.48723 | 0.000498505 | 0.003806 |
| ZNF131 | NM_003432 | 42.30716181 | 17.06868488 | 2.47865 | 2.47865 | 8.35E-06 | 0.000226284 |
| ASPN | NM_017680 | 59.1784498 | 23.88081221 | 2.47808 | 2.47808 | 0.03326973 | 0.06975803 |
| FADS2 | ENST00000278840 | 44.52519424 | 18.04778649 | 2.46706 | 2.46706 | 0.001163902 | 0.006853844 |
| DEPDC1 | NM_001114120 | 16.15277352 | 6.584664799 | 2.4531 | 2.4531 | 4.50E-05 | 0.000708933 |
| ITGAV | NM_002210 | 98.05368874 | 40.01835612 | 2.45022 | 2.45022 | 0.000199862 | 0.002025523 |
| HPRT1 | NM_000194 | 29.97442139 | 12.24550189 | 2.44778 | 2.44778 | 0.000384683 | 0.003167797 |
| IGF2BP3 | NM_006547 | 41.67239176 | 17.06347999 | 2.4422 | 2.4422 | 0.000239237 | 0.002277078 |
| PDK1 | NM_002610 | 45.1336015 | 18.51265616 | 2.43799 | 2.43799 | 4.12E-06 | 0.000142167 |
| GATC | NM_176818 | 213.0768429 | 87.61524411 | 2.43196 | 2.43196 | 0.001098238 | 0.006560537 |
| ILF2 | NM_004515 | 102.7952649 | 42.29983117 | 2.43016 | 2.43016 | 2.16E-07 | 4.98E-05 |
| MSANTD3 | NM_001198807 | 93.31777474 | 38.40573887 | 2.42978 | 2.42978 | 0.000351267 | 0.002972654 |
| NCAPD2 | ENST00000315579 | 48.77804393 | 20.11374753 | 2.4251 | 2.4251 | 6.38E-05 | 0.000906349 |
| SRPRB | NM_021203 | 106.5494991 | 43.95788291 | 2.4239 | 2.4239 | 5.88E-05 | 0.000859918 |
| MLLT11 | ENST00000368921 | 48.88025817 | 20.24578953 | 2.41435 | 2.41435 | 1.64E-05 | 0.000351437 |
| BGN | NM_001711 | 319.326838 | 132.8173676 | 2.40426 | 2.40426 | 0.0214316 | 0.05197621 |
| RPF2 | ENST00000441448 | 88.71400678 | 36.90846927 | 2.40362 | 2.40362 | 0.01471205 | 0.04042258 |
| TARS | NR_047676 | 41.53224636 | 17.30587521 | 2.3999 | 2.3999 | 1.81E-05 | 0.000374516 |
| PSMD2 | ENST00000310118 | 88.77244329 | 37.07153633 | 2.39463 | 2.39463 | 5.97E-05 | 0.000868631 |
| PSMD11 | ENST00000261712 | 84.54162863 | 35.33240381 | 2.39275 | 2.39275 | 2.42E-05 | 0.000461444 |
| NETO2 | ENST00000562435 | 28.06382983 | 11.76100955 | 2.38619 | 2.38619 | 0.000373321 | 0.00309986 |
| NMD3 | ENST00000351193 | 71.26293511 | 29.8957811 | 2.38372 | 2.38372 | 1.49E-05 | 0.000328539 |
| SERPINH1 | ENST00000524558 | 93.55092414 | 39.32486751 | 2.37893 | 2.37893 | 0.000210894 | 0.002084006 |
| CEP55 | ENST00000371485 | 20.15393998 | 8.471926071 | 2.37892 | 2.37892 | 1.28E-05 | 0.000301181 |
| BUB1B | ENST00000287598 | 22.71872553 | 9.55714694 | 2.37713 | 2.37713 | 2.80E-06 | 0.000115039 |
| PLK1 | ENST00000300093 | 43.1434607 | 18.16576227 | 2.375 | 2.375 | 6.93E-05 | 0.000960587 |
| EIF4A2 | NM_001967 | 59.71286975 | 25.15441965 | 2.37385 | 2.37385 | 8.13E-05 | 0.001078495 |
| GGCT | NM_024051 | 76.54519964 | 32.48428397 | 2.35638 | 2.35638 | 5.49E-05 | 0.000820748 |
| KDELR2 | NM_006854 | 157.5504429 | 66.91788763 | 2.35439 | 2.35439 | 0.001019263 | 0.006262355 |
| FNDC3B | NM_022763 | 90.17777444 | 38.38551236 | 2.34926 | 2.34926 | 3.62E-05 | 0.000610732 |
| PLS3 | NM_005032 | 104.1493482 | 44.38529867 | 2.34649 | 2.34649 | 0.000201078 | 0.002027895 |
| NDC80 | NM_006101 | 11.93908195 | 5.088710084 | 2.34618 | 2.34618 | 4.76E-06 | 0.000157007 |
| SHFM1 | ENST00000482389 | 569.3097511 | 242.6864921 | 2.34586 | 2.34586 | 0.000101455 | 0.001262775 |
| CCNB1 | NM_031966 | 31.48144812 | 13.4346795 | 2.34328 | 2.34328 | 1.77E-05 | 0.000368241 |
| SLC7A5 | NM_003486 | 50.83640127 | 21.71846145 | 2.34071 | 2.34071 | 2.31E-06 | 9.96E-05 |
| FZD7 | NM_003507 | 43.02490197 | 18.3857994 | 2.34012 | 2.34012 | 7.41E-06 | 0.000210012 |
| MRPL3 | NM_007208 | 214.2319729 | 91.63075491 | 2.33799 | 2.33799 | 0.001343769 | 0.007560655 |
| CSE1L | NM_001316 | 60.53635897 | 25.90251001 | 2.33708 | 2.33708 | 0.000136415 | 0.001549207 |
| HIF1A | NM_001530 | 111.3463148 | 47.67860102 | 2.33536 | 2.33536 | 0.000601749 | 0.004352753 |
| SPP2 | NM_001251830 | 77.77665283 | 41.31350318 | 1.715579124 | 1.715579124 | 1.24E-03 | 4.29E-03 |
| SPRR2A | NM_005988 | 77.25392732 | 41.36688724 | 1.695739592 | 1.695739592 | 0.001221752 | 0.004248881 |
| SCD | NM_005063 | 76.73120181 | 41.4202713 | 1.675900059 | 1.675900059 | 1.20E-03 | 0.004208092 |
| HIST1H3C | NM_003531 | 76.2084763 | 41.47365536 | 1.656060527 | 1.656060527 | 0.001186773 | 0.004167303 |
| RRM3 | ENST00000349004 | 75.6857508 | 41.52703941 | 1.636220994 | 1.636220994 | 1.17E-03 | 4.13E-03 |
| FN2 | ENST00000343223 | 75.16302529 | 41.58042347 | 1.616381461 | 1.616381461 | 0.001151795 | 0.004085724 |
| HIST2H2AB | NM_175065 | 74.64029978 | 41.63380753 | 1.596541929 | 1.596541929 | 1.13E-03 | 4.04E-03 |
| HIST1H3F | NM_021018 | 74.11757427 | 41.68719159 | 1.576702396 | 1.576702396 | 1.12E-03 | 0.004004145 |
| GPNMB | NM_001005340 | 73.59484877 | 41.74057565 | 1.556862864 | 1.556862864 | 1.10E-03 | 0.003963356 |
| TOP2A | NM_001067 | 73.07212326 | 41.79395971 | 1.537023331 | 1.537023331 | 1.08E-03 | 0.003922567 |
| COL1A2 | ENST00000367054 | 72.54939775 | 41.84734377 | 1.517183799 | 1.517183799 | 0.00106435 | 0.003881778 |
| MAD2L2 | ENST00000437599 | 72.02667224 | 41.90072783 | 1.497344266 | 1.497344266 | 0.001046861 | 0.003840988 |
| HIST1H4B | NM_000110 | 71.50394674 | 41.95411189 | 1.477504734 | 1.477504734 | 1.03E-03 | 3.80E-03 |
| CKS3 | NM_001607 | 70.98122123 | 42.00749595 | 1.457665201 | 1.457665201 | 0.001011883 | 0.00375941 |
| GREM2 | ENST00000300177 | 70.45849572 | 42.06088001 | 1.437825668 | 1.437825668 | 0.000994393 | 0.003718621 |
| PTTG2 | ENST00000352433 | 69.93577021 | 42.11426407 | 1.417986136 | 1.417986136 | 9.77E-04 | 0.003677831 |
| LUM | ENST00000266718 | 69.41304471 | 42.16764813 | 1.398146603 | 1.398146603 | 0.000959415 | 0.003637042 |
| SPARC | NM_003118 | 68.8903192 | 42.22103219 | 1.378307071 | 1.378307071 | 0.000941926 | 0.003596253 |
| GJA2 | NM_000165 | 68.36759369 | 42.27441625 | 1.358467538 | 1.358467538 | 0.000924437 | 0.003555463 |
| TPX3 | NM_012112 | 67.84486818 | 42.32780031 | 1.338628006 | 1.338628006 | 9.07E-04 | 0.003514674 |
| COL3A2 | ENST00000304637 | 67.32214268 | 42.38118437 | 1.318788473 | 1.318788473 | 0.000889459 | 0.003473885 |
| SLC16A2 | NM_001089914 | 66.79941717 | 42.43456842 | 1.298948941 | 1.298948941 | 0.00087197 | 0.003433096 |
| SULF2 | NM_001051623 | 66.27669166 | 42.48795248 | 1.279109408 | 1.279109408 | 0.000854481 | 0.003392306 |
| MKI68 | ENST00000368655 | 65.75396615 | 42.54133654 | 1.259269875 | 1.259269875 | 8.37E-04 | 3.35E-03 |
| SMC5 | NM_401306 | 65.23124065 | 42.5947206 | 1.239430343 | 1.239430343 | 8.20E-04 | 3.31E-03 |
| STMN2 | NM_599211 | 64.70851514 | 42.64810466 | 1.21959081 | 1.21959081 | 8.02E-04 | 0.003269938 |
| RPL36 | CR456878 | 64.18578963 | 42.70148872 | 1.199751278 | 1.199751278 | 0.000784524 | 0.003229149 |
| PRC2 | ENST00000399328 | 63.66306412 | 42.75487278 | 1.179911745 | 1.179911745 | 7.67E-04 | 0.00318836 |
| KIAA0102 | ENST00000418398 | 63.14033862 | 42.80825684 | 1.160072213 | 1.160072213 | 7.50E-04 | 0.003147571 |
| RCN2 | NM_007483 | 62.61761311 | 42.8616409 | 1.14023268 | 1.14023268 | 0.000732057 | 0.003106781 |
| CDKN4 | NM_009774 | 62.0948876 | 42.91502496 | 1.120393148 | 1.120393148 | 7.15E-04 | 0.003065992 |
| ARL6IP2 | ENST00000304415 | 61.57216209 | 42.96840902 | 1.100553615 | 1.100553615 | 0.000697079 | 0.003025203 |
| GJB7 | NM_001110219 | 61.04943658 | 43.02179308 | 1.080714082 | 1.080714082 | 0.00067959 | 0.002984414 |
| COL1A3 | NM_000089 | 60.52671108 | 43.07517714 | 1.06087455 | 1.06087455 | 0.000662101 | 0.002943624 |
| UCHL2 | NM_004181 | 60.00398557 | 43.1285612 | 1.041035017 | 1.041035017 | 0.000644612 | 0.002902835 |
| VCAN | NM_004385 | 59.48126006 | 43.18194526 | 1.021195485 | 1.021195485 | 0.000627123 | 0.002862046 |
| THY2 | ENST00000284241 | 58.95853455 | 43.23532932 | 1.001355952 | 1.001355952 | 0.000609634 | 0.002821256 |
| COL12A2 | NM_004371 | 58.43580905 | 43.28871338 | 0.98151642 | 0.98151642 | 0.000592144 | 0.002780467 |
| H2AFZ | ENST00000296418 | 57.91308354 | 43.34209743 | 0.961676887 | 0.961676887 | 5.75E-04 | 0.002739678 |
| FXR2 | NM_001013439 | 57.39035803 | 43.39548149 | 0.941837355 | 0.941837355 | 5.57E-04 | 0.002698889 |
| COL6A4 | NM_004369 | 56.86763252 | 43.44886555 | 0.921997822 | 0.921997822 | 0.000539677 | 0.002658099 |
| CENPF | NM_016343 | 56.34490702 | 43.50224961 | 0.90215829 | 0.90215829 | 5.22E-04 | 0.00261731 |
| TBL1XR2 | ENST00000430070 | 55.82218151 | 43.55563367 | 0.882318757 | 0.882318757 | 5.05E-04 | 0.002576521 |
| DSC3 | NM_024422 | 55.299456 | 43.60901773 | 0.862479224 | 0.862479224 | 4.87E-04 | 2.54E-03 |
| CTHRC2 | NM_138455 | 54.77673049 | 43.66240179 | 0.842639692 | 0.842639692 | 0.000469721 | 0.002494942 |
| GPI | NM_000175 | 54.25400499 | 43.71578585 | 0.822800159 | 0.822800159 | 4.52E-04 | 2.45E-03 |
| PIGX | NM_001166304 | 53.73127948 | 43.76916991 | 0.802960627 | 0.802960627 | 0.000434743 | 0.002413364 |
| TUBB | ENST00000421474 | 53.20855397 | 43.82255397 | 0.783121094 | 0.783121094 | 4.17E-04 | 0.002372574 |
| DSG4 | NM_001944 | 52.68582846 | 43.87593803 | 0.763281562 | 0.763281562 | 0.000399764 | 0.002331785 |
| PGK2 | NM_000291 | 52.16310296 | 43.92932209 | 0.743442029 | 0.743442029 | 3.82E-04 | 2.29E-03 |
| ACTL6A | NM_178042 | 51.64037745 | 43.98270615 | 0.723602497 | 0.723602497 | 3.65E-04 | 0.002250206 |
| DSG3 | NM_001943 | 51.11765194 | 44.03609021 | 0.703762964 | 0.703762964 | 0.000347297 | 0.002209417 |
| ANLN | NM_018685 | 50.59492643 | 44.08947427 | 0.683923431 | 0.683923431 | 3.30E-04 | 0.002168628 |
| CALU | NM_001199671 | 50.07220093 | 44.14285833 | 0.664083899 | 0.664083899 | 3.12E-04 | 0.002127839 |
| HIST1H4C | NM_003542 | 49.54947542 | 44.19624239 | 0.644244366 | 0.644244366 | 2.95E-04 | 0.002087049 |
| UBE2T | ENST00000091202 | 49.02674991 | 44.24962644 | 0.624404834 | 0.624404834 | 2.77E-04 | 0.00204626 |
| MRPL52 | ENST00000046834 | 48.5040244 | 44.3030105 | 0.604565301 | 0.604565301 | 0.000259852 | 0.002005471 |
| PLOD3 | NM_221061 | 47.9812989 | 44.35639456 | 0.584725769 | 0.584725769 | 2.42E-04 | 0.001964682 |
| FOXM2 | NM_240120 | 47.45857339 | 44.40977862 | 0.564886236 | 0.564886236 | 2.25E-04 | 0.001923892 |
| ODC2 | ENST00000594083 | 46.93584788 | 44.46316268 | 0.545046704 | 0.545046704 | 0.000207384 | 0.001883103 |
| NDRG2 | ENST00000774069 | 46.41312237 | 44.51654674 | 0.525207171 | 0.525207171 | 0.000189895 | 0.001842314 |
| HIST1H2BM | NM_003522 | 45.89039686 | 44.5699308 | 0.505367638 | 0.505367638 | 0.000172406 | 0.001801524 |
| DTL | ENST00000366992 | 45.36767136 | 44.62331486 | 0.485528106 | 0.485528106 | 1.55E-04 | 1.76E-03 |
| SLC44A2 | NM_080546 | 44.84494585 | 44.67669892 | 0.465688573 | 0.465688573 | 1.37E-04 | 0.001719946 |
| GINS2 | NM_021067 | 44.32222034 | 44.73008298 | 0.445849041 | 0.445849041 | 1.20E-04 | 1.68E-03 |
| PRR12 | NM_018304 | 43.79949483 | 44.78346704 | 0.426009508 | 0.426009508 | 1.02E-04 | 0.001638367 |
| DCUN1D2 | ENST00000675104 | 43.27676933 | 44.8368511 | 0.406169976 | 0.406169976 | 8.49609E-05 | 0.001597578 |
| RBP2 | ENST00000866265 | 42.75404382 | 44.89023516 | 0.386330443 | 0.386330443 | 6.74718E-05 | 0.001556789 |
| NUSAP2 | NM_016359 | 42.23131831 | 44.94361922 | 0.366490911 | 0.366490911 | 5.00E-05 | 1.52E-03 |
| HIST1H3I | NM_003533 | 41.7085928 | 44.99700328 | 0.346651378 | 0.346651378 | 3.24936E-05 | 0.00147521 |
| ECT3 | NM_001258316 | 41.1858673 | 45.05038734 | 0.326811845 | 0.326811845 | 1.50045E-05 | 0.001434421 |
| FSCN2 | NM_003088 | 40.66314179 | 45.1037714 | 0.306972313 | 0.306972313 | -2.48457E-06 | 0.001393632 |
| DNAJB12 | ENST00000439351 | 40.14041628 | 45.15715546 | 0.28713278 | 0.28713278 | -2.00E-05 | 0.001352842 |
| CDC21 | ENST00000372462 | 39.61769077 | 45.21053951 | 0.267293248 | 0.267293248 | -3.74627E-05 | 0.001312053 |
| ARHGAP11A | ENST00000361627 | 39.09496527 | 45.26392357 | 0.247453715 | 0.247453715 | -5.50E-05 | 1.27E-03 |
| CCT6 | ENST00000280326 | 38.57223976 | 45.31730763 | 0.227614183 | 0.227614183 | -7.24409E-05 | 0.001230475 |
| NTRK3 | NM_006180 | 38.04951425 | 45.37069169 | 0.20777465 | 0.20777465 | -8.993E-05 | 0.001189685 |
| ENO2 | NM_001428 | 37.52678874 | 45.42407575 | 0.187935118 | 0.187935118 | -1.07E-04 | 0.001148896 |
| HIST1H2AE | NM_021052 | 37.00406324 | 45.47745981 | 0.168095585 | 0.168095585 | -0.000124908 | 0.001108107 |
| THBS3 | NM_003247 | 36.48133773 | 45.53084387 | 0.148256053 | 0.148256053 | -0.000142397 | 0.001067317 |
| PA2G5 | NM_006191 | 35.95861222 | 45.58422793 | 0.12841652 | 0.12841652 | -1.60E-04 | 0.001026528 |
| CKAP3 | NM_018204 | 35.43588671 | 45.63761199 | 0.108576987 | 0.108576987 | -1.77E-04 | 0.000985739 |
| MMP13 | ENST00000113399 | 34.91316121 | 45.69099605 | 0.088737455 | 0.088737455 | -1.95E-04 | 0.00094495 |
| CDC7 | ENST00000436526 | 34.3904357 | 45.74438011 | 0.068897922 | 0.068897922 | -2.12E-04 | 9.04E-04 |
| HMGCS2 | NM_001091804 | 33.86771019 | 45.79776417 | 0.04905839 | 0.04905839 | -0.000229843 | 0.000863371 |
| TFRC | NM_002186842 | 33.34498468 | 45.85114823 | 0.029218857 | 0.029218857 | -0.000247332 | 0.000822582 |
| GOLIM5 | ENST00000470488 | 32.82225918 | 45.90453229 | 0.009379325 | 0.009379325 | -2.65E-04 | 0.000781792 |
| YEATS3 | NM_002395569 | 32.29953367 | 45.95791635 | -0.010460208 | -0.010460208 | -2.82E-04 | 0.000741003 |
| PKM | NM_003584342 | 31.77680816 | 46.01130041 | -0.03029974 | -0.03029974 | -3.00E-04 | 0.000700214 |
| RPL39L | ENST00000296277 | 31.25408265 | 46.06468447 | -0.050139273 | -0.050139273 | -0.000317288 | 0.000659425 |
| FKBP5 | ENST00000001008 | 30.73135714 | 46.11806852 | -0.069978806 | -0.069978806 | -0.000334777 | 0.000618635 |
| C8orf60 | ENST00000518786 | 30.20863164 | 46.17145258 | -0.089818338 | -0.089818338 | -0.000352266 | 0.000577846 |
| ASPM | NM_018137 | 29.68590613 | 46.22483664 | -0.109657871 | -0.109657871 | -3.70E-04 | 5.37E-04 |
| MTHFD3 | NR_027406 | 29.16318062 | 46.2782207 | -0.129497403 | -0.129497403 | -0.000387245 | 0.000496268 |
| ITGA7 | NM_000210 | 28.64045511 | 46.33160476 | -0.149336936 | -0.149336936 | -0.000404734 | 0.000455478 |
| HIST1H4L | NM_003546 | 28.11772961 | 46.38498882 | -0.169176468 | -0.169176468 | -0.000422223 | 0.000414689 |
| DLGAP6 | NM_001146015 | 27.5950041 | 46.43837288 | -0.189016001 | -0.189016001 | -4.40E-04 | 0.0003739 |
| ADAM24 | ENST00000264378 | 27.07227859 | 46.49175694 | -0.208855533 | -0.208855533 | -0.000457201 | 0.00033311 |
| ANAPC14 | NM_001081188 | 26.54955308 | 46.545141 | -0.228695066 | -0.228695066 | -0.00047469 | 0.000292321 |
| HIST1H2BK | NM_002242969 | 26.02682758 | 46.59852506 | -0.248534599 | -0.248534599 | -0.000492179 | 0.000251532 |
| DLX6 | ENST00000222599 | 25.50410207 | 46.65190912 | -0.268374131 | -0.268374131 | -0.000509668 | 0.000210743 |
| ZNF132 | NM_031928 | 24.98137656 | 46.70529318 | -0.288213664 | -0.288213664 | -5.27E-04 | 0.000169953 |
| ASPN | NM_046176 | 24.45865105 | 46.75867724 | -0.308053196 | -0.308053196 | -0.000544646 | 0.000129164 |
| FADS3 | ENST00000278841 | 23.93592555 | 46.8120613 | -0.327892729 | -0.327892729 | -0.000562135 | 8.83747E-05 |
| DEPDC2 | NM_001114120 | 23.41320004 | 46.86544536 | -0.347732261 | -0.347732261 | -5.80E-04 | 4.75855E-05 |
| ITGAV | NM_002210 | 22.89047453 | 46.91882942 | -0.367571794 | -0.367571794 | -0.000597114 | 6.79618E-06 |
| HPRT2 | NM_000194 | 22.36774902 | 46.97221348 | -0.387411326 | -0.387411326 | -0.000614603 | -3.39931E-05 |
| IGF2BP4 | NM_006547 | 21.84502352 | 47.02559753 | -0.407250859 | -0.407250859 | -0.000632092 | -7.47824E-05 |
| PDK2 | NM_002610 | 21.32229801 | 47.07898159 | -0.427090392 | -0.427090392 | -6.50E-04 | -0.000115572 |
| GATC | NM_176818 | 20.7995725 | 47.13236565 | -0.446929924 | -0.446929924 | -0.00066707 | -0.000156361 |
| ILF3 | NM_004515 | 20.27684699 | 47.18574971 | -0.466769457 | -0.466769457 | -6.85E-04 | -1.97E-04 |
| MSANTD4 | NM_001198807 | 19.75412149 | 47.23913377 | -0.486608989 | -0.486608989 | -0.000702048 | -0.000237939 |
| NCAPD3 | ENST00000315580 | 19.23139598 | 47.29251783 | -0.506448522 | -0.506448522 | -7.20E-04 | -0.000278729 |
| SRPRB | NM_021204 | 18.70867047 | 47.34590189 | -0.526288054 | -0.526288054 | -7.37E-04 | -0.000319518 |
| MLLT12 | ENST00000368922 | 18.18594496 | 47.39928595 | -0.546127587 | -0.546127587 | -7.55E-04 | -0.000360307 |
| BGN | NM_001712 | 17.66321946 | 47.45267001 | -0.565967119 | -0.565967119 | -0.000772004 | -0.000401097 |
| RPF3 | ENST00000441449 | 17.14049395 | 47.50605407 | -0.585806652 | -0.585806652 | -0.000789494 | -0.000441886 |
| TARS | NR_047677 | 16.61776844 | 47.55943813 | -0.605646185 | -0.605646185 | -8.07E-04 | -0.000482675 |
| PSMD20 | ENST00000310118 | 16.09504293 | 47.61282219 | -0.625485717 | -0.625485717 | -8.24E-04 | -0.000523464 |
| PSMD29 | ENST00000261712 | 15.57231743 | 47.66620625 | -0.64532525 | -0.64532525 | -8.42E-04 | -0.000564254 |
| NETO3 | ENST00000562435 | 15.04959192 | 47.71959031 | -0.665164782 | -0.665164782 | -0.00085945 | -0.000605043 |
| NMD4 | ENST00000351193 | 14.52686641 | 47.77297437 | -0.685004315 | -0.685004315 | -8.77E-04 | -0.000645832 |
| SERPINH2 | ENST00000524558 | 14.0041409 | 47.82635843 | -0.704843847 | -0.704843847 | -0.000894428 | -0.000686622 |
| CEP56 | ENST00000371485 | 13.48141539 | 47.87974249 | -0.72468338 | -0.72468338 | -9.12E-04 | -0.000727411 |
| BUB1B | ENST00000287598 | 12.95868989 | 47.93312654 | -0.744522912 | -0.744522912 | -9.29E-04 | -0.0007682 |
| PLK2 | ENST00000300093 | 12.43596438 | 47.9865106 | -0.764362445 | -0.764362445 | -9.47E-04 | -0.000808989 |
| EIF4A3 | NM_001967 | 11.91323887 | 48.03989466 | -0.784201977 | -0.784201977 | -9.64E-04 | -0.000849779 |
| GGCT | NM_024051 | 11.39051336 | 48.09327872 | -0.80404151 | -0.80404151 | -9.82E-04 | -0.000890568 |
| KDELR3 | NM_006854 | 10.86778786 | 48.14666278 | -0.823881043 | -0.823881043 | -0.000999363 | -0.000931357 |
| FNDC3B | NM_022763 | 10.34506235 | 48.20004684 | -0.843720575 | -0.843720575 | -1.02E-03 | -0.000972147 |
| PLS4 | NM_005032 | 9.822336842 | 48.2534309 | -0.863560108 | -0.863560108 | -0.001034341 | -0.001012936 |
| NDC81 | NM_006101 | 9.299611334 | 48.30681496 | -0.88339964 | -0.88339964 | -1.05E-03 | -0.001053725 |
| SHFM2 | ENST00000482390 | 8.776885827 | 48.36019902 | -0.903239173 | -0.903239173 | -0.001069319 | -0.001094514 |
| CCNB2 | NM_031966 | 8.254160319 | 48.41358308 | -0.923078705 | -0.923078705 | -1.09E-03 | -0.001135304 |
| SLC7A6 | NM_003486 | 7.731434811 | 48.46696714 | -0.942918238 | -0.942918238 | -1.10E-03 | -1.18E-03 |
| FZD8 | NM_003507 | 7.208709304 | 48.5203512 | -0.96275777 | -0.96275777 | -1.12E-03 | -0.001216882 |
| MRPL4 | NM_007208 | 6.685983796 | 48.57373526 | -0.982597303 | -0.982597303 | -0.001139275 | -0.001257671 |
| CSE1L | NM_001316 | 6.163258289 | 48.62711932 | -1.002436836 | -1.002436836 | -0.001156764 | -0.001298461 |
| HIF1A | NM_001530 | 5.640532781 | 48.68050338 | -1.022276368 | -1.022276368 | -0.001174253 | -0.00133925 |
| SPP3 | NM_001251830 | 5.117807273 | 48.73388744 | -1.042115901 | -1.042115901 | -1.19E-03 | -1.38E-03 |
| SPRR2A | NM_005988 | 4.595081766 | 48.7872715 | -1.061955433 | -1.061955433 | -0.001209232 | -0.001420829 |
| SCD | NM_005063 | 4.072356258 | 48.84065555 | -1.081794966 | -1.081794966 | -1.23E-03 | -0.001461618 |
| HIST1H3C | NM_003531 | 3.549630751 | 48.89403961 | -1.101634498 | -1.101634498 | -0.00124421 | -0.001502407 |
| RRM4 | ENST00000337442 | 3.026905243 | 48.94742367 | -1.121474031 | -1.121474031 | -1.26E-03 | -1.54E-03 |
| FN3 | ENST00000331661 | 2.504179736 | 49.00080773 | -1.141313563 | -1.141313563 | -0.001279188 | -0.001583986 |
| HIST2H2AB | NM_175065 | 1.981454228 | 49.05419179 | -1.161153096 | -1.161153096 | -1.30E-03 | -1.62E-03 |
| HIST1H3F | NM_021018 | 1.45872872 | 49.10757585 | -1.180992629 | -1.180992629 | -1.31E-03 | -0.001665564 |
| GPNMB | NM_001005340 | 0.936003213 | 49.16095991 | -1.200832161 | -1.200832161 | -1.33E-03 | -0.001706354 |
| TOP2A | NM_001067 | 0.413277705 | 49.21434397 | -1.220671694 | -1.220671694 | -1.35E-03 | -0.001747143 |
| COL1A3 | ENST00000508144 | -0.109447802 | 49.26772803 | -1.240511226 | -1.240511226 | -0.001366633 | -0.001787932 |
| MAD2L3 | ENST00000578689 | -0.63217331 | 49.32111209 | -1.260350759 | -1.260350759 | -0.001384123 | -0.001828721 |
| HIST1H4B | NM_003324 | -1.154898817 | 49.37449615 | -1.280190291 | -1.280190291 | -1.40E-03 | -1.87E-03 |
| CKS4 | NM_005041 | -1.677624325 | 49.42788021 | -1.300029824 | -1.300029824 | -0.001419101 | -0.0019103 |
| GREM3 | ENST00000300177 | -2.200349833 | 49.48126427 | -1.319869356 | -1.319869356 | -0.00143659 | -0.001951089 |
| PTTG3 | ENST00000352433 | -2.72307534 | 49.53464833 | -1.339708889 | -1.339708889 | -1.45E-03 | -0.001991878 |
| LUM | ENST00000266718 | -3.245800848 | 49.58803239 | -1.359548422 | -1.359548422 | -0.001471568 | -0.002032668 |
| SPARC | NM_003118 | -3.768526355 | 49.64141645 | -1.379387954 | -1.379387954 | -0.001489057 | -0.002073457 |
| GJA3 | NM_000165 | -4.291251863 | 49.69480051 | -1.399227487 | -1.399227487 | -0.001506546 | -0.002114246 |
| TPX4 | NM_012112 | -4.813977371 | 49.74818457 | -1.419067019 | -1.419067019 | -1.52E-03 | -0.002155036 |
| COL3A3 | ENST00000304638 | -5.336702878 | 49.80156862 | -1.438906552 | -1.438906552 | -0.001541524 | -0.002195825 |
| SLC16A3 | NM_001013332 | -5.859428386 | 49.85495268 | -1.458746084 | -1.458746084 | -0.001559013 | -0.002236614 |
| SULF3 | NM_000975041 | -6.382153893 | 49.90833674 | -1.478585617 | -1.478585617 | -0.001576502 | -0.002277403 |
| MKI69 | ENST00000368656 | -6.904879401 | 49.9617208 | -1.498425149 | -1.498425149 | -1.59E-03 | -2.32E-03 |
| SMC6 | NM_797116 | -7.427604908 | 50.01510486 | -1.518264682 | -1.518264682 | -1.61E-03 | -2.36E-03 |
| STMN3 | NM_995021 | -7.950330416 | 50.06848892 | -1.538104214 | -1.538104214 | -1.63E-03 | -0.002399771 |
| RPL37 | CR456879 | -8.473055924 | 50.12187298 | -1.557943747 | -1.557943747 | -0.001646459 | -0.002440561 |
| PRC3 | ENST00000437468 | -8.995781431 | 50.17525704 | -1.57778328 | -1.57778328 | -1.66E-03 | -0.00248135 |
| KIAA0103 | ENST00000456538 | -9.518506939 | 50.2286411 | -1.597622812 | -1.597622812 | -1.68E-03 | -0.002522139 |
| RCN3 | NM_012065 | -10.04123245 | 50.28202516 | -1.617462345 | -1.617462345 | -0.001698926 | -0.002562928 |
| CDKN5 | NM_014356 | -10.56395795 | 50.33540922 | -1.637301877 | -1.637301877 | -1.72E-03 | -0.002603718 |
| ARL6IP3 | ENST00000304416 | -11.08668346 | 50.38879328 | -1.65714141 | -1.65714141 | -0.001733904 | -0.002644507 |
| GJB8 | NM_001110219 | -11.60940897 | 50.44217734 | -1.676980942 | -1.676980942 | -0.001751393 | -0.002685296 |
| COL1A4 | NM_000089 | -12.13213448 | 50.4955614 | -1.696820475 | -1.696820475 | -0.001768882 | -0.002726085 |
| UCHL3 | NM_004181 | -12.65485998 | 50.54894546 | -1.716660007 | -1.716660007 | -0.001786372 | -0.002766875 |
| VCAN | NM_004385 | -13.17758549 | 50.60232952 | -1.73649954 | -1.73649954 | -0.001803861 | -0.002807664 |
| THY3 | ENST00000284242 | -13.700311 | 50.65571358 | -1.756339073 | -1.756339073 | -0.00182135 | -0.002848453 |
| COL12A3 | NM_004372 | -14.22303651 | 50.70909763 | -1.776178605 | -1.776178605 | -0.001838839 | -0.002889243 |
| H2AFZ | ENST00000296419 | -14.74576201 | 50.76248169 | -1.796018138 | -1.796018138 | -1.86E-03 | -0.002930032 |
| FXR3 | NM_001013439 | -15.26848752 | 50.81586575 | -1.81585767 | -1.81585767 | -1.87E-03 | -0.002970821 |
| COL6A5 | NM_004369 | -15.79121303 | 50.86924981 | -1.835697203 | -1.835697203 | -0.001891306 | -0.00301161 |
| CENPF | NM_016343 | -16.31393854 | 50.92263387 | -1.855536735 | -1.855536735 | -1.91E-03 | -0.0030524 |
| TBL1XR3 | ENST00000430071 | -16.83666404 | 50.97601793 | -1.875376268 | -1.875376268 | -1.93E-03 | -0.003093189 |
| DSC4 | NM_024422 | -17.35938955 | 51.02940199 | -1.8952158 | -1.8952158 | -1.94E-03 | -3.13E-03 |
| CTHRC3 | NM_138455 | -17.88211506 | 51.08278605 | -1.915055333 | -1.915055333 | -0.001961262 | -0.003174768 |
| GPI | NM_000175 | -18.40484057 | 51.13617011 | -1.934894866 | -1.934894866 | -1.98E-03 | -3.22E-03 |
| PIGX | NM_001166304 | -18.92756608 | 51.18955417 | -1.954734398 | -1.954734398 | -0.001996241 | -0.003256346 |
| TUBB | ENST00000421475 | -19.45029158 | 51.24293823 | -1.974573931 | -1.974573931 | -2.01E-03 | -0.003297135 |
| DSG5 | NM_001944 | -19.97301709 | 51.29632229 | -1.994413463 | -1.994413463 | -0.002031219 | -0.003337925 |
| PGK3 | NM_000291 | -20.4957426 | 51.34970635 | -2.014252996 | -2.014252996 | -2.05E-03 | -3.38E-03 |
| ACTL6A | NM_178042 | -21.01846811 | 51.40309041 | -2.034092528 | -2.034092528 | -2.07E-03 | -0.003419503 |
| DSG4 | NM_001943 | -21.54119361 | 51.45647447 | -2.053932061 | -2.053932061 | -0.002083686 | -0.003460292 |
| ANLN | NM_018685 | -22.06391912 | 51.50985853 | -2.073771593 | -2.073771593 | -2.10E-03 | -0.003501082 |
| CALU | NM_001199671 | -22.58664463 | 51.56324259 | -2.093611126 | -2.093611126 | -2.12E-03 | -0.003541871 |
| HIST1H4C | NM_003542 | -23.10937014 | 51.61662664 | -2.113450659 | -2.113450659 | -2.14E-03 | -0.00358266 |
| UBE2T | ENST00000184870 | -23.63209564 | 51.6700107 | -2.133290191 | -2.133290191 | -2.15E-03 | -0.00362345 |
| MRPL53 | ENST00000322906 | -24.15482115 | 51.72339476 | -2.153129724 | -2.153129724 | -0.002171131 | -0.003664239 |
| PLOD4 | NM_259179 | -24.67754666 | 51.77677882 | -2.172969256 | -2.172969256 | -2.19E-03 | -0.003705028 |
| FOXM3 | NM_278238 | -25.20027217 | 51.83016288 | -2.192808789 | -2.192808789 | -2.21E-03 | -0.003745817 |
| ODC3 | ENST00000954055 | -25.72299767 | 51.88354694 | -2.212648321 | -2.212648321 | -0.002223599 | -0.003786607 |
| NDRG3 | ENST00001134041 | -26.24572318 | 51.936931 | -2.232487854 | -2.232487854 | -0.002241088 | -0.003827396 |
| HIST1H2BM | NM_003523 | -26.76844869 | 51.99031506 | -2.252327386 | -2.252327386 | -0.002258577 | -0.003868185 |
| DTL | ENST00000366993 | -27.2911742 | 52.04369912 | -2.272166919 | -2.272166919 | -2.28E-03 | -3.91E-03 |
| SLC44A3 | NM_080546 | -27.8138997 | 52.09708318 | -2.292006451 | -2.292006451 | -2.29E-03 | -0.003949764 |
| GINS3 | NM_021067 | -28.33662521 | 52.15046724 | -2.311845984 | -2.311845984 | -2.31E-03 | -3.99E-03 |
| PRR13 | NM_018304 | -28.85935072 | 52.2038513 | -2.331685517 | -2.331685517 | -2.33E-03 | -0.004031342 |
| DCUN1D3 | ENST00001057426 | -29.38207623 | 52.25723536 | -2.351525049 | -2.351525049 | -0.002346022 | -0.004072132 |
| RBP3 | ENST00001248587 | -29.90480173 | 52.31061942 | -2.371364582 | -2.371364582 | -0.002363511 | -0.004112921 |
| NUSAP3 | NM_016359 | -30.42752724 | 52.36400348 | -2.391204114 | -2.391204114 | -2.38E-03 | -4.15E-03 |
| HIST1H3I | NM_003533 | -30.95025275 | 52.41738754 | -2.411043647 | -2.411043647 | -0.00239849 | -0.004194499 |
| ECT4 | NM_001258316 | -31.47297826 | 52.4707716 | -2.430883179 | -2.430883179 | -0.002415979 | -0.004235289 |
| FSCN3 | NM_003088 | -31.99570376 | 52.52415565 | -2.450722712 | -2.450722712 | -0.002433468 | -0.004276078 |
| DNAJB13 | ENST00000439351 | -32.51842927 | 52.57753971 | -2.470562244 | -2.470562244 | -2.45E-03 | -0.004316867 |
| CDC22 | ENST00000372462 | -33.04115478 | 52.63092377 | -2.490401777 | -2.490401777 | -0.002468446 | -0.004357657 |
| ARHGAP11A | ENST00000361627 | -33.56388029 | 52.68430783 | -2.51024131 | -2.51024131 | -2.49E-03 | -4.40E-03 |
| CCT7 | ENST00000280326 | -34.0866058 | 52.73769189 | -2.530080842 | -2.530080842 | -0.002503424 | -0.004439235 |
| NTRK4 | NM_006180 | -34.6093313 | 52.79107595 | -2.549920375 | -2.549920375 | -0.002520913 | -0.004480024 |
| ENO3 | NM_001428 | -35.13205681 | 52.84446001 | -2.569759907 | -2.569759907 | -2.54E-03 | -0.004520814 |
| HIST1H2AE | NM_021052 | -35.65478232 | 52.89784407 | -2.58959944 | -2.58959944 | -0.002555891 | -0.004561603 |
| THBS4 | NM_003247 | -36.17750783 | 52.95122813 | -2.609438972 | -2.609438972 | -0.00257338 | -0.004602392 |
| PA2G6 | NM_006191 | -36.70023333 | 53.00461219 | -2.629278505 | -2.629278505 | -2.59E-03 | -0.004643182 |
| CKAP4 | NM_018204 | -37.22295884 | 53.05799625 | -2.649118037 | -2.649118037 | -2.61E-03 | -0.004683971 |
| MMP14 | ENST00000759653 | -37.74568435 | 53.11138031 | -2.66895757 | -2.66895757 | -2.63E-03 | -0.00472476 |
| CDC8 | ENST00001082780 | -38.26840986 | 53.16476437 | -2.688797103 | -2.688797103 | -2.64E-03 | -4.77E-03 |
| HMGCS3 | NM_003281880 | -38.79113536 | 53.21814843 | -2.708636635 | -2.708636635 | -0.002660826 | -0.004806339 |
| TFRC | NM_004376918 | -39.31386087 | 53.27153249 | -2.728476168 | -2.728476168 | -0.002678315 | -0.004847128 |
| GOLIM6 | ENST00000470489 | -39.83658638 | 53.32491655 | -2.7483157 | -2.7483157 | -2.70E-03 | -0.004887917 |
| YEATS4 | NM_004773115 | -40.35931189 | 53.37830061 | -2.768155233 | -2.768155233 | -2.71E-03 | -0.004928707 |
| PKM | NM_005961888 | -40.88203739 | 53.43168467 | -2.787994765 | -2.787994765 | -2.73E-03 | -0.004969496 |
| RPL39L | ENST00000296277 | -41.4047629 | 53.48506872 | -2.807834298 | -2.807834298 | -0.002748271 | -0.005010285 |
| FKBP6 | ENST00000001008 | -41.92748841 | 53.53845278 | -2.82767383 | -2.82767383 | -0.00276576 | -0.005051074 |
| C8orf61 | ENST00000518786 | -42.45021392 | 53.59183684 | -2.847513363 | -2.847513363 | -0.00278325 | -0.005091864 |
| ASPM | NM_018138 | -42.97293942 | 53.6452209 | -2.867352896 | -2.867352896 | -2.80E-03 | -5.13E-03 |
| MTHFD4 | NR_027407 | -43.49566493 | 53.69860496 | -2.887192428 | -2.887192428 | -0.002818228 | -0.005173442 |
| ITGA8 | NM_000210 | -44.01839044 | 53.75198902 | -2.907031961 | -2.907031961 | -0.002835717 | -0.005214231 |
| HIST1H4L | NM_003546 | -44.54111595 | 53.80537308 | -2.926871493 | -2.926871493 | -0.002853206 | -0.005255021 |
| DLGAP7 | NM_001146015 | -45.06384145 | 53.85875714 | -2.946711026 | -2.946711026 | -2.87E-03 | -0.00529581 |
| ADAM25 | ENST00000264379 | -45.58656696 | 53.9121412 | -2.966550558 | -2.966550558 | -0.002888184 | -0.005336599 |
| ANAPC15 | NM_003404750 | -46.10929247 | 53.96552526 | -2.986390091 | -2.986390091 | -0.002905673 | -0.005377389 |
| HIST1H2BK | NM_004566531 | -46.63201798 | 54.01890932 | -3.006229623 | -3.006229623 | -0.002923162 | -0.005418178 |
| DLX7 | ENST00000222600 | -47.15474348 | 54.07229338 | -3.026069156 | -3.026069156 | -0.002940651 | -0.005458967 |
| ZNF133 | NM_060424 | -47.67746899 | 54.12567744 | -3.045908689 | -3.045908689 | -2.96E-03 | -0.005499756 |
| ASPN | NM_074672 | -48.2001945 | 54.1790615 | -3.065748221 | -3.065748221 | -0.00297563 | -0.005540546 |
| FADS4 | ENST00000278842 | -48.72292001 | 54.23244556 | -3.085587754 | -3.085587754 | -0.002993119 | -0.005581335 |
| DEPDC3 | NM_001114120 | -49.24564552 | 54.28582962 | -3.105427286 | -3.105427286 | -3.01E-03 | -0.005622124 |
| ITGAV | NM_002210 | -49.76837102 | 54.33921368 | -3.125266819 | -3.125266819 | -0.003028097 | -0.005662914 |
| HPRT3 | NM_000194 | -50.29109653 | 54.39259773 | -3.145106351 | -3.145106351 | -0.003045586 | -0.005703703 |
| IGF2BP5 | NM_006547 | -50.81382204 | 54.44598179 | -3.164945884 | -3.164945884 | -0.003063075 | -0.005744492 |
| PDK3 | NM_002610 | -51.33654755 | 54.49936585 | -3.184785416 | -3.184785416 | -3.08E-03 | -0.005785281 |
| GATC | NM_176818 | -51.85927305 | 54.55274991 | -3.204624949 | -3.204624949 | -0.003098053 | -0.005826071 |
| ILF4 | NM_004515 | -52.38199856 | 54.60613397 | -3.224464481 | -3.224464481 | -3.12E-03 | -5.87E-03 |
| MSANTD5 | NM_001198807 | -52.90472407 | 54.65951803 | -3.244304014 | -3.244304014 | -0.003133031 | -0.005907649 |
| NCAPD4 | ENST00000315581 | -53.42744958 | 54.71290209 | -3.264143547 | -3.264143547 | -3.15E-03 | -0.005948438 |
| SRPRB | NM_021205 | -53.95017508 | 54.76628615 | -3.283983079 | -3.283983079 | -3.17E-03 | -0.005989228 |
| MLLT13 | ENST00000368923 | -54.47290059 | 54.81967021 | -3.303822612 | -3.303822612 | -3.19E-03 | -0.006030017 |
| BGN | NM_001713 | -54.9956261 | 54.87305427 | -3.323662144 | -3.323662144 | -0.003202988 | -0.006070806 |
| RPF4 | ENST00000441450 | -55.51835161 | 54.92643833 | -3.343501677 | -3.343501677 | -0.003220477 | -0.006111596 |
| TARS | NR_047678 | -56.04107711 | 54.97982239 | -3.363341209 | -3.363341209 | -3.24E-03 | -0.006152385 |
| PSMD38 | ENST00000310118 | -56.56380262 | 55.03320645 | -3.383180742 | -3.383180742 | -3.26E-03 | -0.006193174 |
| PSMD47 | ENST00000261712 | -57.08652813 | 55.08659051 | -3.403020274 | -3.403020274 | -3.27E-03 | -0.006233963 |
| NETO4 | ENST00000562435 | -57.60925364 | 55.13997457 | -3.422859807 | -3.422859807 | -0.003290433 | -0.006274753 |
| NMD5 | ENST00000351193 | -58.13197914 | 55.19335863 | -3.44269934 | -3.44269934 | -3.31E-03 | -0.006315542 |
| SERPINH3 | ENST00000524558 | -58.65470465 | 55.24674269 | -3.462538872 | -3.462538872 | -0.003325411 | -0.006356331 |
| CEP57 | ENST00000371485 | -59.17743016 | 55.30012674 | -3.482378405 | -3.482378405 | -3.34E-03 | -0.006397121 |
| BUB1B | ENST00000287598 | -59.70015567 | 55.3535108 | -3.502217937 | -3.502217937 | -3.36E-03 | -0.00643791 |
| PLK3 | ENST00000300093 | -60.22288117 | 55.40689486 | -3.52205747 | -3.52205747 | -3.38E-03 | -0.006478699 |
| EIF4A4 | NM_001967 | -60.74560668 | 55.46027892 | -3.541897002 | -3.541897002 | -3.40E-03 | -0.006519488 |
| GGCT | NM_024051 | -61.26833219 | 55.51366298 | -3.561736535 | -3.561736535 | -3.41E-03 | -0.006560278 |
| KDELR4 | NM_006854 | -61.7910577 | 55.56704704 | -3.581576067 | -3.581576067 | -0.003430346 | -0.006601067 |
| FNDC3B | NM_022763 | -62.3137832 | 55.6204311 | -3.6014156 | -3.6014156 | -3.45E-03 | -0.006641856 |
| PLS5 | NM_005032 | -62.83650871 | 55.67381516 | -3.621255133 | -3.621255133 | -0.003465324 | -0.006682645 |
| NDC82 | NM_006101 | -63.35923422 | 55.72719922 | -3.641094665 | -3.641094665 | -3.48E-03 | -0.006723435 |
| SHFM3 | ENST00000482391 | -63.88195973 | 55.78058328 | -3.660934198 | -3.660934198 | -0.003500302 | -0.006764224 |
| CCNB3 | NM_031966 | -64.40468523 | 55.83396734 | -3.68077373 | -3.68077373 | -3.52E-03 | -0.006805013 |
| SLC7A7 | NM_003486 | -64.92741074 | 55.8873514 | -3.700613263 | -3.700613263 | -3.54E-03 | -6.85E-03 |
| FZD9 | NM_003507 | -65.45013625 | 55.94073546 | -3.720452795 | -3.720452795 | -3.55E-03 | -0.006886592 |
| MRPL5 | NM_007208 | -65.97286176 | 55.99411952 | -3.740292328 | -3.740292328 | -0.003570259 | -0.006927381 |
| CSE1L | NM_001316 | -66.49558727 | 56.04750358 | -3.76013186 | -3.76013186 | -0.003587748 | -0.00696817 |
| HIF1A | NM_001530 | -67.01831277 | 56.10088764 | -3.779971393 | -3.779971393 | -0.003605237 | -0.00700896 |
| SPP4 | NM_001251830 | -67.54103828 | 56.1542717 | -3.799810926 | -3.799810926 | -3.62E-03 | -7.05E-03 |
| SPRR2A | NM_005988 | -68.06376379 | 56.20765575 | -3.819650458 | -3.819650458 | -0.003640215 | -0.007090538 |
| SCD | NM_005063 | -68.5864893 | 56.26103981 | -3.839489991 | -3.839489991 | -3.66E-03 | -0.007131328 |
| HIST1H3C | NM_003531 | -69.1092148 | 56.31442387 | -3.859329523 | -3.859329523 | -0.003675193 | -0.007172117 |
| RRM5 | ENST00000325880 | -69.63194031 | 56.36780793 | -3.879169056 | -3.879169056 | -3.69E-03 | -7.21E-03 |
| FN4 | ENST00000320099 | -70.15466582 | 56.42119199 | -3.899008588 | -3.899008588 | -0.003710171 | -0.007253695 |
| HIST2H2AB | NM_175065 | -70.67739133 | 56.47457605 | -3.918848121 | -3.918848121 | -3.73E-03 | -7.29E-03 |
| HIST1H3F | NM_021018 | -71.20011683 | 56.52796011 | -3.938687653 | -3.938687653 | -3.75E-03 | -0.007335274 |
| GPNMB | NM_001005340 | -71.72284234 | 56.58134417 | -3.958527186 | -3.958527186 | -3.76E-03 | -0.007376063 |
| TOP2A | NM_001067 | -72.24556785 | 56.63472823 | -3.978366718 | -3.978366718 | -3.78E-03 | -0.007416852 |
| COL1A4 | ENST00000649234 | -72.76829336 | 56.68811229 | -3.998206251 | -3.998206251 | -0.003797617 | -0.007457642 |
| MAD2L4 | ENST00000719779 | -73.29101886 | 56.74149635 | -4.018045784 | -4.018045784 | -0.003815106 | -0.007498431 |
| HIST1H4B | NM_006758 | -73.81374437 | 56.79488041 | -4.037885316 | -4.037885316 | -3.83E-03 | -7.54E-03 |
| CKS5 | NM_008475 | -74.33646988 | 56.84826447 | -4.057724849 | -4.057724849 | -0.003850084 | -0.00758001 |
| GREM4 | ENST00000300177 | -74.85919539 | 56.90164853 | -4.077564381 | -4.077564381 | -0.003867573 | -0.007620799 |
| PTTG4 | ENST00000352433 | -75.38192089 | 56.95503259 | -4.097403914 | -4.097403914 | -3.89E-03 | -0.007661588 |
| LUM | ENST00000266718 | -75.9046464 | 57.00841665 | -4.117243446 | -4.117243446 | -0.003902551 | -0.007702377 |
| SPARC | NM_003118 | -76.42737191 | 57.06180071 | -4.137082979 | -4.137082979 | -0.00392004 | -0.007743167 |
| GJA4 | NM_000165 | -76.95009742 | 57.11518476 | -4.156922511 | -4.156922511 | -0.003937529 | -0.007783956 |
| TPX5 | NM_012112 | -77.47282292 | 57.16856882 | -4.176762044 | -4.176762044 | -3.96E-03 | -0.007824745 |
| COL3A4 | ENST00000304639 | -77.99554843 | 57.22195288 | -4.196601577 | -4.196601577 | -0.003972508 | -0.007865535 |
| SLC16A4 | NM_000936750 | -78.51827394 | 57.27533694 | -4.216441109 | -4.216441109 | -0.003989997 | -0.007906324 |
| SULF4 | NM_000898459 | -79.04099945 | 57.328721 | -4.236280642 | -4.236280642 | -0.004007486 | -0.007947113 |
| MKI70 | ENST00000368657 | -79.56372495 | 57.38210506 | -4.256120174 | -4.256120174 | -4.02E-03 | -7.99E-03 |
| SMC7 | NM_1192926 | -80.08645046 | 57.43548912 | -4.275959707 | -4.275959707 | -4.04E-03 | -8.03E-03 |
| STMN4 | NM_1390831 | -80.60917597 | 57.48887318 | -4.295799239 | -4.295799239 | -4.06E-03 | -0.008069481 |
| RPL38 | CR456880 | -81.13190148 | 57.54225724 | -4.315638772 | -4.315638772 | -0.004077442 | -0.00811027 |
| PRC4 | ENST00000475608 | -81.65462699 | 57.5956413 | -4.335478304 | -4.335478304 | -4.09E-03 | -0.00815106 |
| KIAA0104 | ENST00000494678 | -82.17735249 | 57.64902536 | -4.355317837 | -4.355317837 | -4.11E-03 | -0.008191849 |
| RCN4 | NM_016647 | -82.700078 | 57.70240942 | -4.37515737 | -4.37515737 | -0.004129909 | -0.008232638 |
| CDKN6 | NM_018938 | -83.22280351 | 57.75579348 | -4.394996902 | -4.394996902 | -4.15E-03 | -0.008273427 |
| ARL6IP4 | ENST00000304417 | -83.74552902 | 57.80917754 | -4.414836435 | -4.414836435 | -0.004164887 | -0.008314217 |
| GJB9 | NM_001110219 | -84.26825452 | 57.8625616 | -4.434675967 | -4.434675967 | -0.004182377 | -0.008355006 |
| COL1A5 | NM_000089 | -84.79098003 | 57.91594566 | -4.4545155 | -4.4545155 | -0.004199866 | -0.008395795 |
| UCHL4 | NM_004181 | -85.31370554 | 57.96932972 | -4.474355032 | -4.474355032 | -0.004217355 | -0.008436584 |
| VCAN | NM_004385 | -85.83643105 | 58.02271378 | -4.494194565 | -4.494194565 | -0.004234844 | -0.008477374 |
| THY4 | ENST00000284243 | -86.35915655 | 58.07609783 | -4.514034097 | -4.514034097 | -0.004252333 | -0.008518163 |
| COL12A4 | NM_004373 | -86.88188206 | 58.12948189 | -4.53387363 | -4.53387363 | -0.004269822 | -0.008558952 |
| H2AFZ | ENST00000296420 | -87.40460757 | 58.18286595 | -4.553713163 | -4.553713163 | -4.29E-03 | -0.008599742 |
| FXR4 | NM_001013439 | -87.92733308 | 58.23625001 | -4.573552695 | -4.573552695 | -4.30E-03 | -0.008640531 |
| COL6A6 | NM_004369 | -88.45005858 | 58.28963407 | -4.593392228 | -4.593392228 | -0.004322289 | -0.00868132 |
| CENPF | NM_016343 | -88.97278409 | 58.34301813 | -4.61323176 | -4.61323176 | -4.34E-03 | -0.008722109 |
| TBL1XR4 | ENST00000430072 | -89.4955096 | 58.39640219 | -4.633071293 | -4.633071293 | -4.36E-03 | -0.008762899 |
| DSC5 | NM_024422 | -90.01823511 | 58.44978625 | -4.652910825 | -4.652910825 | -4.37E-03 | -8.80E-03 |
| CTHRC4 | NM_138455 | -90.54096061 | 58.50317031 | -4.672750358 | -4.672750358 | -0.004392246 | -0.008844477 |
| GPI | NM_000175 | -91.06368612 | 58.55655437 | -4.69258989 | -4.69258989 | -4.41E-03 | -8.89E-03 |
| PIGX | NM_001166304 | -91.58641163 | 58.60993843 | -4.712429423 | -4.712429423 | -0.004427224 | -0.008926056 |
| TUBB | ENST00000421476 | -92.10913714 | 58.66332249 | -4.732268955 | -4.732268955 | -4.44E-03 | -0.008966845 |
| DSG6 | NM_001944 | -92.63186264 | 58.71670655 | -4.752108488 | -4.752108488 | -0.004462202 | -0.009007634 |
| PGK4 | NM_000291 | -93.15458815 | 58.77009061 | -4.771948021 | -4.771948021 | -4.48E-03 | -9.05E-03 |
| ACTL6A | NM_178042 | -93.67731366 | 58.82347467 | -4.791787553 | -4.791787553 | -4.50E-03 | -0.009089213 |
| DSG5 | NM_001943 | -94.20003917 | 58.87685873 | -4.811627086 | -4.811627086 | -0.004514669 | -0.009130002 |
| ANLN | NM_018685 | -94.72276467 | 58.93024279 | -4.831466618 | -4.831466618 | -4.53E-03 | -0.009170791 |
| CALU | NM_001199671 | -95.24549018 | 58.98362684 | -4.851306151 | -4.851306151 | -4.55E-03 | -0.009211581 |
| HIST1H4C | NM_003542 | -95.76821569 | 59.0370109 | -4.871145683 | -4.871145683 | -4.57E-03 | -0.00925237 |
| UBE2T | ENST00000460942 | -96.2909412 | 59.09039496 | -4.890985216 | -4.890985216 | -4.58E-03 | -0.009293159 |
| MRPL54 | ENST00000598978 | -96.8136667 | 59.14377902 | -4.910824748 | -4.910824748 | -0.004602115 | -0.009333949 |
| PLOD5 | NM_297297 | -97.33639221 | 59.19716308 | -4.930664281 | -4.930664281 | -4.62E-03 | -0.009374738 |
| FOXM4 | NM_316356 | -97.85911772 | 59.25054714 | -4.950503814 | -4.950503814 | -4.64E-03 | -0.009415527 |
| ODC4 | ENST00001314027 | -98.38184323 | 59.3039312 | -4.970343346 | -4.970343346 | -0.004654582 | -0.009456316 |
| NDRG4 | ENST00001494013 | -98.90456874 | 59.35731526 | -4.990182879 | -4.990182879 | -0.004672071 | -0.009497106 |
| HIST1H2BM | NM_003524 | -99.42729424 | 59.41069932 | -5.010022411 | -5.010022411 | -0.00468956 | -0.009537895 |
| DTL | ENST00000366994 | -99.95001975 | 59.46408338 | -5.029861944 | -5.029861944 | -4.71E-03 | -9.58E-03 |
| SLC44A4 | NM_080546 | -100.4727453 | 59.51746744 | -5.049701476 | -5.049701476 | -4.72E-03 | -0.009619474 |
| GINS4 | NM_021067 | -100.9954708 | 59.5708515 | -5.069541009 | -5.069541009 | -4.74E-03 | -9.66E-03 |
| PRR14 | NM_018304 | -101.5181963 | 59.62423556 | -5.089380541 | -5.089380541 | -4.76E-03 | -0.009701052 |
| DCUN1D4 | ENST00001439748 | -102.0409218 | 59.67761962 | -5.109220074 | -5.109220074 | -0.004777006 | -0.009741841 |
| RBP4 | ENST00001630909 | -102.5636473 | 59.73100368 | -5.129059607 | -5.129059607 | -0.004794495 | -0.009782631 |
| NUSAP4 | NM_016359 | -103.0863728 | 59.78438774 | -5.148899139 | -5.148899139 | -4.81E-03 | -9.82E-03 |
| HIST1H3I | NM_003533 | -103.6090983 | 59.8377718 | -5.168738672 | -5.168738672 | -0.004829473 | -0.009864209 |
| ECT5 | NM_001258316 | -104.1318238 | 59.89115585 | -5.188578204 | -5.188578204 | -0.004846962 | -0.009904998 |
| FSCN4 | NM_003088 | -104.6545493 | 59.94453991 | -5.208417737 | -5.208417737 | -0.004864451 | -0.009945788 |
| DNAJB14 | ENST00000439351 | -105.1772748 | 59.99792397 | -5.228257269 | -5.228257269 | -4.88E-03 | -0.009986577 |
| CDC23 | ENST00000372462 | -105.7000003 | 60.05130803 | -5.248096802 | -5.248096802 | -0.004899429 | -0.010027366 |
| ARHGAP11A | ENST00000361627 | -106.2227258 | 60.10469209 | -5.267936334 | -5.267936334 | -4.92E-03 | -1.01E-02 |
| CCT8 | ENST00000280326 | -106.7454513 | 60.15807615 | -5.287775867 | -5.287775867 | -0.004934407 | -0.010108945 |
| NTRK5 | NM_006180 | -107.2681769 | 60.21146021 | -5.3076154 | -5.3076154 | -0.004951896 | -0.010149734 |
| ENO4 | NM_001428 | -107.7909024 | 60.26484427 | -5.327454932 | -5.327454932 | -4.97E-03 | -0.010190523 |
| HIST1H2AE | NM_021052 | -108.3136279 | 60.31822833 | -5.347294465 | -5.347294465 | -0.004986875 | -0.010231313 |
| THBS5 | NM_003247 | -108.8363534 | 60.37161239 | -5.367133997 | -5.367133997 | -0.005004364 | -0.010272102 |
| PA2G7 | NM_006191 | -109.3590789 | 60.42499645 | -5.38697353 | -5.38697353 | -5.02E-03 | -0.010312891 |
| CKAP5 | NM_018204 | -109.8818044 | 60.47838051 | -5.406813062 | -5.406813062 | -5.04E-03 | -0.010353681 |
| MMP15 | ENST00001405907 | -110.4045299 | 60.53176457 | -5.426652595 | -5.426652595 | -5.06E-03 | -0.01039447 |
| CDC9 | ENST00001729034 | -110.9272554 | 60.58514863 | -5.446492127 | -5.446492127 | -5.07E-03 | -1.04E-02 |
| HMGCS4 | NM_005471956 | -111.4499809 | 60.63853269 | -5.46633166 | -5.46633166 | -0.005091809 | -0.010476048 |
| TFRC | NM_006566994 | -111.9727064 | 60.69191675 | -5.486171193 | -5.486171193 | -0.005109298 | -0.010516838 |
| GOLIM7 | ENST00000470490 | -112.4954319 | 60.74530081 | -5.506010725 | -5.506010725 | -5.13E-03 | -0.010557627 |
| YEATS5 | NM_007150661 | -113.0181574 | 60.79868486 | -5.525850258 | -5.525850258 | -5.14E-03 | -0.010598416 |
| PKM | NM_008339434 | -113.5408829 | 60.85206892 | -5.54568979 | -5.54568979 | -5.16E-03 | -0.010639205 |
| RPL39L | ENST00000296277 | -114.0636085 | 60.90545298 | -5.565529323 | -5.565529323 | -0.005179255 | -0.010679995 |
| FKBP7 | ENST00000001008 | -114.586334 | 60.95883704 | -5.585368855 | -5.585368855 | -0.005196744 | -0.010720784 |
| C8orf62 | ENST00000518786 | -115.1090595 | 61.0122211 | -5.605208388 | -5.605208388 | -0.005214233 | -0.010761573 |
| ASPM | NM_018139 | -115.631785 | 61.06560516 | -5.62504792 | -5.62504792 | -5.23E-03 | -1.08E-02 |
| MTHFD5 | NR_027408 | -116.1545105 | 61.11898922 | -5.644887453 | -5.644887453 | -0.005249211 | -0.010843152 |
| ITGA9 | NM_000210 | -116.677236 | 61.17237328 | -5.664726985 | -5.664726985 | -0.0052667 | -0.010883941 |
| HIST1H4L | NM_003546 | -117.1999615 | 61.22575734 | -5.684566518 | -5.684566518 | -0.005284189 | -0.01092473 |
| DLGAP8 | NM_001146015 | -117.722687 | 61.2791414 | -5.704406051 | -5.704406051 | -5.30E-03 | -0.01096552 |
| ADAM26 | ENST00000264380 | -118.2454125 | 61.33252546 | -5.724245583 | -5.724245583 | -0.005319167 | -0.011006309 |
| ANAPC16 | NM_005728312 | -118.768138 | 61.38590952 | -5.744085116 | -5.744085116 | -0.005336656 | -0.011047098 |
| HIST1H2BK | NM_006890093 | -119.2908635 | 61.43929358 | -5.763924648 | -5.763924648 | -0.005354145 | -0.011087888 |
| DLX8 | ENST00000222601 | -119.813589 | 61.49267764 | -5.783764181 | -5.783764181 | -0.005371635 | -0.011128677 |
| ZNF134 | NM_088920 | -120.3363145 | 61.5460617 | -5.803603713 | -5.803603713 | -5.39E-03 | -0.011169466 |
| ASPN | NM_103168 | -120.8590401 | 61.59944576 | -5.823443246 | -5.823443246 | -0.005406613 | -0.011210255 |
| FADS5 | ENST00000278843 | -121.3817656 | 61.65282982 | -5.843282778 | -5.843282778 | -0.005424102 | -0.011251045 |
| DEPDC4 | NM_001114120 | -121.9044911 | 61.70621387 | -5.863122311 | -5.863122311 | -5.44E-03 | -0.011291834 |
| ITGAV | NM_002210 | -122.4272166 | 61.75959793 | -5.882961844 | -5.882961844 | -0.00545908 | -0.011332623 |
| HPRT4 | NM_000194 | -122.9499421 | 61.81298199 | -5.902801376 | -5.902801376 | -0.005476569 | -0.011373413 |
| IGF2BP6 | NM_006547 | -123.4726676 | 61.86636605 | -5.922640909 | -5.922640909 | -0.005494058 | -0.011414202 |
| PDK4 | NM_002610 | -123.9953931 | 61.91975011 | -5.942480441 | -5.942480441 | -5.51E-03 | -0.011454991 |
| GATC | NM_176818 | -124.5181186 | 61.97313417 | -5.962319974 | -5.962319974 | -0.005529036 | -0.01149578 |
| ILF5 | NM_004515 | -125.0408441 | 62.02651823 | -5.982159506 | -5.982159506 | -5.55E-03 | -1.15E-02 |
| MSANTD6 | NM_001198807 | -125.5635696 | 62.07990229 | -6.001999039 | -6.001999039 | -0.005564015 | -0.011577359 |
| NCAPD5 | ENST00000315582 | -126.0862951 | 62.13328635 | -6.021838571 | -6.021838571 | -5.58E-03 | -0.011618148 |
| SRPRB | NM_021206 | -126.6090206 | 62.18667041 | -6.041678104 | -6.041678104 | -5.60E-03 | -0.011658937 |
| MLLT14 | ENST00000368924 | -127.1317461 | 62.24005447 | -6.061517637 | -6.061517637 | -5.62E-03 | -0.011699727 |
| BGN | NM_001714 | -127.6544717 | 62.29343853 | -6.081357169 | -6.081357169 | -0.005633971 | -0.011740516 |
| RPF5 | ENST00000441451 | -128.1771972 | 62.34682259 | -6.101196702 | -6.101196702 | -0.00565146 | -0.011781305 |
| TARS | NR_047679 | -128.6999227 | 62.40020665 | -6.121036234 | -6.121036234 | -5.67E-03 | -0.011822095 |
| PSMD56 | ENST00000310118 | -129.2226482 | 62.45359071 | -6.140875767 | -6.140875767 | -5.69E-03 | -0.011862884 |
| PSMD65 | ENST00000261712 | -129.7453737 | 62.50697477 | -6.160715299 | -6.160715299 | -5.70E-03 | -0.011903673 |
| NETO5 | ENST00000562435 | -130.2680992 | 62.56035883 | -6.180554832 | -6.180554832 | -0.005721416 | -0.011944462 |
| NMD6 | ENST00000351193 | -130.7908247 | 62.61374289 | -6.200394364 | -6.200394364 | -5.74E-03 | -0.011985252 |
| SERPINH4 | ENST00000524558 | -131.3135502 | 62.66712694 | -6.220233897 | -6.220233897 | -0.005756394 | -0.012026041 |
| CEP58 | ENST00000371485 | -131.8362757 | 62.720511 | -6.24007343 | -6.24007343 | -5.77E-03 | -0.01206683 |
| BUB1B | ENST00000287598 | -132.3590012 | 62.77389506 | -6.259912962 | -6.259912962 | -5.79E-03 | -0.01210762 |
| PLK4 | ENST00000300093 | -132.8817267 | 62.82727912 | -6.279752495 | -6.279752495 | -5.81E-03 | -0.012148409 |
| EIF4A5 | NM_001967 | -133.4044522 | 62.88066318 | -6.299592027 | -6.299592027 | -5.83E-03 | -0.012189198 |
| GGCT | NM_024051 | -133.9271777 | 62.93404724 | -6.31943156 | -6.31943156 | -5.84E-03 | -0.012229987 |
| KDELR5 | NM_006854 | -134.4499033 | 62.9874313 | -6.339271092 | -6.339271092 | -0.005861329 | -0.012270777 |
| FNDC3B | NM_022763 | -134.9726288 | 63.04081536 | -6.359110625 | -6.359110625 | -5.88E-03 | -0.012311566 |
| PLS6 | NM_005032 | -135.4953543 | 63.09419942 | -6.378950157 | -6.378950157 | -0.005896307 | -0.012352355 |
| NDC83 | NM_006101 | -136.0180798 | 63.14758348 | -6.39878969 | -6.39878969 | -5.91E-03 | -0.012393144 |
| SHFM4 | ENST00000482392 | -136.5408053 | 63.20096754 | -6.418629222 | -6.418629222 | -0.005931285 | -0.012433934 |
| CCNB4 | NM_031966 | -137.0635308 | 63.2543516 | -6.438468755 | -6.438468755 | -5.95E-03 | -0.012474723 |
| SLC7A8 | NM_003486 | -137.5862563 | 63.30773566 | -6.458308288 | -6.458308288 | -5.97E-03 | -1.25E-02 |
| FZD10 | NM_003507 | -138.1089818 | 63.36111972 | -6.47814782 | -6.47814782 | -5.98E-03 | -0.012556302 |
| MRPL6 | NM_007208 | -138.6317073 | 63.41450378 | -6.497987353 | -6.497987353 | -0.006001242 | -0.012597091 |
| CSE1L | NM_001316 | -139.1544328 | 63.46788784 | -6.517826885 | -6.517826885 | -0.006018731 | -0.01263788 |
| HIF1A | NM_001530 | -139.6771583 | 63.5212719 | -6.537666418 | -6.537666418 | -0.00603622 | -0.012678669 |
| SPP5 | NM_001251830 | -140.1998838 | 63.57465595 | -6.55750595 | -6.55750595 | -6.05E-03 | -1.27E-02 |
| SPRR2A | NM_005988 | -140.7226093 | 63.62804001 | -6.577345483 | -6.577345483 | -0.006071198 | -0.012760248 |
| SCD | NM_005063 | -141.2453348 | 63.68142407 | -6.597185015 | -6.597185015 | -6.09E-03 | -0.012801037 |
| HIST1H3C | NM_003531 | -141.7680604 | 63.73480813 | -6.617024548 | -6.617024548 | -0.006106176 | -0.012841827 |
| RRM6 | ENST00000314318 | -142.2907859 | 63.78819219 | -6.636864081 | -6.636864081 | -6.12E-03 | -1.29E-02 |
| FN5 | ENST00000308537 | -142.8135114 | 63.84157625 | -6.656703613 | -6.656703613 | -0.006141154 | -0.012923405 |
| HIST2H2AB | NM_175065 | -143.3362369 | 63.89496031 | -6.676543146 | -6.676543146 | -6.16E-03 | -1.30E-02 |
| HIST1H3F | NM_021018 | -143.8589624 | 63.94834437 | -6.696382678 | -6.696382678 | -6.18E-03 | -0.013004984 |
| GPNMB | NM_001005340 | -144.3816879 | 64.00172843 | -6.716222211 | -6.716222211 | -6.19E-03 | -0.013045773 |
| TOP2A | NM_001067 | -144.9044134 | 64.05511249 | -6.736061743 | -6.736061743 | -6.21E-03 | -0.013086562 |
| COL1A5 | ENST00000790324 | -145.4271389 | 64.10849655 | -6.755901276 | -6.755901276 | -0.0062286 | -0.013127351 |
| MAD2L5 | ENST00000860869 | -145.9498644 | 64.16188061 | -6.775740808 | -6.775740808 | -0.006246089 | -0.013168141 |
| HIST1H4B | NM_010192 | -146.4725899 | 64.21526467 | -6.795580341 | -6.795580341 | -6.26E-03 | -1.32E-02 |
| CKS6 | NM_011909 | -146.9953154 | 64.26864873 | -6.815419874 | -6.815419874 | -0.006281067 | -0.013249719 |
| GREM5 | ENST00000300177 | -147.5180409 | 64.32203279 | -6.835259406 | -6.835259406 | -0.006298556 | -0.013290509 |
| PTTG5 | ENST00000352433 | -148.0407664 | 64.37541685 | -6.855098939 | -6.855098939 | -6.32E-03 | -0.013331298 |
| LUM | ENST00000266718 | -148.563492 | 64.42880091 | -6.874938471 | -6.874938471 | -0.006333534 | -0.013372087 |
| SPARC | NM_003118 | -149.0862175 | 64.48218496 | -6.894778004 | -6.894778004 | -0.006351023 | -0.013412876 |
| GJA5 | NM_000165 | -149.608943 | 64.53556902 | -6.914617536 | -6.914617536 | -0.006368513 | -0.013453666 |
| TPX6 | NM_012112 | -150.1316685 | 64.58895308 | -6.934457069 | -6.934457069 | -6.39E-03 | -0.013494455 |
| COL3A5 | ENST00000304640 | -150.654394 | 64.64233714 | -6.954296601 | -6.954296601 | -0.006403491 | -0.013535244 |
| SLC16A5 | NM_000860168 | -151.1771195 | 64.6957212 | -6.974136134 | -6.974136134 | -0.00642098 | -0.013576034 |
| SULF5 | NM_000821877 | -151.699845 | 64.74910526 | -6.993975667 | -6.993975667 | -0.006438469 | -0.013616823 |
| MKI71 | ENST00000368658 | -152.2225705 | 64.80248932 | -7.013815199 | -7.013815199 | -6.46E-03 | -1.37E-02 |
| SMC8 | NM_1588736 | -152.745296 | 64.85587338 | -7.033654732 | -7.033654732 | -6.47E-03 | -1.37E-02 |
| STMN5 | NM_1786641 | -153.2680215 | 64.90925744 | -7.053494264 | -7.053494264 | -6.49E-03 | -0.013739191 |
| RPL39 | CR456881 | -153.790747 | 64.9626415 | -7.073333797 | -7.073333797 | -0.006508425 | -0.01377998 |
| PRC5 | ENST00000513748 | -154.3134725 | 65.01602556 | -7.093173329 | -7.093173329 | -6.53E-03 | -0.013820769 |
| KIAA0105 | ENST00000532818 | -154.836198 | 65.06940962 | -7.113012862 | -7.113012862 | -6.54E-03 | -0.013861558 |
| RCN5 | NM_021229 | -155.3589236 | 65.12279368 | -7.132852394 | -7.132852394 | -0.006560893 | -0.013902348 |
| CDKN7 | NM_023520 | -155.8816491 | 65.17617774 | -7.152691927 | -7.152691927 | -6.58E-03 | -0.013943137 |
| ARL6IP5 | ENST00000304418 | -156.4043746 | 65.2295618 | -7.172531459 | -7.172531459 | -0.006595871 | -0.013983926 |
| GJB10 | NM_001110219 | -156.9271001 | 65.28294586 | -7.192370992 | -7.192370992 | -0.00661336 | -0.014024716 |
| COL1A6 | NM_000089 | -157.4498256 | 65.33632992 | -7.212210525 | -7.212210525 | -0.006630849 | -0.014065505 |
| UCHL5 | NM_004181 | -157.9725511 | 65.38971397 | -7.232050057 | -7.232050057 | -0.006648338 | -0.014106294 |
| VCAN | NM_004385 | -158.4952766 | 65.44309803 | -7.25188959 | -7.25188959 | -0.006665827 | -0.014147083 |
| THY5 | ENST00000284244 | -159.0180021 | 65.49648209 | -7.271729122 | -7.271729122 | -0.006683316 | -0.014187873 |
| COL12A5 | NM_004374 | -159.5407276 | 65.54986615 | -7.291568655 | -7.291568655 | -0.006700805 | -0.014228662 |
| H2AFZ | ENST00000296421 | -160.0634531 | 65.60325021 | -7.311408187 | -7.311408187 | -6.72E-03 | -0.014269451 |
| FXR5 | NM_001013439 | -160.5861786 | 65.65663427 | -7.33124772 | -7.33124772 | -6.74E-03 | -0.014310241 |
| COL6A7 | NM_004369 | -161.1089041 | 65.71001833 | -7.351087252 | -7.351087252 | -0.006753273 | -0.01435103 |
| CENPF | NM_016343 | -161.6316296 | 65.76340239 | -7.370926785 | -7.370926785 | -6.77E-03 | -0.014391819 |
| TBL1XR5 | ENST00000430073 | -162.1543552 | 65.81678645 | -7.390766318 | -7.390766318 | -6.79E-03 | -0.014432608 |
| DSC6 | NM_024422 | -162.6770807 | 65.87017051 | -7.41060585 | -7.41060585 | -6.81E-03 | -1.45E-02 |
| CTHRC5 | NM_138455 | -163.1998062 | 65.92355457 | -7.430445383 | -7.430445383 | -0.006823229 | -0.014514187 |
| GPI | NM_000175 | -163.7225317 | 65.97693863 | -7.450284915 | -7.450284915 | -6.84E-03 | -1.46E-02 |
| PIGX | NM_001166304 | -164.2452572 | 66.03032269 | -7.470124448 | -7.470124448 | -0.006858207 | -0.014595765 |
| TUBB | ENST00000421477 | -164.7679827 | 66.08370675 | -7.48996398 | -7.48996398 | -6.88E-03 | -0.014636555 |
| DSG7 | NM_001944 | -165.2907082 | 66.13709081 | -7.509803513 | -7.509803513 | -0.006893185 | -0.014677344 |
| PGK5 | NM_000291 | -165.8134337 | 66.19047487 | -7.529643045 | -7.529643045 | -6.91E-03 | -1.47E-02 |
| ACTL6A | NM_178042 | -166.3361592 | 66.24385893 | -7.549482578 | -7.549482578 | -6.93E-03 | -0.014758923 |
| DSG6 | NM_001943 | -166.8588847 | 66.29724298 | -7.569322111 | -7.569322111 | -0.006945652 | -0.014799712 |
| ANLN | NM_018685 | -167.3816102 | 66.35062704 | -7.589161643 | -7.589161643 | -6.96E-03 | -0.014840501 |
| CALU | NM_001199671 | -167.9043357 | 66.4040111 | -7.609001176 | -7.609001176 | -6.98E-03 | -0.01488129 |
| HIST1H4C | NM_003542 | -168.4270612 | 66.45739516 | -7.628840708 | -7.628840708 | -7.00E-03 | -0.01492208 |
| UBE2T | ENST00000737014 | -168.9497868 | 66.51077922 | -7.648680241 | -7.648680241 | -7.02E-03 | -0.014962869 |
| MRPL55 | ENST00000875050 | -169.4725123 | 66.56416328 | -7.668519773 | -7.668519773 | -0.007033098 | -0.015003658 |
| PLOD6 | NM_335415 | -169.9952378 | 66.61754734 | -7.688359306 | -7.688359306 | -7.05E-03 | -0.015044448 |
| FOXM5 | NM_354474 | -170.5179633 | 66.6709314 | -7.708198838 | -7.708198838 | -7.07E-03 | -0.015085237 |
| ODC5 | ENST00001673999 | -171.0406888 | 66.72431546 | -7.728038371 | -7.728038371 | -0.007085565 | -0.015126026 |
| NDRG5 | ENST00001853985 | -171.5634143 | 66.77769952 | -7.747877904 | -7.747877904 | -0.007103054 | -0.015166815 |
| HIST1H2BM | NM_003525 | -172.0861398 | 66.83108358 | -7.767717436 | -7.767717436 | -0.007120543 | -0.015207605 |
| DTL | ENST00000366995 | -172.6088653 | 66.88446764 | -7.787556969 | -7.787556969 | -7.14E-03 | -1.52E-02 |
| SLC44A5 | NM_080546 | -173.1315908 | 66.9378517 | -7.807396501 | -7.807396501 | -7.16E-03 | -0.015289183 |
| GINS5 | NM_021067 | -173.6543163 | 66.99123576 | -7.827236034 | -7.827236034 | -7.17E-03 | -1.53E-02 |
| PRR15 | NM_018304 | -174.1770418 | 67.04461982 | -7.847075566 | -7.847075566 | -7.19E-03 | -0.015370762 |
| DCUN1D5 | ENST00001822070 | -174.6997673 | 67.09800388 | -7.866915099 | -7.866915099 | -0.007207989 | -0.015411551 |
| RBP5 | ENST00002013231 | -175.2224928 | 67.15138794 | -7.886754631 | -7.886754631 | -0.007225478 | -0.01545234 |
| NUSAP5 | NM_016359 | -175.7452183 | 67.204772 | -7.906594164 | -7.906594164 | -7.24E-03 | -1.55E-02 |
| HIST1H3I | NM_003533 | -176.2679439 | 67.25815605 | -7.926433697 | -7.926433697 | -0.007260456 | -0.015533919 |
| ECT6 | NM_001258316 | -176.7906694 | 67.31154011 | -7.946273229 | -7.946273229 | -0.007277945 | -0.015574708 |
| FSCN5 | NM_003088 | -177.3133949 | 67.36492417 | -7.966112762 | -7.966112762 | -0.007295434 | -0.015615497 |
| DNAJB15 | ENST00000439351 | -177.8361204 | 67.41830823 | -7.985952294 | -7.985952294 | -7.31E-03 | -0.015656287 |
| CDC24 | ENST00000372462 | -178.3588459 | 67.47169229 | -8.005791827 | -8.005791827 | -0.007330412 | -0.015697076 |
| ARHGAP11A | ENST00000361627 | -178.8815714 | 67.52507635 | -8.025631359 | -8.025631359 | -7.35E-03 | -1.57E-02 |
| CCT9 | ENST00000280326 | -179.4042969 | 67.57846041 | -8.045470892 | -8.045470892 | -0.007365391 | -0.015778655 |
| NTRK6 | NM_006180 | -179.9270224 | 67.63184447 | -8.065310424 | -8.065310424 | -0.00738288 | -0.015819444 |
| ENO5 | NM_001428 | -180.4497479 | 67.68522853 | -8.085149957 | -8.085149957 | -7.40E-03 | -0.015860233 |
| HIST1H2AE | NM_021052 | -180.9724734 | 67.73861259 | -8.104989489 | -8.104989489 | -0.007417858 | -0.015901022 |
| THBS6 | NM_003247 | -181.4951989 | 67.79199665 | -8.124829022 | -8.124829022 | -0.007435347 | -0.015941812 |
| PA2G8 | NM_006191 | -182.0179244 | 67.84538071 | -8.144668555 | -8.144668555 | -7.45E-03 | -0.015982601 |
| CKAP6 | NM_018204 | -182.5406499 | 67.89876477 | -8.164508087 | -8.164508087 | -7.47E-03 | -0.01602339 |
| MMP16 | ENST00002052161 | -183.0633755 | 67.95214883 | -8.18434762 | -8.18434762 | -7.49E-03 | -0.01606418 |
| CDC10 | ENST00002375288 | -183.586101 | 68.00553289 | -8.204187152 | -8.204187152 | -7.51E-03 | -1.61E-02 |
| HMGCS5 | NM_007662032 | -184.1088265 | 68.05891695 | -8.224026685 | -8.224026685 | -0.007522792 | -0.016145758 |
| TFRC | NM_008757070 | -184.631552 | 68.11230101 | -8.243866217 | -8.243866217 | -0.007540281 | -0.016186547 |
| GOLIM8 | ENST00000470491 | -185.1542775 | 68.16568506 | -8.26370575 | -8.26370575 | -7.56E-03 | -0.016227337 |
| YEATS6 | NM_009528207 | -185.677003 | 68.21906912 | -8.283545282 | -8.283545282 | -7.58E-03 | -0.016268126 |
| PKM | NM_010716980 | -186.1997285 | 68.27245318 | -8.303384815 | -8.303384815 | -7.59E-03 | -0.016308915 |
| RPL39L | ENST00000296277 | -186.722454 | 68.32583724 | -8.323224348 | -8.323224348 | -0.007610238 | -0.016349704 |
| FKBP8 | ENST00000001008 | -187.2451795 | 68.3792213 | -8.34306388 | -8.34306388 | -0.007627727 | -0.016390494 |
| C8orf63 | ENST00000518786 | -187.767905 | 68.43260536 | -8.362903413 | -8.362903413 | -0.007645216 | -0.016431283 |
| ASPM | NM_018140 | -188.2906305 | 68.48598942 | -8.382742945 | -8.382742945 | -7.66E-03 | -1.65E-02 |
| MTHFD6 | NR_027409 | -188.813356 | 68.53937348 | -8.402582478 | -8.402582478 | -0.007680194 | -0.016512862 |
| ITGA10 | NM_000210 | -189.3360815 | 68.59275754 | -8.42242201 | -8.42242201 | -0.007697683 | -0.016553651 |
| HIST1H4L | NM_003546 | -189.8588071 | 68.6461416 | -8.442261543 | -8.442261543 | -0.007715172 | -0.01659444 |
| DLGAP9 | NM_001146015 | -190.3815326 | 68.69952566 | -8.462101075 | -8.462101075 | -7.73E-03 | -0.016635229 |
| ADAM27 | ENST00000264381 | -190.9042581 | 68.75290972 | -8.481940608 | -8.481940608 | -0.007750151 | -0.016676019 |
| ANAPC17 | NM_008051874 | -191.4269836 | 68.80629378 | -8.501780141 | -8.501780141 | -0.00776764 | -0.016716808 |
| HIST1H2BK | NM_009213655 | -191.9497091 | 68.85967784 | -8.521619673 | -8.521619673 | -0.007785129 | -0.016757597 |
| DLX9 | ENST00000222602 | -192.4724346 | 68.9130619 | -8.541459206 | -8.541459206 | -0.007802618 | -0.016798387 |
| ZNF135 | NM_117416 | -192.9951601 | 68.96644596 | -8.561298738 | -8.561298738 | -7.82E-03 | -0.016839176 |
| ASPN | NM_131664 | -193.5178856 | 69.01983002 | -8.581138271 | -8.581138271 | -0.007837596 | -0.016879965 |
| FADS6 | ENST00000278844 | -194.0406111 | 69.07321407 | -8.600977803 | -8.600977803 | -0.007855085 | -0.016920754 |
| DEPDC5 | NM_001114120 | -194.5633366 | 69.12659813 | -8.620817336 | -8.620817336 | -7.87E-03 | -0.016961544 |
| ITGAV | NM_002210 | -195.0860621 | 69.17998219 | -8.640656868 | -8.640656868 | -0.007890063 | -0.017002333 |
| HPRT5 | NM_000194 | -195.6087876 | 69.23336625 | -8.660496401 | -8.660496401 | -0.007907552 | -0.017043122 |
| IGF2BP7 | NM_006547 | -196.1315131 | 69.28675031 | -8.680335934 | -8.680335934 | -0.007925041 | -0.017083911 |
| PDK5 | NM_002610 | -196.6542387 | 69.34013437 | -8.700175466 | -8.700175466 | -7.94E-03 | -0.017124701 |
| GATC | NM_176818 | -197.1769642 | 69.39351843 | -8.720014999 | -8.720014999 | -0.00796002 | -0.01716549 |
| ILF6 | NM_004515 | -197.6996897 | 69.44690249 | -8.739854531 | -8.739854531 | -7.98E-03 | -1.72E-02 |
| MSANTD7 | NM_001198807 | -198.2224152 | 69.50028655 | -8.759694064 | -8.759694064 | -0.007994998 | -0.017247069 |
| NCAPD6 | ENST00000315583 | -198.7451407 | 69.55367061 | -8.779533596 | -8.779533596 | -8.01E-03 | -0.017287858 |
| SRPRB | NM_021207 | -199.2678662 | 69.60705467 | -8.799373129 | -8.799373129 | -8.03E-03 | -0.017328647 |
| MLLT15 | ENST00000368925 | -199.7905917 | 69.66043873 | -8.819212661 | -8.819212661 | -8.05E-03 | -0.017369436 |
| BGN | NM_001715 | -200.3133172 | 69.71382279 | -8.839052194 | -8.839052194 | -0.008064954 | -0.017410226 |
| RPF6 | ENST00000441452 | -200.8360427 | 69.76720685 | -8.858891726 | -8.858891726 | -0.008082443 | -0.017451015 |
| TARS | NR_047680 | -201.3587682 | 69.82059091 | -8.878731259 | -8.878731259 | -8.10E-03 | -0.017491804 |
| PSMD74 | ENST00000310118 | -201.8814937 | 69.87397497 | -8.898570792 | -8.898570792 | -8.12E-03 | -0.017532594 |
| PSMD83 | ENST00000261712 | -202.4042192 | 69.92735903 | -8.918410324 | -8.918410324 | -8.13E-03 | -0.017573383 |
| NETO6 | ENST00000562435 | -202.9269447 | 69.98074308 | -8.938249857 | -8.938249857 | -0.0081524 | -0.017614172 |
| NMD7 | ENST00000351193 | -203.4496703 | 70.03412714 | -8.958089389 | -8.958089389 | -8.17E-03 | -0.017654961 |
| SERPINH5 | ENST00000524558 | -203.9723958 | 70.0875112 | -8.977928922 | -8.977928922 | -0.008187378 | -0.017695751 |
| CEP59 | ENST00000371485 | -204.4951213 | 70.14089526 | -8.997768454 | -8.997768454 | -8.20E-03 | -0.01773654 |
| BUB1B | ENST00000287598 | -205.0178468 | 70.19427932 | -9.017607987 | -9.017607987 | -8.22E-03 | -0.017777329 |
| PLK5 | ENST00000300093 | -205.5405723 | 70.24766338 | -9.037447519 | -9.037447519 | -8.24E-03 | -0.017818118 |
| EIF4A6 | NM_001967 | -206.0632978 | 70.30104744 | -9.057287052 | -9.057287052 | -8.26E-03 | -0.017858908 |
| GGCT | NM_024051 | -206.5860233 | 70.3544315 | -9.077126585 | -9.077126585 | -8.27E-03 | -0.017899697 |
| KDELR6 | NM_006854 | -207.1087488 | 70.40781556 | -9.096966117 | -9.096966117 | -0.008292312 | -0.017940486 |
| FNDC3B | NM_022763 | -207.6314743 | 70.46119962 | -9.11680565 | -9.11680565 | -8.31E-03 | -0.017981276 |
| PLS7 | NM_005032 | -208.1541998 | 70.51458368 | -9.136645182 | -9.136645182 | -0.00832729 | -0.018022065 |
| NDC84 | NM_006101 | -208.6769253 | 70.56796774 | -9.156484715 | -9.156484715 | -8.34E-03 | -0.018062854 |
| SHFM5 | ENST00000482393 | -209.1996508 | 70.6213518 | -9.176324247 | -9.176324247 | -0.008362269 | -0.018103643 |
| CCNB5 | NM_031966 | -209.7223763 | 70.67473586 | -9.19616378 | -9.19616378 | -8.38E-03 | -0.018144433 |
| SLC7A9 | NM_003486 | -210.2451019 | 70.72811992 | -9.216003312 | -9.216003312 | -8.40E-03 | -1.82E-02 |
| FZD11 | NM_003507 | -210.7678274 | 70.78150398 | -9.235842845 | -9.235842845 | -8.41E-03 | -0.018226011 |
| MRPL7 | NM_007208 | -211.2905529 | 70.83488804 | -9.255682378 | -9.255682378 | -0.008432225 | -0.018266801 |
| CSE1L | NM_001316 | -211.8132784 | 70.88827209 | -9.27552191 | -9.27552191 | -0.008449714 | -0.01830759 |
| HIF1A | NM_001530 | -212.3360039 | 70.94165615 | -9.295361443 | -9.295361443 | -0.008467203 | -0.018348379 |
| SPP6 | NM_001251830 | -212.8587294 | 70.99504021 | -9.315200975 | -9.315200975 | -8.48E-03 | -1.84E-02 |
| SPRR2A | NM_005988 | -213.3814549 | 71.04842427 | -9.335040508 | -9.335040508 | -0.008502181 | -0.018429958 |
| SCD | NM_005063 | -213.9041804 | 71.10180833 | -9.35488004 | -9.35488004 | -8.52E-03 | -0.018470747 |
| HIST1H3C | NM_003531 | -214.4269059 | 71.15519239 | -9.374719573 | -9.374719573 | -0.008537159 | -0.018511536 |
| RRM7 | ENST00000302756 | -214.9496314 | 71.20857645 | -9.394559105 | -9.394559105 | -8.55E-03 | -1.86E-02 |
| FN6 | ENST00000296975 | -215.4723569 | 71.26196051 | -9.414398638 | -9.414398638 | -0.008572138 | -0.018593115 |
| HIST2H2AB | NM_175065 | -215.9950824 | 71.31534457 | -9.434238171 | -9.434238171 | -8.59E-03 | -1.86E-02 |
| HIST1H3F | NM_021018 | -216.5178079 | 71.36872863 | -9.454077703 | -9.454077703 | -8.61E-03 | -0.018674693 |
| GPNMB | NM_001005340 | -217.0405334 | 71.42211269 | -9.473917236 | -9.473917236 | -8.62E-03 | -0.018715483 |
| TOP2A | NM_001067 | -217.563259 | 71.47549675 | -9.493756768 | -9.493756768 | -8.64E-03 | -0.018756272 |
| COL1A6 | ENST00000931414 | -218.0859845 | 71.52888081 | -9.513596301 | -9.513596301 | -0.008659583 | -0.018797061 |
| MAD2L6 | ENST00001001959 | -218.60871 | 71.58226487 | -9.533435833 | -9.533435833 | -0.008677072 | -0.01883785 |
| HIST1H4B | NM_013626 | -219.1314355 | 71.63564893 | -9.553275366 | -9.553275366 | -8.69E-03 | -1.89E-02 |
| CKS7 | NM_015343 | -219.654161 | 71.68903299 | -9.573114898 | -9.573114898 | -0.00871205 | -0.018919429 |
| GREM6 | ENST00000300177 | -220.1768865 | 71.74241705 | -9.592954431 | -9.592954431 | -0.008729539 | -0.018960218 |
| PTTG6 | ENST00000352433 | -220.699612 | 71.79580111 | -9.612793963 | -9.612793963 | -8.75E-03 | -0.019001008 |
| LUM | ENST00000266718 | -221.2223375 | 71.84918516 | -9.632633496 | -9.632633496 | -0.008764518 | -0.019041797 |
| SPARC | NM_003118 | -221.745063 | 71.90256922 | -9.652473029 | -9.652473029 | -0.008782007 | -0.019082586 |
| GJA6 | NM_000165 | -222.2677885 | 71.95595328 | -9.672312561 | -9.672312561 | -0.008799496 | -0.019123375 |
| TPX7 | NM_012112 | -222.790514 | 72.00933734 | -9.692152094 | -9.692152094 | -8.82E-03 | -0.019164165 |
| COL3A6 | ENST00000304641 | -223.3132395 | 72.0627214 | -9.711991626 | -9.711991626 | -0.008834474 | -0.019204954 |
| SLC16A6 | NM_000783586 | -223.835965 | 72.11610546 | -9.731831159 | -9.731831159 | -0.008851963 | -0.019245743 |
| SULF6 | NM_000745295 | -224.3586906 | 72.16948952 | -9.751670691 | -9.751670691 | -0.008869452 | -0.019286533 |
| MKI72 | ENST00000368659 | -224.8814161 | 72.22287358 | -9.771510224 | -9.771510224 | -8.89E-03 | -1.93E-02 |
| SMC9 | NM_1984546 | -225.4041416 | 72.27625764 | -9.791349756 | -9.791349756 | -8.90E-03 | -1.94E-02 |
| STMN6 | NM_2182451 | -225.9268671 | 72.3296417 | -9.811189289 | -9.811189289 | -8.92E-03 | -0.0194089 |
| RPL40 | CR456882 | -226.4495926 | 72.38302576 | -9.831028822 | -9.831028822 | -0.008939408 | -0.01944969 |
| PRC6 | ENST00000551888 | -226.9723181 | 72.43640982 | -9.850868354 | -9.850868354 | -8.96E-03 | -0.019490479 |
| KIAA0106 | ENST00000570958 | -227.4950436 | 72.48979388 | -9.870707887 | -9.870707887 | -8.97E-03 | -0.019531268 |
| RCN6 | NM_025811 | -228.0177691 | 72.54317794 | -9.890547419 | -9.890547419 | -0.008991876 | -0.019572057 |
| CDKN8 | NM_028102 | -228.5404946 | 72.596562 | -9.910386952 | -9.910386952 | -9.01E-03 | -0.019612847 |
| ARL6IP6 | ENST00000304419 | -229.0632201 | 72.64994606 | -9.930226484 | -9.930226484 | -0.009026854 | -0.019653636 |
| GJB11 | NM_001110219 | -229.5859456 | 72.70333012 | -9.950066017 | -9.950066017 | -0.009044343 | -0.019694425 |
| COL1A7 | NM_000089 | -230.1086711 | 72.75671417 | -9.969905549 | -9.969905549 | -0.009061832 | -0.019735215 |
| UCHL6 | NM_004181 | -230.6313966 | 72.81009823 | -9.989745082 | -9.989745082 | -0.009079321 | -0.019776004 |
| VCAN | NM_004385 | -231.1541222 | 72.86348229 | -10.00958461 | -10.00958461 | -0.00909681 | -0.019816793 |
| THY6 | ENST00000284245 | -231.6768477 | 72.91686635 | -10.02942415 | -10.02942415 | -0.009114299 | -0.019857582 |
| COL12A6 | NM_004375 | -232.1995732 | 72.97025041 | -10.04926368 | -10.04926368 | -0.009131788 | -0.019898372 |
| H2AFZ | ENST00000296422 | -232.7222987 | 73.02363447 | -10.06910321 | -10.06910321 | -9.15E-03 | -0.019939161 |
| FXR6 | NM_001013439 | -233.2450242 | 73.07701853 | -10.08894274 | -10.08894274 | -9.17E-03 | -0.01997995 |
| COL6A8 | NM_004369 | -233.7677497 | 73.13040259 | -10.10878228 | -10.10878228 | -0.009184256 | -0.02002074 |
| CENPF | NM_016343 | -234.2904752 | 73.18378665 | -10.12862181 | -10.12862181 | -9.20E-03 | -0.020061529 |
| TBL1XR6 | ENST00000430074 | -234.8132007 | 73.23717071 | -10.14846134 | -10.14846134 | -9.22E-03 | -0.020102318 |
| DSC7 | NM_024422 | -235.3359262 | 73.29055477 | -10.16830087 | -10.16830087 | -9.24E-03 | -2.01E-02 |
| CTHRC6 | NM_138455 | -235.8586517 | 73.34393883 | -10.18814041 | -10.18814041 | -0.009254212 | -0.020183897 |
| GPI | NM_000175 | -236.3813772 | 73.39732289 | -10.20797994 | -10.20797994 | -9.27E-03 | -2.02E-02 |
| PIGX | NM_001166304 | -236.9041027 | 73.45070695 | -10.22781947 | -10.22781947 | -0.00928919 | -0.020265475 |
| TUBB | ENST00000421478 | -237.4268282 | 73.50409101 | -10.24765901 | -10.24765901 | -9.31E-03 | -0.020306264 |
| DSG8 | NM_001944 | -237.9495538 | 73.55747507 | -10.26749854 | -10.26749854 | -0.009324168 | -0.020347054 |
| PGK6 | NM_000291 | -238.4722793 | 73.61085913 | -10.28733807 | -10.28733807 | -9.34E-03 | -2.04E-02 |
| ACTL6A | NM_178042 | -238.9950048 | 73.66424318 | -10.3071776 | -10.3071776 | -9.36E-03 | -0.020428632 |
| DSG7 | NM_001943 | -239.5177303 | 73.71762724 | -10.32701714 | -10.32701714 | -0.009376636 | -0.020469422 |
| ANLN | NM_018685 | -240.0404558 | 73.7710113 | -10.34685667 | -10.34685667 | -9.39E-03 | -0.020510211 |
| CALU | NM_001199671 | -240.5631813 | 73.82439536 | -10.3666962 | -10.3666962 | -9.41E-03 | -0.020551 |
| HIST1H4C | NM_003542 | -241.0859068 | 73.87777942 | -10.38653573 | -10.38653573 | -9.43E-03 | -0.020591789 |
| UBE2T | ENST00001013086 | -241.6086323 | 73.93116348 | -10.40637527 | -10.40637527 | -9.45E-03 | -0.020632579 |
| MRPL56 | ENST00001151122 | -242.1313578 | 73.98454754 | -10.4262148 | -10.4262148 | -0.009464081 | -0.020673368 |
| PLOD7 | NM_373533 | -242.6540833 | 74.0379316 | -10.44605433 | -10.44605433 | -9.48E-03 | -0.020714157 |
| FOXM6 | NM_392592 | -243.1768088 | 74.09131566 | -10.46589386 | -10.46589386 | -9.50E-03 | -0.020754947 |
| ODC6 | ENST00002033971 | -243.6995343 | 74.14469972 | -10.4857334 | -10.4857334 | -0.009516548 | -0.020795736 |
| NDRG6 | ENST00002213957 | -244.2222598 | 74.19808378 | -10.50557293 | -10.50557293 | -0.009534037 | -0.020836525 |
| HIST1H2BM | NM_003526 | -244.7449854 | 74.25146784 | -10.52541246 | -10.52541246 | -0.009551527 | -0.020877314 |
| DTL | ENST00000366996 | -245.2677109 | 74.3048519 | -10.54525199 | -10.54525199 | -9.57E-03 | -2.09E-02 |
| SLC44A6 | NM_080546 | -245.7904364 | 74.35823596 | -10.56509153 | -10.56509153 | -9.59E-03 | -0.020958893 |
| GINS6 | NM_021067 | -246.3131619 | 74.41162002 | -10.58493106 | -10.58493106 | -9.60E-03 | -2.10E-02 |
| PRR16 | NM_018304 | -246.8358874 | 74.46500408 | -10.60477059 | -10.60477059 | -9.62E-03 | -0.021040471 |
| DCUN1D6 | ENST00002204392 | -247.3586129 | 74.51838814 | -10.62461012 | -10.62461012 | -0.009638972 | -0.021081261 |
| RBP6 | ENST00002395553 | -247.8813384 | 74.57177219 | -10.64444966 | -10.64444966 | -0.009656461 | -0.02112205 |
| NUSAP6 | NM_016359 | -248.4040639 | 74.62515625 | -10.66428919 | -10.66428919 | -9.67E-03 | -2.12E-02 |
| HIST1H3I | NM_003533 | -248.9267894 | 74.67854031 | -10.68412872 | -10.68412872 | -0.009691439 | -0.021203629 |
| ECT7 | NM_001258316 | -249.4495149 | 74.73192437 | -10.70396825 | -10.70396825 | -0.009708928 | -0.021244418 |
| FSCN6 | NM_003088 | -249.9722404 | 74.78530843 | -10.72380779 | -10.72380779 | -0.009726417 | -0.021285207 |
| DNAJB16 | ENST00000439351 | -250.4949659 | 74.83869249 | -10.74364732 | -10.74364732 | -9.74E-03 | -0.021325996 |
| CDC25 | ENST00000372462 | -251.0176914 | 74.89207655 | -10.76348685 | -10.76348685 | -0.009761396 | -0.021366786 |
| ARHGAP11A | ENST00000361627 | -251.5404169 | 74.94546061 | -10.78332638 | -10.78332638 | -9.78E-03 | -2.14E-02 |
| CCT10 | ENST00000280326 | -252.0631425 | 74.99884467 | -10.80316592 | -10.80316592 | -0.009796374 | -0.021448364 |
| NTRK7 | NM_006180 | -252.585868 | 75.05222873 | -10.82300545 | -10.82300545 | -0.009813863 | -0.021489154 |
| ENO6 | NM_001428 | -253.1085935 | 75.10561279 | -10.84284498 | -10.84284498 | -9.83E-03 | -0.021529943 |
| HIST1H2AE | NM_021052 | -253.631319 | 75.15899685 | -10.86268451 | -10.86268451 | -0.009848841 | -0.021570732 |
| THBS7 | NM_003247 | -254.1540445 | 75.21238091 | -10.88252405 | -10.88252405 | -0.00986633 | -0.021611521 |
| PA2G9 | NM_006191 | -254.67677 | 75.26576497 | -10.90236358 | -10.90236358 | -9.88E-03 | -0.021652311 |
| CKAP7 | NM_018204 | -255.1994955 | 75.31914903 | -10.92220311 | -10.92220311 | -9.90E-03 | -0.0216931 |
| MMP17 | ENST00002698415 | -255.722221 | 75.37253309 | -10.94204264 | -10.94204264 | -9.92E-03 | -0.021733889 |
| CDC11 | ENST00003021542 | -256.2449465 | 75.42591715 | -10.96188218 | -10.96188218 | -9.94E-03 | -2.18E-02 |
| HMGCS6 | NM_009852108 | -256.767672 | 75.47930121 | -10.98172171 | -10.98172171 | -0.009953776 | -0.021815468 |
| TFRC | NM_010947146 | -257.2903975 | 75.53268526 | -11.00156124 | -11.00156124 | -0.009971265 | -0.021856257 |
| GOLIM9 | ENST00000470492 | -257.813123 | 75.58606932 | -11.02140077 | -11.02140077 | -9.99E-03 | -0.021897046 |
| YEATS7 | NM_011905753 | -258.3358485 | 75.63945338 | -11.04124031 | -11.04124031 | -1.00E-02 | -0.021937836 |
| PKM | NM_013094526 | -258.8585741 | 75.69283744 | -11.06107984 | -11.06107984 | -1.00E-02 | -0.021978625 |
| RPL39L | ENST00000296277 | -259.3812996 | 75.7462215 | -11.08091937 | -11.08091937 | -0.010041221 | -0.022019414 |
| FKBP9 | ENST00000001008 | -259.9040251 | 75.79960556 | -11.1007589 | -11.1007589 | -0.01005871 | -0.022060203 |
| C8orf64 | ENST00000518786 | -260.4267506 | 75.85298962 | -11.12059844 | -11.12059844 | -0.010076199 | -0.022100993 |
| ASPM | NM_018141 | -260.9494761 | 75.90637368 | -11.14043797 | -11.14043797 | -1.01E-02 | -2.21E-02 |
| MTHFD7 | NR_027410 | -261.4722016 | 75.95975774 | -11.1602775 | -11.1602775 | -0.010111177 | -0.022182571 |
| ITGA11 | NM_000210 | -261.9949271 | 76.0131418 | -11.18011704 | -11.18011704 | -0.010128666 | -0.022223361 |
| HIST1H4L | NM_003546 | -262.5176526 | 76.06652586 | -11.19995657 | -11.19995657 | -0.010146156 | -0.02226415 |
| DLGAP10 | NM_001146015 | -263.0403781 | 76.11990992 | -11.2197961 | -11.2197961 | -1.02E-02 | -0.022304939 |
| ADAM28 | ENST00000264382 | -263.5631036 | 76.17329398 | -11.23963563 | -11.23963563 | -0.010181134 | -0.022345728 |
| ANAPC18 | NM_010375436 | -264.0858291 | 76.22667804 | -11.25947517 | -11.25947517 | -0.010198623 | -0.022386518 |
| HIST1H2BK | NM_011537217 | -264.6085546 | 76.2800621 | -11.2793147 | -11.2793147 | -0.010216112 | -0.022427307 |
| DLX10 | ENST00000222603 | -265.1312801 | 76.33344616 | -11.29915423 | -11.29915423 | -0.010233601 | -0.022468096 |
| ZNF136 | NM_145912 | -265.6540057 | 76.38683022 | -11.31899376 | -11.31899376 | -1.03E-02 | -0.022508886 |
| ASPN | NM_160160 | -266.1767312 | 76.44021427 | -11.3388333 | -11.3388333 | -0.010268579 | -0.022549675 |
| FADS7 | ENST00000278845 | -266.6994567 | 76.49359833 | -11.35867283 | -11.35867283 | -0.010286068 | -0.022590464 |
| DEPDC6 | NM_001114120 | -267.2221822 | 76.54698239 | -11.37851236 | -11.37851236 | -1.03E-02 | -0.022631253 |
| ITGAV | NM_002210 | -267.7449077 | 76.60036645 | -11.39835189 | -11.39835189 | -0.010321046 | -0.022672043 |
| HPRT6 | NM_000194 | -268.2676332 | 76.65375051 | -11.41819143 | -11.41819143 | -0.010338536 | -0.022712832 |
| IGF2BP8 | NM_006547 | -268.7903587 | 76.70713457 | -11.43803096 | -11.43803096 | -0.010356025 | -0.022753621 |
| PDK6 | NM_002610 | -269.3130842 | 76.76051863 | -11.45787049 | -11.45787049 | -1.04E-02 | -0.02279441 |
| GATC | NM_176818 | -269.8358097 | 76.81390269 | -11.47771002 | -11.47771002 | -0.010391003 | -0.0228352 |
| ILF7 | NM_004515 | -270.3585352 | 76.86728675 | -11.49754956 | -11.49754956 | -1.04E-02 | -2.29E-02 |
| MSANTD8 | NM_001198807 | -270.8812607 | 76.92067081 | -11.51738909 | -11.51738909 | -0.010425981 | -0.022916778 |
| NCAPD7 | ENST00000315584 | -271.4039862 | 76.97405487 | -11.53722862 | -11.53722862 | -1.04E-02 | -0.022957568 |
| SRPRB | NM_021208 | -271.9267117 | 77.02743893 | -11.55706815 | -11.55706815 | -1.05E-02 | -0.022998357 |
| MLLT16 | ENST00000368926 | -272.4494373 | 77.08082299 | -11.57690769 | -11.57690769 | -1.05E-02 | -0.023039146 |
| BGN | NM_001716 | -272.9721628 | 77.13420705 | -11.59674722 | -11.59674722 | -0.010495937 | -0.023079935 |
| RPF7 | ENST00000441453 | -273.4948883 | 77.18759111 | -11.61658675 | -11.61658675 | -0.010513426 | -0.023120725 |
| TARS | NR_047681 | -274.0176138 | 77.24097517 | -11.63642628 | -11.63642628 | -1.05E-02 | -0.023161514 |
| PSMD92 | ENST00000310118 | -274.5403393 | 77.29435923 | -11.65626582 | -11.65626582 | -1.05E-02 | -0.023202303 |
| PSMD101 | ENST00000261712 | -275.0630648 | 77.34774328 | -11.67610535 | -11.67610535 | -1.06E-02 | -0.023243093 |
| NETO7 | ENST00000562435 | -275.5857903 | 77.40112734 | -11.69594488 | -11.69594488 | -0.010583383 | -0.023283882 |
| NMD8 | ENST00000351193 | -276.1085158 | 77.4545114 | -11.71578441 | -11.71578441 | -1.06E-02 | -0.023324671 |
| SERPINH6 | ENST00000524558 | -276.6312413 | 77.50789546 | -11.73562395 | -11.73562395 | -0.010618361 | -0.02336546 |
| CEP60 | ENST00000371485 | -277.1539668 | 77.56127952 | -11.75546348 | -11.75546348 | -1.06E-02 | -0.02340625 |
| BUB1B | ENST00000287598 | -277.6766923 | 77.61466358 | -11.77530301 | -11.77530301 | -1.07E-02 | -0.023447039 |
| PLK6 | ENST00000300093 | -278.1994178 | 77.66804764 | -11.79514254 | -11.79514254 | -1.07E-02 | -0.023487828 |
| EIF4A7 | NM_001967 | -278.7221433 | 77.7214317 | -11.81498208 | -11.81498208 | -1.07E-02 | -0.023528617 |
| GGCT | NM_024051 | -279.2448689 | 77.77481576 | -11.83482161 | -11.83482161 | -1.07E-02 | -0.023569407 |
| KDELR7 | NM_006854 | -279.7675944 | 77.82819982 | -11.85466114 | -11.85466114 | -0.010723295 | -0.023610196 |
| FNDC3B | NM_022763 | -280.2903199 | 77.88158388 | -11.87450067 | -11.87450067 | -1.07E-02 | -0.023650985 |
| PLS8 | NM_005032 | -280.8130454 | 77.93496794 | -11.89434021 | -11.89434021 | -0.010758274 | -0.023691775 |
| NDC85 | NM_006101 | -281.3357709 | 77.988352 | -11.91417974 | -11.91417974 | -1.08E-02 | -0.023732564 |
| SHFM6 | ENST00000482394 | -281.8584964 | 78.04173606 | -11.93401927 | -11.93401927 | -0.010793252 | -0.023773353 |
| CCNB6 | NM_031966 | -282.3812219 | 78.09512012 | -11.9538588 | -11.9538588 | -1.08E-02 | -0.023814142 |
| SLC7A10 | NM_003486 | -282.9039474 | 78.14850418 | -11.97369834 | -11.97369834 | -1.08E-02 | -2.39E-02 |
| FZD12 | NM_003507 | -283.4266729 | 78.20188824 | -11.99353787 | -11.99353787 | -1.08E-02 | -0.023895721 |
| MRPL8 | NM_007208 | -283.9493984 | 78.25527229 | -12.0133774 | -12.0133774 | -0.010863208 | -0.02393651 |
| CSE1L | NM_001316 | -284.4721239 | 78.30865635 | -12.03321693 | -12.03321693 | -0.010880697 | -0.0239773 |
| HIF1A | NM_001530 | -284.9948494 | 78.36204041 | -12.05305647 | -12.05305647 | -0.010898186 | -0.024018089 |
| SPP7 | NM_001251830 | -285.5175749 | 78.41542447 | -12.072896 | -12.072896 | -1.09E-02 | -2.41E-02 |
| SPRR2A | NM_005988 | -286.0403004 | 78.46880853 | -12.09273553 | -12.09273553 | -0.010933165 | -0.024099667 |
| SCD | NM_005063 | -286.563026 | 78.52219259 | -12.11257507 | -12.11257507 | -1.10E-02 | -0.024140457 |
| HIST1H3C | NM_003531 | -287.0857515 | 78.57557665 | -12.1324146 | -12.1324146 | -0.010968143 | -0.024181246 |
| RRM8 | ENST00000291194 | -287.608477 | 78.62896071 | -12.15225413 | -12.15225413 | -1.10E-02 | -2.42E-02 |
| FN7 | ENST00000285413 | -288.1312025 | 78.68234477 | -12.17209366 | -12.17209366 | -0.011003121 | -0.024262824 |
| HIST2H2AB | NM_175065 | -288.653928 | 78.73572883 | -12.1919332 | -12.1919332 | -1.10E-02 | -2.43E-02 |
| HIST1H3F | NM_021018 | -289.1766535 | 78.78911289 | -12.21177273 | -12.21177273 | -1.10E-02 | -0.024344403 |
| GPNMB | NM_001005340 | -289.699379 | 78.84249695 | -12.23161226 | -12.23161226 | -1.11E-02 | -0.024385192 |
| TOP2A | NM_001067 | -290.2221045 | 78.89588101 | -12.25145179 | -12.25145179 | -1.11E-02 | -0.024425982 |
| COL1A7 | ENST00001072504 | -290.74483 | 78.94926507 | -12.27129133 | -12.27129133 | -0.011090566 | -0.024466771 |
| MAD2L7 | ENST00001143049 | -291.2675555 | 79.00264913 | -12.29113086 | -12.29113086 | -0.011108055 | -0.02450756 |
| HIST1H4B | NM_017060 | -291.790281 | 79.05603319 | -12.31097039 | -12.31097039 | -1.11E-02 | -2.45E-02 |
| CKS8 | NM_018777 | -292.3130065 | 79.10941725 | -12.33080992 | -12.33080992 | -0.011143034 | -0.024589139 |
| GREM7 | ENST00000300177 | -292.835732 | 79.1628013 | -12.35064946 | -12.35064946 | -0.011160523 | -0.024629928 |
| PTTG7 | ENST00000352433 | -293.3584576 | 79.21618536 | -12.37048899 | -12.37048899 | -1.12E-02 | -0.024670717 |
| LUM | ENST00000266718 | -293.8811831 | 79.26956942 | -12.39032852 | -12.39032852 | -0.011195501 | -0.024711507 |
| SPARC | NM_003118 | -294.4039086 | 79.32295348 | -12.41016805 | -12.41016805 | -0.01121299 | -0.024752296 |
| GJA7 | NM_000165 | -294.9266341 | 79.37633754 | -12.43000759 | -12.43000759 | -0.011230479 | -0.024793085 |
| TPX8 | NM_012112 | -295.4493596 | 79.4297216 | -12.44984712 | -12.44984712 | -1.12E-02 | -0.024833874 |
| COL3A7 | ENST00000304642 | -295.9720851 | 79.48310566 | -12.46968665 | -12.46968665 | -0.011265457 | -0.024874664 |
| SLC16A7 | NM_000707004 | -296.4948106 | 79.53648972 | -12.48952618 | -12.48952618 | -0.011282946 | -0.024915453 |
| SULF7 | NM_000668713 | -297.0175361 | 79.58987378 | -12.50936572 | -12.50936572 | -0.011300435 | -0.024956242 |
| MKI73 | ENST00000368660 | -297.5402616 | 79.64325784 | -12.52920525 | -12.52920525 | -1.13E-02 | -2.50E-02 |
| SMC10 | NM_2380356 | -298.0629871 | 79.6966419 | -12.54904478 | -12.54904478 | -1.13E-02 | -2.50E-02 |
| STMN7 | NM_2578261 | -298.5857126 | 79.75002596 | -12.56888431 | -12.56888431 | -1.14E-02 | -0.02507861 |
| RPL41 | CR456883 | -299.1084381 | 79.80341002 | -12.58872385 | -12.58872385 | -0.011370392 | -0.025119399 |
| PRC7 | ENST00000590028 | -299.6311636 | 79.85679408 | -12.60856338 | -12.60856338 | -1.14E-02 | -0.025160189 |
| KIAA0107 | ENST00000609098 | -300.1538892 | 79.91017814 | -12.62840291 | -12.62840291 | -1.14E-02 | -0.025200978 |
| RCN7 | NM_030393 | -300.6766147 | 79.9635622 | -12.64824244 | -12.64824244 | -0.011422859 | -0.025241767 |
| CDKN9 | NM_032684 | -301.1993402 | 80.01694626 | -12.66808198 | -12.66808198 | -1.14E-02 | -0.025282556 |
| ARL6IP7 | ENST00000304420 | -301.7220657 | 80.07033032 | -12.68792151 | -12.68792151 | -0.011457837 | -0.025323346 |
| GJB12 | NM_001110219 | -302.2447912 | 80.12371437 | -12.70776104 | -12.70776104 | -0.011475326 | -0.025364135 |
| COL1A8 | NM_000089 | -302.7675167 | 80.17709843 | -12.72760057 | -12.72760057 | -0.011492815 | -0.025404924 |
| UCHL7 | NM_004181 | -303.2902422 | 80.23048249 | -12.74744011 | -12.74744011 | -0.011510304 | -0.025445714 |
| VCAN | NM_004385 | -303.8129677 | 80.28386655 | -12.76727964 | -12.76727964 | -0.011527794 | -0.025486503 |
| THY7 | ENST00000284246 | -304.3356932 | 80.33725061 | -12.78711917 | -12.78711917 | -0.011545283 | -0.025527292 |
| COL12A7 | NM_004376 | -304.8584187 | 80.39063467 | -12.8069587 | -12.8069587 | -0.011562772 | -0.025568081 |
| H2AFZ | ENST00000296423 | -305.3811442 | 80.44401873 | -12.82679824 | -12.82679824 | -1.16E-02 | -0.025608871 |
| FXR7 | NM_001013439 | -305.9038697 | 80.49740279 | -12.84663777 | -12.84663777 | -1.16E-02 | -0.02564966 |
| COL6A9 | NM_004369 | -306.4265952 | 80.55078685 | -12.8664773 | -12.8664773 | -0.011615239 | -0.025690449 |
| CENPF | NM_016343 | -306.9493208 | 80.60417091 | -12.88631683 | -12.88631683 | -1.16E-02 | -0.025731239 |
| TBL1XR7 | ENST00000430075 | -307.4720463 | 80.65755497 | -12.90615637 | -12.90615637 | -1.17E-02 | -0.025772028 |
| DSC8 | NM_024422 | -307.9947718 | 80.71093903 | -12.9259959 | -12.9259959 | -1.17E-02 | -2.58E-02 |
| CTHRC7 | NM_138455 | -308.5174973 | 80.76432309 | -12.94583543 | -12.94583543 | -0.011685195 | -0.025853606 |
| GPI | NM_000175 | -309.0402228 | 80.81770715 | -12.96567496 | -12.96567496 | -1.17E-02 | -2.59E-02 |
| PIGX | NM_001166304 | -309.5629483 | 80.87109121 | -12.9855145 | -12.9855145 | -0.011720173 | -0.025935185 |
| TUBB | ENST00000421479 | -310.0856738 | 80.92447527 | -13.00535403 | -13.00535403 | -1.17E-02 | -0.025975974 |
| DSG9 | NM_001944 | -310.6083993 | 80.97785933 | -13.02519356 | -13.02519356 | -0.011755152 | -0.026016763 |
| PGK7 | NM_000291 | -311.1311248 | 81.03124338 | -13.0450331 | -13.0450331 | -1.18E-02 | -2.61E-02 |
| ACTL6A | NM_178042 | -311.6538503 | 81.08462744 | -13.06487263 | -13.06487263 | -1.18E-02 | -0.026098342 |
| DSG8 | NM_001943 | -312.1765758 | 81.1380115 | -13.08471216 | -13.08471216 | -0.011807619 | -0.026139131 |
| ANLN | NM_018685 | -312.6993013 | 81.19139556 | -13.10455169 | -13.10455169 | -1.18E-02 | -0.026179921 |
| CALU | NM_001199671 | -313.2220268 | 81.24477962 | -13.12439123 | -13.12439123 | -1.18E-02 | -0.02622071 |
| HIST1H4C | NM_003542 | -313.7447524 | 81.29816368 | -13.14423076 | -13.14423076 | -1.19E-02 | -0.026261499 |
| UBE2T | ENST00001289158 | -314.2674779 | 81.35154774 | -13.16407029 | -13.16407029 | -1.19E-02 | -0.026302288 |
| MRPL57 | ENST00001427194 | -314.7902034 | 81.4049318 | -13.18390982 | -13.18390982 | -0.011895064 | -0.026343078 |
| PLOD8 | NM_411651 | -315.3129289 | 81.45831586 | -13.20374936 | -13.20374936 | -1.19E-02 | -0.026383867 |
| FOXM7 | NM_430710 | -315.8356544 | 81.51169992 | -13.22358889 | -13.22358889 | -1.19E-02 | -0.026424656 |
| ODC7 | ENST00002393943 | -316.3583799 | 81.56508398 | -13.24342842 | -13.24342842 | -0.011947532 | -0.026465446 |
| NDRG7 | ENST00002573929 | -316.8811054 | 81.61846804 | -13.26326795 | -13.26326795 | -0.011965021 | -0.026506235 |
| HIST1H2BM | NM_003527 | -317.4038309 | 81.6718521 | -13.28310749 | -13.28310749 | -0.01198251 | -0.026547024 |
| DTL | ENST00000366997 | -317.9265564 | 81.72523616 | -13.30294702 | -13.30294702 | -1.20E-02 | -2.66E-02 |
| SLC44A7 | NM_080546 | -318.4492819 | 81.77862022 | -13.32278655 | -13.32278655 | -1.20E-02 | -0.026628603 |
| GINS7 | NM_021067 | -318.9720074 | 81.83200428 | -13.34262608 | -13.34262608 | -1.20E-02 | -2.67E-02 |
| PRR17 | NM_018304 | -319.4947329 | 81.88538834 | -13.36246562 | -13.36246562 | -1.21E-02 | -0.026710181 |
| DCUN1D7 | ENST00002586714 | -320.0174584 | 81.93877239 | -13.38230515 | -13.38230515 | -0.012069955 | -0.02675097 |
| RBP7 | ENST00002777875 | -320.5401839 | 81.99215645 | -13.40214468 | -13.40214468 | -0.012087444 | -0.02679176 |
| NUSAP7 | NM_016359 | -321.0629095 | 82.04554051 | -13.42198421 | -13.42198421 | -1.21E-02 | -2.68E-02 |
| HIST1H3I | NM_003533 | -321.585635 | 82.09892457 | -13.44182375 | -13.44182375 | -0.012122422 | -0.026873338 |
| ECT8 | NM_001258316 | -322.1083605 | 82.15230863 | -13.46166328 | -13.46166328 | -0.012139912 | -0.026914128 |
| FSCN7 | NM_003088 | -322.631086 | 82.20569269 | -13.48150281 | -13.48150281 | -0.012157401 | -0.026954917 |
| DNAJB17 | ENST00000439351 | -323.1538115 | 82.25907675 | -13.50134234 | -13.50134234 | -1.22E-02 | -0.026995706 |
| CDC26 | ENST00000372462 | -323.676537 | 82.31246081 | -13.52118188 | -13.52118188 | -0.012192379 | -0.027036495 |
| ARHGAP11A | ENST00000361627 | -324.1992625 | 82.36584487 | -13.54102141 | -13.54102141 | -1.22E-02 | -2.71E-02 |
| CCT11 | ENST00000280326 | -324.721988 | 82.41922893 | -13.56086094 | -13.56086094 | -0.012227357 | -0.027118074 |
| NTRK8 | NM_006180 | -325.2447135 | 82.47261299 | -13.58070047 | -13.58070047 | -0.012244846 | -0.027158863 |
| ENO7 | NM_001428 | -325.767439 | 82.52599705 | -13.60054001 | -13.60054001 | -1.23E-02 | -0.027199653 |
| HIST1H2AE | NM_021052 | -326.2901645 | 82.57938111 | -13.62037954 | -13.62037954 | -0.012279824 | -0.027240442 |
| THBS8 | NM_003247 | -326.81289 | 82.63276517 | -13.64021907 | -13.64021907 | -0.012297313 | -0.027281231 |
| PA2G10 | NM_006191 | -327.3356155 | 82.68614923 | -13.6600586 | -13.6600586 | -1.23E-02 | -0.02732202 |
| CKAP8 | NM_018204 | -327.8583411 | 82.73953329 | -13.67989814 | -13.67989814 | -1.23E-02 | -0.02736281 |
| MMP18 | ENST00003344669 | -328.3810666 | 82.79291735 | -13.69973767 | -13.69973767 | -1.23E-02 | -0.027403599 |
| CDC12 | ENST00003667796 | -328.9037921 | 82.8463014 | -13.7195772 | -13.7195772 | -1.24E-02 | -2.74E-02 |
| HMGCS7 | NM_012042184 | -329.4265176 | 82.89968546 | -13.73941673 | -13.73941673 | -0.012384759 | -0.027485177 |
| TFRC | NM_013137222 | -329.9492431 | 82.95306952 | -13.75925627 | -13.75925627 | -0.012402248 | -0.027525967 |
| GOLIM10 | ENST00000470493 | -330.4719686 | 83.00645358 | -13.7790958 | -13.7790958 | -1.24E-02 | -0.027566756 |
| YEATS8 | NM_014283299 | -330.9946941 | 83.05983764 | -13.79893533 | -13.79893533 | -1.24E-02 | -0.027607545 |
| PKM | NM_015472072 | -331.5174196 | 83.1132217 | -13.81877486 | -13.81877486 | -1.25E-02 | -0.027648335 |
| RPL39L | ENST00000296277 | -332.0401451 | 83.16660576 | -13.8386144 | -13.8386144 | -0.012472204 | -0.027689124 |
| FKBP10 | ENST00000001008 | -332.5628706 | 83.21998982 | -13.85845393 | -13.85845393 | -0.012489693 | -0.027729913 |
| C8orf65 | ENST00000518786 | -333.0855961 | 83.27337388 | -13.87829346 | -13.87829346 | -0.012507182 | -0.027770702 |
| ASPM | NM_018142 | -333.6083216 | 83.32675794 | -13.89813299 | -13.89813299 | -1.25E-02 | -2.78E-02 |
| MTHFD8 | NR_027411 | -334.1310471 | 83.380142 | -13.91797253 | -13.91797253 | -0.012542161 | -0.027852281 |
| ITGA12 | NM_000210 | -334.6537727 | 83.43352606 | -13.93781206 | -13.93781206 | -0.01255965 | -0.02789307 |
| HIST1H4L | NM_003546 | -335.1764982 | 83.48691012 | -13.95765159 | -13.95765159 | -0.012577139 | -0.02793386 |
| DLGAP11 | NM_001146015 | -335.6992237 | 83.54029418 | -13.97749113 | -13.97749113 | -1.26E-02 | -0.027974649 |
| ADAM29 | ENST00000264383 | -336.2219492 | 83.59367824 | -13.99733066 | -13.99733066 | -0.012612117 | -0.028015438 |
| ANAPC19 | NM_012698998 | -336.7446747 | 83.6470623 | -14.01717019 | -14.01717019 | -0.012629606 | -0.028056227 |
| HIST1H2BK | NM_013860779 | -337.2674002 | 83.70044636 | -14.03700972 | -14.03700972 | -0.012647095 | -0.028097017 |
| DLX11 | ENST00000222604 | -337.7901257 | 83.75383041 | -14.05684926 | -14.05684926 | -0.012664584 | -0.028137806 |
| ZNF137 | NM_174408 | -338.3128512 | 83.80721447 | -14.07668879 | -14.07668879 | -1.27E-02 | -0.028178595 |
| ASPN | NM_188656 | -338.8355767 | 83.86059853 | -14.09652832 | -14.09652832 | -0.012699562 | -0.028219384 |
| FADS8 | ENST00000278846 | -339.3583022 | 83.91398259 | -14.11636785 | -14.11636785 | -0.012717051 | -0.028260174 |
| DEPDC7 | NM_001114120 | -339.8810277 | 83.96736665 | -14.13620739 | -14.13620739 | -1.27E-02 | -0.028300963 |
| ITGAV | NM_002210 | -340.4037532 | 84.02075071 | -14.15604692 | -14.15604692 | -0.01275203 | -0.028341752 |
| HPRT7 | NM_000194 | -340.9264787 | 84.07413477 | -14.17588645 | -14.17588645 | -0.012769519 | -0.028382542 |
| IGF2BP9 | NM_006547 | -341.4492043 | 84.12751883 | -14.19572598 | -14.19572598 | -0.012787008 | -0.028423331 |
| PDK7 | NM_002610 | -341.9719298 | 84.18090289 | -14.21556552 | -14.21556552 | -1.28E-02 | -0.02846412 |
| GATC | NM_176818 | -342.4946553 | 84.23428695 | -14.23540505 | -14.23540505 | -0.012821986 | -0.028504909 |
| ILF8 | NM_004515 | -343.0173808 | 84.28767101 | -14.25524458 | -14.25524458 | -1.28E-02 | -2.85E-02 |
| MSANTD9 | NM_001198807 | -343.5401063 | 84.34105507 | -14.27508411 | -14.27508411 | -0.012856964 | -0.028586488 |
| NCAPD8 | ENST00000315585 | -344.0628318 | 84.39443913 | -14.29492365 | -14.29492365 | -1.29E-02 | -0.028627277 |
| SRPRB | NM_021209 | -344.5855573 | 84.44782319 | -14.31476318 | -14.31476318 | -1.29E-02 | -0.028668067 |
| MLLT17 | ENST00000368927 | -345.1082828 | 84.50120725 | -14.33460271 | -14.33460271 | -1.29E-02 | -0.028708856 |
| BGN | NM_001717 | -345.6310083 | 84.55459131 | -14.35444224 | -14.35444224 | -0.012926921 | -0.028749645 |
| RPF8 | ENST00000441454 | -346.1537338 | 84.60797537 | -14.37428178 | -14.37428178 | -0.01294441 | -0.028790434 |
| TARS | NR_047682 | -346.6764593 | 84.66135943 | -14.39412131 | -14.39412131 | -1.30E-02 | -0.028831224 |
| PSMD110 | ENST00000310118 | -347.1991848 | 84.71474348 | -14.41396084 | -14.41396084 | -1.30E-02 | -0.028872013 |
| PSMD119 | ENST00000261712 | -347.7219103 | 84.76812754 | -14.43380037 | -14.43380037 | -1.30E-02 | -0.028912802 |
| NETO8 | ENST00000562435 | -348.2446359 | 84.8215116 | -14.45363991 | -14.45363991 | -0.013014366 | -0.028953592 |
| NMD9 | ENST00000351193 | -348.7673614 | 84.87489566 | -14.47347944 | -14.47347944 | -1.30E-02 | -0.028994381 |
| SERPINH7 | ENST00000524558 | -349.2900869 | 84.92827972 | -14.49331897 | -14.49331897 | -0.013049344 | -0.02903517 |
| CEP61 | ENST00000371485 | -349.8128124 | 84.98166378 | -14.5131585 | -14.5131585 | -1.31E-02 | -0.029075959 |
| BUB1B | ENST00000287598 | -350.3355379 | 85.03504784 | -14.53299804 | -14.53299804 | -1.31E-02 | -0.029116749 |
| PLK7 | ENST00000300093 | -350.8582634 | 85.0884319 | -14.55283757 | -14.55283757 | -1.31E-02 | -0.029157538 |
| EIF4A8 | NM_001967 | -351.3809889 | 85.14181596 | -14.5726771 | -14.5726771 | -1.31E-02 | -0.029198327 |
| GGCT | NM_024051 | -351.9037144 | 85.19520002 | -14.59251663 | -14.59251663 | -1.31E-02 | -0.029239116 |
| KDELR8 | NM_006854 | -352.4264399 | 85.24858408 | -14.61235617 | -14.61235617 | -0.013154279 | -0.029279906 |
| FNDC3B | NM_022763 | -352.9491654 | 85.30196814 | -14.6321957 | -14.6321957 | -1.32E-02 | -0.029320695 |
| PLS9 | NM_005032 | -353.4718909 | 85.3553522 | -14.65203523 | -14.65203523 | -0.013189257 | -0.029361484 |
| NDC86 | NM_006101 | -353.9946164 | 85.40873626 | -14.67187476 | -14.67187476 | -1.32E-02 | -0.029402274 |
| SHFM7 | ENST00000482395 | -354.5173419 | 85.46212032 | -14.6917143 | -14.6917143 | -0.013224235 | -0.029443063 |
| CCNB7 | NM_031966 | -355.0400675 | 85.51550438 | -14.71155383 | -14.71155383 | -1.32E-02 | -0.029483852 |
| SLC7A11 | NM_003486 | -355.562793 | 85.56888844 | -14.73139336 | -14.73139336 | -1.33E-02 | -2.95E-02 |
| FZD13 | NM_003507 | -356.0855185 | 85.62227249 | -14.75123289 | -14.75123289 | -1.33E-02 | -0.029565431 |
| MRPL9 | NM_007208 | -356.608244 | 85.67565655 | -14.77107243 | -14.77107243 | -0.013294191 | -0.02960622 |
| CSE1L | NM_001316 | -357.1309695 | 85.72904061 | -14.79091196 | -14.79091196 | -0.01331168 | -0.029647009 |
| HIF1A | NM_001530 | -357.653695 | 85.78242467 | -14.81075149 | -14.81075149 | -0.01332917 | -0.029687799 |
| SPP8 | NM_001251830 | -358.1764205 | 85.83580873 | -14.83059102 | -14.83059102 | -1.33E-02 | -2.97E-02 |
| SPRR2A | NM_005988 | -358.699146 | 85.88919279 | -14.85043056 | -14.85043056 | -0.013364148 | -0.029769377 |
| SCD | NM_005063 | -359.2218715 | 85.94257685 | -14.87027009 | -14.87027009 | -1.34E-02 | -0.029810166 |
| HIST1H3C | NM_003531 | -359.744597 | 85.99596091 | -14.89010962 | -14.89010962 | -0.013399126 | -0.029850956 |
| RRM9 | ENST00000279632 | -360.2673225 | 86.04934497 | -14.90994916 | -14.90994916 | -1.34E-02 | -2.99E-02 |
| FN8 | ENST00000273851 | -360.790048 | 86.10272903 | -14.92978869 | -14.92978869 | -0.013434104 | -0.029932534 |
| HIST2H2AB | NM_175065 | -361.3127735 | 86.15611309 | -14.94962822 | -14.94962822 | -1.35E-02 | -3.00E-02 |
| HIST1H3F | NM_021018 | -361.835499 | 86.20949715 | -14.96946775 | -14.96946775 | -1.35E-02 | -0.030014113 |
| GPNMB | NM_001005340 | -362.3582246 | 86.26288121 | -14.98930729 | -14.98930729 | -1.35E-02 | -0.030054902 |
| TOP2A | NM_001067 | -362.8809501 | 86.31626527 | -15.00914682 | -15.00914682 | -1.35E-02 | -0.030095691 |
| COL1A8 | ENST00001213594 | -363.4036756 | 86.36964933 | -15.02898635 | -15.02898635 | -0.01352155 | -0.030136481 |
| MAD2L8 | ENST00001284139 | -363.9264011 | 86.42303339 | -15.04882588 | -15.04882588 | -0.013539039 | -0.03017727 |
| HIST1H4B | NM_020494 | -364.4491266 | 86.47641745 | -15.06866542 | -15.06866542 | -1.36E-02 | -3.02E-02 |
| CKS9 | NM_022211 | -364.9718521 | 86.5298015 | -15.08850495 | -15.08850495 | -0.013574017 | -0.030258848 |
| GREM8 | ENST00000300177 | -365.4945776 | 86.58318556 | -15.10834448 | -15.10834448 | -0.013591506 | -0.030299638 |
| PTTG8 | ENST00000352433 | -366.0173031 | 86.63656962 | -15.12818401 | -15.12818401 | -1.36E-02 | -0.030340427 |
| LUM | ENST00000266718 | -366.5400286 | 86.68995368 | -15.14802355 | -15.14802355 | -0.013626484 | -0.030381216 |
| SPARC | NM_003118 | -367.0627541 | 86.74333774 | -15.16786308 | -15.16786308 | -0.013643973 | -0.030422006 |
| GJA8 | NM_000165 | -367.5854796 | 86.7967218 | -15.18770261 | -15.18770261 | -0.013661462 | -0.030462795 |
| TPX9 | NM_012112 | -368.1082051 | 86.85010586 | -15.20754214 | -15.20754214 | -1.37E-02 | -0.030503584 |
| COL3A8 | ENST00000304643 | -368.6309306 | 86.90348992 | -15.22738168 | -15.22738168 | -0.01369644 | -0.030544373 |
| SLC16A8 | NM_000630422 | -369.1536562 | 86.95687398 | -15.24722121 | -15.24722121 | -0.013713929 | -0.030585163 |
| SULF8 | NM_000592131 | -369.6763817 | 87.01025804 | -15.26706074 | -15.26706074 | -0.013731419 | -0.030625952 |
| MKI74 | ENST00000368661 | -370.1991072 | 87.0636421 | -15.28690027 | -15.28690027 | -1.37E-02 | -3.07E-02 |
| SMC11 | NM_2776166 | -370.7218327 | 87.11702616 | -15.30673981 | -15.30673981 | -1.38E-02 | -3.07E-02 |
| STMN8 | NM_2974071 | -371.2445582 | 87.17041022 | -15.32657934 | -15.32657934 | -1.38E-02 | -0.03074832 |
| RPL42 | CR456884 | -371.7672837 | 87.22379428 | -15.34641887 | -15.34641887 | -0.013801375 | -0.030789109 |
| PRC8 | ENST00000628168 | -372.2900092 | 87.27717834 | -15.3662584 | -15.3662584 | -1.38E-02 | -0.030829898 |
| KIAA0108 | ENST00000647238 | -372.8127347 | 87.3305624 | -15.38609794 | -15.38609794 | -1.38E-02 | -0.030870688 |
| RCN8 | NM_034975 | -373.3354602 | 87.38394646 | -15.40593747 | -15.40593747 | -0.013853842 | -0.030911477 |
| CDKN10 | NM_037266 | -373.8581857 | 87.43733051 | -15.425777 | -15.425777 | -1.39E-02 | -0.030952266 |
| ARL6IP8 | ENST00000304421 | -374.3809112 | 87.49071457 | -15.44561653 | -15.44561653 | -0.01388882 | -0.030993055 |
| GJB13 | NM_001110219 | -374.9036367 | 87.54409863 | -15.46545607 | -15.46545607 | -0.013906309 | -0.031033845 |
| COL1A9 | NM_000089 | -375.4263622 | 87.59748269 | -15.4852956 | -15.4852956 | -0.013923799 | -0.031074634 |
| UCHL8 | NM_004181 | -375.9490878 | 87.65086675 | -15.50513513 | -15.50513513 | -0.013941288 | -0.031115423 |
| VCAN | NM_004385 | -376.4718133 | 87.70425081 | -15.52497466 | -15.52497466 | -0.013958777 | -0.031156213 |
| THY8 | ENST00000284247 | -376.9945388 | 87.75763487 | -15.5448142 | -15.5448142 | -0.013976266 | -0.031197002 |
| COL12A8 | NM_004377 | -377.5172643 | 87.81101893 | -15.56465373 | -15.56465373 | -0.013993755 | -0.031237791 |
| H2AFZ | ENST00000296424 | -378.0399898 | 87.86440299 | -15.58449326 | -15.58449326 | -1.40E-02 | -0.03127858 |
| FXR8 | NM_001013439 | -378.5627153 | 87.91778705 | -15.60433279 | -15.60433279 | -1.40E-02 | -0.03131937 |
| COL6A10 | NM_004369 | -379.0854408 | 87.97117111 | -15.62417233 | -15.62417233 | -0.014046222 | -0.031360159 |
| CENPF | NM_016343 | -379.6081663 | 88.02455517 | -15.64401186 | -15.64401186 | -1.41E-02 | -0.031400948 |
| TBL1XR8 | ENST00000430076 | -380.1308918 | 88.07793923 | -15.66385139 | -15.66385139 | -1.41E-02 | -0.031441737 |
| DSC9 | NM_024422 | -380.6536173 | 88.13132329 | -15.68369092 | -15.68369092 | -1.41E-02 | -3.15E-02 |
| CTHRC8 | NM_138455 | -381.1763428 | 88.18470735 | -15.70353046 | -15.70353046 | -0.014116179 | -0.031523316 |
| GPI | NM_000175 | -381.6990683 | 88.23809141 | -15.72336999 | -15.72336999 | -1.41E-02 | -3.16E-02 |
| PIGX | NM_001166304 | -382.2217938 | 88.29147547 | -15.74320952 | -15.74320952 | -0.014151157 | -0.031604895 |
| TUBB | ENST00000421480 | -382.7445194 | 88.34485952 | -15.76304905 | -15.76304905 | -1.42E-02 | -0.031645684 |
| DSG10 | NM_001944 | -383.2672449 | 88.39824358 | -15.78288859 | -15.78288859 | -0.014186135 | -0.031686473 |
| PGK8 | NM_000291 | -383.7899704 | 88.45162764 | -15.80272812 | -15.80272812 | -1.42E-02 | -3.17E-02 |
| ACTL6A | NM_178042 | -384.3126959 | 88.5050117 | -15.82256765 | -15.82256765 | -1.42E-02 | -0.031768052 |
| DSG9 | NM_001943 | -384.8354214 | 88.55839576 | -15.84240719 | -15.84240719 | -0.014238602 | -0.031808841 |
| ANLN | NM_018685 | -385.3581469 | 88.61177982 | -15.86224672 | -15.86224672 | -1.43E-02 | -0.03184963 |
| CALU | NM_001199671 | -385.8808724 | 88.66516388 | -15.88208625 | -15.88208625 | -1.43E-02 | -0.03189042 |
| HIST1H4C | NM_003542 | -386.4035979 | 88.71854794 | -15.90192578 | -15.90192578 | -1.43E-02 | -0.031931209 |
| UBE2T | ENST00001565230 | -386.9263234 | 88.771932 | -15.92176532 | -15.92176532 | -1.43E-02 | -0.031971998 |
| MRPL58 | ENST00001703266 | -387.4490489 | 88.82531606 | -15.94160485 | -15.94160485 | -0.014326048 | -0.032012787 |
| PLOD9 | NM_449769 | -387.9717744 | 88.87870012 | -15.96144438 | -15.96144438 | -1.43E-02 | -0.032053577 |
| FOXM8 | NM_468828 | -388.4944999 | 88.93208418 | -15.98128391 | -15.98128391 | -1.44E-02 | -0.032094366 |
| ODC8 | ENST00002753915 | -389.0172254 | 88.98546824 | -16.00112345 | -16.00112345 | -0.014378515 | -0.032135155 |
| NDRG8 | ENST00002933901 | -389.539951 | 89.0388523 | -16.02096298 | -16.02096298 | -0.014396004 | -0.032175945 |
| HIST1H2BM | NM_003528 | -390.0626765 | 89.09223636 | -16.04080251 | -16.04080251 | -0.014413493 | -0.032216734 |
| DTL | ENST00000366998 | -390.585402 | 89.14562042 | -16.06064204 | -16.06064204 | -1.44E-02 | -3.23E-02 |
| SLC44A8 | NM_080546 | -391.1081275 | 89.19900448 | -16.08048158 | -16.08048158 | -1.44E-02 | -0.032298312 |
| GINS8 | NM_021067 | -391.630853 | 89.25238854 | -16.10032111 | -16.10032111 | -1.45E-02 | -3.23E-02 |
| PRR18 | NM_018304 | -392.1535785 | 89.30577259 | -16.12016064 | -16.12016064 | -1.45E-02 | -0.032379891 |
| DCUN1D8 | ENST00002969036 | -392.676304 | 89.35915665 | -16.14000017 | -16.14000017 | -0.014500938 | -0.03242068 |
| RBP8 | ENST00003160197 | -393.1990295 | 89.41254071 | -16.15983971 | -16.15983971 | -0.014518428 | -0.032461469 |
| NUSAP8 | NM_016359 | -393.721755 | 89.46592477 | -16.17967924 | -16.17967924 | -1.45E-02 | -3.25E-02 |
| HIST1H3I | NM_003533 | -394.2444805 | 89.51930883 | -16.19951877 | -16.19951877 | -0.014553406 | -0.032543048 |
| ECT9 | NM_001258316 | -394.767206 | 89.57269289 | -16.2193583 | -16.2193583 | -0.014570895 | -0.032583837 |
| FSCN8 | NM_003088 | -395.2899315 | 89.62607695 | -16.23919784 | -16.23919784 | -0.014588384 | -0.032624627 |
| DNAJB18 | ENST00000439351 | -395.812657 | 89.67946101 | -16.25903737 | -16.25903737 | -1.46E-02 | -0.032665416 |
| CDC27 | ENST00000372462 | -396.3353825 | 89.73284507 | -16.2788769 | -16.2788769 | -0.014623362 | -0.032706205 |
| ARHGAP11A | ENST00000361627 | -396.8581081 | 89.78622913 | -16.29871643 | -16.29871643 | -1.46E-02 | -3.27E-02 |
| CCT12 | ENST00000280326 | -397.3808336 | 89.83961319 | -16.31855597 | -16.31855597 | -0.01465834 | -0.032787784 |
| NTRK9 | NM_006180 | -397.9035591 | 89.89299725 | -16.3383955 | -16.3383955 | -0.014675829 | -0.032828573 |
| ENO8 | NM_001428 | -398.4262846 | 89.94638131 | -16.35823503 | -16.35823503 | -1.47E-02 | -0.032869362 |
| HIST1H2AE | NM_021052 | -398.9490101 | 89.99976537 | -16.37807456 | -16.37807456 | -0.014710808 | -0.032910152 |
| THBS9 | NM_003247 | -399.4717356 | 90.05314943 | -16.3979141 | -16.3979141 | -0.014728297 | -0.032950941 |
| PA2G11 | NM_006191 | -399.9944611 | 90.10653349 | -16.41775363 | -16.41775363 | -1.47E-02 | -0.03299173 |
| CKAP9 | NM_018204 | -400.5171866 | 90.15991755 | -16.43759316 | -16.43759316 | -1.48E-02 | -0.033032519 |
| MMP19 | ENST00003990923 | -401.0399121 | 90.2133016 | -16.45743269 | -16.45743269 | -1.48E-02 | -0.033073309 |
| CDC13 | ENST00004314050 | -401.5626376 | 90.26668566 | -16.47727223 | -16.47727223 | -1.48E-02 | -3.31E-02 |
| HMGCS8 | NM_014232260 | -402.0853631 | 90.32006972 | -16.49711176 | -16.49711176 | -0.014815742 | -0.033154887 |
| TFRC | NM_015327298 | -402.6080886 | 90.37345378 | -16.51695129 | -16.51695129 | -0.014833231 | -0.033195676 |
| GOLIM11 | ENST00000470494 | -403.1308141 | 90.42683784 | -16.53679082 | -16.53679082 | -1.49E-02 | -0.033236466 |
| YEATS9 | NM_016660845 | -403.6535397 | 90.4802219 | -16.55663036 | -16.55663036 | -1.49E-02 | -0.033277255 |
| PKM | NM_017849618 | -404.1762652 | 90.53360596 | -16.57646989 | -16.57646989 | -1.49E-02 | -0.033318044 |
| RPL39L | ENST00000296277 | -404.6989907 | 90.58699002 | -16.59630942 | -16.59630942 | -0.014903187 | -0.033358834 |
| FKBP11 | ENST00000001008 | -405.2217162 | 90.64037408 | -16.61614895 | -16.61614895 | -0.014920677 | -0.033399623 |
| C8orf66 | ENST00000518786 | -405.7444417 | 90.69375814 | -16.63598849 | -16.63598849 | -0.014938166 | -0.033440412 |
| ASPM | NM_018143 | -406.2671672 | 90.7471422 | -16.65582802 | -16.65582802 | -1.50E-02 | -3.35E-02 |
| MTHFD9 | NR_027412 | -406.7898927 | 90.80052626 | -16.67566755 | -16.67566755 | -0.014973144 | -0.033521991 |
| ITGA13 | NM_000210 | -407.3126182 | 90.85391032 | -16.69550708 | -16.69550708 | -0.014990633 | -0.03356278 |
| HIST1H4L | NM_003546 | -407.8353437 | 90.90729438 | -16.71534662 | -16.71534662 | -0.015008122 | -0.033603569 |
| DLGAP12 | NM_001146015 | -408.3580692 | 90.96067844 | -16.73518615 | -16.73518615 | -1.50E-02 | -0.033644359 |
| ADAM30 | ENST00000264384 | -408.8807947 | 91.0140625 | -16.75502568 | -16.75502568 | -0.0150431 | -0.033685148 |
| ANAPC20 | NM_015022560 | -409.4035202 | 91.06744656 | -16.77486522 | -16.77486522 | -0.015060589 | -0.033725937 |
| HIST1H2BK | NM_016184341 | -409.9262457 | 91.12083061 | -16.79470475 | -16.79470475 | -0.015078078 | -0.033766726 |
| DLX12 | ENST00000222605 | -410.4489713 | 91.17421467 | -16.81454428 | -16.81454428 | -0.015095567 | -0.033807516 |
| ZNF138 | NM_202904 | -410.9716968 | 91.22759873 | -16.83438381 | -16.83438381 | -1.51E-02 | -0.033848305 |
| ASPN | NM_217152 | -411.4944223 | 91.28098279 | -16.85422335 | -16.85422335 | -0.015130546 | -0.033889094 |
| FADS9 | ENST00000278847 | -412.0171478 | 91.33436685 | -16.87406288 | -16.87406288 | -0.015148035 | -0.033929883 |
| DEPDC8 | NM_001114120 | -412.5398733 | 91.38775091 | -16.89390241 | -16.89390241 | -1.52E-02 | -0.033970673 |
| ITGAV | NM_002210 | -413.0625988 | 91.44113497 | -16.91374194 | -16.91374194 | -0.015183013 | -0.034011462 |
| HPRT8 | NM_000194 | -413.5853243 | 91.49451903 | -16.93358148 | -16.93358148 | -0.015200502 | -0.034052251 |
| IGF2BP10 | NM_006547 | -414.1080498 | 91.54790309 | -16.95342101 | -16.95342101 | -0.015217991 | -0.034093041 |
| PDK8 | NM_002610 | -414.6307753 | 91.60128715 | -16.97326054 | -16.97326054 | -1.52E-02 | -0.03413383 |
| GATC | NM_176818 | -415.1535008 | 91.65467121 | -16.99310007 | -16.99310007 | -0.015252969 | -0.034174619 |
| ILF9 | NM_004515 | -415.6762263 | 91.70805527 | -17.01293961 | -17.01293961 | -1.53E-02 | -3.42E-02 |
| MSANTD10 | NM_001198807 | -416.1989518 | 91.76143933 | -17.03277914 | -17.03277914 | -0.015287947 | -0.034256198 |
| NCAPD9 | ENST00000315586 | -416.7216773 | 91.81482339 | -17.05261867 | -17.05261867 | -1.53E-02 | -0.034296987 |
| SRPRB | NM_021210 | -417.2444029 | 91.86820745 | -17.0724582 | -17.0724582 | -1.53E-02 | -0.034337776 |
| MLLT18 | ENST00000368928 | -417.7671284 | 91.92159151 | -17.09229774 | -17.09229774 | -1.53E-02 | -0.034378566 |
| BGN | NM_001718 | -418.2898539 | 91.97497557 | -17.11213727 | -17.11213727 | -0.015357904 | -0.034419355 |
| RPF9 | ENST00000441455 | -418.8125794 | 92.02835962 | -17.1319768 | -17.1319768 | -0.015375393 | -0.034460144 |
| TARS | NR_047683 | -419.3353049 | 92.08174368 | -17.15181633 | -17.15181633 | -1.54E-02 | -0.034500933 |
| PSMD128 | ENST00000310118 | -419.8580304 | 92.13512774 | -17.17165587 | -17.17165587 | -1.54E-02 | -0.034541723 |
| PSMD137 | ENST00000261712 | -420.3807559 | 92.1885118 | -17.1914954 | -17.1914954 | -1.54E-02 | -0.034582512 |
| NETO9 | ENST00000562435 | -420.9034814 | 92.24189586 | -17.21133493 | -17.21133493 | -0.015445349 | -0.034623301 |
| NMD10 | ENST00000351193 | -421.4262069 | 92.29527992 | -17.23117446 | -17.23117446 | -1.55E-02 | -0.03466409 |
| SERPINH8 | ENST00000524558 | -421.9489324 | 92.34866398 | -17.251014 | -17.251014 | -0.015480327 | -0.03470488 |
| CEP62 | ENST00000371485 | -422.4716579 | 92.40204804 | -17.27085353 | -17.27085353 | -1.55E-02 | -0.034745669 |
| BUB1B | ENST00000287598 | -422.9943834 | 92.4554321 | -17.29069306 | -17.29069306 | -1.55E-02 | -0.034786458 |
| PLK8 | ENST00000300093 | -423.5171089 | 92.50881616 | -17.31053259 | -17.31053259 | -1.55E-02 | -0.034827248 |
| EIF4A9 | NM_001967 | -424.0398345 | 92.56220022 | -17.33037213 | -17.33037213 | -1.56E-02 | -0.034868037 |
| GGCT | NM_024051 | -424.56256 | 92.61558428 | -17.35021166 | -17.35021166 | -1.56E-02 | -0.034908826 |
| KDELR9 | NM_006854 | -425.0852855 | 92.66896834 | -17.37005119 | -17.37005119 | -0.015585262 | -0.034949615 |
| FNDC3B | NM_022763 | -425.608011 | 92.7223524 | -17.38989072 | -17.38989072 | -1.56E-02 | -0.034990405 |
| PLS10 | NM_005032 | -426.1307365 | 92.77573646 | -17.40973026 | -17.40973026 | -0.01562024 | -0.035031194 |
| NDC87 | NM_006101 | -426.653462 | 92.82912052 | -17.42956979 | -17.42956979 | -1.56E-02 | -0.035071983 |
| SHFM8 | ENST00000482396 | -427.1761875 | 92.88250458 | -17.44940932 | -17.44940932 | -0.015655218 | -0.035112773 |
| CCNB8 | NM_031966 | -427.698913 | 92.93588863 | -17.46924885 | -17.46924885 | -1.57E-02 | -0.035153562 |
| SLC7A12 | NM_003486 | -428.2216385 | 92.98927269 | -17.48908839 | -17.48908839 | -1.57E-02 | -3.52E-02 |
| FZD14 | NM_003507 | -428.744364 | 93.04265675 | -17.50892792 | -17.50892792 | -1.57E-02 | -0.03523514 |
| MRPL10 | NM_007208 | -429.2670895 | 93.09604081 | -17.52876745 | -17.52876745 | -0.015725175 | -0.03527593 |
| CSE1L | NM_001316 | -429.789815 | 93.14942487 | -17.54860698 | -17.54860698 | -0.015742664 | -0.035316719 |
| HIF1A | NM_001530 | -430.3125405 | 93.20280893 | -17.56844652 | -17.56844652 | -0.015760153 | -0.035357508 |
| SPP9 | NM_001251830 | -430.835266 | 93.25619299 | -17.58828605 | -17.58828605 | -1.58E-02 | -3.54E-02 |
| SPRR2A | NM_005988 | -431.3579916 | 93.30957705 | -17.60812558 | -17.60812558 | -0.015795131 | -0.035439087 |
| SCD | NM_005063 | -431.8807171 | 93.36296111 | -17.62796511 | -17.62796511 | -1.58E-02 | -0.035479876 |
| HIST1H3C | NM_003531 | -432.4034426 | 93.41634517 | -17.64780465 | -17.64780465 | -0.015830109 | -0.035520665 |
| RRM10 | ENST00000268070 | -432.9261681 | 93.46972923 | -17.66764418 | -17.66764418 | -1.58E-02 | -3.56E-02 |
| FN9 | ENST00000262289 | -433.4488936 | 93.52311329 | -17.68748371 | -17.68748371 | -0.015865087 | -0.035602244 |
| HIST2H2AB | NM_175065 | -433.9716191 | 93.57649735 | -17.70732325 | -17.70732325 | -1.59E-02 | -3.56E-02 |
| HIST1H3F | NM_021018 | -434.4943446 | 93.62988141 | -17.72716278 | -17.72716278 | -1.59E-02 | -0.035683822 |
| GPNMB | NM_001005340 | -435.0170701 | 93.68326547 | -17.74700231 | -17.74700231 | -1.59E-02 | -0.035724612 |
| TOP2A | NM_001067 | -435.5397956 | 93.73664953 | -17.76684184 | -17.76684184 | -1.59E-02 | -0.035765401 |
| COL1A9 | ENST00001354684 | -436.0625211 | 93.79003359 | -17.78668138 | -17.78668138 | -0.015952533 | -0.03580619 |
| MAD2L9 | ENST00001425229 | -436.5852466 | 93.84341765 | -17.80652091 | -17.80652091 | -0.015970022 | -0.03584698 |
| HIST1H4B | NM_023928 | -437.1079721 | 93.8968017 | -17.82636044 | -17.82636044 | -1.60E-02 | -3.59E-02 |
| CKS10 | NM_025645 | -437.6306976 | 93.95018576 | -17.84619997 | -17.84619997 | -0.016005 | -0.035928558 |
| GREM9 | ENST00000300177 | -438.1534232 | 94.00356982 | -17.86603951 | -17.86603951 | -0.016022489 | -0.035969347 |
| PTTG9 | ENST00000352433 | -438.6761487 | 94.05695388 | -17.88587904 | -17.88587904 | -1.60E-02 | -0.036010137 |
| LUM | ENST00000266718 | -439.1988742 | 94.11033794 | -17.90571857 | -17.90571857 | -0.016057467 | -0.036050926 |
| SPARC | NM_003118 | -439.7215997 | 94.163722 | -17.9255581 | -17.9255581 | -0.016074956 | -0.036091715 |
| GJA9 | NM_000165 | -440.2443252 | 94.21710606 | -17.94539764 | -17.94539764 | -0.016092445 | -0.036132505 |
| TPX10 | NM_012112 | -440.7670507 | 94.27049012 | -17.96523717 | -17.96523717 | -1.61E-02 | -0.036173294 |
| COL3A9 | ENST00000304644 | -441.2897762 | 94.32387418 | -17.9850767 | -17.9850767 | -0.016127424 | -0.036214083 |
| SLC16A9 | NM_000553840 | -441.8125017 | 94.37725824 | -18.00491623 | -18.00491623 | -0.016144913 | -0.036254872 |
| SULF9 | NM_000515549 | -442.3352272 | 94.4306423 | -18.02475577 | -18.02475577 | -0.016162402 | -0.036295662 |
| MKI75 | ENST00000368662 | -442.8579527 | 94.48402636 | -18.0445953 | -18.0445953 | -1.62E-02 | -3.63E-02 |
| SMC12 | NM_3171976 | -443.3806782 | 94.53741042 | -18.06443483 | -18.06443483 | -1.62E-02 | -3.64E-02 |
| STMN9 | NM_3369881 | -443.9034037 | 94.59079448 | -18.08427436 | -18.08427436 | -1.62E-02 | -0.036418029 |
| RPL43 | CR456885 | -444.4261292 | 94.64417854 | -18.1041139 | -18.1041139 | -0.016232358 | -0.036458819 |
| PRC9 | ENST00000666308 | -444.9488548 | 94.6975626 | -18.12395343 | -18.12395343 | -1.62E-02 | -0.036499608 |
| KIAA0109 | ENST00000685378 | -445.4715803 | 94.75094666 | -18.14379296 | -18.14379296 | -1.63E-02 | -0.036540397 |
| RCN9 | NM_039557 | -445.9943058 | 94.80433071 | -18.16363249 | -18.16363249 | -0.016284825 | -0.036581187 |
| CDKN11 | NM_041848 | -446.5170313 | 94.85771477 | -18.18347203 | -18.18347203 | -1.63E-02 | -0.036621976 |
| ARL6IP9 | ENST00000304422 | -447.0397568 | 94.91109883 | -18.20331156 | -18.20331156 | -0.016319804 | -0.036662765 |
| GJB14 | NM_001110219 | -447.5624823 | 94.96448289 | -18.22315109 | -18.22315109 | -0.016337293 | -0.036703554 |
| COL1A10 | NM_000089 | -448.0852078 | 95.01786695 | -18.24299062 | -18.24299062 | -0.016354782 | -0.036744344 |
| UCHL9 | NM_004181 | -448.6079333 | 95.07125101 | -18.26283016 | -18.26283016 | -0.016372271 | -0.036785133 |
| VCAN | NM_004385 | -449.1306588 | 95.12463507 | -18.28266969 | -18.28266969 | -0.01638976 | -0.036825922 |
| THY9 | ENST00000284248 | -449.6533843 | 95.17801913 | -18.30250922 | -18.30250922 | -0.016407249 | -0.036866712 |
| COL12A9 | NM_004378 | -450.1761098 | 95.23140319 | -18.32234875 | -18.32234875 | -0.016424738 | -0.036907501 |
| H2AFZ | ENST00000296425 | -450.6988353 | 95.28478725 | -18.34218829 | -18.34218829 | -1.64E-02 | -0.03694829 |
| FXR9 | NM_001013439 | -451.2215608 | 95.33817131 | -18.36202782 | -18.36202782 | -1.65E-02 | -0.036989079 |
| COL6A11 | NM_004369 | -451.7442864 | 95.39155537 | -18.38186735 | -18.38186735 | -0.016477205 | -0.037029869 |
| CENPF | NM_016343 | -452.2670119 | 95.44493943 | -18.40170688 | -18.40170688 | -1.65E-02 | -0.037070658 |
| TBL1XR9 | ENST00000430077 | -452.7897374 | 95.49832349 | -18.42154642 | -18.42154642 | -1.65E-02 | -0.037111447 |
| DSC10 | NM_024422 | -453.3124629 | 95.55170755 | -18.44138595 | -18.44138595 | -1.65E-02 | -3.72E-02 |
| CTHRC9 | NM_138455 | -453.8351884 | 95.60509161 | -18.46122548 | -18.46122548 | -0.016547162 | -0.037193026 |
| GPI | NM_000175 | -454.3579139 | 95.65847567 | -18.48106501 | -18.48106501 | -1.66E-02 | -3.72E-02 |
| PIGX | NM_001166304 | -454.8806394 | 95.71185972 | -18.50090455 | -18.50090455 | -0.01658214 | -0.037274604 |
| TUBB | ENST00000421481 | -455.4033649 | 95.76524378 | -18.52074408 | -18.52074408 | -1.66E-02 | -0.037315394 |
| DSG11 | NM_001944 | -455.9260904 | 95.81862784 | -18.54058361 | -18.54058361 | -0.016617118 | -0.037356183 |
| PGK9 | NM_000291 | -456.4488159 | 95.8720119 | -18.56042314 | -18.56042314 | -1.66E-02 | -3.74E-02 |
| ACTL6A | NM_178042 | -456.9715414 | 95.92539596 | -18.58026268 | -18.58026268 | -1.67E-02 | -0.037437761 |
| DSG10 | NM_001943 | -457.4942669 | 95.97878002 | -18.60010221 | -18.60010221 | -0.016669585 | -0.037478551 |
| ANLN | NM_018685 | -458.0169924 | 96.03216408 | -18.61994174 | -18.61994174 | -1.67E-02 | -0.03751934 |
| CALU | NM_001199671 | -458.539718 | 96.08554814 | -18.63978128 | -18.63978128 | -1.67E-02 | -0.037560129 |
| HIST1H4C | NM_003542 | -459.0624435 | 96.1389322 | -18.65962081 | -18.65962081 | -1.67E-02 | -0.037600919 |
| UBE2T | ENST00001841302 | -459.585169 | 96.19231626 | -18.67946034 | -18.67946034 | -1.67E-02 | -0.037641708 |
| MRPL59 | ENST00001979338 | -460.1078945 | 96.24570032 | -18.69929987 | -18.69929987 | -0.016757031 | -0.037682497 |
| PLOD10 | NM_487887 | -460.63062 | 96.29908438 | -18.71913941 | -18.71913941 | -1.68E-02 | -0.037723286 |
| FOXM9 | NM_506946 | -461.1533455 | 96.35246844 | -18.73897894 | -18.73897894 | -1.68E-02 | -0.037764076 |
| ODC9 | ENST00003113887 | -461.676071 | 96.4058525 | -18.75881847 | -18.75881847 | -0.016809498 | -0.037804865 |
| NDRG9 | ENST00003293873 | -462.1987965 | 96.45923656 | -18.778658 | -18.778658 | -0.016826987 | -0.037845654 |
| HIST1H2BM | NM_003529 | -462.721522 | 96.51262062 | -18.79849754 | -18.79849754 | -0.016844476 | -0.037886443 |
| DTL | ENST00000366999 | -463.2442475 | 96.56600468 | -18.81833707 | -18.81833707 | -1.69E-02 | -3.79E-02 |
| SLC44A9 | NM_080546 | -463.766973 | 96.61938873 | -18.8381766 | -18.8381766 | -1.69E-02 | -0.037968022 |
| GINS9 | NM_021067 | -464.2896985 | 96.67277279 | -18.85801613 | -18.85801613 | -1.69E-02 | -3.80E-02 |
| PRR19 | NM_018304 | -464.812424 | 96.72615685 | -18.87785567 | -18.87785567 | -1.69E-02 | -0.038049601 |
| DCUN1D9 | ENST00003351358 | -465.3351496 | 96.77954091 | -18.8976952 | -18.8976952 | -0.016931922 | -0.03809039 |
| RBP9 | ENST00003542519 | -465.8578751 | 96.83292497 | -18.91753473 | -18.91753473 | -0.016949411 | -0.038131179 |
| NUSAP9 | NM_016359 | -466.3806006 | 96.88630903 | -18.93737426 | -18.93737426 | -1.70E-02 | -3.82E-02 |
| HIST1H3I | NM_003533 | -466.9033261 | 96.93969309 | -18.9572138 | -18.9572138 | -0.016984389 | -0.038212758 |
| ECT10 | NM_001258316 | -467.4260516 | 96.99307715 | -18.97705333 | -18.97705333 | -0.017001878 | -0.038253547 |
| FSCN9 | NM_003088 | -467.9487771 | 97.04646121 | -18.99689286 | -18.99689286 | -0.017019367 | -0.038294336 |
| DNAJB19 | ENST00000439351 | -468.4715026 | 97.09984527 | -19.01673239 | -19.01673239 | -1.70E-02 | -0.038335126 |
| CDC28 | ENST00000372462 | -468.9942281 | 97.15322933 | -19.03657193 | -19.03657193 | -0.017054345 | -0.038375915 |
| ARHGAP11A | ENST00000361627 | -469.5169536 | 97.20661339 | -19.05641146 | -19.05641146 | -1.71E-02 | -3.84E-02 |
| CCT13 | ENST00000280326 | -470.0396791 | 97.25999745 | -19.07625099 | -19.07625099 | -0.017089323 | -0.038457493 |
| NTRK10 | NM_006180 | -470.5624046 | 97.31338151 | -19.09609052 | -19.09609052 | -0.017106813 | -0.038498283 |
| ENO9 | NM_001428 | -471.0851301 | 97.36676557 | -19.11593006 | -19.11593006 | -1.71E-02 | -0.038539072 |
| HIST1H2AE | NM_021052 | -471.6078556 | 97.42014963 | -19.13576959 | -19.13576959 | -0.017141791 | -0.038579861 |
| THBS10 | NM_003247 | -472.1305811 | 97.47353369 | -19.15560912 | -19.15560912 | -0.01715928 | -0.03862065 |
| PA2G12 | NM_006191 | -472.6533067 | 97.52691774 | -19.17544865 | -19.17544865 | -1.72E-02 | -0.03866144 |
| CKAP10 | NM_018204 | -473.1760322 | 97.5803018 | -19.19528819 | -19.19528819 | -1.72E-02 | -0.038702229 |
| MMP20 | ENST00004637177 | -473.6987577 | 97.63368586 | -19.21512772 | -19.21512772 | -1.72E-02 | -0.038743018 |
| CDC14 | ENST00004960304 | -474.2214832 | 97.68706992 | -19.23496725 | -19.23496725 | -1.72E-02 | -3.88E-02 |
| HMGCS9 | NM_016422336 | -474.7442087 | 97.74045398 | -19.25480678 | -19.25480678 | -0.017246725 | -0.038824597 |
| TFRC | NM_017517374 | -475.2669342 | 97.79383804 | -19.27464632 | -19.27464632 | -0.017264214 | -0.038865386 |
| GOLIM12 | ENST00000470495 | -475.7896597 | 97.8472221 | -19.29448585 | -19.29448585 | -1.73E-02 | -0.038906175 |
| YEATS10 | NM_019038391 | -476.3123852 | 97.90060616 | -19.31432538 | -19.31432538 | -1.73E-02 | -0.038946965 |

Additional file 4. Table S4. Differential RNA expression in PSCC versus paired controls.

| **Gene symbol** | **RefSeq** | **Mean ISCC** | **Mean control** | **Ratio** | **Fold change** | **P-value** | **Q-value** |
| --- | --- | --- | --- | --- | --- | --- | --- |
| ABI3BP | NM_015429 | 55.21659424 | 30.12898152 | 0.545654 | -1.83266 | 6.38E-06 | 0.01094802 |
| ACTA2 | NM_001141945 | 80.12663581 | 40.53341993 | 0.505867 | -1.9768 | 0.008852402 | 0.1112118 |
| ADAM23 | ENST00000264377 | 16.90361607 | 30.94318936 | 1.83056 | 1.83056 | 0.008953124 | 0.1116302 |
| AKD1 | ENST00000285397 | 20.10064652 | 10.30619579 | 0.512728 | -1.95035 | 0.000261232 | 0.02864118 |
| AKR1B10 | NM_020299 | 27.54636406 | 69.5563344 | 2.52506 | 2.52506 | 0.002006164 | 0.06093259 |
| AKR1C1 | ENST00000434459 | 87.67903409 | 191.8333842 | 2.18789 | 2.18789 | 0.001582425 | 0.05619455 |
| AKR1C2 | NM_001135241 | 83.37078417 | 222.6092914 | 2.67011 | 2.67011 | 0.000465163 | 0.03541919 |
| A2M | ENST00000318602 | 121.2759777 | 54.23566632 | 0.44721 | -2.23609 | 0.000723832 | 0.04076209 |
| APOBEC4 | NM_203454 | 32.29009992 | 14.29174329 | 0.442604 | -2.25936 | 0.000185249 | 0.026672 |
| APOD | NM_001647 | 186.6070911 | 74.40821474 | 0.398741 | -2.50789 | 0.000313816 | 0.03094845 |
| ABCC5 | NM_005688 | 42.72563415 | 82.35018581 | 1.92742 | 1.92742 | 0.004588493 | 0.08316321 |
| AGBL2 | NM_024783 | 27.64238131 | 12.66214573 | 0.45807 | -2.18307 | 0.000305026 | 0.03063523 |
| ATP1B3 | NM_001679 | 41.21592591 | 76.64660579 | 1.85964 | 1.85964 | 0.002164394 | 0.06314647 |
| BEST4 | NM_153274 | 29.61713123 | 15.28950717 | 0.516238 | -1.93709 | 2.52E-05 | 0.01535475 |
| BPIFA1 | NM_130852 | 139.5008401 | 36.42961896 | 0.261143 | -3.82932 | 0.00157352 | 0.05599979 |
| BPIFB1 | NM_033197 | 903.7688358 | 191.9650685 | 0.212405 | -4.70799 | 9.29E-05 | 0.02257817 |
| CDHR3 | NM_152750 | 81.1241752 | 25.55458044 | 0.315005 | -3.17455 | 8.62E-05 | 0.02239032 |
| CASC1 | NM_001082972 | 15.69246406 | 7.603955031 | 0.48456 | -2.06373 | 0.000619111 | 0.03913213 |
| CHST9 | NM_031422 | 48.72228872 | 25.1464005 | 0.516116 | -1.93755 | 0.000753278 | 0.04138195 |
| CKS2 | NM_001827 | 15.39702803 | 27.01034196 | 1.75426 | 1.75426 | 0.009440137 | 0.1143918 |
| CDV3 | ENST00000431519 | 66.62769135 | 117.3041302 | 1.76059 | 1.76059 | 0.001288407 | 0.05075373 |
| CDCA7L | NM_018719 | 20.82031513 | 10.97257328 | 0.527016 | -1.89748 | 0.000181243 | 0.026672 |
| CDR1 | NM_004065 | 174.9404379 | 77.15779448 | 0.441051 | -2.26731 | 0.00696009 | 0.0991925 |
| CCL15 | ENST00000495214 | 66.16837755 | 30.60382344 | 0.462514 | -2.1621 | 6.55E-05 | 0.02125486 |
| C1orf158 | NM_152290 | 44.38775998 | 22.93851354 | 0.516778 | -1.93507 | 0.000918178 | 0.04447154 |
| C1orf168 | NM_001004303 | 18.52138397 | 9.666341739 | 0.5219 | -1.91608 | 2.60E-06 | 0.008512856 |
| C1orf173 | ENST00000326665 | 25.46881622 | 13.34964988 | 0.524155 | -1.90783 | 1.77E-05 | 0.01295435 |
| C1orf87 | ENST00000371201 | 22.13396487 | 12.63698155 | 0.570932 | -1.75152 | 2.37E-05 | 0.01535475 |
| C1orf88 | ENST00000369738 | 37.21313424 | 14.38795852 | 0.386636 | -2.58641 | 0.000229349 | 0.02778307 |
| C11orf88 | ENST00000529167 | 27.58457791 | 15.66985582 | 0.568065 | -1.76036 | 0.001329922 | 0.05099559 |
| C12orf63 | ENST00000342887 | 17.39836704 | 9.171896023 | 0.527168 | -1.89693 | 0.000922645 | 0.04452118 |
| C2orf40 | ENST00000238044 | 27.15789959 | 13.83556831 | 0.509449 | -1.9629 | 1.98E-06 | 0.008103584 |
| C20orf85 | ENST00000371168 | 55.32578074 | 19.56888973 | 0.353704 | -2.82723 | 0.000165214 | 0.02616365 |
| C3orf15 | NM_033364 | 24.16504875 | 12.78978405 | 0.529269 | -1.8894 | 6.87E-05 | 0.02125486 |
| C6orf165 | ENST00000369562 | 14.91394478 | 8.460306909 | 0.567276 | -1.76281 | 0.000703385 | 0.04076209 |
| CLTC | NM_004859 | 45.38708725 | 86.95818961 | 1.91593 | 1.91593 | 0.000193823 | 0.0271099 |
| CLUAP1 | NM_015041 | 35.03900737 | 19.37279221 | 0.55289 | -1.80868 | 0.00103386 | 0.04661158 |
| CCDC108 | NM_194302 | 122.5715207 | 31.62623691 | 0.258024 | -3.87561 | 3.49E-07 | 0.005181383 |
| CCDC146 | NM_020879 | 42.00415094 | 22.25889393 | 0.529921 | -1.88708 | 0.001630172 | 0.05644258 |
| CCDC19 | ENST00000368099 | 42.4534531 | 23.15336535 | 0.545383 | -1.83357 | 0.00058107 | 0.03864177 |
| CCDC30 | ENST00000428554 | 20.6066774 | 10.72904677 | 0.52066 | -1.92064 | 0.000558759 | 0.03795591 |
| CTGF | ENST00000367976 | 103.9625417 | 47.29776149 | 0.45495 | -2.19805 | 2.72E-05 | 0.01535475 |
| CSTA | NM_005213 | 92.45375432 | 302.1015969 | 3.26761 | 3.26761 | 0.000791966 | 0.04236986 |
| CYR61 | NM_001554 | 68.70961052 | 37.39179955 | 0.544198 | -1.83757 | 0.000101511 | 0.02269291 |
| COX6B1 | NM_001863 | 72.48195683 | 133.6022042 | 1.84324 | 1.84324 | 0.02914321 | 0.1939123 |
| CYP2B7P1 | NR_001278 | 64.42103793 | 34.18638572 | 0.530673 | -1.8844 | 0.02657314 | 0.1856961 |
| DCN | NM_133506 | 94.33034245 | 51.72608152 | 0.548349 | -1.82366 | 0.008762106 | 0.1106816 |
| DLEC1 | ENST00000308059 | 48.26013346 | 25.21656745 | 0.522514 | -1.91382 | 0.000213091 | 0.02732415 |
| DSC2 | NM_024422 | 22.61111138 | 50.59524851 | 2.23762 | 2.23762 | 0.002209684 | 0.06335283 |
| DSC3 | NM_024423 | 31.09196732 | 73.86095443 | 2.37556 | 2.37556 | 0.000299851 | 0.03048959 |
| DSG3 | NM_001944 | 26.67710114 | 81.79722529 | 3.0662 | 3.0662 | 0.001773898 | 0.0579646 |
| DSP | ENST00000379802 | 80.2533661 | 168.4947698 | 2.09953 | 2.09953 | 0.000592272 | 0.03864177 |
| DLG1 | NM_004087 | 40.65018464 | 72.94615346 | 1.79449 | 1.79449 | 0.003819338 | 0.07721593 |
| DCDC1 | NM_181807 | 26.64291443 | 14.29471548 | 0.536531 | -1.86383 | 0.000334978 | 0.03188305 |
| DPY19L2P2 | ENST00000435536 | 56.00990204 | 26.19428178 | 0.467672 | -2.13825 | 0.01039843 | 0.1198419 |
| DUSP1 | NM_004417 | 114.625165 | 62.41020303 | 0.544471 | -1.83665 | 0.02442038 | 0.178634 |
| DYNLT3 | NM_006520 | 46.79362207 | 104.7872952 | 2.23935 | 2.23935 | 0.004339298 | 0.08148064 |
| DNAH6 | NM_001370 | 24.9682015 | 12.23235266 | 0.489915 | -2.04117 | 0.001010636 | 0.04604891 |
| DNAH6 | BC015442 | 17.92076103 | 8.711734579 | 0.486127 | -2.05708 | 0.000474679 | 0.03563133 |
| DNAH7 | NM_018897 | 16.57940124 | 9.441002098 | 0.569444 | -1.7561 | 0.000819116 | 0.04297964 |
| DNAH12 | NM_198564 | 38.55082956 | 15.07639924 | 0.391079 | -2.55703 | 7.35E-05 | 0.02125486 |
| DNAH5 | NM_001369 | 24.75537674 | 13.03248234 | 0.526448 | -1.89952 | 0.000590874 | 0.03864177 |
| DNAI1 | NM_012144 | 38.10213674 | 17.52035376 | 0.459826 | -2.17474 | 0.000128469 | 0.02475431 |
| DNAH10 | BC144575 | 20.72413527 | 11.08156703 | 0.534719 | -1.87014 | 0.000434445 | 0.03491225 |
| DYNLRB2 | NM_130897 | 25.97478676 | 13.31619503 | 0.512656 | -1.95062 | 0.000166211 | 0.02616365 |
| DNAAF1 | NM_178452 | 106.7550125 | 36.47813332 | 0.3417 | -2.92654 | 3.86E-05 | 0.01858576 |
| DNAH3 | ENST00000544558 | 19.0304794 | 8.723759514 | 0.458412 | -2.18144 | 0.000105743 | 0.02280548 |
| DNAH3 | NM_017539 | 30.0538935 | 15.37953522 | 0.511731 | -1.95415 | 0.000301972 | 0.03051572 |
| DNAH9 | ENST00000262442 | 31.59271458 | 16.98924694 | 0.537759 | -1.85957 | 9.49E-05 | 0.02257817 |
| DYTN | NM_001093730 | 18.57782883 | 9.485935113 | 0.510606 | -1.95846 | 0.00019914 | 0.0271099 |
| EGR1 | NM_001964 | 617.5704873 | 330.2512015 | 0.53476 | -1.87 | 0.017594 | 0.1533343 |
| EFCAB1 | NM_024593 | 16.4054129 | 8.74446427 | 0.533024 | -1.87609 | 0.000180334 | 0.026672 |
| EFHC1 | NR_033327 | 38.02061597 | 19.54327033 | 0.514019 | -1.94545 | 0.001079904 | 0.04731795 |
| ENKUR | NM_145010 | 25.0236442 | 12.26648505 | 0.490198 | -2.03999 | 0.00088567 | 0.04393695 |
| FAM216B | NM_182508 | 28.69246477 | 12.59526874 | 0.438974 | -2.27804 | 0.00114586 | 0.04849595 |
| FAM91A1 | DQ228141 | 13.66648191 | 25.24017486 | 1.84686 | 1.84686 | 0.01019395 | 0.1190328 |
| FOS | NM_005252 | 198.6589526 | 95.5451012 | 0.480952 | -2.07921 | 0.01695272 | 0.150832 |
| FGFBP1 | NM_005130 | 117.4383668 | 222.8547653 | 1.89763 | 1.89763 | 0.0013484 | 0.0515709 |
| FLRT3 | NM_198391 | 22.96651421 | 12.08043081 | 0.526004 | -1.90113 | 0.000262428 | 0.02864118 |
| FANK1 | ENST00000368693 | 36.51152442 | 20.30706732 | 0.556184 | -1.79797 | 0.000532316 | 0.0374012 |
| GPR87 | NM_023915 | 24.87250657 | 44.12670138 | 1.77412 | 1.77412 | 0.01453876 | 0.1403374 |
| GJB6 | NM_001110219 | 21.74632938 | 55.28514583 | 2.54228 | 2.54228 | 0.000269965 | 0.02926864 |
| GPX2 | ENST00000389614 | 62.08272594 | 148.9671072 | 2.3995 | 2.3995 | 0.00065273 | 0.03972403 |
| GSTA1 | NM_145740 | 110.8657554 | 59.72777194 | 0.53874 | -1.85618 | 0.005978849 | 0.0928825 |
| GSTA2 | NM_000846 | 60.65564446 | 14.06112382 | 0.23182 | -4.3137 | 5.20E-05 | 0.02053801 |
| GPNMB | NM_001005340 | 59.53226995 | 133.399551 | 2.24079 | 2.24079 | 0.01311376 | 0.1329311 |
| HIST1H2BK | NM_080593 | 43.81005133 | 104.7204942 | 2.39033 | 2.39033 | 0.008999026 | 0.1119468 |
| HIST2H2AB | NM_175065 | 63.71274099 | 133.102144 | 2.08909 | 2.08909 | 0.004340092 | 0.08148064 |
| HYDIN | NM_032821 | 46.98831081 | 21.72011746 | 0.462245 | -2.16335 | 8.89E-05 | 0.02239032 |
| IGF2BP3 | NM_006547 | 18.45333925 | 34.49606747 | 1.86937 | 1.86937 | 0.004746828 | 0.08474345 |
| ITGA6 | NM_000210 | 29.20756131 | 56.65651666 | 1.93979 | 1.93979 | 0.001957804 | 0.06030244 |
| IQUB | ENST00000466202 | 10.35474414 | 5.643499318 | 0.545017 | -1.83481 | 0.000524941 | 0.03736411 |
| JUP | NM_002230 | 94.79506173 | 170.4340758 | 1.79791 | 1.79791 | 0.000642179 | 0.03952268 |
| KRT13 | ENST00000246635 | 145.6790895 | 756.890086 | 5.1956 | 5.1956 | 6.79E-06 | 0.01094802 |
| KRT16 | NM_005557 | 52.12956803 | 165.1576516 | 3.1682 | 3.1682 | 0.00107403 | 0.04731795 |
| KRT5 | ENST00000252242 | 169.4293498 | 319.3246246 | 1.8847 | 1.8847 | 7.61E-05 | 0.02125486 |
| KRT6B | NM_005555 | 389.9809927 | 2724.545748 | 6.98635 | 6.98635 | 8.72E-06 | 0.01094802 |
| KRT6C | NM_173086 | 32.31472927 | 127.5942937 | 3.94851 | 3.94851 | 0.000230922 | 0.02778307 |
| LRRC23 | NM_001135217 | 74.09580704 | 31.97538888 | 0.431541 | -2.31727 | 0.000187362 | 0.026672 |
| LRRC46 | NM_033413 | 37.63961588 | 19.08305176 | 0.506994 | -1.97241 | 0.000445253 | 0.03491225 |
| LRRIQ1 | NM_001079910 | 22.71148287 | 9.8476535 | 0.433595 | -2.3063 | 0.000449744 | 0.03491225 |
| MALL | NM_005434 | 90.92593809 | 159.5793318 | 1.75505 | 1.75505 | 0.003897635 | 0.07801853 |
| MGP | NM_001190839 | 77.95069604 | 33.15673399 | 0.425355 | -2.35098 | 0.000138128 | 0.02538796 |
| MS4A8B | ENST00000450141 | 92.28536677 | 32.43411121 | 0.351454 | -2.84532 | 0.000178616 | 0.026672 |
| MFAP4 | NM_001198695 | 49.798832 | 25.15389659 | 0.505111 | -1.97976 | 0.000334121 | 0.03188305 |
| MSMB | NM_002443 | 597.8708244 | 100.2494145 | 0.167678 | -5.96383 | 1.07E-05 | 0.01094802 |
| MAPK6 | NM_002748 | 32.44535396 | 57.60611262 | 1.77549 | 1.77549 | 0.000848261 | 0.04348074 |
| NBEA | NM_015678 | 20.65400993 | 11.75506002 | 0.569141 | -1.75703 | 8.63E-05 | 0.02239032 |
| NTRK2 | NM_006180 | 20.44108683 | 40.74440337 | 1.99326 | 1.99326 | 0.006616017 | 0.09713281 |
| NAMPT | NM_005746 | 371.8198547 | 700.5430985 | 1.8841 | 1.8841 | 0.006281421 | 0.09497507 |
| NEK5 | NM_199289 | 23.02485235 | 12.50818017 | 0.543248 | -1.84078 | 7.71E-05 | 0.02125486 |
| NME5 | NM_003551 | 27.38055908 | 14.50752993 | 0.529848 | -1.88733 | 0.0006389 | 0.03946926 |
| NR4A2 | ENST00000409572 | 51.88659649 | 29.62945121 | 0.571045 | -1.75117 | 0.01534591 | 0.1443827 |
| NR4A3 | NM_173200 | 33.98296906 | 19.0278414 | 0.559923 | -1.78596 | 0.004320013 | 0.08138366 |
| NR4A1 | NM_002135 | 97.42634242 | 55.22424941 | 0.566829 | -1.7642 | 0.005455943 | 0.08947323 |
| ODC1 | ENST00000234111 | 55.74188944 | 101.6079869 | 1.82283 | 1.82283 | 0.01802043 | 0.1550986 |
| PI3 | ENST00000243924 | 41.58899738 | 141.1670915 | 3.39433 | 3.39433 | 0.000727051 | 0.04076209 |
| PLA2G10 | NM_003561 | 108.6187985 | 57.10439972 | 0.525734 | -1.9021 | 0.02728745 | 0.188085 |
| PIH1D2 | ENST00000532211 | 16.9786518 | 8.883203245 | 0.523198 | -1.91132 | 0.001413835 | 0.05244569 |
| PKP1 | ENST00000263946 | 40.57361659 | 79.24126465 | 1.95302 | 1.95302 | 0.002043676 | 0.06127613 |
| PLEKHS1 | ENST00000369312 | 72.20465776 | 27.27183744 | 0.377704 | -2.64758 | 0.000901589 | 0.04434425 |
| PIGR | NM_002644 | 156.3732577 | 73.85583495 | 0.472305 | -2.11728 | 0.000783167 | 0.0420365 |
| KCNRG | NM_199464 | 59.18952607 | 32.07550335 | 0.54191 | -1.84532 | 0.01191435 | 0.1271501 |
| KCNE1 | ENST00000399286 | 52.52964757 | 28.04302352 | 0.533849 | -1.87319 | 0.000437453 | 0.03491225 |
| PRH1-PRR4 | ENST00000228811 | 146.9416938 | 76.89457964 | 0.523302 | -1.91094 | 0.002310751 | 0.06398399 |
| PROM1 | NM_001145847 | 30.40127658 | 14.25691568 | 0.468958 | -2.13239 | 0.000196146 | 0.0271099 |
| PRSS23 | NM_007173 | 167.3830928 | 82.70198202 | 0.494088 | -2.02393 | 0.002119182 | 0.06268083 |
| RP1 | NM_006269 | 16.3002253 | 7.775093367 | 0.476992 | -2.09647 | 7.07E-06 | 0.01094802 |
| RRM2 | ENST00000360566 | 53.48268537 | 119.8038117 | 2.24005 | 2.24005 | 0.000333284 | 0.03188305 |
| RPL39L | ENST00000296277 | 27.1011093 | 52.55951289 | 1.93938 | 1.93938 | 5.52E-05 | 0.02053801 |
| RPF2 | ENST00000441448 | 12.84780503 | 24.76533103 | 1.92761 | 1.92761 | 0.007710665 | 0.1040646 |
| S100A10 | NM_002966 | 110.2977843 | 213.2807576 | 1.93369 | 1.93369 | 0.007171037 | 0.1005066 |
| S100A16 | ENST00000368703 | 30.72284684 | 63.66815267 | 2.07234 | 2.07234 | 0.000598862 | 0.03875058 |
| S100A8 | NM_002964 | 45.20561261 | 186.1742841 | 4.11841 | 4.11841 | 0.008160469 | 0.1075636 |
| S100A9 | NM_002965 | 100.8914424 | 256.4368887 | 2.54171 | 2.54171 | 0.002938424 | 0.07007635 |
| SATB1 | NM_001195470 | 76.4057866 | 42.32505389 | 0.553954 | -1.80521 | 0.003276435 | 0.07237403 |
| SCGB3A1 | ENST00000292641 | 110.4232379 | 58.1287508 | 0.526419 | -1.89963 | 0.001197908 | 0.04949683 |
| SCGB1A1 | NM_003357 | 1501.095449 | 333.730562 | 0.222322 | -4.49798 | 0.001673488 | 0.05698623 |
| STK33 | NM_030906 | 22.93914954 | 12.28563055 | 0.535577 | -1.86715 | 0.000342326 | 0.03201394 |
| SERPINB13 | NM_012397 | 64.78105238 | 167.8117587 | 2.59046 | 2.59046 | 0.000245387 | 0.02778307 |
| SGK1 | NM_001143676 | 62.51844543 | 117.155429 | 1.87393 | 1.87393 | 0.001677103 | 0.05698623 |
| SLITRK6 | NM_032229 | 39.66487232 | 17.21208542 | 0.433936 | -2.30449 | 0.000282598 | 0.02993198 |
| SPRR1A | NM_001199828 | 232.8698414 | 981.952889 | 4.21676 | 4.21676 | 0.000463163 | 0.03541919 |
| SPRR1B | NM_003125 | 22.24933019 | 74.67482259 | 3.35626 | 3.35626 | 0.00113268 | 0.04841505 |
| SPRR2A | NM_005988 | 35.62060216 | 309.5974975 | 8.69157 | 8.69157 | 0.000707139 | 0.04076209 |
| SPRR3 | NM_005416 | 51.3509834 | 257.6805795 | 5.01802 | 5.01802 | 0.000206833 | 0.0272835 |
| SLC44A1 | NM_080546 | 20.13201957 | 37.1448424 | 1.84507 | 1.84507 | 2.66E-05 | 0.01535475 |
| SLC44A4 | ENST00000453831 | 100.062667 | 54.88081496 | 0.548465 | -1.82327 | 0.002409222 | 0.06480839 |
| SPARCL1 | NM_001128310 | 67.50488492 | 37.32059288 | 0.552859 | -1.80878 | 0.002202922 | 0.06326977 |
| SPAG8 | NM_172312 | 23.24341267 | 9.043617659 | 0.389085 | -2.57013 | 1.92E-05 | 0.0130967 |
| SPA17 | ENST00000524614 | 34.57075006 | 17.5576761 | 0.507874 | -1.96899 | 0.001165594 | 0.04887109 |
| SPEF2 | NM_024867 | 16.95184034 | 9.676598479 | 0.570829 | -1.75184 | 0.000621491 | 0.03913213 |
| SPATA18 | NM_145263 | 42.85079306 | 22.17542731 | 0.517503 | -1.93235 | 0.000439682 | 0.03491225 |
| SCD | NM_005063 | 38.87363197 | 98.06388412 | 2.52264 | 2.52264 | 0.002129046 | 0.06268083 |
| SAMD9 | NM_017654 | 20.95772191 | 44.93039998 | 2.14386 | 2.14386 | 0.000502965 | 0.036185 |
| SFN | NM_006142 | 166.4332646 | 295.6343301 | 1.77629 | 1.77629 | 3.51E-05 | 0.01853606 |
| SMC4 | NM_005496 | 23.33558937 | 41.91689651 | 1.79626 | 1.79626 | 0.000256099 | 0.02833821 |
| TEKT1 | ENST00000338694 | 52.64592645 | 24.63828615 | 0.467997 | -2.13676 | 0.000158053 | 0.02567099 |
| TSPAN1 | NM_005727 | 240.7563434 | 95.35852399 | 0.396081 | -2.52474 | 0.001815277 | 0.05836478 |
| TSPAN8 | ENST00000393330 | 131.1069178 | 29.25781243 | 0.223159 | -4.48111 | 0.000155066 | 0.02567099 |
| TTC18 | AF435959 | 20.792625 | 10.02840319 | 0.482307 | -2.07337 | 0.000224065 | 0.02774088 |
| TTC25 | NM_031421 | 23.06254786 | 13.17106409 | 0.571104 | -1.75099 | 0.000206535 | 0.0272835 |
| TOP2A | NM_001067 | 13.42295124 | 28.136286 | 2.09613 | 2.09613 | 0.000155122 | 0.02567099 |
| TFRC | NM_003234 | 24.20444317 | 44.95407518 | 1.85727 | 1.85727 | 0.000312492 | 0.03094845 |
| TM4SF1 | ENST00000305366 | 72.8178379 | 165.1027111 | 2.26733 | 2.26733 | 0.000106431 | 0.02280548 |
| TMC5 | NM_001105248 | 59.12105053 | 32.72882718 | 0.55359 | -1.80639 | 0.001197072 | 0.04949683 |
| TMPRSS11A | ENST00000334830 | 16.92096568 | 31.27482702 | 1.84828 | 1.84828 | 0.03315756 | 0.2051711 |
| TFF3 | ENST00000518498 | 156.9531311 | 80.88386319 | 0.515337 | -1.94048 | 8.13E-06 | 0.01094802 |
| TUBB | ENST00000421473 | 164.4163544 | 315.3324869 | 1.91788 | 1.91788 | 0.001131501 | 0.04841505 |
| TUBA1B | NM_006082 | 204.1590909 | 516.6272745 | 2.53052 | 2.53052 | 0.00403848 | 0.07889435 |
| TUBA4B | NR_003063 | 72.67866427 | 30.85152716 | 0.424491 | -2.35576 | 4.06E-05 | 0.01899022 |
| TUBA1A | NM_001270399 | 164.1316893 | 68.38793869 | 0.416664 | -2.40001 | 1.35E-05 | 0.01295435 |
| TPPP3 | ENST00000562206 | 112.9390037 | 56.24956366 | 0.498053 | -2.00782 | 0.000135708 | 0.02524613 |
| UBXN10 | ENST00000375099 | 57.21969887 | 29.53164877 | 0.516111 | -1.93757 | 0.002424303 | 0.06484962 |
| LOC100506948 | ENST00000551938 | 26.79960812 | 14.61562886 | 0.545369 | -1.83362 | 0.02447837 | 0.1788012 |
| VWA3B | NM_144992 | 26.95105793 | 14.27362628 | 0.529614 | -1.88817 | 0.000146778 | 0.02567099 |
| WFDC6 | NM_080827 | 41.12061681 | 22.30538276 | 0.542437 | -1.84353 | 0.000225256 | 0.02774088 |
| WDR63 | NM_145172 | 17.31691461 | 9.186402524 | 0.530487 | -1.88506 | 0.000528735 | 0.0374012 |
| WDR96 | NM_025145 | 27.12779721 | 11.22901908 | 0.41393 | -2.41587 | 0.000107265 | 0.02280548 |
| ZBBX | ENST00000307529 | 27.42633611 | 10.30862494 | 0.375867 | -2.66052 | 9.76E-05 | 0.02257817 |
| ZMAT1 | NR_046008 | 25.92226722 | 12.71103851 | 0.49035 | -2.03936 | 6.33E-07 | 0.005181383 |
| ZC3H11A | ENST00000545588 | 58.86997052 | 103.0485218 | 1.75044 | 1.75044 | 0.03697405 | 0.2151631 |
| ZNF273 | NR_003099 | 54.34668001 | 20.45837994 | 0.376443 | -2.65645 | 0.000293267 | 0.03019521 |
| ZNF474 | ENST00000296600 | 36.22842043 | 20.40555436 | 0.563246 | -1.77542 | 0.00088877 | 0.04395754 |

Additional file 5. Table S5. Differential RNA expression in PSCC and ISCC.

| **Gene symbol** | **RefSeq** | **Mean ISCC** | **Mean control** | **Ratio** | **Fold change** | **P-value** | **Q-value** |
| --- | --- | --- | --- | --- | --- | --- | --- |
| ABI3BP | NM_015429 | 55.21659424 | 30.12898152 | 0.545654 | -1.83266 | 6.38E-06 | 0.01094802 |
| ADAM23 | ENST00000264377 | 16.90361607 | 30.94318936 | 1.83056 | 1.83056 | 0.008953124 | 0.1116302 |
| AKD1 | ENST00000285397 | 20.10064652 | 10.30619579 | 0.512728 | -1.95035 | 0.000261232 | 0.02864118 |
| A2M | ENST00000318602 | 121.2759777 | 54.23566632 | 0.44721 | -2.23609 | 0.000723832 | 0.04076209 |
| APOBEC4 | NM_203454 | 32.29009992 | 14.29174329 | 0.442604 | -2.25936 | 0.000185249 | 0.026672 |
| APOD | NM_001647 | 186.6070911 | 74.40821474 | 0.398741 | -2.50789 | 0.000313816 | 0.03094845 |
| ABCC5 | NM_005688 | 42.72563415 | 82.35018581 | 1.92742 | 1.92742 | 0.004588493 | 0.08316321 |
| AGBL2 | NM_024783 | 27.64238131 | 12.66214573 | 0.45807 | -2.18307 | 0.000305026 | 0.03063523 |
| BEST4 | NM_153274 | 29.61713123 | 15.28950717 | 0.516238 | -1.93709 | 2.52E-05 | 0.01535475 |
| BPIFA1 | NM_130852 | 139.5008401 | 36.42961896 | 0.261143 | -3.82932 | 0.00157352 | 0.05599979 |
| BPIFB1 | NM_033197 | 903.7688358 | 191.9650685 | 0.212405 | -4.70799 | 9.29E-05 | 0.02257817 |
| CDHR3 | NM_152750 | 81.1241752 | 25.55458044 | 0.315005 | -3.17455 | 8.62E-05 | 0.02239032 |
| CASC1 | NM_001082972 | 15.69246406 | 7.603955031 | 0.48456 | -2.06373 | 0.000619111 | 0.03913213 |
| CHST9 | NM_031422 | 48.72228872 | 25.1464005 | 0.516116 | -1.93755 | 0.000753278 | 0.04138195 |
| CKS2 | NM_001827 | 15.39702803 | 27.01034196 | 1.75426 | 1.75426 | 0.009440137 | 0.1143918 |
| CDV3 | ENST00000431519 | 66.62769135 | 117.3041302 | 1.76059 | 1.76059 | 0.001288407 | 0.05075373 |
| CDCA7L | NM_018719 | 20.82031513 | 10.97257328 | 0.527016 | -1.89748 | 0.000181243 | 0.026672 |
| CCL15 | ENST00000495214 | 66.16837755 | 30.60382344 | 0.462514 | -2.1621 | 6.55E-05 | 0.02125486 |
| C1orf158 | NM_152290 | 44.38775998 | 22.93851354 | 0.516778 | -1.93507 | 0.000918178 | 0.04447154 |
| C1orf168 | NM_001004303 | 18.52138397 | 9.666341739 | 0.5219 | -1.91608 | 2.60E-06 | 0.008512856 |
| C1orf173 | ENST00000326665 | 25.46881622 | 13.34964988 | 0.524155 | -1.90783 | 1.77E-05 | 0.01295435 |
| C1orf87 | ENST00000371201 | 22.13396487 | 12.63698155 | 0.570932 | -1.75152 | 2.37E-05 | 0.01535475 |
| C1orf88 | ENST00000369738 | 37.21313424 | 14.38795852 | 0.386636 | -2.58641 | 0.000229349 | 0.02778307 |
| C11orf88 | ENST00000529167 | 27.58457791 | 15.66985582 | 0.568065 | -1.76036 | 0.001329922 | 0.05099559 |
| C12orf63 | ENST00000342887 | 17.39836704 | 9.171896023 | 0.527168 | -1.89693 | 0.000922645 | 0.04452118 |
| C2orf40 | ENST00000238044 | 27.15789959 | 13.83556831 | 0.509449 | -1.9629 | 1.98E-06 | 0.008103584 |
| C20orf85 | ENST00000371168 | 55.32578074 | 19.56888973 | 0.353704 | -2.82723 | 0.000165214 | 0.02616365 |
| C3orf15 | NM_033364 | 24.16504875 | 12.78978405 | 0.529269 | -1.8894 | 6.87E-05 | 0.02125486 |
| C6orf165 | ENST00000369562 | 14.91394478 | 8.460306909 | 0.567276 | -1.76281 | 0.000703385 | 0.04076209 |
| CLUAP1 | NM_015041 | 35.03900737 | 19.37279221 | 0.55289 | -1.80868 | 0.00103386 | 0.04661158 |
| CCDC108 | NM_194302 | 122.5715207 | 31.62623691 | 0.258024 | -3.87561 | 3.49E-07 | 0.005181383 |
| CCDC146 | NM_020879 | 42.00415094 | 22.25889393 | 0.529921 | -1.88708 | 0.001630172 | 0.05644258 |
| CCDC19 | ENST00000368099 | 42.4534531 | 23.15336535 | 0.545383 | -1.83357 | 0.00058107 | 0.03864177 |
| CCDC30 | ENST00000428554 | 20.6066774 | 10.72904677 | 0.52066 | -1.92064 | 0.000558759 | 0.03795591 |
| CTGF | ENST00000367976 | 103.9625417 | 47.29776149 | 0.45495 | -2.19805 | 2.72E-05 | 0.01535475 |
| COX6B1 | NM_001863 | 72.48195683 | 133.6022042 | 1.84324 | 1.84324 | 0.02914321 | 0.1939123 |
| CYP2B7P1 | NR_001278 | 64.42103793 | 34.18638572 | 0.530673 | -1.8844 | 0.02657314 | 0.1856961 |
| DLEC1 | ENST00000308059 | 48.26013346 | 25.21656745 | 0.522514 | -1.91382 | 0.000213091 | 0.02732415 |
| DSC2 | NM_024422 | 22.61111138 | 50.59524851 | 2.23762 | 2.23762 | 0.002209684 | 0.06335283 |
| DSG3 | NM_001944 | 26.67710114 | 81.79722529 | 3.0662 | 3.0662 | 0.001773898 | 0.0579646 |
| DSP | ENST00000379802 | 80.2533661 | 168.4947698 | 2.09953 | 2.09953 | 0.000592272 | 0.03864177 |
| DLG1 | NM_004087 | 40.65018464 | 72.94615346 | 1.79449 | 1.79449 | 0.003819338 | 0.07721593 |
| DCDC1 | NM_181807 | 26.64291443 | 14.29471548 | 0.536531 | -1.86383 | 0.000334978 | 0.03188305 |
| DPY19L2P2 | ENST00000435536 | 56.00990204 | 26.19428178 | 0.467672 | -2.13825 | 0.01039843 | 0.1198419 |
| DUSP1 | NM_004417 | 114.625165 | 62.41020303 | 0.544471 | -1.83665 | 0.02442038 | 0.178634 |
| DNAI1 | NM_012144 | 38.10213674 | 17.52035376 | 0.459826 | -2.17474 | 0.000128469 | 0.02475431 |
| DNAH6 | NM_001370 | 24.9682015 | 12.23235266 | 0.489915 | -2.04117 | 0.001010636 | 0.04604891 |
| DNAH6 | BC015442 | 17.92076103 | 8.711734579 | 0.486127 | -2.05708 | 0.000474679 | 0.03563133 |
| DNAH12 | NM_198564 | 38.55082956 | 15.07639924 | 0.391079 | -2.55703 | 7.35E-05 | 0.02125486 |
| DNAAF1 | NM_178452 | 106.7550125 | 36.47813332 | 0.3417 | -2.92654 | 3.86E-05 | 0.01858576 |
| DYNLRB2 | NM_130897 | 25.97478676 | 13.31619503 | 0.512656 | -1.95062 | 0.000166211 | 0.02616365 |
| DNAH7 | NM_018897 | 16.57940124 | 9.441002098 | 0.569444 | -1.7561 | 0.000819116 | 0.04297964 |
| DNAH9 | ENST00000262442 | 31.59271458 | 16.98924694 | 0.537759 | -1.85957 | 9.49E-05 | 0.02257817 |
| DNAH10 | BC144575 | 20.72413527 | 11.08156703 | 0.534719 | -1.87014 | 0.000434445 | 0.03491225 |
| DNAH3 | ENST00000544558 | 19.0304794 | 8.723759514 | 0.458412 | -2.18144 | 0.000105743 | 0.02280548 |
| DNAH3 | NM_017539 | 30.0538935 | 15.37953522 | 0.511731 | -1.95415 | 0.000301972 | 0.03051572 |
| DNAH5 | NM_001369 | 24.75537674 | 13.03248234 | 0.526448 | -1.89952 | 0.000590874 | 0.03864177 |
| DYTN | NM_001093730 | 18.57782883 | 9.485935113 | 0.510606 | -1.95846 | 0.00019914 | 0.0271099 |
| EGR1 | NM_001964 | 617.5704873 | 330.2512015 | 0.53476 | -1.87 | 0.017594 | 0.1533343 |
| EFCAB1 | NM_024593 | 16.4054129 | 8.74446427 | 0.533024 | -1.87609 | 0.000180334 | 0.026672 |
| EFHC1 | NR_033327 | 38.02061597 | 19.54327033 | 0.514019 | -1.94545 | 0.001079904 | 0.04731795 |
| ENKUR | NM_145010 | 25.0236442 | 12.26648505 | 0.490198 | -2.03999 | 0.00088567 | 0.04393695 |
| FAM216B | NM_182508 | 28.69246477 | 12.59526874 | 0.438974 | -2.27804 | 0.00114586 | 0.04849595 |
| FOS | NM_005252 | 198.6589526 | 95.5451012 | 0.480952 | -2.07921 | 0.01695272 | 0.150832 |
| FANK1 | ENST00000368693 | 36.51152442 | 20.30706732 | 0.556184 | -1.79797 | 0.000532316 | 0.0374012 |
| GJB6 | NM_001110219 | 21.74632938 | 55.28514583 | 2.54228 | 2.54228 | 0.000269965 | 0.02926864 |
| GSTA1 | NM_145740 | 110.8657554 | 59.72777194 | 0.53874 | -1.85618 | 0.005978849 | 0.0928825 |
| GSTA2 | NM_000846 | 60.65564446 | 14.06112382 | 0.23182 | -4.3137 | 5.20E-05 | 0.02053801 |
| GPNMB | NM_001005340 | 59.53226995 | 133.399551 | 2.24079 | 2.24079 | 0.01311376 | 0.1329311 |
| HIST1H2BK | NM_080593 | 43.81005133 | 104.7204942 | 2.39033 | 2.39033 | 0.008999026 | 0.1119468 |
| HIST2H2AB | NM_175065 | 63.71274099 | 133.102144 | 2.08909 | 2.08909 | 0.004340092 | 0.08148064 |
| HYDIN | NM_032821 | 46.98831081 | 21.72011746 | 0.462245 | -2.16335 | 8.89E-05 | 0.02239032 |
| IGF2BP3 | NM_006547 | 18.45333925 | 34.49606747 | 1.86937 | 1.86937 | 0.004746828 | 0.08474345 |
| ITGA6 | NM_000210 | 29.20756131 | 56.65651666 | 1.93979 | 1.93979 | 0.001957804 | 0.06030244 |
| IQUB | ENST00000466202 | 10.35474414 | 5.643499318 | 0.545017 | -1.83481 | 0.000524941 | 0.03736411 |
| JUP | NM_002230 | 94.79506173 | 170.4340758 | 1.79791 | 1.79791 | 0.000642179 | 0.03952268 |
| KRT16 | NM_005557 | 52.12956803 | 165.1576516 | 3.1682 | 3.1682 | 0.00107403 | 0.04731795 |
| LRRC23 | NM_001135217 | 74.09580704 | 31.97538888 | 0.431541 | -2.31727 | 0.000187362 | 0.026672 |
| LRRC46 | NM_033413 | 37.63961588 | 19.08305176 | 0.506994 | -1.97241 | 0.000445253 | 0.03491225 |
| LRRIQ1 | NM_001079910 | 22.71148287 | 9.8476535 | 0.433595 | -2.3063 | 0.000449744 | 0.03491225 |
| MS4A8B | ENST00000450141 | 92.28536677 | 32.43411121 | 0.351454 | -2.84532 | 0.000178616 | 0.026672 |
| MFAP4 | NM_001198695 | 49.798832 | 25.15389659 | 0.505111 | -1.97976 | 0.000334121 | 0.03188305 |
| MSMB | NM_002443 | 597.8708244 | 100.2494145 | 0.167678 | -5.96383 | 1.07E-05 | 0.01094802 |
| NBEA | NM_015678 | 20.65400993 | 11.75506002 | 0.569141 | -1.75703 | 8.63E-05 | 0.02239032 |
| NTRK2 | NM_006180 | 20.44108683 | 40.74440337 | 1.99326 | 1.99326 | 0.006616017 | 0.09713281 |
| NEK5 | NM_199289 | 23.02485235 | 12.50818017 | 0.543248 | -1.84078 | 7.71E-05 | 0.02125486 |
| NME5 | NM_003551 | 27.38055908 | 14.50752993 | 0.529848 | -1.88733 | 0.0006389 | 0.03946926 |
| NR4A2 | ENST00000409572 | 51.88659649 | 29.62945121 | 0.571045 | -1.75117 | 0.01534591 | 0.1443827 |
| NR4A3 | NM_173200 | 33.98296906 | 19.0278414 | 0.559923 | -1.78596 | 0.004320013 | 0.08138366 |
| NR4A1 | NM_002135 | 97.42634242 | 55.22424941 | 0.566829 | -1.7642 | 0.005455943 | 0.08947323 |
| ODC1 | ENST00000234111 | 55.74188944 | 101.6079869 | 1.82283 | 1.82283 | 0.01802043 | 0.1550986 |
| PLA2G10 | NM_003561 | 108.6187985 | 57.10439972 | 0.525734 | -1.9021 | 0.02728745 | 0.188085 |
| PIH1D2 | ENST00000532211 | 16.9786518 | 8.883203245 | 0.523198 | -1.91132 | 0.001413835 | 0.05244569 |
| PKP1 | ENST00000263946 | 40.57361659 | 79.24126465 | 1.95302 | 1.95302 | 0.002043676 | 0.06127613 |
| PLEKHS1 | ENST00000369312 | 72.20465776 | 27.27183744 | 0.377704 | -2.64758 | 0.000901589 | 0.04434425 |
| PIGR | NM_002644 | 156.3732577 | 73.85583495 | 0.472305 | -2.11728 | 0.000783167 | 0.0420365 |
| KCNRG | NM_199464 | 59.18952607 | 32.07550335 | 0.54191 | -1.84532 | 0.01191435 | 0.1271501 |
| KCNE1 | ENST00000399286 | 52.52964757 | 28.04302352 | 0.533849 | -1.87319 | 0.000437453 | 0.03491225 |
| PRH1-PRR4 | ENST00000228811 | 146.9416938 | 76.89457964 | 0.523302 | -1.91094 | 0.002310751 | 0.06398399 |
| PROM1 | NM_001145847 | 30.40127658 | 14.25691568 | 0.468958 | -2.13239 | 0.000196146 | 0.0271099 |
| PRSS23 | NM_007173 | 167.3830928 | 82.70198202 | 0.494088 | -2.02393 | 0.002119182 | 0.06268083 |
| RP1 | NM_006269 | 16.3002253 | 7.775093367 | 0.476992 | -2.09647 | 7.07E-06 | 0.01094802 |
| RRM2 | ENST00000360566 | 53.48268537 | 119.8038117 | 2.24005 | 2.24005 | 0.000333284 | 0.03188305 |
| RPL39L | ENST00000296277 | 27.1011093 | 52.55951289 | 1.93938 | 1.93938 | 5.52E-05 | 0.02053801 |
| RPF2 | ENST00000441448 | 12.84780503 | 24.76533103 | 1.92761 | 1.92761 | 0.007710665 | 0.1040646 |
| SATB1 | NM_001195470 | 76.4057866 | 42.32505389 | 0.553954 | -1.80521 | 0.003276435 | 0.07237403 |
| SCGB3A1 | ENST00000292641 | 110.4232379 | 58.1287508 | 0.526419 | -1.89963 | 0.001197908 | 0.04949683 |
| SCGB1A1 | NM_003357 | 1501.095449 | 333.730562 | 0.222322 | -4.49798 | 0.001673488 | 0.05698623 |
| STK33 | NM_030906 | 22.93914954 | 12.28563055 | 0.535577 | -1.86715 | 0.000342326 | 0.03201394 |
| SLITRK6 | NM_032229 | 39.66487232 | 17.21208542 | 0.433936 | -2.30449 | 0.000282598 | 0.02993198 |
| SPRR2A | NM_005988 | 35.62060216 | 309.5974975 | 8.69157 | 8.69157 | 0.000707139 | 0.04076209 |
| SLC44A1 | NM_080546 | 20.13201957 | 37.1448424 | 1.84507 | 1.84507 | 2.66E-05 | 0.01535475 |
| SLC44A4 | ENST00000453831 | 100.062667 | 54.88081496 | 0.548465 | -1.82327 | 0.002409222 | 0.06480839 |
| SPAG8 | NM_172312 | 23.24341267 | 9.043617659 | 0.389085 | -2.57013 | 1.92E-05 | 0.0130967 |
| SPA17 | ENST00000524614 | 34.57075006 | 17.5576761 | 0.507874 | -1.96899 | 0.001165594 | 0.04887109 |
| SPEF2 | NM_024867 | 16.95184034 | 9.676598479 | 0.570829 | -1.75184 | 0.000621491 | 0.03913213 |
| SPATA18 | NM_145263 | 42.85079306 | 22.17542731 | 0.517503 | -1.93235 | 0.000439682 | 0.03491225 |
| SCD | NM_005063 | 38.87363197 | 98.06388412 | 2.52264 | 2.52264 | 0.002129046 | 0.06268083 |
| SMC4 | NM_005496 | 23.33558937 | 41.91689651 | 1.79626 | 1.79626 | 0.000256099 | 0.02833821 |
| TEKT1 | ENST00000338694 | 52.64592645 | 24.63828615 | 0.467997 | -2.13676 | 0.000158053 | 0.02567099 |
| TSPAN1 | NM_005727 | 240.7563434 | 95.35852399 | 0.396081 | -2.52474 | 0.001815277 | 0.05836478 |
| TSPAN8 | ENST00000393330 | 131.1069178 | 29.25781243 | 0.223159 | -4.48111 | 0.000155066 | 0.02567099 |
| TTC18 | AF435959 | 20.792625 | 10.02840319 | 0.482307 | -2.07337 | 0.000224065 | 0.02774088 |
| TTC25 | NM_031421 | 23.06254786 | 13.17106409 | 0.571104 | -1.75099 | 0.000206535 | 0.0272835 |
| TOP2A | NM_001067 | 13.42295124 | 28.136286 | 2.09613 | 2.09613 | 0.000155122 | 0.02567099 |
| TFRC | NM_003234 | 24.20444317 | 44.95407518 | 1.85727 | 1.85727 | 0.000312492 | 0.03094845 |
| TMC5 | NM_001105248 | 59.12105053 | 32.72882718 | 0.55359 | -1.80639 | 0.001197072 | 0.04949683 |
| TFF3 | ENST00000518498 | 156.9531311 | 80.88386319 | 0.515337 | -1.94048 | 8.13E-06 | 0.01094802 |
| TUBB | ENST00000421473 | 164.4163544 | 315.3324869 | 1.91788 | 1.91788 | 0.001131501 | 0.04841505 |
| TUBA1A | NM_001270399 | 164.1316893 | 68.38793869 | 0.416664 | -2.40001 | 1.35E-05 | 0.01295435 |
| TUBA4B | NR_003063 | 72.67866427 | 30.85152716 | 0.424491 | -2.35576 | 4.06E-05 | 0.01899022 |
| TPPP3 | ENST00000562206 | 112.9390037 | 56.24956366 | 0.498053 | -2.00782 | 0.000135708 | 0.02524613 |
| UBXN10 | ENST00000375099 | 57.21969887 | 29.53164877 | 0.516111 | -1.93757 | 0.002424303 | 0.06484962 |
| LOC100506948 | ENST00000551938 | 26.79960812 | 14.61562886 | 0.545369 | -1.83362 | 0.02447837 | 0.1788012 |
| VWA3B | NM_144992 | 26.95105793 | 14.27362628 | 0.529614 | -1.88817 | 0.000146778 | 0.02567099 |
| WFDC6 | NM_080827 | 41.12061681 | 22.30538276 | 0.542437 | -1.84353 | 0.000225256 | 0.02774088 |
| WDR63 | NM_145172 | 17.31691461 | 9.186402524 | 0.530487 | -1.88506 | 0.000528735 | 0.0374012 |
| WDR96 | NM_025145 | 27.12779721 | 11.22901908 | 0.41393 | -2.41587 | 0.000107265 | 0.02280548 |
| ZMAT1 | NR_046008 | 25.92226722 | 12.71103851 | 0.49035 | -2.03936 | 6.33E-07 | 0.005181383 |
| ZBBX | ENST00000307529 | 27.42633611 | 10.30862494 | 0.375867 | -2.66052 | 9.76E-05 | 0.02257817 |
| ZNF273 | NR_003099 | 54.34668001 | 20.45837994 | 0.376443 | -2.65645 | 0.000293267 | 0.03019521 |
| ZNF474 | ENST00000296600 | 36.22842043 | 20.40555436 | 0.563246 | -1.77542 | 0.00088877 | 0.04395754 |

Additional file 6. Table S6. Differential RNA expression in high-grade PSCC versus paired controls.

| **Gene symbol** | **RefSeq** | **Mean ISCC** | **Mean control** | **Ratio** | **Fold change** | **P-value** | **Q-value** |
| --- | --- | --- | --- | --- | --- | --- | --- |
| MLLT11 | ENST00000368921 | 25.27168579 | 46.77254415 | 1.8508 | 1.8508 | 0.01932064 | 0.3574549 |
| SPRR1A | NM_001199828 | 239.2607606 | 965.3109942 | 4.03456 | 4.03456 | 0.04164113 | 0.4415689 |
| SPRR1B | NM_003125 | 22.43407819 | 87.79701588 | 3.91356 | 3.91356 | 0.0103863 | 0.3202938 |
| DAP3 | NM_033657 | 23.91294647 | 43.8592732 | 1.83412 | 1.83412 | 0.004379796 | 0.2829272 |
| XCL1 | NM_002995 | 7.362011483 | 18.64710808 | 2.53289 | 2.53289 | 0.00111544 | 0.2619793 |
| PRDX6 | ENST00000340385 | 101.6883081 | 179.8038373 | 1.76819 | 1.76819 | 0.03609548 | 0.4179626 |
| PKP1 | ENST00000263946 | 44.15791044 | 92.44606455 | 2.09354 | 2.09354 | 0.007717419 | 0.30416 |
| ZC3H11A | ENST00000545588 | 65.32303876 | 129.5827617 | 1.98373 | 1.98373 | 0.006516142 | 0.3013964 |
| DEGS1 | ENST00000323699 | 27.08364483 | 50.15772471 | 1.85196 | 1.85196 | 0.001691579 | 0.2619793 |
| CNIH4 | ENST00000465271 | 16.92202129 | 31.63325261 | 1.86935 | 1.86935 | 0.006997857 | 0.30416 |
| TMEM54 | NM_033504 | 46.60490835 | 84.53928467 | 1.81397 | 1.81397 | 0.003072968 | 0.2823126 |
| SF3A3 | ENST00000373019 | 149.9564742 | 271.8769437 | 1.81305 | 1.81305 | 0.003116605 | 0.2823126 |
| HIST2H2AB | NM_175065 | 65.14172415 | 162.3698548 | 2.49257 | 2.49257 | 0.003545282 | 0.2823126 |
| SPRR2A | NM_005988 | 40.50056137 | 335.0261739 | 8.27213 | 8.27213 | 0.01069638 | 0.3221695 |
| RIT1 | NM_006912 | 18.5896796 | 35.83680212 | 1.92778 | 1.92778 | 0.007433951 | 0.30416 |
| TMCO1 | NM_001256165 | 38.24104387 | 67.04742405 | 1.75328 | 1.75328 | 0.00091549 | 0.2619793 |
| NUCKS1 | ENST00000367142 | 113.410468 | 206.8311973 | 1.82374 | 1.82374 | 0.006677554 | 0.3028223 |
| TOMM20 | NM_014765 | 12.79670078 | 26.47135244 | 2.06861 | 2.06861 | 0.001900254 | 0.2619793 |
| RAB10 | NM_016131 | 49.01665059 | 89.90254178 | 1.83412 | 1.83412 | 0.02246689 | 0.3702301 |
| TACR1 | NM_001058 | 41.79185815 | 73.92292819 | 1.76885 | 1.76885 | 0.01199954 | 0.326829 |
| ITGA6 | NM_000210 | 29.51568665 | 57.13171766 | 1.93564 | 1.93564 | 0.004555192 | 0.2829272 |
| ADAM23 | ENST00000264377 | 20.2048537 | 49.71950403 | 2.46077 | 2.46077 | 0.001488647 | 0.2619793 |
| ODC1 | ENST00000234111 | 57.50358512 | 114.4354316 | 1.99005 | 1.99005 | 0.03323736 | 0.4080264 |
| COX7A2L | ENST00000378669 | 35.6925235 | 67.97488969 | 1.90447 | 1.90447 | 0.001156302 | 0.2619793 |
| TPRKB | NM_016058 | 16.92941246 | 29.72592915 | 1.75588 | 1.75588 | 0.004742468 | 0.2829272 |
| MOB1A | ENST00000396049 | 35.57544752 | 62.32374406 | 1.75188 | 1.75188 | 0.008494522 | 0.30416 |
| Sep-10 | NM_144710 | 16.82704568 | 32.40714449 | 1.9259 | 1.9259 | 0.02690322 | 0.3861136 |
| LOC440894 | NR_046110 | 17.94288535 | 32.59299447 | 1.81648 | 1.81648 | 0.01033781 | 0.3201187 |
| TIGD1 | NM_145702 | 11.25067769 | 20.1370438 | 1.78985 | 1.78985 | 0.000963834 | 0.2619793 |
| DTX3L | ENST00000296161 | 21.20760794 | 37.86177126 | 1.78529 | 1.78529 | 0.0442548 | 0.450333 |
| PARP14 | ENST00000474629 | 47.38997517 | 91.02367942 | 1.92075 | 1.92075 | 0.02355689 | 0.3769318 |
| CDV3 | ENST00000431519 | 73.20549297 | 135.7153411 | 1.8539 | 1.8539 | 0.02626905 | 0.3851257 |
| ATP1B3 | NM_001679 | 39.49505155 | 89.01027625 | 2.25369 | 2.25369 | 0.002117798 | 0.2653251 |
| SMC4 | NM_005496 | 23.43008041 | 45.81887083 | 1.95556 | 1.95556 | 0.002008502 | 0.2619793 |
| NDUFB5 | ENST00000259037 | 22.26275143 | 39.67229629 | 1.78199 | 1.78199 | 0.005836994 | 0.2961425 |
| SOX2-OT | NR_004053 | 140.458488 | 267.2451582 | 1.90267 | 1.90267 | 0.005840723 | 0.2961425 |
| PSMD2 | ENST00000310118 | 29.77665932 | 53.12982295 | 1.78427 | 1.78427 | 0.006199551 | 0.2969162 |
| TP63 | NM_003722 | 64.97577358 | 123.8980782 | 1.90683 | 1.90683 | 0.02467813 | 0.3788541 |
| PIGX | NM_001166304 | 11.74333277 | 21.12237273 | 1.79866 | 1.79866 | 0.000629493 | 0.2619793 |
| PAK2 | NM_002577 | 25.25522518 | 46.87932851 | 1.85623 | 1.85623 | 0.003811039 | 0.2823126 |
| PARP9 | NM_001146106 | 30.36989467 | 66.12848753 | 2.17743 | 2.17743 | 0.01816611 | 0.3527027 |
| CNBP | NM_001127192 | 56.96958533 | 102.5646666 | 1.80034 | 1.80034 | 0.02688756 | 0.3861136 |
| MRPL3 | NM_007208 | 63.05636061 | 153.9139301 | 2.44089 | 2.44089 | 0.001626832 | 0.2619793 |
| TM4SF1 | ENST00000305366 | 70.78589459 | 193.8974473 | 2.73922 | 2.73922 | 0.001107354 | 0.2619793 |
| GPR87 | NM_023915 | 24.03008756 | 48.91821986 | 2.0357 | 2.0357 | 0.01649662 | 0.341818 |
| TNFSF10 | NM_003810 | 23.89919302 | 41.96166445 | 1.75578 | 1.75578 | 0.01355 | 0.3309165 |
| TBL1XR1 | ENST00000430069 | 37.78678839 | 80.26504871 | 2.12416 | 2.12416 | 0.005392237 | 0.2875071 |
| MRPL47 | ENST00000476781 | 16.13162647 | 28.73505688 | 1.78129 | 1.78129 | 0.002497019 | 0.2730527 |
| DNAJC19 | NM_145261 | 11.50939425 | 21.90001572 | 1.9028 | 1.9028 | 0.002847129 | 0.2823126 |
| DCUN1D1 | ENST00000292782 | 93.31712791 | 185.7360437 | 1.99038 | 1.99038 | 0.01035599 | 0.3201187 |
| ABCC5 | NM_005688 | 46.45171639 | 94.852244 | 2.04194 | 2.04194 | 0.0256789 | 0.3837192 |
| ALG3 | NM_005787 | 99.25789816 | 185.3014037 | 1.86687 | 1.86687 | 0.000232337 | 0.2619793 |
| IGF2BP2 | NM_006548 | 43.07891473 | 79.68520602 | 1.84974 | 1.84974 | 0.01804539 | 0.3527027 |
| EIF4A2 | NM_001967 | 27.20839593 | 48.33847319 | 1.7766 | 1.7766 | 0.004568163 | 0.2829272 |
| RPL39L | ENST00000296277 | 25.95714848 | 55.82927824 | 2.15082 | 2.15082 | 0.005433237 | 0.2875071 |
| ACAP2 | NM_012287 | 27.08908953 | 49.57325209 | 1.83001 | 1.83001 | 0.005711973 | 0.2935743 |
| TFRC | NM_003234 | 24.945713 | 49.14967596 | 1.97027 | 1.97027 | 0.004331966 | 0.2829272 |
| DLG1 | NM_004087 | 39.91059826 | 77.49976335 | 1.94184 | 1.94184 | 0.0212529 | 0.3677429 |
| UCHL1 | NM_004181 | 25.16069729 | 51.49891028 | 2.04679 | 2.04679 | 0.03537081 | 0.4146834 |
| SPP1 | NM_001251830 | 26.69726412 | 93.46925566 | 3.50109 | 3.50109 | 0.04238981 | 0.444771 |
| OCIAD2 | ENST00000508632 | 33.21516095 | 61.31210365 | 1.8459 | 1.8459 | 7.72E-05 | 0.2619793 |
| CXCL9 | ENST00000264888 | 14.33748429 | 28.61064268 | 1.99551 | 1.99551 | 0.02286866 | 0.3719904 |
| CCT5 | ENST00000280326 | 50.15042424 | 88.69310201 | 1.76854 | 1.76854 | 0.006100042 | 0.2961425 |
| HINT1 | ENST00000304043 | 28.31118046 | 56.84296877 | 2.00778 | 2.00778 | 0.006176781 | 0.2965765 |
| DSP | ENST00000379802 | 77.10860699 | 154.9329298 | 2.00928 | 2.00928 | 0.02617127 | 0.3851257 |
| TUBB | ENST00000421473 | 162.0988449 | 298.7262071 | 1.84286 | 1.84286 | 0.04915107 | 0.4627806 |
| RPF2 | ENST00000441448 | 9.787022389 | 25.26100268 | 2.58107 | 2.58107 | 0.0142726 | 0.334671 |
| ABRACL | NM_021243 | 9.395500394 | 19.12091954 | 2.03511 | 2.03511 | 0.008171133 | 0.30416 |
| TPMT | NM_000367 | 9.874378864 | 18.57332236 | 1.88097 | 1.88097 | 0.0116028 | 0.3237456 |
| HIST1H2BK | NM_080593 | 42.38288823 | 117.7636106 | 2.77857 | 2.77857 | 0.02677498 | 0.3861136 |
| LSM2 | NM_021177 | 18.64374784 | 43.14914298 | 2.3144 | 2.3144 | 0.002769382 | 0.2823126 |
| SGK1 | NM_001143676 | 58.62076946 | 112.5825952 | 1.92052 | 1.92052 | 0.01969914 | 0.3593679 |
| PERP | NM_022121 | 285.3858511 | 630.2541926 | 2.20843 | 2.20843 | 0.02198203 | 0.3700809 |
| TCP1 | NM_030752 | 38.78427693 | 83.1555125 | 2.14405 | 2.14405 | 0.000598356 | 0.2619793 |
| AKR1B10 | NM_020299 | 29.67014351 | 95.65377494 | 3.22389 | 3.22389 | 0.03185753 | 0.4031813 |
| SAMD9 | NM_017654 | 21.42405998 | 44.53136717 | 2.07857 | 2.07857 | 0.02006808 | 0.3623759 |
| NAMPT | NM_005746 | 481.4192432 | 1033.268873 | 2.14628 | 2.14628 | 0.01269003 | 0.3293106 |
| FAM91A1 | DQ228141 | 13.25155723 | 26.65972512 | 2.01182 | 2.01182 | 0.009212846 | 0.3074379 |
| C8orf59 | ENST00000518786 | 48.56011725 | 109.06996 | 2.24608 | 2.24608 | 0.01178725 | 0.3246917 |
| ANXA2P2 | NR_003573 | 7.535848701 | 13.21138672 | 1.75314 | 1.75314 | 0.006996281 | 0.30416 |
| NTRK2 | NM_006180 | 24.1501459 | 58.17389512 | 2.40884 | 2.40884 | 0.003410871 | 0.2823126 |
| ANP32B | NM_006401 | 49.56775454 | 89.13684546 | 1.79829 | 1.79829 | 0.001860904 | 0.2619793 |
| SLC44A1 | NM_080546 | 18.75001702 | 38.70210148 | 2.06412 | 2.06412 | 0.001111655 | 0.2619793 |
| IFNA17 | NM_021268 | 7.501816635 | 15.17253244 | 2.02252 | 2.02252 | 0.02205677 | 0.3700809 |
| AKR1C1 | ENST00000434459 | 83.64920941 | 262.8817173 | 3.14267 | 3.14267 | 0.002414007 | 0.2724029 |
| AKR1C3 | ENST00000380554 | 26.76600652 | 56.01417275 | 2.09274 | 2.09274 | 0.007812582 | 0.30416 |
| SCD | NM_005063 | 39.66872161 | 123.3949891 | 3.11064 | 3.11064 | 0.007736683 | 0.30416 |
| AKR1C2 | NM_001135241 | 90.62329236 | 291.4773562 | 3.21636 | 3.21636 | 0.01253611 | 0.3293106 |
| SLC25A16 | ENST00000265870 | 12.26980146 | 21.76970571 | 1.77425 | 1.77425 | 0.01007901 | 0.3201187 |
| CFL1 | ENST00000525451 | 152.1436172 | 295.859826 | 1.94461 | 1.94461 | 0.000167892 | 0.2619793 |
| TPI1 | NM_001258026 | 27.14397309 | 56.03514275 | 2.06436 | 2.06436 | 0.04130852 | 0.4412572 |
| HNRNPA1 | ENST00000546500 | 22.55178932 | 42.31625356 | 1.8764 | 1.8764 | 0.002384542 | 0.2724029 |
| RAP1B | ENST00000250559 | 231.2853378 | 430.9270944 | 1.86319 | 1.86319 | 0.02480946 | 0.3788541 |
| TAS2R31 | NM_176885 | 19.99462059 | 39.38378872 | 1.96973 | 1.96973 | 0.001305032 | 0.2619793 |
| TUBA1B | NM_006082 | 209.9264293 | 606.6245186 | 2.88971 | 2.88971 | 0.02083749 | 0.3654614 |
| KRT5 | ENST00000252242 | 144.0564054 | 268.6883576 | 1.86515 | 1.86515 | 0.003930079 | 0.2823126 |
| KRT6C | NM_173086 | 36.87548195 | 160.0945072 | 4.34148 | 4.34148 | 0.004266328 | 0.2829272 |
| KRT6B | NM_005555 | 432.8819995 | 3538.200283 | 8.17336 | 8.17336 | 0.00325098 | 0.2823126 |
| LUM | ENST00000266718 | 18.7775899 | 34.16861819 | 1.81965 | 1.81965 | 0.04476018 | 0.451367 |
| GJB6 | NM_001110219 | 26.1585379 | 65.65850518 | 2.51002 | 2.51002 | 0.009322472 | 0.3088629 |
| SLIRP | NR_052025 | 39.09846328 | 70.71772703 | 1.8087 | 1.8087 | 0.01150619 | 0.3237456 |
| GPX2 | ENST00000389614 | 58.44306759 | 152.6559488 | 2.61203 | 2.61203 | 0.02761211 | 0.3877955 |
| LGMN | NM_005606 | 43.27164315 | 76.6173913 | 1.77061 | 1.77061 | 0.03679774 | 0.4208782 |
| C14orf2 | NM_001127393 | 69.32577816 | 129.0995355 | 1.86221 | 1.86221 | 0.002079659 | 0.2641199 |
| NUSAP1 | NM_016359 | 12.83605526 | 23.8059451 | 1.85462 | 1.85462 | 0.00358342 | 0.2823126 |
| MAPK6 | NM_002748 | 30.86992344 | 67.02465579 | 2.17119 | 2.17119 | 0.008764476 | 0.30416 |
| NOP10 | NM_018648 | 54.59890074 | 104.4913712 | 1.9138 | 1.9138 | 0.02697194 | 0.3861136 |
| ARPP19 | ENST00000566423 | 37.70724914 | 68.15936548 | 1.8076 | 1.8076 | 0.02409371 | 0.3782574 |
| FOPNL | NM_144600 | 13.58130305 | 23.90002132 | 1.75978 | 1.75978 | 0.00176739 | 0.2619793 |
| SLC7A5 | NM_003486 | 27.42006337 | 48.44648196 | 1.76682 | 1.76682 | 0.004601535 | 0.2829272 |
| TMEM97 | NM_014573 | 20.09855672 | 40.7288733 | 2.02647 | 2.02647 | 0.007587395 | 0.30416 |
| RPL41 | NM_021104 | 10.84967558 | 19.87289295 | 1.83167 | 1.83167 | 0.01210128 | 0.3270424 |
| PSMB3 | ENST00000225426 | 15.93094563 | 29.02411154 | 1.82187 | 1.82187 | 0.01851153 | 0.3544509 |
| NME2 | ENST00000393190 | 74.15643581 | 136.907721 | 1.8462 | 1.8462 | 0.000474127 | 0.2619793 |
| TOP2A | NM_001067 | 13.27536847 | 24.91685365 | 1.87693 | 1.87693 | 0.004289963 | 0.2829272 |
| KRT13 | ENST00000246635 | 156.2974033 | 595.439038 | 3.80965 | 3.80965 | 0.01115352 | 0.3221695 |
| KRT16 | NM_005557 | 59.09073341 | 207.1827386 | 3.50617 | 3.50617 | 0.008129287 | 0.30416 |
| CLTC | NM_004859 | 41.75276976 | 107.4692416 | 2.57394 | 2.57394 | 9.02E-05 | 0.2619793 |
| ACTG1 | ENST00000575842 | 54.81884399 | 105.0221627 | 1.91582 | 1.91582 | 0.0020143 | 0.2619793 |
| FOXK2 | NM_004514 | 36.41598592 | 70.89392003 | 1.94679 | 1.94679 | 0.000596577 | 0.2619793 |
| DSG3 | NM_001944 | 31.37557514 | 98.23123962 | 3.1308 | 3.1308 | 0.003854641 | 0.2823126 |
| DSG2 | NM_001943 | 23.17664765 | 45.81378964 | 1.97673 | 1.97673 | 0.002021851 | 0.2619793 |
| SERPINB13 | NM_012397 | 57.39010067 | 141.3844847 | 2.46356 | 2.46356 | 0.01920061 | 0.356823 |
| DSC3 | NM_024423 | 28.79846478 | 64.03683057 | 2.22363 | 2.22363 | 0.004185392 | 0.2829272 |
| DSC2 | NM_024422 | 25.98054879 | 60.05198377 | 2.31142 | 2.31142 | 0.005157119 | 0.2847931 |
| GPI | NM_000175 | 82.91550487 | 169.2345123 | 2.04106 | 2.04106 | 0.006081211 | 0.2961425 |
| UBA2 | NM_005499 | 28.74800626 | 55.405604 | 1.92729 | 1.92729 | 0.01094131 | 0.3221695 |
| COX6B1 | NM_001863 | 52.6072598 | 158.8465559 | 3.01949 | 3.01949 | 0.01515727 | 0.3385777 |
| SPINT2 | NM_021102 | 72.19915264 | 128.8760168 | 1.785 | 1.785 | 0.002638869 | 0.2823126 |
| CYP2A7 | NM_000764 | 6.856869191 | 20.31776773 | 2.96313 | 2.96313 | 0.000427768 | 0.2619793 |
| PI3 | ENST00000243924 | 40.45230484 | 129.9542495 | 3.21252 | 3.21252 | 0.02689548 | 0.3861136 |
| SLMO2 | NM_016045 | 35.04022175 | 82.87183716 | 2.36505 | 2.36505 | 0.000486609 | 0.2619793 |
| MIF | ENST00000215754 | 74.33192163 | 134.5118999 | 1.80961 | 1.80961 | 0.000252873 | 0.2619793 |
| DDTL | NM_001084393 | 46.21662485 | 91.88516182 | 1.98813 | 1.98813 | 0.007825882 | 0.30416 |
| WBP5 | NM_001006613 | 38.09025391 | 69.96201809 | 1.83674 | 1.83674 | 0.01311118 | 0.3294062 |
| FAM223A | AY168775 | 25.7046952 | 145.4026749 | 5.65664 | 5.65664 | 0.01930108 | 0.3574549 |
| DYNLT3 | NM_006520 | 48.94467489 | 130.509408 | 2.66648 | 2.66648 | 0.03088599 | 0.3979765 |
| TCEAL3 | NM_001006933 | 7.652228744 | 15.69257284 | 2.05073 | 2.05073 | 0.04466814 | 0.4511603 |
| GABRE | NM_004961 | 36.27540962 | 64.11677694 | 1.7675 | 1.7675 | 0.003938537 | 0.2823126 |
| ATP2B3 | NM_021949 | 28.82942185 | 55.70210719 | 1.93212 | 1.93212 | 0.004238483 | 0.2829272 |
| S100A16 | ENST00000368703 | 31.41322156 | 61.58555078 | 1.9605 | 1.9605 | 0.02242101 | 0.3702301 |
| C1orf158 | NM_152290 | 41.397737 | 21.88635804 | 0.528686 | -1.89148 | 0.009972743 | 0.3201187 |
| CCDC30 | ENST00000428554 | 19.2931927 | 9.91745537 | 0.514039 | -1.94538 | 0.007057291 | 0.30416 |
| TSPAN1 | NM_005727 | 196.0027383 | 81.07807889 | 0.41366 | -2.41745 | 0.02016922 | 0.3623759 |
| WDR63 | NM_145172 | 16.29886954 | 8.952185928 | 0.549252 | -1.82066 | 0.005613832 | 0.2899184 |
| CYR61 | NM_001554 | 65.56527387 | 34.99992681 | 0.533818 | -1.8733 | 0.000517887 | 0.2619793 |
| C1orf88 | ENST00000369738 | 27.86495658 | 13.53365864 | 0.485689 | -2.05893 | 0.01111452 | 0.3221695 |
| LCE2D | NM_178430 | 33.15512525 | 16.23696404 | 0.489725 | -2.04196 | 0.003793019 | 0.2823126 |
| BEST4 | NM_153274 | 29.40561992 | 14.52231957 | 0.493863 | -2.02485 | 0.001164414 | 0.2619793 |
| HIST2H2BF | NM_001024599 | 38.35014161 | 20.61267733 | 0.537488 | -1.86051 | 0.007446104 | 0.30416 |
| APOBEC4 | NM_203454 | 30.83079102 | 12.68762385 | 0.411522 | -2.43 | 5.49E-05 | 0.2619793 |
| PIGR | NM_002644 | 155.1844294 | 81.67541641 | 0.526311 | -1.90002 | 0.01939013 | 0.3575232 |
| ACTG2 | ENST00000409624 | 55.1183196 | 20.82392333 | 0.377805 | -2.64687 | 0.04189392 | 0.4417732 |
| DNAH6 | NM_001370 | 23.89803345 | 11.92064175 | 0.498813 | -2.00476 | 0.004896728 | 0.2829272 |
| DNAH6 | BC015442 | 15.08517994 | 8.404837928 | 0.557162 | -1.79481 | 0.00297221 | 0.2823126 |
| C2orf40 | ENST00000238044 | 25.47270031 | 13.82550238 | 0.542758 | -1.84244 | 0.006451022 | 0.301055 |
| TUBA4B | NR_003063 | 60.07613105 | 27.00453872 | 0.449506 | -2.22467 | 0.001317099 | 0.2619793 |
| DES | NM_001927 | 70.4535257 | 37.84209354 | 0.537118 | -1.86179 | 0.04155679 | 0.4413006 |
| NR4A2 | ENST00000409572 | 52.62440094 | 25.12653797 | 0.477467 | -2.09439 | 0.01821761 | 0.3527027 |
| DYTN | NM_001093730 | 17.93753821 | 8.920286209 | 0.497296 | -2.01087 | 0.003786316 | 0.2823126 |
| CCDC108 | NM_194302 | 97.80796155 | 34.14020935 | 0.349053 | -2.8649 | 0.000510634 | 0.2619793 |
| SATB1 | NM_001195470 | 73.80824084 | 37.86728285 | 0.51305 | -1.94913 | 0.0203004 | 0.3623759 |
| DNAH12 | NM_198564 | 33.71503055 | 14.82201824 | 0.439626 | -2.27466 | 0.001124756 | 0.2619793 |
| ABI3BP | NM_015429 | 50.83710602 | 26.17758313 | 0.514931 | -1.94201 | 0.000258446 | 0.2619793 |
| ZBBX | ENST00000307529 | 24.60961188 | 10.2230941 | 0.415409 | -2.40726 | 0.001630688 | 0.2619793 |
| SPATA18 | NM_145263 | 36.56166839 | 20.15771214 | 0.551333 | -1.81379 | 0.000381882 | 0.2619793 |
| SPARCL1 | NM_001128310 | 61.08304172 | 31.46901245 | 0.515184 | -1.94105 | 0.007366322 | 0.30416 |
| EGR1 | NM_001964 | 522.1208641 | 270.2348633 | 0.51757 | -1.93211 | 0.01426816 | 0.334671 |
| NME5 | NM_003551 | 25.87631006 | 13.0455874 | 0.504152 | -1.98353 | 0.003716718 | 0.2823126 |
| DUSP1 | NM_004417 | 112.7333061 | 47.83550753 | 0.424323 | -2.3567 | 0.001790884 | 0.2619793 |
| HIST1H2BE | NM_003523 | 8.884619552 | 4.151146938 | 0.467228 | -2.14028 | 0.02013604 | 0.3623759 |
| C6orf165 | ENST00000369562 | 14.02092863 | 7.998613826 | 0.570479 | -1.75291 | 0.003246455 | 0.2823126 |
| PLN | ENST00000357525 | 43.79699555 | 17.2735176 | 0.394398 | -2.53551 | 0.0293358 | 0.3917696 |
| SLC44A4 | ENST00000453831 | 90.10592064 | 47.91880415 | 0.531807 | -1.88038 | 0.006440017 | 0.301055 |
| GSTA2 | NM_000846 | 41.99891056 | 14.73086252 | 0.350744 | -2.85108 | 0.007808262 | 0.30416 |
| AKD1 | ENST00000285397 | 17.9973177 | 10.09535683 | 0.560939 | -1.78273 | 0.003663228 | 0.2823126 |
| CTGF | ENST00000367976 | 93.25828539 | 45.41478043 | 0.486978 | -2.05348 | 0.000352774 | 0.2619793 |
| CDCA7L | NM_018719 | 19.6974914 | 10.98787121 | 0.557832 | -1.79265 | 0.002413417 | 0.2724029 |
| SPDYE1 | NM_175064 | 6.212612191 | 3.17745127 | 0.511451 | -1.95522 | 0.002174634 | 0.2654719 |
| ZNF273 | NR_003099 | 49.39660553 | 24.0944673 | 0.487776 | -2.05012 | 0.01887387 | 0.3565552 |
| PVRIG | NM_024070 | 72.36950507 | 40.8600765 | 0.564602 | -1.77116 | 0.00308457 | 0.2823126 |
| CDHR3 | NM_152750 | 67.56012068 | 23.42764446 | 0.346766 | -2.88379 | 0.00136992 | 0.2619793 |
| IQUB | ENST00000466202 | 10.11342671 | 5.544702289 | 0.548251 | -1.82398 | 0.004153064 | 0.2829272 |
| RP1 | NM_006269 | 14.86739519 | 7.786527048 | 0.523732 | -1.90938 | 0.000266389 | 0.2619793 |
| EGR3 | NM_004430 | 31.29781421 | 17.60532555 | 0.562512 | -1.77774 | 0.00923514 | 0.3074379 |
| DNAI1 | NM_012144 | 32.73336467 | 16.93715905 | 0.517427 | -1.93264 | 0.00109453 | 0.2619793 |
| NR4A3 | NM_173200 | 35.54488347 | 17.53821481 | 0.493407 | -2.02672 | 0.01794344 | 0.3527027 |
| AQP3 | NM_004925 | 254.2827878 | 128.8536862 | 0.506734 | -1.97342 | 0.03474017 | 0.411593 |
| TPM2 | ENST00000378292 | 74.43297527 | 39.55861469 | 0.531463 | -1.8816 | 0.03654424 | 0.4187383 |
| SPAG8 | NM_172312 | 15.96820232 | 8.218015206 | 0.514648 | -1.94307 | 0.000618916 | 0.2619793 |
| ZNF658 | NM_033160 | 7.305383449 | 3.427864304 | 0.469227 | -2.13117 | 0.009756852 | 0.3155183 |
| MSMB | NM_002443 | 489.3863324 | 95.50471138 | 0.195152 | -5.12421 | 0.01253568 | 0.3293106 |
| PLEKHS1 | ENST00000369312 | 58.24046625 | 29.43478123 | 0.505402 | -1.97862 | 0.03389317 | 0.4102268 |
| ENKUR | NM_145010 | 21.09662043 | 11.40962919 | 0.540828 | -1.84902 | 0.001027419 | 0.2619793 |
| TTC18 | AF435959 | 17.21530696 | 9.578833654 | 0.556416 | -1.79722 | 0.001052875 | 0.2619793 |
| ACTA2 | NM_001141945 | 103.7530545 | 38.87309307 | 0.374671 | -2.66901 | 0.01332611 | 0.3301055 |
| SORBS1 | ENST00000371247 | 33.68372999 | 19.1254263 | 0.567791 | -1.76121 | 0.02585281 | 0.3847028 |
| WDR96 | NM_025145 | 23.70385827 | 10.52960929 | 0.444215 | -2.25116 | 0.000419738 | 0.2619793 |
| NUCB2 | NM_005013 | 41.45602829 | 22.28730083 | 0.537614 | -1.86007 | 0.0111795 | 0.3221695 |
| MS4A8B | ENST00000450141 | 82.02205587 | 34.00228982 | 0.414549 | -2.41226 | 0.0084924 | 0.30416 |
| SCGB1A1 | NM_003357 | 896.1894917 | 237.6805437 | 0.265213 | -3.77055 | 0.02165551 | 0.3695205 |
| DCDC1 | NM_181807 | 26.41215294 | 14.13411846 | 0.535139 | -1.86867 | 0.003351574 | 0.2823126 |
| AGBL2 | NM_024783 | 23.65953804 | 13.3937682 | 0.566105 | -1.76646 | 0.007762012 | 0.30416 |
| LRRC23 | NM_001135217 | 61.29680617 | 30.27174634 | 0.493857 | -2.02488 | 0.003532507 | 0.2823126 |
| NR4A1 | NM_002135 | 97.10071407 | 46.3275987 | 0.47711 | -2.09595 | 0.007557567 | 0.30416 |
| LRRIQ1 | NM_001079910 | 20.19211322 | 9.920205453 | 0.491291 | -2.03545 | 0.001757835 | 0.2619793 |
| PLEKHG7 | NM_001004330 | 20.27275142 | 11.5276777 | 0.56863 | -1.75861 | 0.001971431 | 0.2619793 |
| C12orf63 | ENST00000342887 | 16.03197212 | 9.029336635 | 0.563208 | -1.77554 | 0.009490111 | 0.3106091 |
| DNAH10 | BC144575 | 18.93416639 | 10.63781539 | 0.561831 | -1.77989 | 0.003246219 | 0.2823126 |
| A2M | ENST00000318602 | 101.438394 | 37.53071838 | 0.369986 | -2.7028 | 0.00031309 | 0.2619793 |
| MGP | NM_001190839 | 68.34623685 | 33.79153542 | 0.494415 | -2.02259 | 0.007517912 | 0.30416 |
| CASC1 | NM_001082972 | 13.45490221 | 6.849601231 | 0.509079 | -1.96433 | 0.000937621 | 0.2619793 |
| TUBA1A | NM_001270399 | 134.5464019 | 71.01786085 | 0.527831 | -1.89455 | 0.01079216 | 0.3221695 |
| TSPAN8 | ENST00000393330 | 96.17099898 | 21.74783677 | 0.226138 | -4.42208 | 0.02663798 | 0.3859483 |
| DCN | NM_133506 | 86.57688567 | 45.2479335 | 0.522632 | -1.91339 | 0.04629878 | 0.4560034 |
| FAM216B | NM_182508 | 23.15994625 | 12.04489573 | 0.520073 | -1.92281 | 0.0218507 | 0.3700809 |
| SLITRK6 | NM_032229 | 26.34212817 | 13.7536249 | 0.522114 | -1.91529 | 0.006567556 | 0.3022413 |
| FOS | NM_005252 | 175.7498929 | 67.35625398 | 0.383251 | -2.60926 | 0.007917785 | 0.30416 |
| TC2N | ENST00000435962 | 48.09416616 | 27.40524258 | 0.569827 | -1.75492 | 0.03421105 | 0.411593 |
| DNAAF1 | NM_178452 | 94.7753517 | 35.01084556 | 0.369409 | -2.70703 | 0.000401375 | 0.2619793 |
| NDE1 | NM_001143979 | 61.4985291 | 22.56727002 | 0.366957 | -2.72512 | 0.01481841 | 0.3373427 |
| DNAH3 | ENST00000544558 | 18.17823218 | 9.858990971 | 0.542348 | -1.84383 | 0.007335075 | 0.30416 |
| DNAH3 | NM_017539 | 28.37089976 | 14.72116559 | 0.518885 | -1.92721 | 0.000659339 | 0.2619793 |
| TPPP3 | ENST00000562206 | 99.53555111 | 56.04524221 | 0.563065 | -1.776 | 0.000122733 | 0.2619793 |
| HYDIN | NM_032821 | 39.69402624 | 19.87647473 | 0.500743 | -1.99703 | 0.001655537 | 0.2619793 |
| DNAH9 | ENST00000262442 | 28.9742618 | 16.50544351 | 0.569659 | -1.75544 | 0.001457919 | 0.2619793 |
| LRRC46 | NM_033413 | 33.40749723 | 18.3731871 | 0.549975 | -1.81827 | 0.001202115 | 0.2619793 |
| ALOX15 | NM_001140 | 53.95818815 | 30.20865403 | 0.559852 | -1.78619 | 0.000348258 | 0.2619793 |
| TEKT1 | ENST00000338694 | 43.78758565 | 23.07102186 | 0.526884 | -1.89795 | 0.004806369 | 0.2829272 |
| MFAP4 | NM_001198695 | 47.45538825 | 25.09155541 | 0.528739 | -1.89129 | 0.01572066 | 0.3413743 |
| SLFN13 | NM_144682 | 32.91560933 | 18.74235064 | 0.569405 | -1.75622 | 0.001760662 | 0.2619793 |
| CCL15 | ENST00000495214 | 56.01960866 | 27.34376504 | 0.48811 | -2.04872 | 0.000845338 | 0.2619793 |
| SERPINB11 | ENST00000544088 | 35.10586084 | 15.82561873 | 0.450797 | -2.2183 | 0.04109898 | 0.4405255 |
| FOSB | NM_006732 | 81.85904893 | 42.66999392 | 0.521262 | -1.91842 | 0.04725579 | 0.4582142 |
| BPIFB1 | NM_033197 | 873.6247993 | 202.7601042 | 0.23209 | -4.30867 | 0.02418807 | 0.3787958 |
| MYL9 | NM_006097 | 129.0261787 | 69.63110437 | 0.539666 | -1.853 | 0.0328009 | 0.4068991 |
| C20orf85 | ENST00000371168 | 39.28100674 | 15.33610272 | 0.390421 | -2.56134 | 0.00033377 | 0.2619793 |
| KCNE1 | ENST00000399286 | 48.58907289 | 26.92342415 | 0.554104 | -1.80472 | 0.01934239 | 0.3574549 |
| TFF3 | ENST00000518498 | 157.1305617 | 88.02308314 | 0.560191 | -1.7851 | 0.00883458 | 0.30416 |
| RPSA | NM_002295 | 22.5924684 | 11.50038297 | 0.509036 | -1.9645 | 0.02713885 | 0.3861136 |
| ZMAT1 | NR_046008 | 23.3822199 | 12.55082244 | 0.536769 | -1.863 | 0.00186804 | 0.2619793 |
| CDR1 | NM_004065 | 169.4598868 | 68.38983483 | 0.403574 | -2.47786 | 0.03496554 | 0.4125439 |
| APOD | NM_001647 | 170.1437091 | 50.99238812 | 0.299702 | -3.33664 | 0.00024407 | 0.2619793 |

Additional file 7. Table S7. Differential RNA expression in low-grade PSCC versus paired controls.

| **Gene symbol** | **RefSeq** | **Mean ISCC** | **Mean control** | **Ratio** | **Fold change** | **P-value** | **Q-value** |
| --- | --- | --- | --- | --- | --- | --- | --- |
| ACSM2B | ENST00000329697 | 34.95265157 | 17.90313086 | 0.512212 | -1.95232 | 0.003640623 | 0.2652425 |
| AKD1 | ENST00000285397 | 19.61166339 | 10.44912332 | 0.532803 | -1.87687 | 0.004213862 | 0.266425 |
| ARL6IP1 | ENST00000304414 | 37.98005264 | 67.22984881 | 1.77014 | 1.77014 | 0.01356279 | 0.309908 |
| AKR1B10 | NM_020299 | 25.21045061 | 56.24605474 | 2.23106 | 2.23106 | 0.000429712 | 0.199632 |
| AKR1C2 | NM_001135241 | 88.20447825 | 185.9949963 | 2.10869 | 2.10869 | 0.000361751 | 0.1958968 |
| A2M | ENST00000318602 | 122.244013 | 69.32385607 | 0.567094 | -1.76338 | 0.02350728 | 0.3435971 |
| ANXA1 | ENST00000257497 | 163.6318752 | 347.8351694 | 2.12571 | 2.12571 | 0.007231765 | 0.2814498 |
| MKI67 | ENST00000368654 | 17.84081974 | 31.95057523 | 1.79088 | 1.79088 | 0.01491251 | 0.3154484 |
| APOBEC4 | NM_203454 | 32.95464673 | 15.47234538 | 0.469506 | -2.1299 | 0.006661047 | 0.2756742 |
| APOD | NM_001647 | 195.9484024 | 95.72607159 | 0.488525 | -2.04698 | 0.007271736 | 0.2818056 |
| ARMC3 | NM_173081 | 16.68789407 | 9.284601563 | 0.556367 | -1.79737 | 0.008354489 | 0.2844976 |
| ABCC5 | NM_005688 | 42.51529373 | 74.94550254 | 1.76279 | 1.76279 | 0.008886931 | 0.286724 |
| AGBL2 | NM_024783 | 27.65368816 | 12.1967934 | 0.441052 | -2.2673 | 0.002618625 | 0.2583437 |
| BEST4 | NM_153274 | 30.34043784 | 15.82342498 | 0.521528 | -1.91744 | 0.002000091 | 0.2532133 |
| BPIFA1 | NM_130852 | 191.2107732 | 30.20614145 | 0.157973 | -6.3302 | 0.00050642 | 0.199632 |
| BPIFB1 | NM_033197 | 1034.057002 | 185.088314 | 0.178989 | -5.58694 | 7.62E-05 | 0.08506703 |
| CDHR3 | NM_152750 | 78.73482573 | 27.0787643 | 0.343923 | -2.90763 | 0.003283922 | 0.2583437 |
| CASC1 | NM_001082972 | 15.89344537 | 8.15248567 | 0.512944 | -1.94953 | 0.01243671 | 0.3022021 |
| CHST9 | NM_031422 | 51.91033885 | 29.56339397 | 0.56951 | -1.7559 | 0.01538777 | 0.3166662 |
| CDCA7L | NM_018719 | 19.88667257 | 10.96246248 | 0.551244 | -1.81408 | 0.005540857 | 0.2705934 |
| CDR1 | NM_004065 | 165.6207932 | 83.61906462 | 0.504882 | -1.98066 | 0.02539271 | 0.3520678 |
| CCL15 | ENST00000495214 | 68.44294817 | 32.99052885 | 0.482015 | -2.07462 | 0.001880379 | 0.2532133 |
| CXCL1 | NM_001511 | 419.1811407 | 238.5966538 | 0.569194 | -1.75687 | 0.002187413 | 0.2537682 |
| CLCA4 | NM_012128 | 13.6260925 | 24.09713961 | 1.76846 | 1.76846 | 0.005024199 | 0.2672274 |
| C1orf158 | NM_152290 | 42.6256521 | 23.66806734 | 0.555253 | -1.80098 | 0.01179643 | 0.294789 |
| C1orf168 | NM_001004303 | 18.02428343 | 9.566293137 | 0.530743 | -1.88415 | 0.000315199 | 0.189144 |
| C1orf173 | ENST00000326665 | 26.07165028 | 13.21376786 | 0.506824 | -1.97307 | 0.00051285 | 0.199632 |
| C1orf88 | ENST00000369738 | 36.61467284 | 14.9872107 | 0.409321 | -2.44307 | 0.003092668 | 0.2583437 |
| C12orf63 | ENST00000342887 | 16.84933794 | 9.268141047 | 0.550062 | -1.81798 | 0.01102187 | 0.2890686 |
| C2orf40 | ENST00000238044 | 28.49328392 | 13.84218705 | 0.485805 | -2.05844 | 0.000145447 | 0.1531503 |
| C20orf85 | ENST00000371168 | 58.95368136 | 23.02150108 | 0.390501 | -2.56081 | 0.004015135 | 0.266425 |
| C3orf15 | NM_033364 | 24.17979319 | 12.84789409 | 0.531348 | -1.88201 | 0.002060725 | 0.2532133 |
| C6orf165 | ENST00000369562 | 15.49509828 | 8.782794208 | 0.566812 | -1.76425 | 0.009984593 | 0.286724 |
| CCDC108 | NM_194302 | 121.6008919 | 30.0538935 | 0.247153 | -4.04607 | 1.53E-05 | 0.08506703 |
| CCDC146 | NM_020879 | 40.95194305 | 22.49776841 | 0.54937 | -1.82027 | 0.01721097 | 0.3191793 |
| CCDC19 | ENST00000368099 | 42.88288313 | 23.80363507 | 0.555087 | -1.80152 | 0.008644312 | 0.286724 |
| CCDC30 | ENST00000428554 | 20.79954408 | 11.30673121 | 0.543602 | -1.83958 | 0.01001324 | 0.286724 |
| CTGF | ENST00000367976 | 104.6102205 | 48.59614607 | 0.464546 | -2.15264 | 0.001203661 | 0.2497402 |
| CSTA | NM_005213 | 91.35741978 | 366.8534392 | 4.01557 | 4.01557 | 6.14E-05 | 0.08506703 |
| CYB5A | NM_148923 | 124.009772 | 64.72135917 | 0.521908 | -1.91604 | 0.007123667 | 0.2803794 |
| DLEC1 | ENST00000308059 | 46.67182575 | 24.38073983 | 0.522389 | -1.91428 | 0.002654774 | 0.2583437 |
| DSC2 | NM_024422 | 21.35852331 | 45.13328866 | 2.11313 | 2.11313 | 0.02224441 | 0.3386391 |
| DSC3 | NM_024423 | 32.1761543 | 81.23389977 | 2.52466 | 2.52466 | 0.003066486 | 0.2583437 |
| DSG3 | NM_001944 | 25.93394902 | 72.39860529 | 2.79166 | 2.79166 | 0.01897527 | 0.3275087 |
| DSP | ENST00000379802 | 79.13039205 | 178.1884481 | 2.25183 | 2.25183 | 0.002148577 | 0.2532133 |
| DCDC1 | NM_181807 | 25.90035559 | 14.40282592 | 0.556087 | -1.79828 | 0.005293442 | 0.2705934 |
| DYNLT3 | NM_006520 | 43.87903826 | 90.52221617 | 2.06299 | 2.06299 | 0.01759455 | 0.3199653 |
| DNAH6 | NM_001370 | 23.67233315 | 12.44461765 | 0.525706 | -1.90221 | 0.01782388 | 0.3202573 |
| DNAH6 | BC015442 | 18.37751762 | 8.922574239 | 0.485515 | -2.05967 | 0.008260054 | 0.2841299 |
| DNAH12 | NM_198564 | 38.07283248 | 15.24854834 | 0.400508 | -2.49683 | 0.002478475 | 0.2581064 |
| DNAH5 | NM_001369 | 24.60534773 | 12.93125148 | 0.525545 | -1.90279 | 0.006855265 | 0.2764471 |
| DNAI1 | NM_012144 | 40.0100354 | 17.92026417 | 0.447894 | -2.23267 | 0.002386772 | 0.2581064 |
| DNAH10 | BC144575 | 20.52314609 | 11.38766458 | 0.554869 | -1.80223 | 0.00924562 | 0.286724 |
| DYNLRB2 | NM_130897 | 26.75543352 | 13.70452101 | 0.512215 | -1.9523 | 0.00179995 | 0.2532133 |
| DNAAF1 | NM_178452 | 105.4985968 | 37.49015798 | 0.355362 | -2.81403 | 0.001710898 | 0.2532133 |
| DNAH3 | ENST00000544558 | 17.92423946 | 8.040582382 | 0.448587 | -2.22922 | 0.000694102 | 0.2055554 |
| DNAH3 | NM_017539 | 29.58061208 | 15.83461626 | 0.535305 | -1.86809 | 0.009108611 | 0.286724 |
| DNAH9 | ENST00000262442 | 31.8159876 | 17.31967556 | 0.544371 | -1.83698 | 0.003434306 | 0.2583437 |
| DYTN | NM_001093730 | 18.28108762 | 9.882869566 | 0.540607 | -1.84977 | 0.004473789 | 0.266425 |
| EFCAB1 | NM_024593 | 16.51265273 | 8.788457659 | 0.532227 | -1.8789 | 0.00318317 | 0.2583437 |
| EFHC1 | NR_033327 | 38.19839193 | 19.55221298 | 0.51186 | -1.95366 | 0.01087153 | 0.2881671 |
| ENKUR | NM_145010 | 25.01584014 | 12.8732998 | 0.514605 | -1.94324 | 0.01700689 | 0.3191793 |
| EMP1 | NM_001423 | 33.27369086 | 58.63783773 | 1.76229 | 1.76229 | 0.01626947 | 0.3187478 |
| EMP2 | ENST00000342147 | 84.90278659 | 154.3294948 | 1.81772 | 1.81772 | 0.002336422 | 0.255971 |
| FAM216B | NM_182508 | 29.15052578 | 12.97605559 | 0.44514 | -2.24648 | 0.01168975 | 0.294789 |
| FGFBP1 | NM_005130 | 119.3074233 | 275.3971845 | 2.3083 | 2.3083 | 0.003892485 | 0.266425 |
| FLRT3 | NM_198391 | 23.64707767 | 13.0785437 | 0.553071 | -1.80809 | 0.006235836 | 0.2750615 |
| GJB2 | NM_004004 | 24.63265107 | 46.3638993 | 1.88221 | 1.88221 | 0.000579305 | 0.199632 |
| GJB6 | NM_001110219 | 20.24256212 | 49.29707012 | 2.43532 | 2.43532 | 0.000574339 | 0.199632 |
| GPX2 | ENST00000389614 | 63.78918761 | 146.5582122 | 2.29754 | 2.29754 | 0.00050687 | 0.199632 |
| GSTA1 | NM_145740 | 114.9976046 | 62.61038207 | 0.54445 | -1.83672 | 0.04060387 | 0.3987455 |
| GSTA2 | NM_000846 | 67.98714111 | 13.63166612 | 0.200504 | -4.98743 | 0.000455285 | 0.199632 |
| GPNMB | NM_001005340 | 58.83611165 | 120.1397703 | 2.04194 | 2.04194 | 0.03854429 | 0.3933995 |
| GOLGA8A | NR_027409 | 61.09870936 | 34.41916039 | 0.563339 | -1.77513 | 0.02185185 | 0.3372177 |
| GBP6 | NM_198460 | 20.97981426 | 39.72457831 | 1.89347 | 1.89347 | 0.007104175 | 0.2803794 |
| HBB | ENST00000335295 | 61.69963288 | 28.80385491 | 0.46684 | -2.14206 | 0.04085158 | 0.3989046 |
| HIST1H3C | NM_003531 | 27.62284478 | 61.58555078 | 2.22951 | 2.22951 | 0.0212257 | 0.3354459 |
| HIST1H3F | NM_021018 | 26.63349768 | 49.05505823 | 1.84186 | 1.84186 | 0.01170658 | 0.294789 |
| HIST1H2BK | NM_080593 | 44.33364263 | 96.8385787 | 2.18431 | 2.18431 | 0.03693015 | 0.3894378 |
| HYDIN | NM_032821 | 46.73721942 | 23.04337295 | 0.493043 | -2.02822 | 0.002835293 | 0.2583437 |
| ITGA6 | NM_000210 | 30.43416755 | 56.34204407 | 1.85127 | 1.85127 | 0.0211319 | 0.3354459 |
| IL1RN | ENST00000409930 | 21.34061725 | 37.79097932 | 1.77084 | 1.77084 | 5.11E-05 | 0.08506703 |
| IL8 | NM_000584 | 84.65773588 | 45.5692904 | 0.538277 | -1.85778 | 0.007394386 | 0.2818056 |
| IVL | NM_005547 | 19.83628564 | 35.6233182 | 1.79587 | 1.79587 | 0.04669772 | 0.4115077 |
| IQUB | ENST00000466202 | 10.0720818 | 5.710313687 | 0.566947 | -1.76383 | 0.01086616 | 0.2881671 |
| JUP | NM_002230 | 95.12416635 | 175.3191768 | 1.84307 | 1.84307 | 0.004618824 | 0.266425 |
| KRT10 | ENST00000269576 | 27.10449082 | 57.90596237 | 2.13639 | 2.13639 | 0.001412047 | 0.2497402 |
| KRT13 | ENST00000246635 | 126.9274238 | 888.1687493 | 6.99744 | 6.99744 | 1.40E-05 | 0.08506703 |
| KRT16 | NM_005557 | 51.40048261 | 141.9914254 | 2.76246 | 2.76246 | 0.01028304 | 0.286724 |
| KRT4 | ENST00000551956 | 46.17627847 | 97.05630287 | 2.10185 | 2.10185 | 0.006883633 | 0.2770017 |
| KRT5 | ENST00000252242 | 181.2704561 | 358.2765975 | 1.97648 | 1.97648 | 0.000537852 | 0.199632 |
| KRT6B | NM_005555 | 330.8560843 | 2288.997227 | 6.91835 | 6.91835 | 0.000329322 | 0.189144 |
| KRT6C | NM_173086 | 30.15300749 | 109.6825342 | 3.63753 | 3.63753 | 0.006269427 | 0.2750615 |
| LRRC23 | NM_001135217 | 77.49224309 | 33.16385933 | 0.427965 | -2.33664 | 0.002196699 | 0.2537682 |
| LRRC46 | NM_033413 | 37.49587538 | 19.57146709 | 0.521964 | -1.91584 | 0.008811014 | 0.286724 |
| LRRIQ1 | NM_001079910 | 22.19111111 | 9.799512631 | 0.441599 | -2.2645 | 0.00860195 | 0.286724 |
| LYPD3 | NM_014400 | 51.34457691 | 92.5037534 | 1.80162 | 1.80162 | 0.00107629 | 0.2436402 |
| MALL | NM_005434 | 84.19251072 | 157.1893866 | 1.86703 | 1.86703 | 0.002023141 | 0.2532133 |
| MAL2 | NM_052886 | 114.866954 | 203.4414726 | 1.7711 | 1.7711 | 0.006616579 | 0.2756742 |
| MGP | NM_001190839 | 83.53043233 | 32.73994516 | 0.391953 | -2.55133 | 0.000735664 | 0.2112619 |
| MNS1 | NM_018365 | 17.04905653 | 9.312057716 | 0.54619 | -1.83086 | 0.003484353 | 0.2600004 |
| MS4A8B | ENST00000450141 | 92.5480059 | 31.42912059 | 0.339598 | -2.94466 | 0.001537268 | 0.2497402 |
| MFAP4 | NM_001198695 | 51.67268695 | 25.19560162 | 0.4876 | -2.05086 | 0.002728711 | 0.2583437 |
| MSMB | NM_002443 | 780.4919813 | 103.5432718 | 0.132663 | -7.53787 | 4.29E-05 | 0.08506703 |
| MRPS17 | ENST00000285298 | 11.05011899 | 21.70627106 | 1.96436 | 1.96436 | 0.02118527 | 0.3354459 |
| NEK10 | NM_199347 | 25.82577969 | 14.75405895 | 0.571292 | -1.75042 | 0.01173069 | 0.294789 |
| NEK5 | NM_199289 | 23.37557584 | 12.43177161 | 0.531825 | -1.88032 | 0.001709363 | 0.2532133 |
| PI3 | ENST00000243924 | 39.72320159 | 149.1747966 | 3.75535 | 3.75535 | 0.002558075 | 0.2583437 |
| PICALM | NM_007166 | 28.56546283 | 59.54217429 | 2.08441 | 2.08441 | 0.005132119 | 0.2672274 |
| PIGX | NM_001166304 | 11.16297763 | 20.72743945 | 1.85681 | 1.85681 | 0.007617338 | 0.2818056 |
| PLA2G10 | NM_003561 | 122.0120647 | 50.18415435 | 0.411305 | -2.43129 | 0.02663041 | 0.3551024 |
| PIH1D2 | ENST00000532211 | 18.26145737 | 8.669507267 | 0.474745 | -2.10639 | 0.003407804 | 0.2583437 |
| PKP1 | ENST00000263946 | 39.41191648 | 71.50290741 | 1.81425 | 1.81425 | 0.01046066 | 0.286724 |
| PLEKHS1 | ENST00000369312 | 76.60836362 | 25.91903319 | 0.33833 | -2.9557 | 0.00182865 | 0.2532133 |
| PLEKHH1 | NM_020715 | 32.5419771 | 18.02890661 | 0.554017 | -1.805 | 0.007396245 | 0.2818056 |
| PIGR | NM_002644 | 175.7304027 | 69.0634261 | 0.393009 | -2.54447 | 0.001618378 | 0.2525787 |
| KCNRG | NM_199464 | 62.12060607 | 30.50555345 | 0.491072 | -2.03636 | 0.003887199 | 0.266425 |
| KCNE1 | ENST00000399286 | 50.91433496 | 28.81523739 | 0.565955 | -1.76692 | 0.003847563 | 0.266425 |
| POU5F1P4 | NR_034180 | 60.91814051 | 120.6162101 | 1.97997 | 1.97997 | 0.002324226 | 0.255971 |
| PRH1-PRR4 | ENST00000228811 | 179.5410591 | 72.90369339 | 0.406056 | -2.46272 | 0.001408476 | 0.2497402 |
| PROM1 | NM_001145847 | 30.67986012 | 14.09547314 | 0.459437 | -2.17658 | 0.002168422 | 0.2536965 |
| PRSS23 | NM_007173 | 183.4839423 | 91.8246763 | 0.500451 | -1.9982 | 0.009538223 | 0.286724 |
| PSMB3 | ENST00000225426 | 19.13576935 | 33.66552364 | 1.75929 | 1.75929 | 0.01320148 | 0.3072697 |
| RP1 | NM_006269 | 16.11799069 | 7.767498189 | 0.481915 | -2.07506 | 0.000807895 | 0.2156663 |
| RRM2 | ENST00000360566 | 49.89938052 | 139.6043417 | 2.7977 | 2.7977 | 0.000176274 | 0.1618296 |
| RPL39L | ENST00000296277 | 28.30509773 | 50.48664837 | 1.78365 | 1.78365 | 0.00029214 | 0.189144 |
| S100A10 | NM_002966 | 110.1243734 | 209.320529 | 1.90077 | 1.90077 | 0.007036785 | 0.2803794 |
| S100A14 | ENST00000368702 | 54.95237768 | 102.2091114 | 1.85995 | 1.85995 | 3.66E-05 | 0.08506703 |
| S100A16 | ENST00000368703 | 29.56154977 | 65.09523337 | 2.20202 | 2.20202 | 0.006191037 | 0.2750615 |
| S100A8 | NM_002964 | 41.57804445 | 229.1724801 | 5.51185 | 5.51185 | 0.005748854 | 0.2705934 |
| S100A9 | NM_002965 | 92.97300623 | 289.0589636 | 3.10905 | 3.10905 | 0.001381146 | 0.2497402 |
| SCGB3A1 | ENST00000292641 | 117.6599893 | 57.96981595 | 0.492691 | -2.02967 | 0.002593802 | 0.2583437 |
| SCGB1A1 | NM_003357 | 1409.629201 | 418.4669863 | 0.296869 | -3.36849 | 0.0189305 | 0.3275087 |
| STK33 | NM_030906 | 23.61120882 | 12.81711804 | 0.54284 | -1.84216 | 0.006345018 | 0.2756742 |
| SERPINB5 | NM_002639 | 32.14539114 | 61.04156306 | 1.89892 | 1.89892 | 0.004880264 | 0.266425 |
| SERPINB13 | NM_012397 | 65.18689261 | 188.1213787 | 2.88587 | 2.88587 | 0.000761163 | 0.2121556 |
| SERPINB2 | NM_001143818 | 18.03215604 | 38.62224175 | 2.14186 | 2.14186 | 0.01505157 | 0.31618 |
| SGK1 | NM_001143676 | 62.11586979 | 120.3072688 | 1.93682 | 1.93682 | 0.007822846 | 0.2818056 |
| SSSCA1 | ENST00000309328 | 19.0690359 | 35.07108113 | 1.83916 | 1.83916 | 0.000268534 | 0.181772 |
| SLITRK6 | NM_032229 | 40.58205452 | 19.98824636 | 0.492541 | -2.03029 | 0.00998316 | 0.286724 |
| SPRR1A | NM_001199828 | 218.1550409 | 993.2134581 | 4.55277 | 4.55277 | 0.000752338 | 0.2121556 |
| SPRR1B | NM_003125 | 20.80444649 | 67.03534197 | 3.22217 | 3.22217 | 0.008709118 | 0.286724 |
| SPRR2A | NM_005988 | 33.01088702 | 293.7286131 | 8.8979 | 8.8979 | 0.005083687 | 0.2672274 |
| SPRR3 | NM_005416 | 43.4293965 | 259.3434279 | 5.97163 | 5.97163 | 5.47E-05 | 0.08506703 |
| SUMO2 | NM_006937 | 7.496306805 | 16.44731295 | 2.19406 | 2.19406 | 0.04233081 | 0.4008724 |
| SPAG6 | NM_012443 | 23.86228014 | 13.26322767 | 0.555823 | -1.79913 | 0.002148057 | 0.2532133 |
| SPAG8 | NM_172312 | 25.59304676 | 9.639644885 | 0.376652 | -2.65497 | 0.000780486 | 0.2143886 |
| SPA17 | ENST00000524614 | 39.62282942 | 17.58873718 | 0.443907 | -2.25273 | 0.002451082 | 0.2581064 |
| SPEF2 | NM_024867 | 16.37769022 | 9.146628147 | 0.558479 | -1.79058 | 0.006141157 | 0.2747064 |
| SPATA18 | NM_145263 | 43.98714315 | 23.63167524 | 0.537241 | -1.86136 | 0.008968766 | 0.286724 |
| SCD | NM_005063 | 41.46694908 | 84.13650586 | 2.02901 | 2.02901 | 0.03116499 | 0.3737281 |
| SAMD9 | NM_017654 | 19.18716957 | 45.19840634 | 2.35567 | 2.35567 | 0.001797509 | 0.2532133 |
| SFN | NM_006142 | 158.4957121 | 292.9194131 | 1.84811 | 1.84811 | 6.17E-05 | 0.08506703 |
| ST13P4 | NR_002183 | 8.770201562 | 4.810810593 | 0.548541 | -1.82302 | 0.02635195 | 0.3548661 |
| TEKT1 | ENST00000338694 | 51.98847747 | 25.74178157 | 0.495142 | -2.01962 | 0.004577901 | 0.266425 |
| TSPAN1 | NM_005727 | 258.0541461 | 106.2508078 | 0.411737 | -2.42874 | 0.007589774 | 0.2818056 |
| TSPAN8 | ENST00000393330 | 181.293074 | 35.65543253 | 0.196672 | -5.08462 | 0.000226603 | 0.1809222 |
| TTC18 | AF435959 | 21.16282032 | 10.33975431 | 0.488582 | -2.04674 | 0.005711252 | 0.2705934 |
| TTC25 | NM_031421 | 24.01693261 | 13.26635379 | 0.552376 | -1.81036 | 0.005084879 | 0.2672274 |
| TOP2A | NM_001067 | 13.67112441 | 30.51041715 | 2.23175 | 2.23175 | 0.003008614 | 0.2583437 |
| TM4SF1 | ENST00000305366 | 68.9476751 | 148.3221241 | 2.15123 | 2.15123 | 0.001906096 | 0.2532133 |
| TMC5 | NM_001105248 | 59.38843046 | 30.85644602 | 0.51957 | -1.92467 | 0.004075925 | 0.266425 |
| TMPRSS11D | NM_004262 | 21.76110634 | 41.58467351 | 1.91096 | 1.91096 | 0.0153361 | 0.3164692 |
| TMPRSS11A | ENST00000334830 | 15.04779297 | 35.92683648 | 2.3875 | 2.3875 | 0.01198759 | 0.2973482 |
| TMEM190 | NM_139172 | 151.0591747 | 82.01637074 | 0.542942 | -1.84182 | 0.004950161 | 0.2672274 |
| TFF3 | ENST00000518498 | 164.3388766 | 76.44922652 | 0.465193 | -2.14964 | 3.37E-05 | 0.08506703 |
| TPM3P9 | NR_003148 | 22.11632849 | 12.08419948 | 0.546394 | -1.83018 | 0.01583506 | 0.3176545 |
| TUBB | ENST00000421473 | 158.5055998 | 326.9123342 | 2.06247 | 2.06247 | 0.007168981 | 0.2803794 |
| TUBA1B | NM_006082 | 208.269822 | 464.1766047 | 2.22872 | 2.22872 | 0.04536235 | 0.4085781 |
| TUBA4B | NR_003063 | 73.98546678 | 33.71596535 | 0.455708 | -2.19439 | 0.001510993 | 0.2497402 |
| TUBA1A | NM_001270399 | 168.1202853 | 66.68868054 | 0.396672 | -2.52097 | 4.99E-05 | 0.08506703 |
| TPPP3 | ENST00000562206 | 117.118892 | 56.38658246 | 0.481449 | -2.07706 | 0.002511316 | 0.2583437 |
| UBXN10 | ENST00000375099 | 56.98893788 | 31.0463121 | 0.544781 | -1.8356 | 0.02276268 | 0.3402605 |
| LOC100506948 | ENST00000551938 | 27.28469478 | 13.12122035 | 0.480901 | -2.07943 | 0.03677189 | 0.3894378 |
| VWA3B | NM_144992 | 26.89246322 | 14.07097116 | 0.523231 | -1.9112 | 0.003593924 | 0.2645668 |
| WFDC6 | NM_080827 | 40.46380263 | 22.68395037 | 0.560601 | -1.7838 | 0.003249873 | 0.2583437 |
| WDR63 | NM_145172 | 17.14659254 | 9.345941456 | 0.545058 | -1.83467 | 0.01015323 | 0.286724 |
| WDR66 | NM_144668 | 51.64010396 | 28.8642133 | 0.558951 | -1.78907 | 0.006842407 | 0.2764471 |
| WDR96 | NM_025145 | 27.16524211 | 11.72088824 | 0.431467 | -2.31767 | 0.00455855 | 0.266425 |
| ZBBX | ENST00000307529 | 26.69411843 | 10.36609058 | 0.388327 | -2.57515 | 0.003168875 | 0.2583437 |
| ZMAT1 | NR_046008 | 28.02883741 | 12.81889499 | 0.457348 | -2.18652 | 1.82E-05 | 0.08506703 |
| ZNF273 | NR_003099 | 49.05403817 | 18.34455496 | 0.373968 | -2.67403 | 0.003253859 | 0.2583437 |
| ZNF818P | AK128250 | 22.92722749 | 11.7223507 | 0.511288 | -1.95584 | 0.01645339 | 0.3189553 |

Additional file 8. Table S8. Differential RNA expression in high-grade PSCC and low-grade PSCC.

| **Gene symbol** | **RefSeq** | **Mean ISCC** | **Mean control** | **Ratio** | **Fold change** | **P-value** | **Q-value** |
| --- | --- | --- | --- | --- | --- | --- | --- |
| AKD1 | ENST00000285397 | 19.61166339 | 10.44912332 | 0.532803 | -1.87687 | 0.004213862 | 0.266425 |
| AKR1B10 | NM_020299 | 25.21045061 | 56.24605474 | 2.23106 | 2.23106 | 0.000429712 | 0.199632 |
| AKR1C2 | NM_001135241 | 88.20447825 | 185.9949963 | 2.10869 | 2.10869 | 0.000361751 | 0.1958968 |
| A2M | ENST00000318602 | 122.244013 | 69.32385607 | 0.567094 | -1.76338 | 0.02350728 | 0.3435971 |
| APOBEC4 | NM_203454 | 32.95464673 | 15.47234538 | 0.469506 | -2.1299 | 0.006661047 | 0.2756742 |
| APOD | NM_001647 | 195.9484024 | 95.72607159 | 0.488525 | -2.04698 | 0.007271736 | 0.2818056 |
| ABCC5 | NM_005688 | 42.51529373 | 74.94550254 | 1.76279 | 1.76279 | 0.008886931 | 0.286724 |
| AGBL2 | NM_024783 | 27.65368816 | 12.1967934 | 0.441052 | -2.2673 | 0.002618625 | 0.2583437 |
| BEST4 | NM_153274 | 30.34043784 | 15.82342498 | 0.521528 | -1.91744 | 0.002000091 | 0.2532133 |
| BPIFB1 | NM_033197 | 1034.057002 | 185.088314 | 0.178989 | -5.58694 | 7.62E-05 | 0.08506703 |
| CDHR3 | NM_152750 | 78.73482573 | 27.0787643 | 0.343923 | -2.90763 | 0.003283922 | 0.2583437 |
| CASC1 | NM_001082972 | 15.89344537 | 8.15248567 | 0.512944 | -1.94953 | 0.01243671 | 0.3022021 |
| CDCA7L | NM_018719 | 19.88667257 | 10.96246248 | 0.551244 | -1.81408 | 0.005540857 | 0.2705934 |
| CDR1 | NM_004065 | 165.6207932 | 83.61906462 | 0.504882 | -1.98066 | 0.02539271 | 0.3520678 |
| CCL15 | ENST00000495214 | 68.44294817 | 32.99052885 | 0.482015 | -2.07462 | 0.001880379 | 0.2532133 |
| C1orf158 | NM_152290 | 42.6256521 | 23.66806734 | 0.555253 | -1.80098 | 0.01179643 | 0.294789 |
| C1orf88 | ENST00000369738 | 36.61467284 | 14.9872107 | 0.409321 | -2.44307 | 0.003092668 | 0.2583437 |
| C12orf63 | ENST00000342887 | 16.84933794 | 9.268141047 | 0.550062 | -1.81798 | 0.01102187 | 0.2890686 |
| C2orf40 | ENST00000238044 | 28.49328392 | 13.84218705 | 0.485805 | -2.05844 | 0.000145447 | 0.1531503 |
| C20orf85 | ENST00000371168 | 58.95368136 | 23.02150108 | 0.390501 | -2.56081 | 0.004015135 | 0.266425 |
| C6orf165 | ENST00000369562 | 15.49509828 | 8.782794208 | 0.566812 | -1.76425 | 0.009984593 | 0.286724 |
| CCDC108 | NM_194302 | 121.6008919 | 30.0538935 | 0.247153 | -4.04607 | 1.53E-05 | 0.08506703 |
| CCDC30 | ENST00000428554 | 20.79954408 | 11.30673121 | 0.543602 | -1.83958 | 0.01001324 | 0.286724 |
| CTGF | ENST00000367976 | 104.6102205 | 48.59614607 | 0.464546 | -2.15264 | 0.001203661 | 0.2497402 |
| DSC2 | NM_024422 | 21.35852331 | 45.13328866 | 2.11313 | 2.11313 | 0.02224441 | 0.3386391 |
| DSC3 | NM_024423 | 32.1761543 | 81.23389977 | 2.52466 | 2.52466 | 0.003066486 | 0.2583437 |
| DSG3 | NM_001944 | 25.93394902 | 72.39860529 | 2.79166 | 2.79166 | 0.01897527 | 0.3275087 |
| DSP | ENST00000379802 | 79.13039205 | 178.1884481 | 2.25183 | 2.25183 | 0.002148577 | 0.2532133 |
| DCDC1 | NM_181807 | 25.90035559 | 14.40282592 | 0.556087 | -1.79828 | 0.005293442 | 0.2705934 |
| DNAI1 | NM_012144 | 40.0100354 | 17.92026417 | 0.447894 | -2.23267 | 0.002386772 | 0.2581064 |
| DNAH3 | ENST00000544558 | 17.92423946 | 8.040582382 | 0.448587 | -2.22922 | 0.000694102 | 0.2055554 |
| DNAH3 | NM_017539 | 29.58061208 | 15.83461626 | 0.535305 | -1.86809 | 0.009108611 | 0.286724 |
| DNAH6 | NM_001370 | 23.67233315 | 12.44461765 | 0.525706 | -1.90221 | 0.01782388 | 0.3202573 |
| DNAH6 | BC015442 | 18.37751762 | 8.922574239 | 0.485515 | -2.05967 | 0.008260054 | 0.2841299 |
| DNAH12 | NM_198564 | 38.07283248 | 15.24854834 | 0.400508 | -2.49683 | 0.002478475 | 0.2581064 |
| DNAH9 | ENST00000262442 | 31.8159876 | 17.31967556 | 0.544371 | -1.83698 | 0.003434306 | 0.2583437 |
| DYNLT3 | NM_006520 | 43.87903826 | 90.52221617 | 2.06299 | 2.06299 | 0.01759455 | 0.3199653 |
| DNAH10 | BC144575 | 20.52314609 | 11.38766458 | 0.554869 | -1.80223 | 0.00924562 | 0.286724 |
| DNAAF1 | NM_178452 | 105.4985968 | 37.49015798 | 0.355362 | -2.81403 | 0.001710898 | 0.2532133 |
| DYTN | NM_001093730 | 18.28108762 | 9.882869566 | 0.540607 | -1.84977 | 0.004473789 | 0.266425 |
| ENKUR | NM_145010 | 25.01584014 | 12.8732998 | 0.514605 | -1.94324 | 0.01700689 | 0.3191793 |
| FAM216B | NM_182508 | 29.15052578 | 12.97605559 | 0.44514 | -2.24648 | 0.01168975 | 0.294789 |
| GJB6 | NM_001110219 | 20.24256212 | 49.29707012 | 2.43532 | 2.43532 | 0.000574339 | 0.199632 |
| GPX2 | ENST00000389614 | 63.78918761 | 146.5582122 | 2.29754 | 2.29754 | 0.00050687 | 0.199632 |
| GSTA2 | NM_000846 | 67.98714111 | 13.63166612 | 0.200504 | -4.98743 | 0.000455285 | 0.199632 |
| HIST1H2BK | NM_080593 | 44.33364263 | 96.8385787 | 2.18431 | 2.18431 | 0.03693015 | 0.3894378 |
| HYDIN | NM_032821 | 46.73721942 | 23.04337295 | 0.493043 | -2.02822 | 0.002835293 | 0.2583437 |
| ITGA6 | NM_000210 | 30.43416755 | 56.34204407 | 1.85127 | 1.85127 | 0.0211319 | 0.3354459 |
| IQUB | ENST00000466202 | 10.0720818 | 5.710313687 | 0.566947 | -1.76383 | 0.01086616 | 0.2881671 |
| KRT13 | ENST00000246635 | 126.9274238 | 888.1687493 | 6.99744 | 6.99744 | 1.40E-05 | 0.08506703 |
| KRT16 | NM_005557 | 51.40048261 | 141.9914254 | 2.76246 | 2.76246 | 0.01028304 | 0.286724 |
| KRT5 | ENST00000252242 | 181.2704561 | 358.2765975 | 1.97648 | 1.97648 | 0.000537852 | 0.199632 |
| KRT6B | NM_005555 | 330.8560843 | 2288.997227 | 6.91835 | 6.91835 | 0.000329322 | 0.189144 |
| KRT6C | NM_173086 | 30.15300749 | 109.6825342 | 3.63753 | 3.63753 | 0.006269427 | 0.2750615 |
| LRRC23 | NM_001135217 | 77.49224309 | 33.16385933 | 0.427965 | -2.33664 | 0.002196699 | 0.2537682 |
| LRRC46 | NM_033413 | 37.49587538 | 19.57146709 | 0.521964 | -1.91584 | 0.008811014 | 0.286724 |
| LRRIQ1 | NM_001079910 | 22.19111111 | 9.799512631 | 0.441599 | -2.2645 | 0.00860195 | 0.286724 |
| MGP | NM_001190839 | 83.53043233 | 32.73994516 | 0.391953 | -2.55133 | 0.000735664 | 0.2112619 |
| MS4A8B | ENST00000450141 | 92.5480059 | 31.42912059 | 0.339598 | -2.94466 | 0.001537268 | 0.2497402 |
| MFAP4 | NM_001198695 | 51.67268695 | 25.19560162 | 0.4876 | -2.05086 | 0.002728711 | 0.2583437 |
| MSMB | NM_002443 | 780.4919813 | 103.5432718 | 0.132663 | -7.53787 | 4.29E-05 | 0.08506703 |
| PI3 | ENST00000243924 | 39.72320159 | 149.1747966 | 3.75535 | 3.75535 | 0.002558075 | 0.2583437 |
| PIGX | NM_001166304 | 11.16297763 | 20.72743945 | 1.85681 | 1.85681 | 0.007617338 | 0.2818056 |
| PKP1 | ENST00000263946 | 39.41191648 | 71.50290741 | 1.81425 | 1.81425 | 0.01046066 | 0.286724 |
| PLEKHS1 | ENST00000369312 | 76.60836362 | 25.91903319 | 0.33833 | -2.9557 | 0.00182865 | 0.2532133 |
| PIGR | NM_002644 | 175.7304027 | 69.0634261 | 0.393009 | -2.54447 | 0.001618378 | 0.2525787 |
| KCNE1 | ENST00000399286 | 50.91433496 | 28.81523739 | 0.565955 | -1.76692 | 0.003847563 | 0.266425 |
| PSMB3 | ENST00000225426 | 19.13576935 | 33.66552364 | 1.75929 | 1.75929 | 0.01320148 | 0.3072697 |
| RP1 | NM_006269 | 16.11799069 | 7.767498189 | 0.481915 | -2.07506 | 0.000807895 | 0.2156663 |
| RPL39L | ENST00000296277 | 28.30509773 | 50.48664837 | 1.78365 | 1.78365 | 0.00029214 | 0.189144 |
| S100A16 | ENST00000368703 | 29.56154977 | 65.09523337 | 2.20202 | 2.20202 | 0.006191037 | 0.2750615 |
| SCGB1A1 | NM_003357 | 1409.629201 | 418.4669863 | 0.296869 | -3.36849 | 0.0189305 | 0.3275087 |
| SERPINB13 | NM_012397 | 65.18689261 | 188.1213787 | 2.88587 | 2.88587 | 0.000761163 | 0.2121556 |
| SGK1 | NM_001143676 | 62.11586979 | 120.3072688 | 1.93682 | 1.93682 | 0.007822846 | 0.2818056 |
| SLITRK6 | NM_032229 | 40.58205452 | 19.98824636 | 0.492541 | -2.03029 | 0.00998316 | 0.286724 |
| SPRR1A | NM_001199828 | 218.1550409 | 993.2134581 | 4.55277 | 4.55277 | 0.000752338 | 0.2121556 |
| SPRR1B | NM_003125 | 20.80444649 | 67.03534197 | 3.22217 | 3.22217 | 0.008709118 | 0.286724 |
| SPRR2A | NM_005988 | 33.01088702 | 293.7286131 | 8.8979 | 8.8979 | 0.005083687 | 0.2672274 |
| SPAG8 | NM_172312 | 25.59304676 | 9.639644885 | 0.376652 | -2.65497 | 0.000780486 | 0.2143886 |
| SPATA18 | NM_145263 | 43.98714315 | 23.63167524 | 0.537241 | -1.86136 | 0.008968766 | 0.286724 |
| SCD | NM_005063 | 41.46694908 | 84.13650586 | 2.02901 | 2.02901 | 0.03116499 | 0.3737281 |
| SAMD9 | NM_017654 | 19.18716957 | 45.19840634 | 2.35567 | 2.35567 | 0.001797509 | 0.2532133 |
| TEKT1 | ENST00000338694 | 51.98847747 | 25.74178157 | 0.495142 | -2.01962 | 0.004577901 | 0.266425 |
| TSPAN1 | NM_005727 | 258.0541461 | 106.2508078 | 0.411737 | -2.42874 | 0.007589774 | 0.2818056 |
| TSPAN8 | ENST00000393330 | 181.293074 | 35.65543253 | 0.196672 | -5.08462 | 0.000226603 | 0.1809222 |
| TTC18 | AF435959 | 21.16282032 | 10.33975431 | 0.488582 | -2.04674 | 0.005711252 | 0.2705934 |
| TOP2A | NM_001067 | 13.67112441 | 30.51041715 | 2.23175 | 2.23175 | 0.003008614 | 0.2583437 |
| TM4SF1 | ENST00000305366 | 68.9476751 | 148.3221241 | 2.15123 | 2.15123 | 0.001906096 | 0.2532133 |
| TFF3 | ENST00000518498 | 164.3388766 | 76.44922652 | 0.465193 | -2.14964 | 3.37E-05 | 0.08506703 |
| TUBA1A | NM_001270399 | 168.1202853 | 66.68868054 | 0.396672 | -2.52097 | 4.99E-05 | 0.08506703 |
| TUBB | ENST00000421473 | 158.5055998 | 326.9123342 | 2.06247 | 2.06247 | 0.007168981 | 0.2803794 |
| TUBA4B | NR_003063 | 73.98546678 | 33.71596535 | 0.455708 | -2.19439 | 0.001510993 | 0.2497402 |
| TUBA1B | NM_006082 | 208.269822 | 464.1766047 | 2.22872 | 2.22872 | 0.04536235 | 0.4085781 |
| TPPP3 | ENST00000562206 | 117.118892 | 56.38658246 | 0.481449 | -2.07706 | 0.002511316 | 0.2583437 |
| WDR63 | NM_145172 | 17.14659254 | 9.345941456 | 0.545058 | -1.83467 | 0.01015323 | 0.286724 |
| WDR96 | NM_025145 | 27.16524211 | 11.72088824 | 0.431467 | -2.31767 | 0.00455855 | 0.266425 |
| ZMAT1 | NR_046008 | 28.02883741 | 12.81889499 | 0.457348 | -2.18652 | 1.82E-05 | 0.08506703 |
| ZBBX | ENST00000307529 | 26.69411843 | 10.36609058 | 0.388327 | -2.57515 | 0.003168875 | 0.2583437 |
| ZNF273 | NR_003099 | 49.05403817 | 18.34455496 | 0.373968 | -2.67403 | 0.003253859 | 0.2583437 |

Additional file 9. Table S9. Ingenuity Pathway Analysis.

| **Contrast** | **Ingenuity Canonical Pathways** | **-log(p-value)** | **Ratio** | **Z-score** | **Molecules** |
| --- | --- | --- | --- | --- | --- |
| all ISCC versus paired controls | Cell Cycle: G2/M DNA Damage Checkpoint Regulation | 5.66E00 | 3.16E-01 | -1.414 | TOP2A,YWHAQ,YWHAG,AURKA,SKP2,PRKDC,CKS2,CCNB2,YWHAB,CDK1,CCNB1,CHEK1 |
| all ISCC versus paired controls | GADD45 Signaling | 5.04E00 | 5E-01 |  | CDK4,CCND2,CDK2,GADD45B,PCNA,CDK1,CCNB1 |
| all ISCC versus paired controls | p53 Signaling | 4.24E00 | 2E-01 | -0.905 | JUN,CDK4,CCND2,BIRC5,SERPINE2,CDK2,GADD45B,PCNA,TOPBP1,PIK3CA,SNAI2,HIF1A,BAX,PRKDC,CHEK1 |
| all ISCC versus paired controls | Pyrimidine Deoxyribonucleotides De Novo Biosynthesis I | 3.72E00 | 4E-01 |  | RRM2,RRM1,AK9,AK7,NME1,NME5 |
| all ISCC versus paired controls | Agranulocyte Adhesion and Diapedesis | 3.33E00 | 1.56E-01 |  | CLDN3,ITGA6,MSN,CLDN4,CCL11,CXCL2,VCAM1,GNAI3,CXCL8,CXCL6,GLG1,FN1,CXCL17,ITGB1,CCL2,CXCL1,MMP14 |
| all ISCC versus paired controls | Oleate Biosynthesis II (Animals) | 3.32E00 | 5.71E-01 |  | CYB5A,SCD,FADS1,FADS2 |
| all ISCC versus paired controls | Granulocyte Adhesion and Diapedesis | 3.24E00 | 1.53E-01 |  | CLDN3,ITGA6,MSN,CLDN4,CCL11,THY1,CXCL2,VCAM1,GNAI3,CXCL8,CXCL6,GLG1,CXCL17,ITGB1,CCL2,CXCL1,MMP14 |
| all ISCC versus paired controls | Cell Cycle Control of Chromosomal Replication | 2.95E00 | 3E-01 |  | CDK4,CDK2,MCM6,MCM4,MCM5,CDC6 |
| all ISCC versus paired controls | Atherosclerosis Signaling | 2.85E00 | 1.63E-01 |  | COL10A1,COL3A1,COL1A2,CCL11,COL1A1,VCAM1,CXCL8,PDGFC,GLG1,CLU,CCL2,F3,ALOX15 |
| all ISCC versus paired controls | Hepatic Fibrosis / Hepatic Stellate Cell Activation | 2.78E00 | 1.33E-01 |  | COL10A1,COL6A3,KLF6,COL3A1,COL21A1,CTGF,COL1A2,COL1A1,VCAM1,CXCL8,PDGFC,A2M,COL12A1,COL5A2,FN1,STAT1,CCL2,BAX,HGF |
| all ISCC versus paired controls | ATM Signaling | 2.76E00 | 2E-01 | -1.134 | JUN,CBX5,MAPK10,CDK2,GADD45B,CCNB2,CDK1,CCNB1,CHEK1 |
| all ISCC versus paired controls | Granzyme A Signaling | 2.76E00 | 3.33E-01 |  | HIST1H1A,HMGB2,HIST1H1E,NME1,SET |
| all ISCC versus paired controls | Mitotic Roles of Polo-Like Kinase | 2.56E00 | 1.88E-01 |  | ANAPC13,RAD21,HSP90B1,PRC1,HSP90AB1,CCNB2,KIF11,CDK1,CCNB1 |
| all ISCC versus paired controls | Remodeling of Epithelial Adherens Junctions | 2.37E00 | 1.76E-01 | 1.000 | ARPC2,TUBB4A,RAB7A,RALA,MAPRE1,TUBA4A,NME1,HGF,ACTR3 |
| all ISCC versus paired controls | DNA damage-induced 14-3-3σ Signaling | 2.28E00 | 3.33E-01 |  | CDK2,CCNB2,CDK1,CCNB1 |
| all ISCC versus paired controls | Pyrimidine Ribonucleotides Interconversion | 2.16E00 | 2.5E-01 |  | AK9,AK7,NME1,NME5,CTPS1 |
| all ISCC versus paired controls | Role of IL-17A in Arthritis | 1.99E00 | 1.79E-01 |  | CXCL6,PIK3CA,CCL2,MAPK10,PTGS2,CXCL1,CXCL8 |
| all ISCC versus paired controls | Intrinsic Prothrombin Activation Pathway | 1.97E00 | 2.27E-01 |  | PROS1,COL10A1,COL3A1,COL1A2,COL1A1 |
| all ISCC versus paired controls | Pyrimidine Ribonucleotides De Novo Biosynthesis | 1.97E00 | 2.27E-01 |  | AK9,AK7,NME1,NME5,CTPS1 |
| all ISCC versus paired controls | Pyruvate Fermentation to Lactate | 1.94E00 | 6.67E-01 |  | LDHA,LDHB |
| all ISCC versus paired controls | RAN Signaling | 1.91E00 | 2.67E-01 |  | CSE1L,XPO1,KPNA4,KPNB1 |
| all ISCC versus paired controls | Glycolysis I | 1.91E00 | 2.67E-01 |  | PGK1,PKM,ENO1,GPI |
| all ISCC versus paired controls | Inhibition of Matrix Metalloproteases | 1.74E00 | 2E-01 |  | A2M,THBS2,TIMP4,ADAM12,MMP14 |
| all ISCC versus paired controls | Salvage Pathways of Pyrimidine Ribonucleotides | 1.73E00 | 1.41E-01 |  | PAK2,CDK4,AK9,TTK,AK7,CDK2,NME1,NME5,CDK1 |
| all ISCC versus paired controls | IL-17 Signaling | 1.67E00 | 1.45E-01 |  | JUN,PIK3CA,CCL2,MAPK10,PTGS2,CXCL1,CCL11,CXCL8 |
| all ISCC versus paired controls | Role of IL-17A in Psoriasis | 1.67E00 | 3E-01 |  | CXCL6,CXCL1,CXCL8 |
| all ISCC versus paired controls | Estrogen-mediated S-phase Entry | 1.62E00 | 2.22E-01 |  | CDK4,SKP2,CDK2,CDK1 |
| all ISCC versus paired controls | IL-17A Signaling in Gastric Cells | 1.54E00 | 2.11E-01 |  | JUN,MAPK10,CXCL1,CXCL8 |
| all ISCC versus paired controls | tRNA Charging | 1.54E00 | 1.79E-01 |  | EPRS,YARS,AARS,TARS,IARS |
| all ISCC versus paired controls | Pancreatic Adenocarcinoma Signaling | 1.48E00 | 1.22E-01 | 0.333 | CDK4,PIK3CA,STAT1,MAPK10,PTGS2,RALA,BIRC5,CDK2,PA2G4,PDGFC |
| all ISCC versus paired controls | Role of Tissue Factor in Cancer | 1.48E00 | 1.22E-01 |  | CFL1,PIK3CA,ITGAV,ITGB1,ITGA6,CXCL1,CTGF,F3,EGR1,CXCL8 |
| all ISCC versus paired controls | Superpathway of Serine and Glycine Biosynthesis I | 1.45E00 | 4E-01 |  | PSAT1,SHMT2 |
| all ISCC versus paired controls | Pentose Phosphate Pathway (Non-oxidative Branch) | 1.45E00 | 4E-01 |  | TKT,TALDO1 |
| all ISCC versus paired controls | Leucine Degradation I | 1.45E00 | 4E-01 |  | BCAT1,MCCC1 |
| all ISCC versus paired controls | Fatty Acid α-oxidation | 1.44E00 | 2.5E-01 |  | BCO2,PTGS2,ALDH1A3 |
| all ISCC versus paired controls | Aryl Hydrocarbon Receptor Signaling | 1.43E00 | 1.16E-01 | 0.707 | JUN,CDK4,CCND2,HSP90B1,ALDH3B1,CDK2,TYR,BAX,HSP90AB1,ALDH1A3,CHEK1 |
| all ISCC versus paired controls | Cyclins and Cell Cycle Regulation | 1.43E00 | 1.31E-01 |  | CDK4,CCND2,SKP2,CDK2,PA2G4,CCNB2,CDK1,CCNB1 |
| all ISCC versus paired controls | Hypoxia Signaling in the Cardiovascular System | 1.4E00 | 1.37E-01 |  | JUN,HSP90B1,UBE2V2,HIF1A,LDHA,UBE2C,HSP90AB1 |
| all ISCC versus paired controls | γ-linolenate Biosynthesis II (Animals) | 1.35E00 | 2.31E-01 |  | CYB5A,FADS1,FADS2 |
| all ISCC versus paired controls | Gluconeogenesis I | 1.35E00 | 2.31E-01 |  | PGK1,ENO1,GPI |
| all ISCC versus paired controls | HIF1α Signaling | 1.22E00 | 1.19E-01 |  | JUN,PIK3CA,MAPK10,HIF1A,LDHA,MMP14,PDGFC,LDHB |
| all ISCC versus paired controls | Myc Mediated Apoptosis Signaling | 1.21E00 | 1.33E-01 |  | YWHAQ,PIK3CA,YWHAG,MAPK10,BAX,YWHAB |
| all ISCC versus paired controls | L-dopachrome Biosynthesis | 1.2E00 | 1E00 |  | TYR |
| all ISCC versus paired controls | Putrescine Biosynthesis III | 1.2E00 | 1E00 |  | ODC1 |
| all ISCC versus paired controls | ILK Signaling | 1.17E00 | 1E-01 | 0.577 | JUN,DSP,MAPK10,MUC1,PDGFC,FN1,CFL1,PIK3CA,SNAI2,ITGB1,HIF1A,PTGS2,TMSB10/TMSB4X |
| all ISCC versus paired controls | NAD Phosphorylation and Dephosphorylation | 1.17E00 | 2.86E-01 |  | ACP1,NADK2 |
| all ISCC versus paired controls | Folate Transformations I | 1.17E00 | 2.86E-01 |  | SHMT2,MTHFD2 |
| all ISCC versus paired controls | Complement System | 1.16E00 | 1.6E-01 |  | CD59,CD55,CFD,C6 |
| all ISCC versus paired controls | IL-8 Signaling | 1.11E00 | 9.59E-02 | 0.277 | JUN,PAK2,CCND2,ITGAV,GNB2L1,MAPK10,VCAM1,GNAI3,CXCL8,PDGFC,PIK3CA,PTGS2,CXCL1,BAX |
| all ISCC versus paired controls | Prostanoid Biosynthesis | 1.06E00 | 2.5E-01 |  | PTGDS,PTGS2 |
| all ISCC versus paired controls | Pentose Phosphate Pathway | 1.06E00 | 2.5E-01 |  | TKT,TALDO1 |
| all ISCC versus paired controls | IL-22 Signaling | 1.05E00 | 1.76E-01 |  | SOCS3,STAT1,MAPK10 |
| all ISCC versus paired controls | Role of IL-17F in Allergic Inflammatory Airway Diseases | 1.01E00 | 1.43E-01 |  | CXCL6,CCL2,CXCL1,CXCL8 |
| all ISCC versus paired controls | HGF Signaling | 1.01E00 | 1.03E-01 | 0.000 | JUN,PIK3CA,ITGB1,MAPK10,PTGS2,CDK2,ELF3,HGF,ETS2 |
| all ISCC versus paired controls | CDK5 Signaling | 9.99E-01 | 1.07E-01 | 0.000 | PPP1CC,ITGB1,MAPK10,ITGA6,LAMC1,FOSB,NTRK2,EGR1 |
| all ISCC versus paired controls | Superpathway of Cholesterol Biosynthesis | 9.95E-01 | 1.67E-01 |  | HMGCS1,SC5D,DHCR7 |
| all ISCC versus paired controls | Endoplasmic Reticulum Stress Pathway | 9.95E-01 | 1.67E-01 |  | CALR,HSP90B1,HSPA5 |
| all ISCC versus paired controls | Role of CHK Proteins in Cell Cycle Checkpoint Control | 9.76E-01 | 1.25E-01 | 1.000 | CDK2,CLSPN,PCNA,CDK1,CHEK1 |
| all ISCC versus paired controls | Cholesterol Biosynthesis I | 9.71E-01 | 2.22E-01 |  | SC5D,DHCR7 |
| all ISCC versus paired controls | Cholesterol Biosynthesis II (via 24,25-dihydrolanosterol) | 9.71E-01 | 2.22E-01 |  | SC5D,DHCR7 |
| all ISCC versus paired controls | Cholesterol Biosynthesis III (via Desmosterol) | 9.71E-01 | 2.22E-01 |  | SC5D,DHCR7 |
| all ISCC versus paired controls | Role of JAK family kinases in IL-6-type Cytokine Signaling | 9.4E-01 | 1.58E-01 |  | SOCS3,STAT1,MAPK10 |
| all ISCC versus paired controls | Diphthamide Biosynthesis | 9.12E-01 | 5E-01 |  | EEF2 |
| all ISCC versus paired controls | Guanine and Guanosine Salvage I | 9.12E-01 | 5E-01 |  | HPRT1 |
| all ISCC versus paired controls | Eumelanin Biosynthesis | 9.12E-01 | 5E-01 |  | TYR |
| all ISCC versus paired controls | Glycine Biosynthesis I | 9.12E-01 | 5E-01 |  | SHMT2 |
| all ISCC versus paired controls | Small Cell Lung Cancer Signaling | 9.06E-01 | 1.11E-01 |  | CDK4,PIK3CA,PTGS2,SKP2,CDK2,PA2G4 |
| all ISCC versus paired controls | Granzyme B Signaling | 8.92E-01 | 2E-01 |  | LMNB1,PRKDC |
| all ISCC versus paired controls | Mismatch Repair in Eukaryotes | 8.92E-01 | 2E-01 |  | EXO1,PCNA |
| all ISCC versus paired controls | 14-3-3-mediated Signaling | 8.77E-01 | 9.68E-02 |  | JUN,TUBB4A,YWHAQ,PIK3CA,YWHAG,MAPK10,TUBA4A,BAX,YWHAB |
| all ISCC versus paired controls | Protein Ubiquitination Pathway | 8.7E-01 | 8.46E-02 |  | HSPB11,DNAJB13,UCHL1,HSP90B1,PSMD11,DNAJC21,USP2,UBE2V2,HSPA5,SKP2,DNAJB11,PSMD14,UBE2C,PSMB2,DNAJB6,HSP90AB1,PSMD2 |
| all ISCC versus paired controls | TR/RXR Activation | 8.64E-01 | 1.03E-01 |  | UCP2,TBL1XR1,PPARGC1A,PIK3CA,COL6A3,HIF1A,ENO1 |
| all ISCC versus paired controls | Antiproliferative Role of TOB in T Cell Signaling | 8.42E-01 | 1.43E-01 |  | TOB1,SKP2,CDK2 |
| all ISCC versus paired controls | Noradrenaline and Adrenaline Degradation | 8.42E-01 | 1.43E-01 |  | ADH1B,ADH6,ALDH1A3 |
| all ISCC versus paired controls | HIPPO signaling | 8.4E-01 | 1.01E-01 | -1.000 | PPP1CC,YWHAQ,DLG1,YWHAG,SKP2,FRMD6,YWHAB |
| all ISCC versus paired controls | Agrin Interactions at Neuromuscular Junction | 8.26E-01 | 1.05E-01 | 0.447 | JUN,PAK2,ITGB1,MAPK10,ITGA6,LAMC1 |
| all ISCC versus paired controls | Differential Regulation of Cytokine Production in Macrophages and T Helper Cells by IL-17A and IL-17F | 8.23E-01 | 1.82E-01 |  | CCL2,CXCL1 |
| all ISCC versus paired controls | PAK Signaling | 7.95E-01 | 9.86E-02 | 1.633 | PAK2,CFL1,PIK3CA,ITGB1,MAPK10,NCK1,PDGFC |
| all ISCC versus paired controls | Cdc42 Signaling | 7.75E-01 | 9.41E-02 | 1.414 | JUN,ARPC2,PAK2,CFL1,ITGB1,MAPK10,RALA,ACTR3 |
| all ISCC versus paired controls | Extrinsic Prothrombin Activation Pathway | 7.61E-01 | 1.67E-01 |  | PROS1,F3 |
| all ISCC versus paired controls | MIF Regulation of Innate Immunity | 7.57E-01 | 1.3E-01 |  | JUN,MAPK10,PTGS2 |
| all ISCC versus paired controls | Ethanol Degradation II | 7.57E-01 | 1.3E-01 |  | ADH1B,ADH6,ALDH1A3 |
| all ISCC versus paired controls | Rac Signaling | 7.55E-01 | 9.3E-02 | 2.121 | JUN,ARPC2,PAK2,CFL1,PIK3CA,ITGB1,ACTR3,WASF1 |
| all ISCC versus paired controls | Glucocorticoid Receptor Signaling | 7.54E-01 | 8.1E-02 |  | JUN,HSP90B1,MAPK10,HLTF,CCL11,VCAM1,CXCL8,FKBP4,TAF13,A2M,POLR2H,PIK3CA,STAT1,HSPA5,CCL2,PTGS2,HSP90AB1 |
| all ISCC versus paired controls | Serine Biosynthesis | 7.5E-01 | 3.33E-01 |  | PSAT1 |
| all ISCC versus paired controls | Spermine and Spermidine Degradation I | 7.5E-01 | 3.33E-01 |  | SAT1 |
| all ISCC versus paired controls | Histidine Degradation III | 7.5E-01 | 3.33E-01 |  | MTHFD2 |
| all ISCC versus paired controls | Folate Polyglutamylation | 7.5E-01 | 3.33E-01 |  | SHMT2 |
| all ISCC versus paired controls | S-adenosyl-L-methionine Biosynthesis | 7.5E-01 | 3.33E-01 |  | MAT1A |
| all ISCC versus paired controls | Leukocyte Extravasation Signaling | 7.43E-01 | 8.51E-02 | 2.333 | PIK3CA,CLDN3,ITGB1,MAPK10,ITGA6,MSN,CLDN4,TIMP4,THY1,GNAI3,VCAM1,MMP14 |
| all ISCC versus paired controls | ERK/MAPK Signaling | 7.43E-01 | 8.51E-02 | 1.000 | HIST1H3C,PPP1CC,YWHAQ,PAK2,PIK3CA,YWHAG,STAT1,ITGB1,ATF1,ELF3,YWHAB,ETS2 |
| all ISCC versus paired controls | Actin Nucleation by ARP-WASP Complex | 7.34E-01 | 1.04E-01 | 2.000 | ARPC2,ITGB1,ACTR3,NCK1,WASF1 |
| all ISCC versus paired controls | Pyridoxal 5'-phosphate Salvage Pathway | 7.08E-01 | 1.02E-01 |  | PAK2,CDK4,TTK,CDK2,CDK1 |
| all ISCC versus paired controls | Regulation of Actin-based Motility by Rho | 7.07E-01 | 9.68E-02 | 2.236 | ARPC2,PAK2,CFL1,ITGB1,ACTR3,WASF1 |
| all ISCC versus paired controls | Putrescine Degradation III | 7.06E-01 | 1.54E-01 |  | SAT1,ALDH1A3 |
| all ISCC versus paired controls | Cell Cycle: G1/S Checkpoint Regulation | 6.84E-01 | 1E-01 |  | CDK4,CCND2,SKP2,CDK2,PA2G4 |
| all ISCC versus paired controls | IL-9 Signaling | 6.82E-01 | 1.2E-01 |  | PIK3CA,SOCS3,STAT1 |
| all ISCC versus paired controls | IL-17A Signaling in Fibroblasts | 6.82E-01 | 1.2E-01 |  | JUN,CCL2,NFKBIZ |
| all ISCC versus paired controls | Caveolar-mediated Endocytosis Signaling | 6.6E-01 | 9.8E-02 |  | ITGAV,COPB2,ITGB1,ITGA6,CD55 |
| all ISCC versus paired controls | CD40 Signaling | 6.6E-01 | 9.8E-02 | 0.000 | JUN,PIK3CA,ATF1,MAPK10,PTGS2 |
| all ISCC versus paired controls | IL-17A Signaling in Airway Cells | 6.6E-01 | 9.8E-02 |  | CXCL6,PIK3CA,MAPK10,CXCL1,CCL11 |
| all ISCC versus paired controls | GDNF Family Ligand-Receptor Interactions | 6.6E-01 | 9.8E-02 | 0.447 | JUN,PIK3CA,MAPK10,DOK3,NCK1 |
| all ISCC versus paired controls | Chemokine Signaling | 6.6E-01 | 9.8E-02 | -1.342 | JUN,CFL1,CCL2,CCL11,GNAI3 |
| all ISCC versus paired controls | IGF-1 Signaling | 6.54E-01 | 8.97E-02 |  | JUN,YWHAQ,PIK3CA,YWHAG,SOCS3,CTGF,YWHAB |
| all ISCC versus paired controls | Role of JAK2 in Hormone-like Cytokine Signaling | 6.49E-01 | 1.15E-01 |  | SOCS3,STAT1,HLTF |
| all ISCC versus paired controls | Tetrahydrofolate Salvage from 5,10-methenyltetrahydrofolate | 6.38E-01 | 2.5E-01 |  | MTHFD2 |
| all ISCC versus paired controls | Salvage Pathways of Pyrimidine Deoxyribonucleotides | 6.38E-01 | 2.5E-01 |  | TK1 |
| all ISCC versus paired controls | dTMP De Novo Biosynthesis | 6.38E-01 | 2.5E-01 |  | SHMT2 |
| all ISCC versus paired controls | UVB-Induced MAPK Signaling | 6.36E-01 | 1.03E-01 |  | JUN,HIST1H3C,PIK3CA,MAPK10 |
| all ISCC versus paired controls | phagosome maturation | 6.35E-01 | 8.86E-02 |  | CALR,TUBB4A,RAB7A,ATP6V1B2,YKT6,LAMP2,TUBA4A |
| all ISCC versus paired controls | Epithelial Adherens Junction Signaling | 6.29E-01 | 8.41E-02 |  | ARPC2,TUBB4A,SNAI2,JUP,LMO7,TUBA4A,HGF,ACTR3,WASF1 |
| all ISCC versus paired controls | ErbB Signaling | 6.25E-01 | 9.09E-02 |  | JUN,AREG,PAK2,PIK3CA,MAPK10,NCK1 |
| all ISCC versus paired controls | Docosahexaenoic Acid (DHA) Signaling | 6.17E-01 | 1.11E-01 |  | PIK3CA,BAX,ALOX15 |
| all ISCC versus paired controls | Differential Regulation of Cytokine Production in Intestinal Epithelial Cells by IL-17A and IL-17F | 6.12E-01 | 1.33E-01 |  | CCL2,CXCL1 |
| all ISCC versus paired controls | PPAR Signaling | 5.87E-01 | 8.82E-02 | -0.816 | JUN,PPARGC1A,HSP90B1,PTGS2,HSP90AB1,PDGFC |
| all ISCC versus paired controls | Regulation of Cellular Mechanics by Calpain Protease | 5.87E-01 | 9.76E-02 |  | CDK4,ITGB1,CDK2,CDK1 |
| all ISCC versus paired controls | Mechanisms of Viral Exit from Host Cells | 5.87E-01 | 1.07E-01 |  | XPO1,SNF8,LMNB1 |
| all ISCC versus paired controls | Hereditary Breast Cancer Signaling | 5.84E-01 | 8.33E-02 |  | POLR2H,CDK4,PIK3CA,HLTF,GADD45B,CDK1,CCNB1,CHEK1 |
| all ISCC versus paired controls | Role of BRCA1 in DNA Damage Response | 5.74E-01 | 9.09E-02 |  | TOPBP1,STAT1,ATF1,HLTF,CHEK1 |
| all ISCC versus paired controls | PCP pathway | 5.74E-01 | 9.09E-02 | -0.447 | JUN,WNT5A,MAPK10,FZD7,JUNB |
| all ISCC versus paired controls | Paxillin Signaling | 5.68E-01 | 8.43E-02 | 1.342 | PAK2,PIK3CA,ITGAV,ITGB1,MAPK10,ITGA6,NCK1 |
| all ISCC versus paired controls | Coagulation System | 5.58E-01 | 1.03E-01 |  | A2M,PROS1,F3 |
| all ISCC versus paired controls | Phosphatidylethanolamine Biosynthesis II | 5.55E-01 | 2E-01 |  | EPT1 |
| all ISCC versus paired controls | Adenine and Adenosine Salvage III | 5.55E-01 | 2E-01 |  | HPRT1 |
| all ISCC versus paired controls | GDP-mannose Biosynthesis | 5.55E-01 | 2E-01 |  | GPI |
| all ISCC versus paired controls | HMGB1 Signaling | 5.52E-01 | 8.33E-02 | -0.378 | JUN,PIK3CA,CCL2,MAPK10,RBBP7,VCAM1,CXCL8 |
| all ISCC versus paired controls | LPS/IL-1 Mediated Inhibition of RXR Function | 5.43E-01 | 7.75E-02 | 0.000 | JUN,HMGCS1,XPO1,FMO5,CYP2B6,PPARGC1A,ALDH3B1,ABCA1,SULT1E1,ALDH1A3,HS3ST1 |
| all ISCC versus paired controls | Bupropion Degradation | 5.34E-01 | 1.18E-01 |  | CYP4B1,CYP2B6 |
| all ISCC versus paired controls | PI3K/AKT Signaling | 5.26E-01 | 8E-02 | 2.121 | YWHAQ,PIK3CA,YWHAG,ITGB1,HSP90B1,PTGS2,HSP90AB1,YWHAB |
| all ISCC versus paired controls | RhoGDI Signaling | 5.25E-01 | 7.75E-02 | -1.414 | ARPC2,PAK2,CFL1,GNB2L1,ITGB1,MSN,GDI2,ACTR3,GNAI3,WASF1 |
| all ISCC versus paired controls | IL-6 Signaling | 5.07E-01 | 8.05E-02 | -0.378 | JUN,A2M,PIK3CA,SOCS3,MAPK10,COL1A1,CXCL8 |
| all ISCC versus paired controls | Polyamine Regulation in Colon Cancer | 5E-01 | 1.11E-01 |  | SAT1,ODC1 |
| all ISCC versus paired controls | Retinoate Biosynthesis I | 5E-01 | 1.11E-01 |  | ALDH1A3,RDH10 |
| all ISCC versus paired controls | Acetone Degradation I (to Methylglyoxal) | 5E-01 | 1.11E-01 |  | CYP4B1,CYP2B6 |
| all ISCC versus paired controls | Renal Cell Carcinoma Signaling | 5E-01 | 8.47E-02 | 0.000 | JUN,PAK2,PIK3CA,HIF1A,HGF |
| all ISCC versus paired controls | Bladder Cancer Signaling | 5E-01 | 8.47E-02 |  | CDK4,PA2G4,CXCL8,MMP14,PDGFC |
| all ISCC versus paired controls | Airway Pathology in Chronic Obstructive Pulmonary Disease | 4.89E-01 | 1.67E-01 |  | CXCL8 |
| all ISCC versus paired controls | Acyl-CoA Hydrolysis | 4.89E-01 | 1.67E-01 |  | PPT1 |
| all ISCC versus paired controls | Ketogenesis | 4.89E-01 | 1.67E-01 |  | HMGCS1 |
| all ISCC versus paired controls | Signaling by Rho Family GTPases | 4.84E-01 | 7.34E-02 | 1.265 | JUN,PAK2,GNB2L1,MAPK10,MSN,ACTR3,WASF1,GNAI3,ARPC2,CFL1,PIK3CA,ITGB1,STMN1 |
| all ISCC versus paired controls | Ephrin B Signaling | 4.83E-01 | 8.33E-02 |  | CFL1,ACP1,GNB2L1,CAP1,GNAI3 |
| all ISCC versus paired controls | Aldosterone Signaling in Epithelial Cells | 4.82E-01 | 7.63E-02 |  | HSPB11,DNAJC21,DNAJB13,PIK3CA,HSP90B1,HSPA5,DNAJB11,DNAJB6,HSP90AB1 |
| all ISCC versus paired controls | CCR5 Signaling in Macrophages | 4.8E-01 | 8.7E-02 |  | JUN,GNB2L1,MAPK10,GNAI3 |
| all ISCC versus paired controls | Role of JAK1 and JAK3 in γc Cytokine Signaling | 4.8E-01 | 8.7E-02 |  | PIK3CA,SOCS3,STAT1,IL7 |
| all ISCC versus paired controls | Germ Cell-Sertoli Cell Junction Signaling | 4.79E-01 | 7.52E-02 |  | TUBB4A,A2M,PAK2,CFL1,PIK3CA,ITGB1,MAPK10,ITGA6,JUP,TUBA4A |
| all ISCC versus paired controls | Ovarian Cancer Signaling | 4.74E-01 | 7.69E-02 |  | CDK4,PIK3CA,WNT5A,PTGS2,PA2G4,FZD7,GJA1,PDGFC |
| all ISCC versus paired controls | Hematopoiesis from Pluripotent Stem Cells | 4.68E-01 | 1.05E-01 |  | CXCL8,IL7 |
| all ISCC versus paired controls | PDGF Signaling | 4.66E-01 | 8.2E-02 | 0.447 | JUN,PIK3CA,ACP1,STAT1,PDGFC |
| all ISCC versus paired controls | Role of MAPK Signaling in the Pathogenesis of Influenza | 4.61E-01 | 8.51E-02 |  | CCL2,MAPK10,PTGS2,BAX |
| all ISCC versus paired controls | Unfolded protein response | 4.61E-01 | 8.51E-02 |  | CALR,HSP90B1,HSPA5,PPP1R15A |
| all ISCC versus paired controls | Oxidative Phosphorylation | 4.5E-01 | 8.06E-02 |  | COX7B,CYB5A,COX6B1,SDHA,NDUFB5 |
| all ISCC versus paired controls | Eicosanoid Signaling | 4.38E-01 | 8.82E-02 |  | PTGDS,PTGS2,ALOX15 |
| all ISCC versus paired controls | Actin Cytoskeleton Signaling | 4.36E-01 | 7.24E-02 |  | ARPC2,PAK2,FN1,CFL1,PIK3CA,ITGB1,MSN,ACTR3,WASF1,TMSB10/TMSB4X,PDGFC |
| all ISCC versus paired controls | Integrin Signaling | 4.36E-01 | 7.24E-02 | 3.000 | TSPAN3,ARPC2,PAK2,TSPAN6,PIK3CA,ITGAV,ITGB1,RALA,ITGA6,ACTR3,NCK1 |
| all ISCC versus paired controls | Hematopoiesis from Multipotent Stem Cells | 4.35E-01 | 1.43E-01 |  | IL7 |
| all ISCC versus paired controls | Calcium Transport I | 4.35E-01 | 1.43E-01 |  | ATP2C1 |
| all ISCC versus paired controls | Glycine Betaine Degradation | 4.35E-01 | 1.43E-01 |  | SHMT2 |
| all ISCC versus paired controls | Virus Entry via Endocytic Pathways | 4.35E-01 | 7.94E-02 |  | PIK3CA,ITGB1,ITGA6,CD55,TFRC |
| all ISCC versus paired controls | EIF2 Signaling | 4.26E-01 | 7.25E-02 | 2.000 | PPP1CC,RPL8,EIF2S3,PIK3CA,RPL18,EIF3H,PPP1R15A,EIF4A2,EIF4G1,RPL30 |
| all ISCC versus paired controls | Ephrin Receptor Signaling | 4.26E-01 | 7.25E-02 |  | ARPC2,PAK2,CFL1,ACP1,GNB2L1,ITGB1,ACTR3,NCK1,GNAI3,PDGFC |
| all ISCC versus paired controls | Angiopoietin Signaling | 4.26E-01 | 8.16E-02 |  | PAK2,PIK3CA,BIRC5,NCK1 |
| all ISCC versus paired controls | Growth Hormone Signaling | 4.26E-01 | 8.16E-02 | 1.000 | A2M,PIK3CA,SOCS3,STAT1 |
| all ISCC versus paired controls | Prostate Cancer Signaling | 4.2E-01 | 7.81E-02 |  | PIK3CA,HSP90B1,CDK2,PA2G4,HSP90AB1 |
| all ISCC versus paired controls | p70S6K Signaling | 4.16E-01 | 7.45E-02 | 2.000 | YWHAQ,PIK3CA,YWHAG,EEF2,YWHAB,GNAI3,BCAP31 |
| all ISCC versus paired controls | Mitochondrial Dysfunction | 4.15E-01 | 7.34E-02 |  | UCP2,COX7B,CYB5A,COX6B1,MAPK10,SDHA,NDUFB5,NDUFA4L2 |
| all ISCC versus paired controls | Neurotrophin/TRK Signaling | 4.09E-01 | 8E-02 |  | JUN,PIK3CA,NTRK2,SPRY1 |
| all ISCC versus paired controls | Macropinocytosis Signaling | 4.09E-01 | 8E-02 |  | PIK3CA,ITGB1,HGF,PDGFC |
| all ISCC versus paired controls | Sertoli Cell-Sertoli Cell Junction Signaling | 4.07E-01 | 7.14E-02 |  | JUN,TUBB4A,A2M,DLG1,CLDN3,ITGB1,MAPK10,JUP,CLDN4,TUBA4A |
| all ISCC versus paired controls | Role of Oct4 in Mammalian Embryonic Stem Cell Pluripotency | 3.98E-01 | 8.33E-02 |  | FOXA1,SPP1,ETS2 |
| all ISCC versus paired controls | Nicotine Degradation II | 3.98E-01 | 8.33E-02 |  | CYP4B1,FMO5,CYP2B6 |
| all ISCC versus paired controls | Dendritic Cell Maturation | 3.94E-01 | 7.21E-02 |  | COL10A1,PIK3CA,STAT1,MAPK10,COL3A1,COL1A2,COL1A1,FSCN1 |
| all ISCC versus paired controls | The Visual Cycle | 3.9E-01 | 1.25E-01 |  | RDH10 |
| all ISCC versus paired controls | Mevalonate Pathway I | 3.9E-01 | 1.25E-01 |  | HMGCS1 |
| all ISCC versus paired controls | Purine Nucleotides De Novo Biosynthesis II | 3.9E-01 | 1.25E-01 |  | GMPS |
| all ISCC versus paired controls | UDP-N-acetyl-D-galactosamine Biosynthesis II | 3.9E-01 | 1.25E-01 |  | GPI |
| all ISCC versus paired controls | Sonic Hedgehog Signaling | 3.88E-01 | 9.09E-02 |  | CDK1,CCNB1 |
| all ISCC versus paired controls | Serotonin Degradation | 3.8E-01 | 8.11E-02 |  | ADH1B,ADH6,ALDH1A3 |
| all ISCC versus paired controls | CCR3 Signaling in Eosinophils | 3.8E-01 | 7.32E-02 |  | PAK2,CFL1,PIK3CA,GNB2L1,CCL11,GNAI3 |
| all ISCC versus paired controls | Role of Osteoblasts, Osteoclasts and Chondrocytes in Rheumatoid Arthritis | 3.65E-01 | 6.88E-02 |  | JUN,DLX5,PIK3CA,WNT5A,ITGB1,MAPK10,COL1A1,SPP1,FZD7,MMP14,IL7 |
| all ISCC versus paired controls | Oncostatin M Signaling | 3.65E-01 | 8.7E-02 |  | STAT1,EPAS1 |
| all ISCC versus paired controls | Estrogen Biosynthesis | 3.65E-01 | 8.7E-02 |  | CYP4B1,CYP2B6 |
| all ISCC versus paired controls | Interferon Signaling | 3.65E-01 | 8.7E-02 |  | STAT1,BAX |
| all ISCC versus paired controls | JAK/Stat Signaling | 3.63E-01 | 7.55E-02 | 1.000 | JUN,PIK3CA,SOCS3,STAT1 |
| all ISCC versus paired controls | IL-12 Signaling and Production in Macrophages | 3.6E-01 | 7.07E-02 |  | JUN,RAB7A,PIK3CA,STAT1,CLU,MAPK10,ALOX15 |
| all ISCC versus paired controls | NRF2-mediated Oxidative Stress Response | 3.58E-01 | 6.92E-02 |  | JUN,DNAJA4,DNAJC21,DNAJB13,PIK3CA,DNAJB11,DNAJB6,STIP1,JUNB |
| all ISCC versus paired controls | LXR/RXR Activation | 3.56E-01 | 7.14E-02 | 0.447 | SCD,SAA2,CLU,CCL2,PTGS2,ABCA1 |
| all ISCC versus paired controls | nNOS Signaling in Skeletal Muscle Cells | 3.52E-01 | 1.11E-01 |  | DMD |
| all ISCC versus paired controls | Isoleucine Degradation I | 3.52E-01 | 1.11E-01 |  | BCAT1 |
| all ISCC versus paired controls | Colanic Acid Building Blocks Biosynthesis | 3.52E-01 | 1.11E-01 |  | GPI |
| all ISCC versus paired controls | BER pathway | 3.52E-01 | 1.11E-01 |  | PCNA |
| all ISCC versus paired controls | Renin-Angiotensin Signaling | 3.45E-01 | 7.06E-02 | -0.816 | JUN,PAK2,PIK3CA,STAT1,CCL2,MAPK10 |
| all ISCC versus paired controls | RhoA Signaling | 3.35E-01 | 6.98E-02 | 2.449 | ARPC2,CFL1,MSN,ANLN,ACTR3,WASF1 |
| all ISCC versus paired controls | Colorectal Cancer Metastasis Signaling | 3.3E-01 | 6.67E-02 | 0.905 | JUN,PIK3CA,GNB2L1,STAT1,WNT5A,MAPK10,PTGS2,BIRC5,BAX,FZD7,MMP14,PDGFC |
| all ISCC versus paired controls | Thrombopoietin Signaling | 3.3E-01 | 7.5E-02 |  | JUN,PIK3CA,STAT1 |
| all ISCC versus paired controls | Glutamate Receptor Signaling | 3.3E-01 | 7.5E-02 |  | SLC1A1,GRM7,SLC38A1 |
| all ISCC versus paired controls | tRNA Splicing | 3.23E-01 | 8E-02 |  | PDE8B,TSEN15 |
| all ISCC versus paired controls | LPS-stimulated MAPK Signaling | 3.22E-01 | 7.14E-02 | 0.000 | JUN,PIK3CA,ATF1,MAPK10 |
| all ISCC versus paired controls | Cleavage and Polyadenylation of Pre-mRNA | 3.19E-01 | 1E-01 |  | NUDT21 |
| all ISCC versus paired controls | DNA Double-Strand Break Repair by Non-Homologous End Joining | 3.19E-01 | 1E-01 |  | PRKDC |
| all ISCC versus paired controls | Chondroitin Sulfate Degradation (Metazoa) | 3.19E-01 | 1E-01 |  | GM2A |
| all ISCC versus paired controls | Histamine Degradation | 3.19E-01 | 1E-01 |  | ALDH1A3 |
| all ISCC versus paired controls | Superpathway of Geranylgeranyldiphosphate Biosynthesis I (via Mevalonate) | 3.19E-01 | 1E-01 |  | HMGCS1 |
| all ISCC versus paired controls | Telomerase Signaling | 3.18E-01 | 6.94E-02 | 0.447 | PIK3CA,HSP90B1,HSP90AB1,ELF3,ETS2 |
| all ISCC versus paired controls | Role of Hypercytokinemia/hyperchemokinemia in the Pathogenesis of Influenza | 3.05E-01 | 7.69E-02 |  | CCL2,CXCL8 |
| all ISCC versus paired controls | Inhibition of Angiogenesis by TSP1 | 3.05E-01 | 7.69E-02 |  | JUN,MAPK10 |
| all ISCC versus paired controls | D-myo-inositol (1,4,5,6)-Tetrakisphosphate Biosynthesis | 3.04E-01 | 6.74E-02 |  | PPP1CC,ACP1,SOCS3,EYA4,NUDT11,SET |
| all ISCC versus paired controls | D-myo-inositol (3,4,5,6)-tetrakisphosphate Biosynthesis | 3.04E-01 | 6.74E-02 |  | PPP1CC,ACP1,SOCS3,EYA4,NUDT11,SET |
| all ISCC versus paired controls | Lymphotoxin β Receptor Signaling | 3E-01 | 7.14E-02 |  | PIK3CA,CXCL1,VCAM1 |
| all ISCC versus paired controls | Dermatan Sulfate Degradation (Metazoa) | 2.9E-01 | 9.09E-02 |  | GM2A |
| all ISCC versus paired controls | Molecular Mechanisms of Cancer | 2.9E-01 | 6.4E-02 |  | JUN,PAK2,CDK4,CCND2,AURKA,WNT5A,MAPK10,CDK2,FZD7,BMPR1B,GNAI3,PIK3CA,ITGB1,HIF1A,RALA,PA2G4,BAX,PRKDC,CHEK1 |
| all ISCC versus paired controls | 4-1BB Signaling in T Lymphocytes | 2.87E-01 | 7.41E-02 |  | JUN,MAPK10 |
| all ISCC versus paired controls | Thyroid Cancer Signaling | 2.87E-01 | 7.41E-02 |  | CXCL1,NTRK2 |
| all ISCC versus paired controls | Semaphorin Signaling in Neurons | 2.87E-01 | 6.98E-02 |  | PAK2,CFL1,ITGB1 |
| all ISCC versus paired controls | NF-κB Activation by Viruses | 2.86E-01 | 6.78E-02 |  | PIK3CA,ITGAV,ITGB1,ITGA6 |
| all ISCC versus paired controls | Prolactin Signaling | 2.86E-01 | 6.78E-02 | 1.000 | JUN,PIK3CA,SOCS3,STAT1 |
| all ISCC versus paired controls | BMP signaling pathway | 2.75E-01 | 6.67E-02 |  | JUN,MAPK10,BMPR1B,GREM1 |
| all ISCC versus paired controls | EGF Signaling | 2.74E-01 | 6.82E-02 |  | JUN,PIK3CA,STAT1 |
| all ISCC versus paired controls | Cell Cycle Regulation by BTG Family Proteins | 2.71E-01 | 7.14E-02 |  | CDK4,CDK2 |
| all ISCC versus paired controls | Clathrin-mediated Endocytosis Signaling | 2.7E-01 | 6.38E-02 |  | ARPC2,RAB7A,SNAP91,PIK3CA,CLU,ITGB1,ACTR3,TFRC,PDGFC |
| all ISCC versus paired controls | Regulation of the Epithelial-Mesenchymal Transition Pathway | 2.7E-01 | 6.38E-02 |  | PIK3CA,SNAI2,JAG1,CLDN3,WNT5A,HIF1A,EGR1,HGF,FZD7 |
| all ISCC versus paired controls | CXCR4 Signaling | 2.69E-01 | 6.42E-02 | 0.000 | JUN,PAK2,PIK3CA,GNB2L1,MAPK10,EGR1,GNAI3 |
| all ISCC versus paired controls | 3-phosphoinositide Biosynthesis | 2.69E-01 | 6.42E-02 |  | PPP1CC,PIK3CA,ACP1,SOCS3,EYA4,NUDT11,SET |
| all ISCC versus paired controls | Tec Kinase Signaling | 2.69E-01 | 6.42E-02 | 1.342 | PAK2,PIK3CA,GNB2L1,STAT1,ITGB1,MAPK10,GNAI3 |
| all ISCC versus paired controls | Lipid Antigen Presentation by CD1 | 2.65E-01 | 8.33E-02 |  | CALR |
| all ISCC versus paired controls | γ-glutamyl Cycle | 2.65E-01 | 8.33E-02 |  | GGCT |
| all ISCC versus paired controls | Methionine Degradation I (to Homocysteine) | 2.65E-01 | 8.33E-02 |  | MAT1A |
| all ISCC versus paired controls | PXR/RXR Activation | 2.49E-01 | 6.52E-02 |  | CYP2B6,PPARGC1A,SCD |
| all ISCC versus paired controls | Glioma Invasiveness Signaling | 2.49E-01 | 6.52E-02 |  | PIK3CA,ITGAV,TIMP4 |
| all ISCC versus paired controls | fMLP Signaling in Neutrophils | 2.48E-01 | 6.33E-02 | 2.000 | ARPC2,PIK3CA,GNB2L1,ACTR3,GNAI3 |
| all ISCC versus paired controls | STAT3 Pathway | 2.44E-01 | 6.35E-02 |  | SOCS3,MAPK10,NTRK2,BMPR1B |
| all ISCC versus paired controls | CDP-diacylglycerol Biosynthesis I | 2.42E-01 | 7.69E-02 |  | CDS1 |
| all ISCC versus paired controls | NAD Salvage Pathway II | 2.42E-01 | 7.69E-02 |  | ACP1 |
| all ISCC versus paired controls | Tryptophan Degradation X (Mammalian, via Tryptamine) | 2.42E-01 | 7.69E-02 |  | ALDH1A3 |
| all ISCC versus paired controls | Parkinson's Signaling | 2.42E-01 | 7.69E-02 |  | UCHL1 |
| all ISCC versus paired controls | Melanoma Signaling | 2.41E-01 | 6.67E-02 |  | CDK4,PIK3CA |
| all ISCC versus paired controls | MSP-RON Signaling Pathway | 2.41E-01 | 6.67E-02 |  | PIK3CA,CCL2 |
| all ISCC versus paired controls | Nicotine Degradation III | 2.41E-01 | 6.67E-02 |  | CYP4B1,CYP2B6 |
| all ISCC versus paired controls | Non-Small Cell Lung Cancer Signaling | 2.38E-01 | 6.38E-02 |  | CDK4,PIK3CA,PA2G4 |
| all ISCC versus paired controls | ERK5 Signaling | 2.38E-01 | 6.38E-02 |  | YWHAQ,YWHAG,YWHAB |
| all ISCC versus paired controls | Role of PI3K/AKT Signaling in the Pathogenesis of Influenza | 2.38E-01 | 6.38E-02 |  | PIK3CA,PLAC8,GNAI3 |
| all ISCC versus paired controls | FcγRIIB Signaling in B Lymphocytes | 2.28E-01 | 6.45E-02 |  | PIK3CA,MAPK10 |
| all ISCC versus paired controls | April Mediated Signaling | 2.28E-01 | 6.45E-02 |  | JUN,MAPK10 |
| all ISCC versus paired controls | OX40 Signaling Pathway | 2.28E-01 | 6.45E-02 |  | JUN,MAPK10 |
| all ISCC versus paired controls | UVC-Induced MAPK Signaling | 2.28E-01 | 6.45E-02 |  | JUN,MAPK10 |
| all ISCC versus paired controls | Cardiomyocyte Differentiation via BMP Receptors | 2.22E-01 | 7.14E-02 |  | BMPR1B |
| all ISCC versus paired controls | Tumoricidal Function of Hepatic Natural Killer Cells | 2.22E-01 | 7.14E-02 |  | BAX |
| all ISCC versus paired controls | Valine Degradation I | 2.22E-01 | 7.14E-02 |  | BCAT1 |
| all ISCC versus paired controls | Oxidative Ethanol Degradation III | 2.22E-01 | 7.14E-02 |  | ALDH1A3 |
| all ISCC versus paired controls | Cysteine Biosynthesis III (mammalia) | 2.22E-01 | 7.14E-02 |  | MAT1A |
| all ISCC versus paired controls | Phosphatidylglycerol Biosynthesis II (Non-plastidic) | 2.04E-01 | 6.67E-02 |  | CDS1 |
| all ISCC versus paired controls | Retinol Biosynthesis | 2.04E-01 | 6.67E-02 |  | RDH10 |
| all ISCC versus paired controls | Ethanol Degradation IV | 2.04E-01 | 6.67E-02 |  | ALDH1A3 |
| all PSCC versus paired controls | Pyrimidine Deoxyribonucleotides De Novo Biosynthesis I | 3.54E00 | 2E-01 |  | RRM2,AK9,NME5 |
| all PSCC versus paired controls | Salvage Pathways of Pyrimidine Ribonucleotides | 2.6E00 | 6.25E-02 |  | AK9,SGK1,NME5,MAPK6 |
| all PSCC versus paired controls | CDK5 Signaling | 2.35E00 | 5.33E-02 |  | ITGA6,NTRK2,EGR1,MAPK6 |
| all PSCC versus paired controls | Cell Cycle: G2/M DNA Damage Checkpoint Regulation | 2.34E00 | 7.89E-02 |  | TOP2A,SFN,CKS2 |
| all PSCC versus paired controls | Role of Tissue Factor in Cancer | 2.22E00 | 4.88E-02 |  | CYR61,ITGA6,CTGF,EGR1 |
| all PSCC versus paired controls | Putrescine Biosynthesis III | 2.05E00 | 1E00 |  | ODC1 |
| all PSCC versus paired controls | Pyrimidine Ribonucleotides Interconversion | 1.87E00 | 1E-01 |  | AK9,NME5 |
| all PSCC versus paired controls | Pyrimidine Ribonucleotides De Novo Biosynthesis | 1.79E00 | 9.09E-02 |  | AK9,NME5 |
| all PSCC versus paired controls | Virus Entry via Endocytic Pathways | 1.73E00 | 4.76E-02 |  | CLTC,ITGA6,TFRC |
| all PSCC versus paired controls | NAD Biosynthesis III | 1.58E00 | 3.33E-01 |  | NAMPT |
| all PSCC versus paired controls | Germ Cell-Sertoli Cell Junction Signaling | 1.51E00 | 3.01E-02 |  | A2M,ITGA6,JUP,TUBA1B |
| all PSCC versus paired controls | IGF-1 Signaling | 1.49E00 | 3.85E-02 |  | SFN,CYR61,CTGF |
| all PSCC versus paired controls | Sertoli Cell-Sertoli Cell Junction Signaling | 1.44E00 | 2.86E-02 |  | A2M,DLG1,JUP,TUBA1B |
| all PSCC versus paired controls | Methylglyoxal Degradation III | 1.28E00 | 1.67E-01 |  | AKR1C1/AKR1C2 |
| all PSCC versus paired controls | Oleate Biosynthesis II (Animals) | 1.22E00 | 1.43E-01 |  | SCD |
| all PSCC versus paired controls | ERK5 Signaling | 1.19E00 | 4.26E-02 |  | SFN,SGK1 |
| all PSCC versus paired controls | Pyridoxal 5'-phosphate Salvage Pathway | 1.15E00 | 4.08E-02 |  | SGK1,MAPK6 |
| all PSCC versus paired controls | Role of IL-17A in Psoriasis | 1.07E00 | 1E-01 |  | S100A9 |
| all PSCC versus paired controls | Bile Acid Biosynthesis, Neutral Pathway | 1.03E00 | 9.09E-02 |  | AKR1C1/AKR1C2 |
| all PSCC versus paired controls | DNA damage-induced 14-3-3σ Signaling | 9.93E-01 | 8.33E-02 |  | SFN |
| all PSCC versus paired controls | HIPPO signaling | 9.02E-01 | 2.9E-02 |  | SFN,DLG1 |
| all PSCC versus paired controls | Polyamine Regulation in Colon Cancer | 8.28E-01 | 5.56E-02 |  | ODC1 |
| all PSCC versus paired controls | Inhibition of Matrix Metalloproteases | 6.99E-01 | 4E-02 |  | A2M |
| all PSCC versus paired controls | 14-3-3-mediated Signaling | 6.99E-01 | 2.15E-02 |  | SFN,TUBA1B |
| all PSCC versus paired controls | Thyroid Cancer Signaling | 6.69E-01 | 3.7E-02 |  | NTRK2 |
| all PSCC versus paired controls | Coagulation System | 6.42E-01 | 3.45E-02 |  | A2M |
| all PSCC versus paired controls | Epithelial Adherens Junction Signaling | 6.09E-01 | 1.87E-02 |  | JUP,TUBA1B |
| all PSCC versus paired controls | Nur77 Signaling in T Lymphocytes | 6.04E-01 | 3.12E-02 |  | NR4A1 |
| all PSCC versus paired controls | Calcium-induced T Lymphocyte Apoptosis | 5.41E-01 | 2.63E-02 |  | NR4A1 |
| all PSCC versus paired controls | Myc Mediated Apoptosis Signaling | 4.8E-01 | 2.22E-02 |  | SFN |
| all PSCC versus paired controls | PXR/RXR Activation | 4.72E-01 | 2.17E-02 |  | SCD |
| all PSCC versus paired controls | Growth Hormone Signaling | 4.5E-01 | 2.04E-02 |  | A2M |
| all PSCC versus paired controls | Clathrin-mediated Endocytosis Signaling | 4.47E-01 | 1.42E-02 |  | CLTC,TFRC |
| all PSCC versus paired controls | Acute Myeloid Leukemia Signaling | 4.43E-01 | 2E-02 |  | JUP |
| all PSCC versus paired controls | Neurotrophin/TRK Signaling | 4.43E-01 | 2E-02 |  | NTRK2 |
| all PSCC versus paired controls | Hepatic Fibrosis / Hepatic Stellate Cell Activation | 4.39E-01 | 1.4E-02 |  | A2M,CTGF |
| all PSCC versus paired controls | Caveolar-mediated Endocytosis Signaling | 4.36E-01 | 1.96E-02 |  | ITGA6 |
| all PSCC versus paired controls | Remodeling of Epithelial Adherens Junctions | 4.36E-01 | 1.96E-02 |  | TUBA1B |
| all PSCC versus paired controls | Agrin Interactions at Neuromuscular Junction | 3.99E-01 | 1.75E-02 |  | ITGA6 |
| all PSCC versus paired controls | CTLA4 Signaling in Cytotoxic T Lymphocytes | 3.93E-01 | 1.72E-02 |  | CLTC |
| all PSCC versus paired controls | NF-κB Activation by Viruses | 3.87E-01 | 1.69E-02 |  | ITGA6 |
| all PSCC versus paired controls | Oxidative Phosphorylation | 3.71E-01 | 1.61E-02 |  | COX6B1 |
| all PSCC versus paired controls | STAT3 Pathway | 3.66E-01 | 1.59E-02 |  | NTRK2 |
| all PSCC versus paired controls | Neuregulin Signaling | 3.56E-01 | 1.54E-02 |  | DCN |
| all PSCC versus paired controls | Huntington's Disease Signaling | 3.52E-01 | 1.18E-02 |  | SGK1,CLTC |
| all PSCC versus paired controls | Reelin Signaling in Neurons | 3.51E-01 | 1.52E-02 |  | ITGA6 |
| all PSCC versus paired controls | HIF1α Signaling | 3.46E-01 | 1.49E-02 |  | MAPK6 |
| all PSCC versus paired controls | TR/RXR Activation | 3.41E-01 | 1.47E-02 |  | AKR1C1/AKR1C2 |
| all PSCC versus paired controls | VEGF Signaling | 3.32E-01 | 1.43E-02 |  | SFN |
| all PSCC versus paired controls | Neuropathic Pain Signaling In Dorsal Horn Neurons | 3.19E-01 | 1.37E-02 |  | NTRK2 |
| all PSCC versus paired controls | p53 Signaling | 3.11E-01 | 1.33E-02 |  | SFN |
| all PSCC versus paired controls | Corticotropin Releasing Hormone Signaling | 3.03E-01 | 1.3E-02 |  | NR4A1 |
| all PSCC versus paired controls | phagosome maturation | 2.95E-01 | 1.27E-02 |  | TUBA1B |
| all PSCC versus paired controls | Paxillin Signaling | 2.8E-01 | 1.2E-02 |  | ITGA6 |
| all PSCC versus paired controls | LXR/RXR Activation | 2.77E-01 | 1.19E-02 |  | SCD |
| all PSCC versus paired controls | IL-6 Signaling | 2.67E-01 | 1.15E-02 |  | A2M |
| all PSCC versus paired controls | Axonal Guidance Signaling | 2.63E-01 | 9.35E-03 |  | ADAM23,TUBA1B,NTRK2 |
| all PSCC versus paired controls | Glucocorticoid Receptor Signaling | 2.52E-01 | 9.52E-03 |  | A2M,SGK1 |
| all PSCC versus paired controls | p70S6K Signaling | 2.45E-01 | 1.06E-02 |  | SFN |
| all PSCC versus paired controls | Hereditary Breast Cancer Signaling | 2.39E-01 | 1.04E-02 |  | SFN |
| all PSCC versus paired controls | PTEN Signaling | 2.36E-01 | 1.03E-02 |  | NTRK2 |
| all PSCC versus paired controls | Insulin Receptor Signaling | 2.31E-01 | 1.01E-02 |  | SGK1 |
| all PSCC versus paired controls | GNRH Signaling | 2.28E-01 | 1E-02 |  | EGR1 |
| all PSCC versus paired controls | PI3K/AKT Signaling | 2.28E-01 | 1E-02 |  | SFN |
| all PSCC versus paired controls | Human Embryonic Stem Cell Pluripotency | 2.15E-01 | 9.52E-03 |  | NTRK2 |
| all PSCC versus paired controls | Mitochondrial Dysfunction | 2.05E-01 | 9.17E-03 |  | COX6B1 |
| all PSCC versus paired controls | CXCR4 Signaling | 2.05E-01 | 9.17E-03 |  | EGR1 |
| all PSCC versus paired controls | Agranulocyte Adhesion and Diapedesis | 2.05E-01 | 9.17E-03 |  | ITGA6 |
| all PSCC versus paired controls | Granulocyte Adhesion and Diapedesis | 2E-01 | 9.01E-03 |  | ITGA6 |
| high-grade PSCC versus paired controls | Retinoic acid Mediated Apoptosis Signaling | 3.67E00 | 1.14E-01 | 2.236 | TNFSF10,IFNA17,PARP9,PARP14,DAP3 |
| high-grade PSCC versus paired controls | Salvage Pathways of Pyrimidine Ribonucleotides | 2.91E00 | 7.81E-02 |  | PAK2,AK9,SGK1,NME5,MAPK6 |
| high-grade PSCC versus paired controls | Germ Cell-Sertoli Cell Junction Signaling | 2.84E00 | 5.26E-02 |  | A2M,PAK2,CFL1,ACTG1,SORBS1,ITGA6,TUBA1B |
| high-grade PSCC versus paired controls | CDK5 Signaling | 2.6E00 | 6.67E-02 | -1.000 | ITGA6,FOSB,NTRK2,EGR1,MAPK6 |
| high-grade PSCC versus paired controls | Role of Tissue Factor in Cancer | 2.43E00 | 6.1E-02 |  | CFL1,CYR61,ITGA6,CTGF,EGR1 |
| high-grade PSCC versus paired controls | Virus Entry via Endocytic Pathways | 2.09E00 | 6.35E-02 |  | ACTG1,CLTC,ITGA6,TFRC |
| high-grade PSCC versus paired controls | Putrescine Biosynthesis III | 1.9E00 | 1E00 |  | ODC1 |
| high-grade PSCC versus paired controls | Death Receptor Signaling | 1.89E00 | 5.56E-02 | 2.000 | ACTG1,TNFSF10,PARP9,PARP14 |
| high-grade PSCC versus paired controls | Pyrimidine Deoxyribonucleotides De Novo Biosynthesis I | 1.83E00 | 1.33E-01 |  | AK9,NME5 |
| high-grade PSCC versus paired controls | Pyridoxal 5'-phosphate Salvage Pathway | 1.63E00 | 6.12E-02 |  | PAK2,SGK1,MAPK6 |
| high-grade PSCC versus paired controls | Pyrimidine Ribonucleotides Interconversion | 1.59E00 | 1E-01 |  | AK9,NME5 |
| high-grade PSCC versus paired controls | Pyrimidine Ribonucleotides De Novo Biosynthesis | 1.51E00 | 9.09E-02 |  | AK9,NME5 |
| high-grade PSCC versus paired controls | Sertoli Cell-Sertoli Cell Junction Signaling | 1.5E00 | 3.57E-02 |  | A2M,DLG1,ACTG1,SORBS1,TUBA1B |
| high-grade PSCC versus paired controls | Agrin Interactions at Neuromuscular Junction | 1.46E00 | 5.26E-02 |  | PAK2,ACTG1,ITGA6 |
| high-grade PSCC versus paired controls | NAD Biosynthesis III | 1.43E00 | 3.33E-01 |  | NAMPT |
| high-grade PSCC versus paired controls | Oxidative Phosphorylation | 1.37E00 | 4.84E-02 |  | COX6B1,COX7A2L,NDUFB5 |
| high-grade PSCC versus paired controls | Epithelial Adherens Junction Signaling | 1.34E00 | 3.74E-02 |  | ACTG1,SORBS1,RAP1B,TUBA1B |
| high-grade PSCC versus paired controls | Agranulocyte Adhesion and Diapedesis | 1.31E00 | 3.67E-02 |  | CXCL9,ACTG1,ITGA6,XCL1 |
| high-grade PSCC versus paired controls | GDP-mannose Biosynthesis | 1.21E00 | 2E-01 |  | GPI |
| high-grade PSCC versus paired controls | Eicosanoid Signaling | 1.17E00 | 5.88E-02 |  | PRDX6,ALOX15 |
| high-grade PSCC versus paired controls | Methylglyoxal Degradation III | 1.14E00 | 1.67E-01 |  | AKR1C1/AKR1C2 |
| high-grade PSCC versus paired controls | Pathogenesis of Multiple Sclerosis | 1.07E00 | 1.43E-01 |  | CXCL9 |
| high-grade PSCC versus paired controls | Oleate Biosynthesis II (Animals) | 1.07E00 | 1.43E-01 |  | SCD |
| high-grade PSCC versus paired controls | Paxillin Signaling | 1.06E00 | 3.61E-02 |  | PAK2,ACTG1,ITGA6 |
| high-grade PSCC versus paired controls | UDP-N-acetyl-D-galactosamine Biosynthesis II | 1.02E00 | 1.25E-01 |  | GPI |
| high-grade PSCC versus paired controls | Ephrin Receptor Signaling | 1.02E00 | 2.9E-02 |  | PAK2,CFL1,SORBS1,RAP1B |
| high-grade PSCC versus paired controls | Semaphorin Signaling in Neurons | 9.93E-01 | 4.65E-02 |  | PAK2,CFL1 |
| high-grade PSCC versus paired controls | Hepatic Fibrosis / Hepatic Stellate Cell Activation | 9.74E-01 | 2.8E-02 |  | CXCL9,A2M,TNFSF10,CTGF |
| high-grade PSCC versus paired controls | Colanic Acid Building Blocks Biosynthesis | 9.68E-01 | 1.11E-01 |  | GPI |
| high-grade PSCC versus paired controls | Triacylglycerol Degradation | 9.25E-01 | 1E-01 |  | PRDX6 |
| high-grade PSCC versus paired controls | Integrin Signaling | 9.03E-01 | 2.63E-02 | 2.000 | PAK2,ACTG1,ITGA6,RAP1B |
| high-grade PSCC versus paired controls | Bile Acid Biosynthesis, Neutral Pathway | 8.86E-01 | 9.09E-02 |  | AKR1C1/AKR1C2 |
| high-grade PSCC versus paired controls | Caveolar-mediated Endocytosis Signaling | 8.71E-01 | 3.92E-02 |  | ACTG1,ITGA6 |
| high-grade PSCC versus paired controls | Crosstalk between Dendritic Cells and Natural Killer Cells | 8.71E-01 | 3.92E-02 |  | ACTG1,TNFSF10 |
| high-grade PSCC versus paired controls | Remodeling of Epithelial Adherens Junctions | 8.71E-01 | 3.92E-02 |  | ACTG1,TUBA1B |
| high-grade PSCC versus paired controls | Gluconeogenesis I | 8.19E-01 | 7.69E-02 |  | GPI |
| high-grade PSCC versus paired controls | Parkinson's Signaling | 8.19E-01 | 7.69E-02 |  | UCHL1 |
| high-grade PSCC versus paired controls | Mitochondrial Dysfunction | 8.01E-01 | 2.75E-02 |  | COX6B1,COX7A2L,NDUFB5 |
| high-grade PSCC versus paired controls | Tec Kinase Signaling | 8.01E-01 | 2.75E-02 |  | PAK2,ACTG1,TNFSF10 |
| high-grade PSCC versus paired controls | Role of Lipids/Lipid Rafts in the Pathogenesis of Influenza | 7.89E-01 | 7.14E-02 |  | IFNA17 |
| high-grade PSCC versus paired controls | Telomere Extension by Telomerase | 7.89E-01 | 7.14E-02 |  | HNRNPA1 |
| high-grade PSCC versus paired controls | Glutathione Redox Reactions I | 7.89E-01 | 7.14E-02 |  | PRDX6 |
| high-grade PSCC versus paired controls | Granulocyte Adhesion and Diapedesis | 7.85E-01 | 2.7E-02 |  | CXCL9,ITGA6,XCL1 |
| high-grade PSCC versus paired controls | Glycolysis I | 7.62E-01 | 6.67E-02 |  | GPI |
| high-grade PSCC versus paired controls | Calcium Signaling | 7.46E-01 | 2.59E-02 |  | TP63,RAP1B,TPM2 |
| high-grade PSCC versus paired controls | Regulation of Actin-based Motility by Rho | 7.37E-01 | 3.23E-02 |  | PAK2,CFL1 |
| high-grade PSCC versus paired controls | Polyamine Regulation in Colon Cancer | 6.91E-01 | 5.56E-02 |  | ODC1 |
| high-grade PSCC versus paired controls | UVA-Induced MAPK Signaling | 6.86E-01 | 2.99E-02 |  | PARP9,PARP14 |
| high-grade PSCC versus paired controls | TR/RXR Activation | 6.76E-01 | 2.94E-02 |  | TBL1XR1,AKR1C1/AKR1C2 |
| high-grade PSCC versus paired controls | FAK Signaling | 6.76E-01 | 2.94E-02 |  | PAK2,ACTG1 |
| high-grade PSCC versus paired controls | HIPPO signaling | 6.67E-01 | 2.9E-02 |  | DLG1,MOB1A |
| high-grade PSCC versus paired controls | Axonal Guidance Signaling | 6.62E-01 | 1.87E-02 |  | PAK2,CFL1,ADAM23,RAP1B,TUBA1B,NTRK2 |
| high-grade PSCC versus paired controls | RhoGDI Signaling | 6.55E-01 | 2.33E-02 |  | PAK2,CFL1,ACTG1 |
| high-grade PSCC versus paired controls | PAK Signaling | 6.49E-01 | 2.82E-02 |  | PAK2,CFL1 |
| high-grade PSCC versus paired controls | ILK Signaling | 6.48E-01 | 2.31E-02 |  | CFL1,ACTG1,DSP |
| high-grade PSCC versus paired controls | p53 Signaling | 6.14E-01 | 2.67E-02 |  | TP63,PERP |
| high-grade PSCC versus paired controls | EIF2 Signaling | 6E-01 | 2.17E-02 |  | EIF4A2,RPL41,RPSA |
| high-grade PSCC versus paired controls | Corticotropin Releasing Hormone Signaling | 5.98E-01 | 2.6E-02 |  | NR4A1,RAP1B |
| high-grade PSCC versus paired controls | IGF-1 Signaling | 5.9E-01 | 2.56E-02 |  | CYR61,CTGF |
| high-grade PSCC versus paired controls | Leukocyte Extravasation Signaling | 5.82E-01 | 2.13E-02 |  | ACTG1,ITGA6,RAP1B |
| high-grade PSCC versus paired controls | Clathrin-mediated Endocytosis Signaling | 5.82E-01 | 2.13E-02 |  | ACTG1,CLTC,TFRC |
| high-grade PSCC versus paired controls | B Cell Receptor Signaling | 5.82E-01 | 2.13E-02 |  | CFL1,RAP1B,EGR1 |
| high-grade PSCC versus paired controls | phagosome maturation | 5.82E-01 | 2.53E-02 |  | PRDX6,TUBA1B |
| high-grade PSCC versus paired controls | Atherosclerosis Signaling | 5.74E-01 | 2.5E-02 |  | PRDX6,ALOX15 |
| high-grade PSCC versus paired controls | Inhibition of Matrix Metalloproteases | 5.66E-01 | 4E-02 |  | A2M |
| high-grade PSCC versus paired controls | CCR3 Signaling in Eosinophils | 5.59E-01 | 2.44E-02 |  | PAK2,CFL1 |
| high-grade PSCC versus paired controls | Role of Hypercytokinemia/hyperchemokinemia in the Pathogenesis of Influenza | 5.52E-01 | 3.85E-02 |  | IFNA17 |
| high-grade PSCC versus paired controls | Docosahexaenoic Acid (DHA) Signaling | 5.38E-01 | 3.7E-02 |  | ALOX15 |
| high-grade PSCC versus paired controls | Thyroid Cancer Signaling | 5.38E-01 | 3.7E-02 |  | NTRK2 |
| high-grade PSCC versus paired controls | Cdc42 Signaling | 5.38E-01 | 2.35E-02 |  | PAK2,CFL1 |
| high-grade PSCC versus paired controls | Rac Signaling | 5.31E-01 | 2.33E-02 |  | PAK2,CFL1 |
| high-grade PSCC versus paired controls | RhoA Signaling | 5.31E-01 | 2.33E-02 |  | CFL1,ACTG1 |
| high-grade PSCC versus paired controls | Mechanisms of Viral Exit from Host Cells | 5.25E-01 | 3.57E-02 |  | ACTG1 |
| high-grade PSCC versus paired controls | Actin Cytoskeleton Signaling | 5.24E-01 | 1.97E-02 |  | PAK2,CFL1,ACTG1 |
| high-grade PSCC versus paired controls | Coagulation System | 5.12E-01 | 3.45E-02 |  | A2M |
| high-grade PSCC versus paired controls | Role of Cytokines in Mediating Communication between Immune Cells | 5.12E-01 | 3.45E-02 |  | IFNA17 |
| high-grade PSCC versus paired controls | MSP-RON Signaling Pathway | 5E-01 | 3.33E-02 |  | ACTG1 |
| high-grade PSCC versus paired controls | Nur77 Signaling in T Lymphocytes | 4.77E-01 | 3.12E-02 |  | NR4A1 |
| high-grade PSCC versus paired controls | Role of RIG1-like Receptors in Antiviral Innate Immunity | 4.56E-01 | 2.94E-02 |  | IFNA17 |
| high-grade PSCC versus paired controls | Phospholipases | 4.56E-01 | 2.94E-02 |  | PRDX6 |
| high-grade PSCC versus paired controls | IL-12 Signaling and Production in Macrophages | 4.5E-01 | 2.02E-02 |  | IFNA17,ALOX15 |
| high-grade PSCC versus paired controls | GNRH Signaling | 4.45E-01 | 2E-02 |  | PAK2,EGR1 |
| high-grade PSCC versus paired controls | Role of Oct4 in Mammalian Embryonic Stem Cell Pluripotency | 4.36E-01 | 2.78E-02 |  | SPP1 |
| high-grade PSCC versus paired controls | Heparan Sulfate Biosynthesis (Late Stages) | 4.36E-01 | 2.78E-02 |  | PRDX6 |
| high-grade PSCC versus paired controls | Calcium-induced T Lymphocyte Apoptosis | 4.18E-01 | 2.63E-02 |  | NR4A1 |
| high-grade PSCC versus paired controls | Cell Cycle: G2/M DNA Damage Checkpoint Regulation | 4.18E-01 | 2.63E-02 |  | TOP2A |
| high-grade PSCC versus paired controls | Signaling by Rho Family GTPases | 4.14E-01 | 1.69E-02 |  | PAK2,CFL1,ACTG1 |
| high-grade PSCC versus paired controls | Ephrin A Signaling | 4.09E-01 | 2.56E-02 |  | CFL1 |
| high-grade PSCC versus paired controls | TNFR1 Signaling | 4E-01 | 2.5E-02 |  | PAK2 |
| high-grade PSCC versus paired controls | CXCR4 Signaling | 3.98E-01 | 1.83E-02 |  | PAK2,EGR1 |
| high-grade PSCC versus paired controls | Antiproliferative Role of Somatostatin Receptor 2 | 3.84E-01 | 2.38E-02 |  | RAP1B |
| high-grade PSCC versus paired controls | Endothelin-1 Signaling | 3.8E-01 | 1.77E-02 |  | PRDX6,MAPK6 |
| high-grade PSCC versus paired controls | Gap Junction Signaling | 3.8E-01 | 1.77E-02 |  | ACTG1,TUBA1B |
| high-grade PSCC versus paired controls | Heparan Sulfate Biosynthesis | 3.76E-01 | 2.33E-02 |  | PRDX6 |
| high-grade PSCC versus paired controls | Regulation of eIF4 and p70S6K Signaling | 3.66E-01 | 1.72E-02 |  | EIF4A2,RPSA |
| high-grade PSCC versus paired controls | PXR/RXR Activation | 3.55E-01 | 2.17E-02 |  | SCD |
| high-grade PSCC versus paired controls | ERK5 Signaling | 3.48E-01 | 2.13E-02 |  | SGK1 |
| high-grade PSCC versus paired controls | Role of MAPK Signaling in the Pathogenesis of Influenza | 3.48E-01 | 2.13E-02 |  | PRDX6 |
| high-grade PSCC versus paired controls | Role of PI3K/AKT Signaling in the Pathogenesis of Influenza | 3.48E-01 | 2.13E-02 |  | IFNA17 |
| high-grade PSCC versus paired controls | Activation of IRF by Cytosolic Pattern Recognition Receptors | 3.35E-01 | 2.04E-02 |  | IFNA17 |
| high-grade PSCC versus paired controls | Angiopoietin Signaling | 3.35E-01 | 2.04E-02 |  | PAK2 |
| high-grade PSCC versus paired controls | Growth Hormone Signaling | 3.35E-01 | 2.04E-02 |  | A2M |
| high-grade PSCC versus paired controls | Altered T Cell and B Cell Signaling in Rheumatoid Arthritis | 3.28E-01 | 2E-02 |  | SPP1 |
| high-grade PSCC versus paired controls | Neurotrophin/TRK Signaling | 3.28E-01 | 2E-02 |  | NTRK2 |
| high-grade PSCC versus paired controls | Chemokine Signaling | 3.22E-01 | 1.96E-02 |  | CFL1 |
| high-grade PSCC versus paired controls | GABA Receptor Signaling | 3.16E-01 | 1.92E-02 |  | GABRE |
| high-grade PSCC versus paired controls | VDR/RXR Activation | 3.1E-01 | 1.89E-02 |  | SPP1 |
| high-grade PSCC versus paired controls | Systemic Lupus Erythematosus Signaling | 3E-01 | 1.5E-02 |  | LSM2,IFNA17 |
| high-grade PSCC versus paired controls | CTLA4 Signaling in Cytotoxic T Lymphocytes | 2.83E-01 | 1.72E-02 |  | CLTC |
| high-grade PSCC versus paired controls | NF-κB Activation by Viruses | 2.78E-01 | 1.69E-02 |  | ITGA6 |
| high-grade PSCC versus paired controls | Renal Cell Carcinoma Signaling | 2.78E-01 | 1.69E-02 |  | PAK2 |
| high-grade PSCC versus paired controls | ERK/MAPK Signaling | 2.74E-01 | 1.42E-02 |  | PAK2,RAP1B |
| high-grade PSCC versus paired controls | Role of Wnt/GSK-3β Signaling in the Pathogenesis of Influenza | 2.73E-01 | 1.67E-02 |  | IFNA17 |
| high-grade PSCC versus paired controls | Ephrin B Signaling | 2.73E-01 | 1.67E-02 |  | CFL1 |
| high-grade PSCC versus paired controls | Antioxidant Action of Vitamin C | 2.73E-01 | 1.67E-02 |  | PRDX6 |
| high-grade PSCC versus paired controls | mTOR Signaling | 2.62E-01 | 1.38E-02 |  | EIF4A2,RPSA |
| high-grade PSCC versus paired controls | STAT3 Pathway | 2.59E-01 | 1.59E-02 |  | NTRK2 |
| high-grade PSCC versus paired controls | Neuregulin Signaling | 2.5E-01 | 1.54E-02 |  | DCN |
| high-grade PSCC versus paired controls | Fcγ Receptor-mediated Phagocytosis in Macrophages and Monocytes | 2.46E-01 | 1.52E-02 |  | ACTG1 |
| high-grade PSCC versus paired controls | Reelin Signaling in Neurons | 2.46E-01 | 1.52E-02 |  | ITGA6 |
| high-grade PSCC versus paired controls | ErbB Signaling | 2.46E-01 | 1.52E-02 |  | PAK2 |
| high-grade PSCC versus paired controls | HIF1α Signaling | 2.42E-01 | 1.49E-02 |  | MAPK6 |
| high-grade PSCC versus paired controls | Nitric Oxide Signaling in the Cardiovascular System | 2.38E-01 | 1.47E-02 |  | PLN |
| high-grade PSCC versus paired controls | VEGF Signaling | 2.3E-01 | 1.43E-02 |  | ACTG1 |
| high-grade PSCC versus paired controls | Neuropathic Pain Signaling In Dorsal Horn Neurons | 2.19E-01 | 1.37E-02 |  | NTRK2 |
| high-grade PSCC versus paired controls | Natural Killer Cell Signaling | 2.15E-01 | 1.35E-02 |  | PAK2 |
| high-grade PSCC versus paired controls | Sperm Motility | 2.15E-01 | 1.35E-02 |  | PRDX6 |
| high-grade PSCC versus paired controls | Cellular Effects of Sildenafil (Viagra) | 2.05E-01 | 1.3E-02 |  | ACTG1 |
| low-grade PSCC versus paired controls | Role of IL-17A in Psoriasis | 4.15E00 | 3E-01 |  | S100A9,CXCL1,CXCL8 |
| low-grade PSCC versus paired controls | Oleate Biosynthesis II (Animals) | 2.83E00 | 2.86E-01 |  | CYB5A,SCD |
| low-grade PSCC versus paired controls | Role of Tissue Factor in Cancer | 2.27E00 | 4.88E-02 |  | ITGA6,CXCL1,CTGF,CXCL8 |
| low-grade PSCC versus paired controls | Pyrimidine Deoxyribonucleotides De Novo Biosynthesis I | 2.15E00 | 1.33E-01 |  | RRM2,AK9 |
| low-grade PSCC versus paired controls | IL-17A Signaling in Gastric Cells | 1.95E00 | 1.05E-01 |  | CXCL1,CXCL8 |
| low-grade PSCC versus paired controls | Agranulocyte Adhesion and Diapedesis | 1.85E00 | 3.67E-02 |  | IL1RN,ITGA6,CXCL1,CXCL8 |
| low-grade PSCC versus paired controls | Granulocyte Adhesion and Diapedesis | 1.82E00 | 3.6E-02 |  | IL1RN,ITGA6,CXCL1,CXCL8 |
| low-grade PSCC versus paired controls | Role of Hypercytokinemia/hyperchemokinemia in the Pathogenesis of Influenza | 1.68E00 | 7.69E-02 |  | IL1RN,CXCL8 |
| low-grade PSCC versus paired controls | Role of IL-17F in Allergic Inflammatory Airway Diseases | 1.62E00 | 7.14E-02 |  | CXCL1,CXCL8 |
| low-grade PSCC versus paired controls | Role of Cytokines in Mediating Communication between Immune Cells | 1.59E00 | 6.9E-02 |  | IL1RN,CXCL8 |
| low-grade PSCC versus paired controls | Germ Cell-Sertoli Cell Junction Signaling | 1.56E00 | 3.01E-02 |  | A2M,ITGA6,JUP,TUBA1B |
| low-grade PSCC versus paired controls | IL-6 Signaling | 1.41E00 | 3.45E-02 |  | A2M,IL1RN,CXCL8 |
| low-grade PSCC versus paired controls | Cell Cycle: G2/M DNA Damage Checkpoint Regulation | 1.38E00 | 5.26E-02 |  | TOP2A,SFN |
| low-grade PSCC versus paired controls | Role of IL-17A in Arthritis | 1.36E00 | 5.13E-02 |  | CXCL1,CXCL8 |
| low-grade PSCC versus paired controls | Airway Pathology in Chronic Obstructive Pulmonary Disease | 1.3E00 | 1.67E-01 |  | CXCL8 |
| low-grade PSCC versus paired controls | Methylglyoxal Degradation III | 1.3E00 | 1.67E-01 |  | AKR1C1/AKR1C2 |
| low-grade PSCC versus paired controls | Communication between Innate and Adaptive Immune Cells | 1.28E00 | 4.65E-02 |  | IL1RN,CXCL8 |
| low-grade PSCC versus paired controls | ERK5 Signaling | 1.21E00 | 4.26E-02 |  | SFN,SGK1 |
| low-grade PSCC versus paired controls | IL-17 Signaling | 1.09E00 | 3.64E-02 |  | CXCL1,CXCL8 |
| low-grade PSCC versus paired controls | Differential Regulation of Cytokine Production in Macrophages and T Helper Cells by IL-17A and IL-17F | 1.04E00 | 9.09E-02 |  | CXCL1 |
| low-grade PSCC versus paired controls | Bile Acid Biosynthesis, Neutral Pathway | 1.04E00 | 9.09E-02 |  | AKR1C1/AKR1C2 |
| low-grade PSCC versus paired controls | DNA damage-induced 14-3-3σ Signaling | 1.01E00 | 8.33E-02 |  | SFN |
| low-grade PSCC versus paired controls | Salvage Pathways of Pyrimidine Ribonucleotides | 9.82E-01 | 3.12E-02 |  | AK9,SGK1 |
| low-grade PSCC versus paired controls | γ-linolenate Biosynthesis II (Animals) | 9.75E-01 | 7.69E-02 |  | CYB5A |
| low-grade PSCC versus paired controls | Glucocorticoid Receptor Signaling | 9.74E-01 | 1.9E-02 |  | A2M,IL1RN,SGK1,CXCL8 |
| low-grade PSCC versus paired controls | Sertoli Cell-Sertoli Cell Junction Signaling | 9.25E-01 | 2.14E-02 |  | A2M,JUP,TUBA1B |
| low-grade PSCC versus paired controls | Differential Regulation of Cytokine Production in Intestinal Epithelial Cells by IL-17A and IL-17F | 9.17E-01 | 6.67E-02 |  | CXCL1 |
| low-grade PSCC versus paired controls | Hepatic Fibrosis / Hepatic Stellate Cell Activation | 9.05E-01 | 2.1E-02 |  | A2M,CTGF,CXCL8 |
| low-grade PSCC versus paired controls | p53 Signaling | 8.69E-01 | 2.67E-02 |  | SERPINB5,SFN |
| low-grade PSCC versus paired controls | IGF-1 Signaling | 8.41E-01 | 2.56E-02 |  | SFN,CTGF |
| low-grade PSCC versus paired controls | Atherosclerosis Signaling | 8.24E-01 | 2.5E-02 |  | IL1RN,CXCL8 |
| low-grade PSCC versus paired controls | p38 MAPK Signaling | 8.24E-01 | 2.5E-02 |  | HIST1H3C,IL1RN |
| low-grade PSCC versus paired controls | Hematopoiesis from Pluripotent Stem Cells | 8.21E-01 | 5.26E-02 |  | CXCL8 |
| low-grade PSCC versus paired controls | Pyrimidine Ribonucleotides Interconversion | 8.01E-01 | 5E-02 |  | AK9 |
| low-grade PSCC versus paired controls | LXR/RXR Activation | 7.91E-01 | 2.38E-02 |  | IL1RN,SCD |
| low-grade PSCC versus paired controls | Pyrimidine Ribonucleotides De Novo Biosynthesis | 7.63E-01 | 4.55E-02 |  | AK9 |
| low-grade PSCC versus paired controls | 14-3-3-mediated Signaling | 7.22E-01 | 2.15E-02 |  | SFN,TUBA1B |
| low-grade PSCC versus paired controls | Graft-versus-Host Disease Signaling | 7.13E-01 | 4E-02 |  | IL1RN |
| low-grade PSCC versus paired controls | Inhibition of Matrix Metalloproteases | 7.13E-01 | 4E-02 |  | A2M |
| low-grade PSCC versus paired controls | Thyroid Cancer Signaling | 6.83E-01 | 3.7E-02 |  | CXCL1 |
| low-grade PSCC versus paired controls | Hepatic Cholestasis | 6.69E-01 | 1.98E-02 |  | IL1RN,CXCL8 |
| low-grade PSCC versus paired controls | Coagulation System | 6.55E-01 | 3.45E-02 |  | A2M |
| low-grade PSCC versus paired controls | Epithelial Adherens Junction Signaling | 6.32E-01 | 1.87E-02 |  | JUP,TUBA1B |
| low-grade PSCC versus paired controls | UVB-Induced MAPK Signaling | 5.44E-01 | 2.56E-02 |  | HIST1H3C |
| low-grade PSCC versus paired controls | Lymphotoxin β Receptor Signaling | 5.17E-01 | 2.38E-02 |  | CXCL1 |
| low-grade PSCC versus paired controls | Acute Phase Response Signaling | 5.09E-01 | 1.53E-02 |  | A2M,IL1RN |
| low-grade PSCC versus paired controls | IL-10 Signaling | 5.01E-01 | 2.27E-02 |  | IL1RN |
| low-grade PSCC versus paired controls | Myc Mediated Apoptosis Signaling | 4.93E-01 | 2.22E-02 |  | SFN |
| low-grade PSCC versus paired controls | PXR/RXR Activation | 4.85E-01 | 2.17E-02 |  | SCD |
| low-grade PSCC versus paired controls | TREM1 Signaling | 4.7E-01 | 2.08E-02 |  | CXCL8 |
| low-grade PSCC versus paired controls | IL-15 Signaling | 4.63E-01 | 2.04E-02 |  | CXCL8 |
| low-grade PSCC versus paired controls | Growth Hormone Signaling | 4.63E-01 | 2.04E-02 |  | A2M |
| low-grade PSCC versus paired controls | Pyridoxal 5'-phosphate Salvage Pathway | 4.63E-01 | 2.04E-02 |  | SGK1 |
| low-grade PSCC versus paired controls | Acute Myeloid Leukemia Signaling | 4.56E-01 | 2E-02 |  | JUP |
| low-grade PSCC versus paired controls | Altered T Cell and B Cell Signaling in Rheumatoid Arthritis | 4.56E-01 | 2E-02 |  | IL1RN |
| low-grade PSCC versus paired controls | Caveolar-mediated Endocytosis Signaling | 4.49E-01 | 1.96E-02 |  | ITGA6 |
| low-grade PSCC versus paired controls | IL-17A Signaling in Airway Cells | 4.49E-01 | 1.96E-02 |  | CXCL1 |
| low-grade PSCC versus paired controls | Remodeling of Epithelial Adherens Junctions | 4.49E-01 | 1.96E-02 |  | TUBA1B |
| low-grade PSCC versus paired controls | Toll-like Receptor Signaling | 4.49E-01 | 1.96E-02 |  | IL1RN |
| low-grade PSCC versus paired controls | IL-8 Signaling | 4.48E-01 | 1.37E-02 |  | CXCL1,CXCL8 |
| low-grade PSCC versus paired controls | Agrin Interactions at Neuromuscular Junction | 4.11E-01 | 1.75E-02 |  | ITGA6 |
| low-grade PSCC versus paired controls | NF-κB Activation by Viruses | 3.99E-01 | 1.69E-02 |  | ITGA6 |
| low-grade PSCC versus paired controls | Bladder Cancer Signaling | 3.99E-01 | 1.69E-02 |  | CXCL8 |
| low-grade PSCC versus paired controls | Oxidative Phosphorylation | 3.83E-01 | 1.61E-02 |  | CYB5A |
| low-grade PSCC versus paired controls | Virus Entry via Endocytic Pathways | 3.77E-01 | 1.59E-02 |  | ITGA6 |
| low-grade PSCC versus paired controls | Reelin Signaling in Neurons | 3.62E-01 | 1.52E-02 |  | ITGA6 |
| low-grade PSCC versus paired controls | TR/RXR Activation | 3.53E-01 | 1.47E-02 |  | AKR1C1/AKR1C2 |
| low-grade PSCC versus paired controls | PPAR Signaling | 3.53E-01 | 1.47E-02 |  | IL1RN |
| low-grade PSCC versus paired controls | HIPPO signaling | 3.48E-01 | 1.45E-02 |  | SFN |
| low-grade PSCC versus paired controls | VEGF Signaling | 3.43E-01 | 1.43E-02 |  | SFN |
| low-grade PSCC versus paired controls | CDK5 Signaling | 3.22E-01 | 1.33E-02 |  | ITGA6 |
| low-grade PSCC versus paired controls | Corticotropin Releasing Hormone Signaling | 3.14E-01 | 1.3E-02 |  | IVL |
| low-grade PSCC versus paired controls | Cholecystokinin/Gastrin-mediated Signaling | 3.14E-01 | 1.3E-02 |  | IL1RN |
| low-grade PSCC versus paired controls | phagosome maturation | 3.06E-01 | 1.27E-02 |  | TUBA1B |
| low-grade PSCC versus paired controls | Paxillin Signaling | 2.91E-01 | 1.2E-02 |  | ITGA6 |
| low-grade PSCC versus paired controls | Role of Pattern Recognition Receptors in Recognition of Bacteria and Viruses | 2.87E-01 | 1.19E-02 |  | CXCL8 |
| low-grade PSCC versus paired controls | HMGB1 Signaling | 2.87E-01 | 1.19E-02 |  | CXCL8 |
| low-grade PSCC versus paired controls | FXR/RXR Activation | 2.74E-01 | 1.14E-02 |  | IL1RN |
| low-grade PSCC versus paired controls | p70S6K Signaling | 2.55E-01 | 1.06E-02 |  | SFN |
| low-grade PSCC versus paired controls | Role of Macrophages, Fibroblasts and Endothelial Cells in Rheumatoid Arthritis | 2.5E-01 | 9.13E-03 |  | IL1RN,CXCL8 |
| low-grade PSCC versus paired controls | Hereditary Breast Cancer Signaling | 2.49E-01 | 1.04E-02 |  | SFN |
| low-grade PSCC versus paired controls | Insulin Receptor Signaling | 2.4E-01 | 1.01E-02 |  | SGK1 |
| low-grade PSCC versus paired controls | PI3K/AKT Signaling | 2.38E-01 | 1E-02 |  | SFN |
| low-grade PSCC versus paired controls | Estrogen Receptor Signaling | 2.32E-01 | 9.8E-03 |  | HIST1H3C |
| low-grade PSCC versus paired controls | Mitochondrial Dysfunction | 2.15E-01 | 9.17E-03 |  | CYB5A |
| low-grade PSCC versus paired controls | Dendritic Cell Maturation | 2.1E-01 | 9.01E-03 |  | IL1RN |
| low-grade PSCC versus paired controls | Gap Junction Signaling | 2.05E-01 | 8.85E-03 |  | TUBA1B |

Additional file 10. Table S10. IPA results for genes associated with Clusters 1-12.

| **Cluster** | **Ingenuity Canonical Pathways** | **-log(p-value)** | **Ratio** | **Z-score** | **Molecules** |
| --- | --- | --- | --- | --- | --- |
| 1 | Agranulocyte Adhesion and Diapedesis | 4.72E+00 | 4.59E-02 |  | CXCL6,CLDN3,CLDN9,CLDN8,CLDN4 |
| 1 | Granulocyte Adhesion and Diapedesis | 4.68E00 | 4.5E-02 |  | CXCL6,CLDN3,CLDN9,CLDN8,CLDN4 |
| 1 | Tight Junction Signaling | 3.26E00 | 3.23E-02 |  | CLDN3,CLDN9,CLDN8,CLDN4 |
| 1 | Sertoli Cell-Sertoli Cell Junction Signaling | 3.07E00 | 2.86E-02 |  | CLDN3,CLDN9,CLDN8,CLDN4 |
| 1 | Leukocyte Extravasation Signaling | 3.06E00 | 2.84E-02 |  | CLDN3,CLDN9,CLDN8,CLDN4 |
| 1 | Role of IL-17A in Psoriasis | 1.52E00 | 1E-01 |  | CXCL6 |
| 1 | eNOS Signaling | 1.43E00 | 2.02E-02 |  | CHRNA10,PRKAA2 |
| 1 | AMPK Signaling | 1.32E00 | 1.75E-02 |  | CHRNA10,PRKAA2 |
| 1 | IL-17A Signaling in Fibroblasts | 1.13E00 | 4E-02 |  | NFKBIZ |
| 1 | Role of IL-17F in Allergic Inflammatory Airway Diseases | 1.08E00 | 3.57E-02 |  | CXCL6 |
| 1 | nNOS Signaling in Neurons | 1E00 | 2.94E-02 |  | CAPN2 |
| 1 | Calcium-induced T Lymphocyte Apoptosis | 9.57E-01 | 2.63E-02 |  | CAPN2 |
| 1 | Amyloid Processing | 9.57E-01 | 2.63E-02 |  | CAPN2 |
| 1 | Role of IL-17A in Arthritis | 9.47E-01 | 2.56E-02 |  | CXCL6 |
| 1 | Regulation of Cellular Mechanics by Calpain Protease | 9.26E-01 | 2.44E-02 |  | CAPN2 |
| 1 | Pyridoxal 5'-phosphate Salvage Pathway | 8.54E-01 | 2.04E-02 |  | PRKAA2 |
| 1 | IL-17A Signaling in Airway Cells | 8.38E-01 | 1.96E-02 |  | CXCL6 |
| 1 | Hypoxia Signaling in the Cardiovascular System | 8.38E-01 | 1.96E-02 |  | UBE2E3 |
| 1 | VDR/RXR Activation | 8.22E-01 | 1.89E-02 |  | KLF4 |
| 1 | Salvage Pathways of Pyrimidine Ribonucleotides | 7.48E-01 | 1.56E-02 |  | PRKAA2 |
| 1 | Amyotrophic Lateral Sclerosis Signaling | 7.3E-01 | 1.49E-02 |  | CAPN2 |
| 1 | Apoptosis Signaling | 7.3E-01 | 1.49E-02 |  | CAPN2 |
| 1 | FAK Signaling | 7.24E-01 | 1.47E-02 |  | CAPN2 |
| 1 | RhoA Signaling | 6.33E-01 | 1.16E-02 |  | RHPN2 |
| 1 | Type II Diabetes Mellitus Signaling | 6.12E-01 | 1.1E-02 |  | PRKAA2 |
| 1 | Adipogenesis pathway | 5.95E-01 | 1.05E-02 |  | KLF5 |
| 1 | Calcium Signaling | 5.22E-01 | 8.62E-03 |  | CHRNA10 |
| 1 | NRF2-mediated Oxidative Stress Response | 4.81E-01 | 7.69E-03 |  | UBE2E3 |
| 1 | PPARα/RXRα Activation | 4.78E-01 | 7.63E-03 |  | PRKAA2 |
| 1 | Regulation of the Epithelial-Mesenchymal Transition Pathway | 4.52E-01 | 7.09E-03 |  | CLDN3 |
| 1 | mTOR Signaling | 4.43E-01 | 6.9E-03 |  | PRKAA2 |
| 1 | Integrin Signaling | 4.26E-01 | 6.58E-03 |  | CAPN2 |
| 1 | Huntington's Disease Signaling | 3.9E-01 | 5.92E-03 |  | CAPN2 |
| 1 | Protein Ubiquitination Pathway | 3.34E-01 | 4.98E-03 |  | UBE2E3 |
| 1 | Glucocorticoid Receptor Signaling | 3.2E-01 | 4.76E-03 |  | PRKAA2 |
| 2 | Atherosclerosis Signaling | 3.74E00 | 3.75E-02 |  | CCR2,SAA4,CCL11 |
| 2 | Agranulocyte Adhesion and Diapedesis | 3.34E00 | 2.75E-02 |  | CCL19,CCL11,CXCL2 |
| 2 | Granulocyte Adhesion and Diapedesis | 3.32E00 | 2.7E-02 |  | CCL19,CCL11,CXCL2 |
| 2 | Acute Phase Response Signaling | 3.11E00 | 2.29E-02 |  | JUN,SAA2,SAA4 |
| 2 | Production of Nitric Oxide and Reactive Oxygen Species in Macrophages | 3.03E00 | 2.16E-02 |  | JUN,SAA4,RHOU |
| 2 | Remodeling of Epithelial Adherens Junctions | 2.64E00 | 3.92E-02 |  | HGF,MAPRE3 |
| 2 | Chemokine Signaling | 2.64E00 | 3.92E-02 |  | JUN,CCL11 |
| 2 | IL-17 Signaling | 2.57E00 | 3.64E-02 |  | JUN,CCL11 |
| 2 | Renal Cell Carcinoma Signaling | 2.51E00 | 3.39E-02 |  | JUN,HGF |
| 2 | Cholecystokinin/Gastrin-mediated Signaling | 2.28E00 | 2.6E-02 |  | JUN,RHOU |
| 2 | LXR/RXR Activation | 2.21E00 | 2.38E-02 |  | SAA2,SAA4 |
| 2 | HMGB1 Signaling | 2.21E00 | 2.38E-02 |  | JUN,RHOU |
| 2 | HGF Signaling | 2.18E00 | 2.3E-02 |  | JUN,HGF |
| 2 | FXR/RXR Activation | 2.17E00 | 2.27E-02 |  | SAA2,SAA4 |
| 2 | Aryl Hydrocarbon Receptor Signaling | 2.11E00 | 2.11E-02 |  | JUN,CYP1B1 |
| 2 | IL-12 Signaling and Production in Macrophages | 2.07E00 | 2.02E-02 |  | JUN,SAA4 |
| 2 | CXCR4 Signaling | 1.99E00 | 1.83E-02 |  | JUN,RHOU |
| 2 | ILK Signaling | 1.85E00 | 1.54E-02 |  | JUN,RHOU |
| 2 | Extrinsic Prothrombin Activation Pathway | 1.77E00 | 8.33E-02 |  | PROS1 |
| 2 | IL-8 Signaling | 1.75E00 | 1.37E-02 |  | JUN,RHOU |
| 2 | Cardiac Hypertrophy Signaling | 1.65E00 | 1.21E-02 |  | JUN,RHOU |
| 2 | Bupropion Degradation | 1.62E00 | 5.88E-02 |  | CYP1B1 |
| 2 | Acetone Degradation I (to Methylglyoxal) | 1.6E00 | 5.56E-02 |  | CYP1B1 |
| 2 | Signaling by Rho Family GTPases | 1.59E00 | 1.13E-02 |  | JUN,RHOU |
| 2 | Colorectal Cancer Metastasis Signaling | 1.58E00 | 1.11E-02 |  | JUN,RHOU |
| 2 | IL-17A Signaling in Gastric Cells | 1.58E00 | 5.26E-02 |  | JUN |
| 2 | Intrinsic Prothrombin Activation Pathway | 1.51E00 | 4.55E-02 |  | PROS1 |
| 2 | MIF Regulation of Innate Immunity | 1.49E00 | 4.35E-02 |  | JUN |
| 2 | Estrogen Biosynthesis | 1.49E00 | 4.35E-02 |  | CYP1B1 |
| 2 | TNFR2 Signaling | 1.46E00 | 4E-02 |  | JUN |
| 2 | IL-17A Signaling in Fibroblasts | 1.46E00 | 4E-02 |  | JUN |
| 2 | Glucocorticoid Receptor Signaling | 1.46E00 | 9.52E-03 |  | JUN,CCL11 |
| 2 | Inhibition of Angiogenesis by TSP1 | 1.44E00 | 3.85E-02 |  | JUN |
| 2 | 4-1BB Signaling in T Lymphocytes | 1.43E00 | 3.7E-02 |  | JUN |
| 2 | Coagulation System | 1.39E00 | 3.45E-02 |  | PROS1 |
| 2 | MSP-RON Signaling Pathway | 1.38E00 | 3.33E-02 |  | CCR2 |
| 2 | Nicotine Degradation III | 1.38E00 | 3.33E-02 |  | CYP1B1 |
| 2 | April Mediated Signaling | 1.37E00 | 3.23E-02 |  | JUN |
| 2 | OX40 Signaling Pathway | 1.37E00 | 3.23E-02 |  | JUN |
| 2 | UVC-Induced MAPK Signaling | 1.37E00 | 3.23E-02 |  | JUN |
| 2 | B Cell Activating Factor Signaling | 1.35E00 | 3.12E-02 |  | JUN |
| 2 | iNOS Signaling | 1.35E00 | 3.12E-02 |  | JUN |
| 2 | Melatonin Degradation I | 1.33E00 | 2.94E-02 |  | CYP1B1 |
| 2 | Nicotine Degradation II | 1.3E00 | 2.78E-02 |  | CYP1B1 |
| 2 | Superpathway of Melatonin Degradation | 1.3E00 | 2.78E-02 |  | CYP1B1 |
| 2 | UVB-Induced MAPK Signaling | 1.27E00 | 2.56E-02 |  | JUN |
| 2 | Thrombopoietin Signaling | 1.26E00 | 2.5E-02 |  | JUN |
| 2 | TNFR1 Signaling | 1.26E00 | 2.5E-02 |  | JUN |
| 2 | ErbB2-ErbB3 Signaling | 1.26E00 | 2.5E-02 |  | JUN |
| 2 | IL-2 Signaling | 1.26E00 | 2.5E-02 |  | JUN |
| 2 | Estrogen-Dependent Breast Cancer Signaling | 1.25E00 | 2.44E-02 |  | JUN |
| 2 | Semaphorin Signaling in Neurons | 1.23E00 | 2.33E-02 |  | RHOU |
| 2 | IL-10 Signaling | 1.22E00 | 2.27E-02 |  | JUN |
| 2 | CD27 Signaling in Lymphocytes | 1.22E00 | 2.27E-02 |  | JUN |
| 2 | EGF Signaling | 1.22E00 | 2.27E-02 |  | JUN |
| 2 | ATM Signaling | 1.21E00 | 2.22E-02 |  | JUN |
| 2 | CCR5 Signaling in Macrophages | 1.2E00 | 2.17E-02 |  | JUN |
| 2 | Glioma Invasiveness Signaling | 1.2E00 | 2.17E-02 |  | RHOU |
| 2 | Molecular Mechanisms of Cancer | 1.19E00 | 6.73E-03 |  | JUN,RHOU |
| 2 | Actin Nucleation by ARP-WASP Complex | 1.18E00 | 2.08E-02 |  | RHOU |
| 2 | Activation of IRF by Cytosolic Pattern Recognition Receptors | 1.17E00 | 2.04E-02 |  | JUN |
| 2 | Neurotrophin/TRK Signaling | 1.16E00 | 2E-02 |  | JUN |
| 2 | Macropinocytosis Signaling | 1.16E00 | 2E-02 |  | HGF |
| 2 | Erythropoietin Signaling | 1.16E00 | 1.96E-02 |  | JUN |
| 2 | CD40 Signaling | 1.16E00 | 1.96E-02 |  | JUN |
| 2 | IL-17A Signaling in Airway Cells | 1.16E00 | 1.96E-02 |  | CCL11 |
| 2 | GDNF Family Ligand-Receptor Interactions | 1.16E00 | 1.96E-02 |  | JUN |
| 2 | Toll-like Receptor Signaling | 1.16E00 | 1.96E-02 |  | JUN |
| 2 | Hypoxia Signaling in the Cardiovascular System | 1.16E00 | 1.96E-02 |  | JUN |
| 2 | IL-3 Signaling | 1.14E00 | 1.89E-02 |  | JUN |
| 2 | JAK/Stat Signaling | 1.14E00 | 1.89E-02 |  | JUN |
| 2 | PCP pathway | 1.12E00 | 1.82E-02 |  | JUN |
| 2 | LPS-stimulated MAPK Signaling | 1.12E00 | 1.79E-02 |  | JUN |
| 2 | Agrin Interactions at Neuromuscular Junction | 1.11E00 | 1.75E-02 |  | JUN |
| 2 | Prolactin Signaling | 1.1E00 | 1.69E-02 |  | JUN |
| 2 | Ceramide Signaling | 1.09E00 | 1.67E-02 |  | JUN |
| 2 | BMP signaling pathway | 1.09E00 | 1.67E-02 |  | JUN |
| 2 | PDGF Signaling | 1.08E00 | 1.64E-02 |  | JUN |
| 2 | Regulation of Actin-based Motility by Rho | 1.07E00 | 1.61E-02 |  | RHOU |
| 2 | Regulation of IL-2 Expression in Activated and Anergic T Lymphocytes | 1.07E00 | 1.59E-02 |  | JUN |
| 2 | ErbB Signaling | 1.05E00 | 1.52E-02 |  | JUN |
| 2 | FGF Signaling | 1.05E00 | 1.52E-02 |  | HGF |
| 2 | HIF1α Signaling | 1.04E00 | 1.49E-02 |  | JUN |
| 2 | UVA-Induced MAPK Signaling | 1.04E00 | 1.49E-02 |  | JUN |
| 2 | TGF-β Signaling | 1.04E00 | 1.49E-02 |  | JUN |
| 2 | PPAR Signaling | 1.04E00 | 1.47E-02 |  | JUN |
| 2 | IL-1 Signaling | 1.02E00 | 1.41E-02 |  | JUN |
| 2 | T Cell Receptor Signaling | 1.02E00 | 1.41E-02 |  | JUN |
| 2 | RANK Signaling in Osteoclasts | 1.01E00 | 1.39E-02 |  | JUN |
| 2 | SAPK/JNK Signaling | 1.01E00 | 1.37E-02 |  | JUN |
| 2 | p53 Signaling | 9.95E-01 | 1.33E-02 |  | JUN |
| 2 | phagosome formation | 9.95E-01 | 1.33E-02 |  | RHOU |
| 2 | Corticotropin Releasing Hormone Signaling | 9.85E-01 | 1.3E-02 |  | JUN |
| 2 | IGF-1 Signaling | 9.79E-01 | 1.28E-02 |  | JUN |
| 2 | Sphingosine-1-phosphate Signaling | 9.64E-01 | 1.23E-02 |  | RHOU |
| 2 | CCR3 Signaling in Eosinophils | 9.59E-01 | 1.22E-02 |  | CCL11 |
| 2 | Gα12/13 Signaling | 9.59E-01 | 1.22E-02 |  | JUN |
| 2 | CD28 Signaling in T Helper Cells | 9.49E-01 | 1.19E-02 |  | JUN |
| 2 | Renin-Angiotensin Signaling | 9.44E-01 | 1.18E-02 |  | JUN |
| 2 | Cdc42 Signaling | 9.44E-01 | 1.18E-02 |  | JUN |
| 2 | Androgen Signaling | 9.39E-01 | 1.16E-02 |  | JUN |
| 2 | Rac Signaling | 9.39E-01 | 1.16E-02 |  | JUN |
| 2 | PKCθ Signaling in T Lymphocytes | 9.39E-01 | 1.16E-02 |  | JUN |
| 2 | P2Y Purigenic Receptor Signaling Pathway | 9.34E-01 | 1.15E-02 |  | JUN |
| 2 | IL-6 Signaling | 9.34E-01 | 1.15E-02 |  | JUN |
| 2 | Relaxin Signaling | 9.21E-01 | 1.11E-02 |  | JUN |
| 2 | 14-3-3-mediated Signaling | 9.07E-01 | 1.08E-02 |  | JUN |
| 2 | PI3K Signaling in B Lymphocytes | 8.94E-01 | 1.04E-02 |  | JUN |
| 2 | GNRH Signaling | 8.78E-01 | 1E-02 |  | JUN |
| 2 | Hepatic Cholestasis | 8.74E-01 | 9.9E-03 |  | JUN |
| 2 | Epithelial Adherens Junction Signaling | 8.5E-01 | 9.35E-03 |  | HGF |
| 2 | Gαq Signaling | 8.47E-01 | 9.26E-03 |  | RHOU |
| 2 | Tec Kinase Signaling | 8.43E-01 | 9.17E-03 |  | RHOU |
| 2 | Endothelin-1 Signaling | 8.28E-01 | 8.85E-03 |  | JUN |
| 2 | Role of NFAT in Regulation of the Immune Response | 8.18E-01 | 8.62E-03 |  | JUN |
| 2 | Glioblastoma Multiforme Signaling | 8.08E-01 | 8.4E-03 |  | RHOU |
| 2 | Tight Junction Signaling | 7.91E-01 | 8.06E-03 |  | JUN |
| 2 | RhoGDI Signaling | 7.75E-01 | 7.75E-03 |  | RHOU |
| 2 | NRF2-mediated Oxidative Stress Response | 7.72E-01 | 7.69E-03 |  | JUN |
| 2 | PPARα/RXRα Activation | 7.69E-01 | 7.63E-03 |  | JUN |
| 2 | Germ Cell-Sertoli Cell Junction Signaling | 7.63E-01 | 7.52E-03 |  | RHOU |
| 2 | Systemic Lupus Erythematosus Signaling | 7.63E-01 | 7.52E-03 |  | JUN |
| 2 | Thrombin Signaling | 7.57E-01 | 7.41E-03 |  | RHOU |
| 2 | Wnt/β-catenin Signaling | 7.57E-01 | 7.41E-03 |  | JUN |
| 2 | Sertoli Cell-Sertoli Cell Junction Signaling | 7.43E-01 | 7.14E-03 |  | JUN |
| 2 | Clathrin-mediated Endocytosis Signaling | 7.4E-01 | 7.09E-03 |  | SAA4 |
| 2 | Regulation of the Epithelial-Mesenchymal Transition Pathway | 7.4E-01 | 7.09E-03 |  | HGF |
| 2 | B Cell Receptor Signaling | 7.4E-01 | 7.09E-03 |  | JUN |
| 2 | LPS/IL-1 Mediated Inhibition of RXR Function | 7.37E-01 | 7.04E-03 |  | JUN |
| 2 | Hepatic Fibrosis / Hepatic Stellate Cell Activation | 7.35E-01 | 6.99E-03 |  | HGF |
| 2 | mTOR Signaling | 7.29E-01 | 6.9E-03 |  | RHOU |
| 2 | RAR Activation | 7.18E-01 | 6.71E-03 |  | JUN |
| 2 | Integrin Signaling | 7.11E-01 | 6.58E-03 |  | RHOU |
| 2 | Phospholipase C Signaling | 6.93E-01 | 6.29E-03 |  | RHOU |
| 2 | Role of Osteoblasts, Osteoclasts and Chondrocytes in Rheumatoid Arthritis | 6.91E-01 | 6.25E-03 |  | JUN |
| 2 | Huntington's Disease Signaling | 6.69E-01 | 5.92E-03 |  | JUN |
| 2 | Xenobiotic Metabolism Signaling | 6.22E-01 | 5.24E-03 |  | CYP1B1 |
| 2 | Role of Macrophages, Fibroblasts and Endothelial Cells in Rheumatoid Arthritis | 5.71E-01 | 4.57E-03 |  | JUN |
| 3 | Pyrimidine Deoxyribonucleotides De Novo Biosynthesis I | 3.28E00 | 1.9E-01 |  | AK9,AK8,NME5,AK1 |
| 3 | Pyrimidine Ribonucleotides Interconversion | 2.91E00 | 1.54E-01 |  | AK9,AK8,NME5,AK1 |
| 3 | Pyrimidine Ribonucleotides De Novo Biosynthesis | 2.79E00 | 1.43E-01 |  | AK9,AK8,NME5,AK1 |
| 3 | LPS/IL-1 Mediated Inhibition of RXR Function | 1.82E00 | 4.33E-02 | 1.000 | FABP6,FMO5,CYP2B6,PPARGC1A,SLC27A2,GSTA2,IL1R2,SULT1E1,GSTA1 |
| 3 | Nicotine Degradation II | 1.79E00 | 7.55E-02 |  | CYP4B1,FMO5,CYP2B6,UGT2A1 |
| 3 | PXR/RXR Activation | 1.54E00 | 6.35E-02 |  | CYP2B6,PPARGC1A,GSTA2,GSTA1 |
| 3 | L-DOPA Degradation | 1.44E00 | 5E-01 |  | LRTOMT |
| 3 | Methionine Salvage II (Mammalian) | 1.27E00 | 3.33E-01 |  | BHMT2 |
| 3 | Nicotine Degradation III | 1.26E00 | 6.38E-02 |  | CYP4B1,CYP2B6,UGT2A1 |
| 3 | Melatonin Degradation I | 1.19E00 | 6E-02 |  | CYP4B1,CYP2B6,UGT2A1 |
| 3 | Bupropion Degradation | 1.15E00 | 8.33E-02 |  | CYP4B1,CYP2B6 |
| 3 | Glutathione-mediated Detoxification | 1.12E00 | 8E-02 |  | GSTA2,GSTA1 |
| 3 | Acetone Degradation I (to Methylglyoxal) | 1.12E00 | 8E-02 |  | CYP4B1,CYP2B6 |
| 3 | Superpathway of Melatonin Degradation | 1.09E00 | 5.45E-02 |  | CYP4B1,CYP2B6,UGT2A1 |
| 3 | Salvage Pathways of Pyrimidine Ribonucleotides | 1.06E00 | 4.4E-02 |  | AK9,AK8,NME5,AK1 |
| 3 | Noradrenaline and Adrenaline Degradation | 9.32E-01 | 6.25E-02 |  | ADH1B,LRTOMT |
| 3 | Estrogen Biosynthesis | 8.49E-01 | 5.56E-02 |  | CYP4B1,CYP2B6 |
| 3 | Prostanoid Biosynthesis | 8.13E-01 | 1.11E-01 |  | PTGDS |
| 3 | Glycine Betaine Degradation | 7.71E-01 | 1E-01 |  | BHMT2 |
| 3 | Atherosclerosis Signaling | 7.35E-01 | 3.31E-02 |  | CLU,APOD,F3,ALOX15 |
| 3 | Xenobiotic Metabolism Signaling | 7.12E-01 | 2.73E-02 |  | FMO5,CYP2B6,PPARGC1A,GSTA2,UGT2A1,SULT1E1,GSTA1 |
| 3 | FXR/RXR Activation | 7.02E-01 | 3.2E-02 |  | FABP6,PPARGC1A,CLU,APOD |
| 3 | Fatty Acid Activation | 6.68E-01 | 7.69E-02 |  | SLC27A2 |
| 3 | IL-12 Signaling and Production in Macrophages | 6.54E-01 | 3.05E-02 |  | FOS,CLU,APOD,ALOX15 |
| 3 | nNOS Signaling in Skeletal Muscle Cells | 6.4E-01 | 7.14E-02 |  | DMD |
| 3 | Phenylalanine Degradation IV (Mammalian, via Side Chain) | 6.4E-01 | 7.14E-02 |  | SLC27A2 |
| 3 | PPAR Signaling | 6.38E-01 | 3.33E-02 |  | FOS,PPARGC1A,IL1R2 |
| 3 | Aryl Hydrocarbon Receptor Signaling | 6.24E-01 | 2.96E-02 |  | FOS,GSTA2,NFIA,GSTA1 |
| 3 | Extrinsic Prothrombin Activation Pathway | 5.9E-01 | 6.25E-02 |  | F3 |
| 3 | Serotonin Degradation | 5.71E-01 | 3.64E-02 |  | ADH1B,UGT2A1 |
| 3 | γ-linolenate Biosynthesis II (Animals) | 5.67E-01 | 5.88E-02 |  | SLC27A2 |
| 3 | Mitochondrial L-carnitine Shuttle Pathway | 5.67E-01 | 5.88E-02 |  | SLC27A2 |
| 3 | Glutamate Receptor Signaling | 5.6E-01 | 3.57E-02 |  | SLC1A1,GRM7 |
| 3 | Cardiomyocyte Differentiation via BMP Receptors | 5.27E-01 | 5.26E-02 |  | BMPR1B |
| 3 | GADD45 Signaling | 5.27E-01 | 5.26E-02 |  | GADD45B |
| 3 | Eicosanoid Signaling | 5E-01 | 3.23E-02 |  | PTGDS,ALOX15 |
| 3 | Dopamine Degradation | 4.91E-01 | 4.76E-02 |  | LRTOMT |
| 3 | IL-10 Signaling | 4.48E-01 | 2.94E-02 |  | FOS,IL1R2 |
| 3 | Granulocyte Adhesion and Diapedesis | 4.43E-01 | 2.42E-02 |  | CXCL17,C5AR1,CCL15,IL1R2 |
| 3 | IL-17A Signaling in Gastric Cells | 4.3E-01 | 4E-02 |  | FOS |
| 3 | Antiproliferative Role of TOB in T Cell Signaling | 4.17E-01 | 3.85E-02 |  | TOB1 |
| 3 | LXR/RXR Activation | 4.14E-01 | 2.48E-02 |  | CLU,APOD,IL1R2 |
| 3 | TNFR2 Signaling | 3.92E-01 | 3.57E-02 |  | FOS |
| 3 | NRF2-mediated Oxidative Stress Response | 3.86E-01 | 2.26E-02 |  | FOS,DNAJA4,GSTA2,GSTA1 |
| 3 | Thyroid Hormone Metabolism II (via Conjugation and/or Degradation) | 3.8E-01 | 3.45E-02 |  | UGT2A1 |
| 3 | Fatty Acid β-oxidation I | 3.8E-01 | 3.45E-02 |  | SLC27A2 |
| 3 | Regulation of IL-2 Expression in Activated and Anergic T Lymphocytes | 3.75E-01 | 2.56E-02 |  | FOS,TOB1 |
| 3 | Ethanol Degradation II | 3.69E-01 | 3.33E-02 |  | ADH1B |
| 3 | Superpathway of Methionine Degradation | 3.59E-01 | 3.23E-02 |  | BHMT2 |
| 3 | TR/RXR Activation | 3.32E-01 | 2.35E-02 |  | UCP2,PPARGC1A |
| 3 | Oncostatin M Signaling | 3.29E-01 | 2.94E-02 |  | EPAS1 |
| 3 | Stearate Biosynthesis I (Animals) | 3.29E-01 | 2.94E-02 |  | SLC27A2 |
| 3 | TGF-β Signaling | 3.21E-01 | 2.3E-02 |  | FOS,BMPR1B |
| 3 | Coagulation System | 3.2E-01 | 2.86E-02 |  | F3 |
| 3 | Cell Cycle Regulation by BTG Family Proteins | 3.2E-01 | 2.86E-02 |  | BTG2 |
| 3 | IL-17A Signaling in Fibroblasts | 3.2E-01 | 2.86E-02 |  | FOS |
| 3 | tRNA Splicing | 3.2E-01 | 2.86E-02 |  | PDE8B |
| 3 | Complement System | 3.12E-01 | 2.78E-02 |  | C5AR1 |
| 3 | April Mediated Signaling | 2.95E-01 | 2.63E-02 |  | FOS |
| 3 | Docosahexaenoic Acid (DHA) Signaling | 2.88E-01 | 2.56E-02 |  | ALOX15 |
| 3 | B Cell Activating Factor Signaling | 2.8E-01 | 2.5E-02 |  | FOS |
| 3 | MIF Regulation of Innate Immunity | 2.73E-01 | 2.44E-02 |  | FOS |
| 3 | CDK5 Signaling | 2.67E-01 | 2.04E-02 |  | FOSB,EGR1 |
| 3 | Dermatan Sulfate Biosynthesis (Late Stages) | 2.66E-01 | 2.38E-02 |  | SULT1E1 |
| 3 | UVC-Induced MAPK Signaling | 2.66E-01 | 2.38E-02 |  | FOS |
| 3 | Neuropathic Pain Signaling In Dorsal Horn Neurons | 2.62E-01 | 2.02E-02 |  | FOS,GRM7 |
| 3 | iNOS Signaling | 2.59E-01 | 2.33E-02 |  | FOS |
| 3 | Chondroitin Sulfate Biosynthesis (Late Stages) | 2.53E-01 | 2.27E-02 |  | SULT1E1 |
| 3 | cAMP-mediated signaling | 2.48E-01 | 1.85E-02 |  | DUSP1,AKAP14,PDE8B,GRM7 |
| 3 | Role of Oct4 in Mammalian Embryonic Stem Cell Pluripotency | 2.47E-01 | 2.22E-02 |  | FBXO15 |
| 3 | TNFR1 Signaling | 2.35E-01 | 2.13E-02 |  | FOS |
| 3 | Corticotropin Releasing Hormone Signaling | 2.3E-01 | 1.87E-02 |  | FOS,NR4A1 |
| 3 | Role of Tissue Factor in Cancer | 2.3E-01 | 1.87E-02 |  | F3,EGR1 |
| 3 | Heparan Sulfate Biosynthesis (Late Stages) | 2.29E-01 | 2.08E-02 |  | SULT1E1 |
| 3 | CD27 Signaling in Lymphocytes | 2.13E-01 | 1.96E-02 |  | FOS |
| 3 | Nur77 Signaling in T Lymphocytes | 2.13E-01 | 1.96E-02 |  | NR4A1 |
| 3 | Chondroitin Sulfate Biosynthesis | 2.08E-01 | 1.92E-02 |  | SULT1E1 |
| 3 | UVB-Induced MAPK Signaling | 2.03E-01 | 1.89E-02 |  | FOS |
| 3 | Unfolded protein response | 2.03E-01 | 1.89E-02 |  | PPP1R15A |
| 3 | IL-2 Signaling | 2.03E-01 | 1.89E-02 |  | FOS |
| 4 | Choline Biosynthesis III | 2.61E00 | 1.54E-01 |  | PCYT1A,PLD1 |
| 4 | p53 Signaling | 2.61E00 | 4.08E-02 | 1.000 | TP63,PERP,TOPBP1,BAX |
| 4 | PCP pathway | 2.27E00 | 4.84E-02 |  | WNT5A,DVL3,SDC1 |
| 4 | Gluconeogenesis I | 2.05E00 | 8E-02 |  | ME1,GPI |
| 4 | Epithelial Adherens Junction Signaling | 2.03E00 | 2.8E-02 |  | ACTR2,RAP1B,TUBA1B,WASF1 |
| 4 | Role of BRCA1 in DNA Damage Response | 1.99E00 | 3.85E-02 |  | BRIP1,TOPBP1,PLK1 |
| 4 | Cell Cycle Control of Chromosomal Replication | 1.98E00 | 7.41E-02 |  | CDC45,MCM4 |
| 4 | Salvage Pathways of Pyrimidine Ribonucleotides | 1.81E00 | 3.3E-02 |  | MAPK6,NME2,PLK1 |
| 4 | L-carnitine Biosynthesis | 1.77E00 | 3.33E-01 |  | BBOX1 |
| 4 | CDK5 Signaling | 1.73E00 | 3.06E-02 |  | ITGA6,NTRK2,MAPK6 |
| 4 | Rac Signaling | 1.67E00 | 2.91E-02 |  | ACTR2,PLD1,WASF1 |
| 4 | Pentose Phosphate Pathway (Oxidative Branch) | 1.64E00 | 2.5E-01 |  | PGD |
| 4 | NAD Biosynthesis III | 1.64E00 | 2.5E-01 |  | NAMPT |
| 4 | RhoA Signaling | 1.5E00 | 2.5E-02 |  | ACTR2,PLD1,WASF1 |
| 4 | GDP-mannose Biosynthesis | 1.47E00 | 1.67E-01 |  | GPI |
| 4 | Phosphatidylcholine Biosynthesis I | 1.4E00 | 1.43E-01 |  | PCYT1A |
| 4 | Wnt/Ca+ pathway | 1.4E00 | 3.64E-02 |  | WNT5A,DVL3 |
| 4 | Actin Nucleation by ARP-WASP Complex | 1.39E00 | 3.57E-02 |  | ACTR2,WASF1 |
| 4 | UDP-N-acetyl-D-galactosamine Biosynthesis II | 1.3E00 | 1.11E-01 |  | GPI |
| 4 | Pyridoxal 5'-phosphate Salvage Pathway | 1.29E00 | 3.17E-02 |  | MAPK6,PLK1 |
| 4 | Remodeling of Epithelial Adherens Junctions | 1.26E00 | 3.03E-02 |  | ACTR2,TUBA1B |
| 4 | Pentose Phosphate Pathway | 1.25E00 | 1E-01 |  | PGD |
| 4 | Dolichyl-diphosphooligosaccharide Biosynthesis | 1.25E00 | 1E-01 |  | ALG3 |
| 4 | Basal Cell Carcinoma Signaling | 1.22E00 | 2.9E-02 |  | WNT5A,DVL3 |
| 4 | Role of Wnt/GSK-3β Signaling in the Pathogenesis of Influenza | 1.18E00 | 2.74E-02 |  | WNT5A,DVL3 |
| 4 | Cholesterol Biosynthesis I | 1.14E00 | 7.69E-02 |  | DHCR7 |
| 4 | Cholesterol Biosynthesis II (via 24,25-dihydrolanosterol) | 1.14E00 | 7.69E-02 |  | DHCR7 |
| 4 | Cholesterol Biosynthesis III (via Desmosterol) | 1.14E00 | 7.69E-02 |  | DHCR7 |
| 4 | EIF2 Signaling | 1.12E00 | 1.74E-02 |  | RPL37A,EIF4A2,EIF4G1 |
| 4 | Colanic Acid Building Blocks Biosynthesis | 1.11E00 | 7.14E-02 |  | GPI |
| 4 | mTOR Signaling | 1.06E00 | 1.65E-02 |  | EIF4A2,PLD1,EIF4G1 |
| 4 | Regulation of the Epithelial-Mesenchymal Transition Pathway | 1.06E00 | 1.65E-02 |  | WNT5A,HMGA2,DVL3 |
| 4 | Regulation of Actin-based Motility by Rho | 1.05E00 | 2.3E-02 |  | ACTR2,WASF1 |
| 4 | Axonal Guidance Signaling | 1.01E00 | 1.17E-02 |  | ACTR2,WNT5A,RAP1B,TUBA1B,NTRK2 |
| 4 | Fcγ Receptor-mediated Phagocytosis in Macrophages and Monocytes | 1E00 | 2.15E-02 |  | ACTR2,PLD1 |
| 4 | Integrin Signaling | 9.97E-01 | 1.55E-02 |  | ACTR2,ITGA6,RAP1B |
| 4 | Pyrimidine Deoxyribonucleotides De Novo Biosynthesis I | 9.44E-01 | 4.76E-02 |  | NME2 |
| 4 | NGF Signaling | 9.07E-01 | 1.89E-02 |  | RAP1B,BAX |
| 4 | Tumoricidal Function of Hepatic Natural Killer Cells | 8.89E-01 | 4.17E-02 |  | BAX |
| 4 | Glycolysis I | 8.89E-01 | 4.17E-02 |  | GPI |
| 4 | Pyrimidine Ribonucleotides Interconversion | 8.57E-01 | 3.85E-02 |  | NME2 |
| 4 | 14-3-3-mediated Signaling | 8.44E-01 | 1.72E-02 |  | TUBA1B,BAX |
| 4 | Superpathway of Cholesterol Biosynthesis | 8.42E-01 | 3.7E-02 |  | DHCR7 |
| 4 | p70S6K Signaling | 8.32E-01 | 1.69E-02 |  | PLD1,BCAP31 |
| 4 | Colorectal Cancer Metastasis Signaling | 8.32E-01 | 1.3E-02 |  | WNT5A,TLR6,BAX |
| 4 | Pyrimidine Ribonucleotides De Novo Biosynthesis | 8.27E-01 | 3.57E-02 |  | NME2 |
| 4 | Signaling by Rho Family GTPases | 8.24E-01 | 1.29E-02 |  | ACTR2,PLD1,WASF1 |
| 4 | Adipogenesis pathway | 7.87E-01 | 1.59E-02 |  | EZH2,WNT5A |
| 4 | Inhibition of Angiogenesis by TSP1 | 7.74E-01 | 3.12E-02 |  | SDC1 |
| 4 | Human Embryonic Stem Cell Pluripotency | 7.61E-01 | 1.53E-02 |  | WNT5A,NTRK2 |
| 4 | Oncostatin M Signaling | 7.5E-01 | 2.94E-02 |  | PLAU |
| 4 | Interferon Signaling | 7.5E-01 | 2.94E-02 |  | BAX |
| 4 | Protein Ubiquitination Pathway | 7.47E-01 | 1.18E-02 |  | PSMD3,UBE2C,DNAJB6 |
| 4 | Coagulation System | 7.39E-01 | 2.86E-02 |  | PLAU |
| 4 | Regulation of eIF4 and p70S6K Signaling | 7.08E-01 | 1.41E-02 |  | EIF4A2,EIF4G1 |
| 4 | Inhibition of Matrix Metalloproteases | 7.07E-01 | 2.63E-02 |  | SDC1 |
| 4 | Docosahexaenoic Acid (DHA) Signaling | 6.96E-01 | 2.56E-02 |  | BAX |
| 4 | Thyroid Cancer Signaling | 6.96E-01 | 2.56E-02 |  | NTRK2 |
| 4 | Neuroprotective Role of THOP1 in Alzheimer's Disease | 6.87E-01 | 2.5E-02 |  | NTS |
| 4 | Germ Cell-Sertoli Cell Junction Signaling | 6.47E-01 | 1.28E-02 |  | ITGA6,TUBA1B |
| 4 | Granulocyte Adhesion and Diapedesis | 6.12E-01 | 1.21E-02 |  | ITGA6,SDC1 |
| 4 | Cell Cycle: G2/M DNA Damage Checkpoint Regulation | 6.09E-01 | 2.04E-02 |  | PLK1 |
| 4 | Wnt/β-catenin Signaling | 6.08E-01 | 1.2E-02 |  | WNT5A,DVL3 |
| 4 | Endothelin-1 Signaling | 6.01E-01 | 1.19E-02 |  | PLD1,MAPK6 |
| 4 | NF-κB Signaling | 5.97E-01 | 1.18E-02 |  | NTRK2,TLR6 |
| 4 | Calcium Signaling | 5.93E-01 | 1.18E-02 |  | TP63,RAP1B |
| 4 | RhoGDI Signaling | 5.86E-01 | 1.16E-02 |  | ACTR2,WASF1 |
| 4 | Ephrin Receptor Signaling | 5.86E-01 | 1.16E-02 |  | ACTR2,RAP1B |
| 4 | Sertoli Cell-Sertoli Cell Junction Signaling | 5.83E-01 | 1.16E-02 |  | DLG1,TUBA1B |
| 4 | NRF2-mediated Oxidative Stress Response | 5.69E-01 | 1.13E-02 |  | FTL,DNAJB6 |
| 4 | Role of CHK Proteins in Cell Cycle Checkpoint Control | 5.66E-01 | 1.82E-02 |  | PLK1 |
| 4 | Phospholipases | 5.66E-01 | 1.82E-02 |  | PLD1 |
| 4 | Glioma Invasiveness Signaling | 5.53E-01 | 1.75E-02 |  | PLAU |
| 4 | IL-8 Signaling | 5.49E-01 | 1.09E-02 |  | BAX,PLD1 |
| 4 | Myc Mediated Apoptosis Signaling | 5.47E-01 | 1.72E-02 |  | BAX |
| 4 | Induction of Apoptosis by HIV1 | 5.4E-01 | 1.69E-02 |  | BAX |
| 4 | Retinoic acid Mediated Apoptosis Signaling | 5.4E-01 | 1.69E-02 |  | DAP3 |
| 4 | Antiproliferative Role of Somatostatin Receptor 2 | 5.34E-01 | 1.67E-02 |  | RAP1B |
| 4 | Leukocyte Extravasation Signaling | 5.17E-01 | 1.04E-02 |  | ITGA6,RAP1B |
| 4 | Mitotic Roles of Polo-Like Kinase | 5.17E-01 | 1.59E-02 |  | PLK1 |
| 4 | Hypoxia Signaling in the Cardiovascular System | 5.17E-01 | 1.59E-02 |  | UBE2C |
| 4 | Agrin Interactions at Neuromuscular Junction | 4.94E-01 | 1.49E-02 |  | ITGA6 |
| 4 | Role of MAPK Signaling in the Pathogenesis of Influenza | 4.94E-01 | 1.49E-02 |  | BAX |
| 4 | Neurotrophin/TRK Signaling | 4.94E-01 | 1.49E-02 |  | NTRK2 |
| 4 | TREM1 Signaling | 4.84E-01 | 1.45E-02 |  | TLR6 |
| 4 | Caveolar-mediated Endocytosis Signaling | 4.74E-01 | 1.41E-02 |  | ITGA6 |
| 4 | Molecular Mechanisms of Cancer | 4.69E-01 | 8.36E-03 |  | WNT5A,RAP1B,BAX |
| 4 | Actin Cytoskeleton Signaling | 4.69E-01 | 9.52E-03 |  | ACTR2,WASF1 |
| 4 | Toll-like Receptor Signaling | 4.69E-01 | 1.39E-02 |  | TLR6 |
| 4 | NF-κB Activation by Viruses | 4.64E-01 | 1.37E-02 |  | ITGA6 |
| 4 | STAT3 Pathway | 4.64E-01 | 1.37E-02 |  | NTRK2 |
| 4 | Role of Osteoblasts, Osteoclasts and Chondrocytes in Rheumatoid Arthritis | 4.58E-01 | 9.35E-03 |  | DLX5,WNT5A |
| 4 | Reelin Signaling in Neurons | 4.37E-01 | 1.27E-02 |  | ITGA6 |
| 4 | Altered T Cell and B Cell Signaling in Rheumatoid Arthritis | 4.28E-01 | 1.23E-02 |  | TLR6 |
| 4 | Communication between Innate and Adaptive Immune Cells | 4.24E-01 | 1.22E-02 |  | TLR6 |
| 4 | Phospholipase C Signaling | 4.17E-01 | 8.66E-03 |  | RAP1B,PLD1 |
| 4 | TR/RXR Activation | 4.12E-01 | 1.18E-02 |  | ME1 |
| 4 | HIPPO signaling | 4.12E-01 | 1.18E-02 |  | DLG1 |
| 4 | Apoptosis Signaling | 4E-01 | 1.14E-02 |  | BAX |
| 4 | Virus Entry via Endocytic Pathways | 3.96E-01 | 1.12E-02 |  | ITGA6 |
| 4 | Crosstalk between Dendritic Cells and Natural Killer Cells | 3.96E-01 | 1.12E-02 |  | CD69 |
| 4 | Mouse Embryonic Stem Cell Pluripotency | 3.78E-01 | 1.06E-02 |  | DVL3 |
| 4 | Antioxidant Action of Vitamin C | 3.75E-01 | 1.05E-02 |  | PLD1 |
| 4 | Amyotrophic Lateral Sclerosis Signaling | 3.68E-01 | 1.03E-02 |  | BAX |
| 4 | Paxillin Signaling | 3.65E-01 | 1.02E-02 |  | ITGA6 |
| 4 | Neuropathic Pain Signaling In Dorsal Horn Neurons | 3.61E-01 | 1.01E-02 |  | NTRK2 |
| 4 | HIF1α Signaling | 3.58E-01 | 1E-02 |  | MAPK6 |
| 4 | phagosome formation | 3.52E-01 | 9.8E-03 |  | TLR6 |
| 4 | HGF Signaling | 3.46E-01 | 9.62E-03 |  | RAP1B |
| 4 | Pancreatic Adenocarcinoma Signaling | 3.4E-01 | 9.43E-03 |  | PLD1 |
| 4 | fMLP Signaling in Neutrophils | 3.37E-01 | 9.35E-03 |  | ACTR2 |
| 4 | Corticotropin Releasing Hormone Signaling | 3.37E-01 | 9.35E-03 |  | RAP1B |
| 4 | Role of Tissue Factor in Cancer | 3.37E-01 | 9.35E-03 |  | ITGA6 |
| 4 | Role of NANOG in Mammalian Embryonic Stem Cell Pluripotency | 3.31E-01 | 9.17E-03 |  | WNT5A |
| 4 | CD28 Signaling in T Helper Cells | 3.2E-01 | 8.85E-03 |  | ACTR2 |
| 4 | Synaptic Long Term Potentiation | 3.12E-01 | 8.62E-03 |  | RAP1B |
| 4 | Role of Macrophages, Fibroblasts and Endothelial Cells in Rheumatoid Arthritis | 3.08E-01 | 6.97E-03 |  | WNT5A,TLR6 |
| 4 | PTEN Signaling | 3.06E-01 | 8.47E-03 |  | NTRK2 |
| 4 | phagosome maturation | 3.06E-01 | 8.47E-03 |  | TUBA1B |
| 4 | Role of Pattern Recognition Receptors in Recognition of Bacteria and Viruses | 3.04E-01 | 8.4E-03 |  | TLR6 |
| 4 | LXR/RXR Activation | 2.99E-01 | 8.26E-03 |  | ABCA1 |
| 4 | FXR/RXR Activation | 2.89E-01 | 8E-03 |  | SDC1 |
| 4 | Cdc42 Signaling | 2.8E-01 | 7.75E-03 |  | ACTR2 |
| 4 | Ovarian Cancer Signaling | 2.77E-01 | 7.69E-03 |  | WNT5A |
| 4 | Relaxin Signaling | 2.73E-01 | 7.58E-03 |  | RAP1B |
| 4 | Aryl Hydrocarbon Receptor Signaling | 2.67E-01 | 7.41E-03 |  | BAX |
| 4 | Glioblastoma Multiforme Signaling | 2.46E-01 | 6.9E-03 |  | WNT5A |
| 4 | Gαq Signaling | 2.46E-01 | 6.9E-03 |  | PLD1 |
| 4 | Aldosterone Signaling in Epithelial Cells | 2.35E-01 | 6.62E-03 |  | DNAJB6 |
| 4 | Gap Junction Signaling | 2.35E-01 | 6.62E-03 |  | TUBA1B |
| 4 | PPARα/RXRα Activation | 2.11E-01 | 6.06E-03 |  | ABCA1 |
| 4 | Acute Phase Response Signaling | 2.07E-01 | 5.95E-03 |  | FTL |
| 4 | B Cell Receptor Signaling | 2.02E-01 | 5.85E-03 |  | RAP1B |
| 5 | Ethanol Degradation II | 2.46E00 | 1E-01 |  | DHRS9,ADH1A,ADH6 |
| 5 | Noradrenaline and Adrenaline Degradation | 2.38E00 | 9.38E-02 |  | DHRS9,ADH1A,ADH6 |
| 5 | The Visual Cycle | 2E00 | 1.33E-01 |  | DHRS9,RBP2 |
| 5 | L-dopachrome Biosynthesis | 1.99E00 | 1E00 |  | TYR |
| 5 | Serotonin Degradation | 1.73E00 | 5.45E-02 |  | DHRS9,ADH1A,ADH6 |
| 5 | Eicosanoid Signaling | 1.59E00 | 4.84E-02 |  | PLA2G10,RARRES3,HPGDS |
| 5 | IL-17A Signaling in Gastric Cells | 1.57E00 | 8E-02 |  | MAPK10,CXCL1 |
| 5 | Glutathione-mediated Detoxification | 1.57E00 | 8E-02 |  | GSTA3,HPGDS |
| 5 | Endothelin-1 Signaling | 1.53E00 | 2.98E-02 | -2.236 | MAPK15,PLA2G10,MAPK10,RARRES3,GNA14 |
| 5 | PRPP Biosynthesis I | 1.52E00 | 3.33E-01 |  | PRPS1L1 |
| 5 | S-adenosyl-L-methionine Biosynthesis | 1.52E00 | 3.33E-01 |  | MAT1A |
| 5 | Role of MAPK Signaling in the Pathogenesis of Influenza | 1.5E00 | 4.48E-02 |  | PLA2G10,MAPK10,RARRES3 |
| 5 | NRF2-mediated Oxidative Stress Response | 1.45E00 | 2.82E-02 |  | CYP4Z1,GSTA3,DNAJB13,MAFK,EPHX1 |
| 5 | Retinoate Biosynthesis I | 1.43E00 | 6.67E-02 |  | DHRS9,RBP2 |
| 5 | Retinol Biosynthesis | 1.43E00 | 6.67E-02 |  | DHRS9,RBP2 |
| 5 | Retinoate Biosynthesis II | 1.39E00 | 2.5E-01 |  | RBP2 |
| 5 | Creatine-phosphate Biosynthesis | 1.39E00 | 2.5E-01 |  | CKB |
| 5 | Eumelanin Biosynthesis | 1.39E00 | 2.5E-01 |  | TYR |
| 5 | Aryl Hydrocarbon Receptor Signaling | 1.3E00 | 2.96E-02 |  | GSTA3,TFF1,ALDH3B1,TYR |
| 5 | April Mediated Signaling | 1.24E00 | 5.26E-02 |  | MAPK10,NFAT5 |
| 5 | Thyroid Cancer Signaling | 1.22E00 | 5.13E-02 |  | CXCL1,NTF4 |
| 5 | B Cell Activating Factor Signaling | 1.2E00 | 5E-02 |  | MAPK10,NFAT5 |
| 5 | MIF Regulation of Innate Immunity | 1.18E00 | 4.88E-02 |  | PLA2G10,MAPK10 |
| 5 | UVC-Induced MAPK Signaling | 1.16E00 | 4.76E-02 |  | SMPD3,MAPK10 |
| 5 | Antioxidant Action of Vitamin C | 1.13E00 | 3.16E-02 |  | PLA2G10,MAPK10,RARRES3 |
| 5 | Sphingomyelin Metabolism | 1.1E00 | 1.25E-01 |  | SMPD3 |
| 5 | Prostanoid Biosynthesis | 1.05E00 | 1.11E-01 |  | HPGDS |
| 5 | Calcium Transport I | 1.05E00 | 1.11E-01 |  | ATP2C2 |
| 5 | Role of IL-17A in Arthritis | 9.77E-01 | 3.7E-02 |  | MAPK10,CXCL1 |
| 5 | Phospholipases | 9.63E-01 | 3.64E-02 |  | PLA2G10,RARRES3 |
| 5 | Hematopoiesis from Multipotent Stem Cells | 9.35E-01 | 8.33E-02 |  | IL7 |
| 5 | Androgen Biosynthesis | 9.35E-01 | 8.33E-02 |  | SRD5A2 |
| 5 | Role of IL-17A in Psoriasis | 9.03E-01 | 7.69E-02 |  | CXCL1 |
| 5 | Atherosclerosis Signaling | 8.95E-01 | 2.48E-02 |  | PLA2G10,RARRES3,SELE |
| 5 | IL-17A Signaling in Airway Cells | 8.56E-01 | 3.12E-02 |  | MAPK10,CXCL1 |
| 5 | Fatty Acid α-oxidation | 8.45E-01 | 6.67E-02 |  | BCO2 |
| 5 | Agrin Interactions at Neuromuscular Junction | 8.24E-01 | 2.99E-02 |  | MAPK10,NRG4 |
| 5 | CDP-diacylglycerol Biosynthesis I | 8.19E-01 | 6.25E-02 |  | CDS1 |
| 5 | Methionine Degradation I (to Homocysteine) | 8.19E-01 | 6.25E-02 |  | MAT1A |
| 5 | Synaptic Long Term Depression | 7.88E-01 | 2.21E-02 |  | PLA2G10,RARRES3,GNA14 |
| 5 | IL-17 Signaling | 7.75E-01 | 2.78E-02 |  | MAPK10,CXCL1 |
| 5 | Differential Regulation of Cytokine Production in Macrophages and T Helper Cells by IL-17A and IL-17F | 7.72E-01 | 5.56E-02 |  | CXCL1 |
| 5 | Phosphatidylglycerol Biosynthesis II (Non-plastidic) | 7.72E-01 | 5.56E-02 |  | CDS1 |
| 5 | Cysteine Biosynthesis III (mammalia) | 7.72E-01 | 5.56E-02 |  | MAT1A |
| 5 | BMP signaling pathway | 7.57E-01 | 2.7E-02 |  | MAPK10,BMP15 |
| 5 | Role of Osteoblasts, Osteoclasts and Chondrocytes in Rheumatoid Arthritis | 7.53E-01 | 1.87E-02 |  | MAPK10,BMP15,NFAT5,IL7 |
| 5 | Regulation of IL-2 Expression in Activated and Anergic T Lymphocytes | 7.22E-01 | 2.56E-02 |  | MAPK10,NFAT5 |
| 5 | Pyrimidine Deoxyribonucleotides De Novo Biosynthesis I | 7.12E-01 | 4.76E-02 |  | AK7 |
| 5 | Differential Regulation of Cytokine Production in Intestinal Epithelial Cells by IL-17A and IL-17F | 6.76E-01 | 4.35E-02 |  | CXCL1 |
| 5 | ErbB Signaling | 6.66E-01 | 2.35E-02 |  | MAPK10,NRG4 |
| 5 | IL-22 Signaling | 6.6E-01 | 4.17E-02 |  | MAPK10 |
| 5 | UVA-Induced MAPK Signaling | 6.51E-01 | 2.3E-02 |  | SMPD3,MAPK10 |
| 5 | Role of JAK family kinases in IL-6-type Cytokine Signaling | 6.44E-01 | 4E-02 |  | MAPK10 |
| 5 | Pyrimidine Ribonucleotides Interconversion | 6.3E-01 | 3.85E-02 |  | AK7 |
| 5 | IL-1 Signaling | 6.22E-01 | 2.2E-02 |  | MAPK10,GNA14 |
| 5 | B Cell Development | 6.02E-01 | 3.57E-02 |  | IL7 |
| 5 | Pyrimidine Ribonucleotides De Novo Biosynthesis | 6.02E-01 | 3.57E-02 |  | AK7 |
| 5 | CDK5 Signaling | 5.77E-01 | 2.04E-02 |  | MAPK15,MAPK10 |
| 5 | HIF1α Signaling | 5.64E-01 | 2E-02 |  | MAPK15,MAPK10 |
| 5 | 4-1BB Signaling in T Lymphocytes | 5.64E-01 | 3.23E-02 |  | MAPK10 |
| 5 | Superpathway of Methionine Degradation | 5.64E-01 | 3.23E-02 |  | MAT1A |
| 5 | Inhibition of Angiogenesis by TSP1 | 5.52E-01 | 3.12E-02 |  | MAPK10 |
| 5 | ILK Signaling | 5.49E-01 | 1.66E-02 |  | MAPK10,MUC1,TMSB10/TMSB4X |
| 5 | HGF Signaling | 5.41E-01 | 1.92E-02 |  | MAPK10,ELF3 |
| 5 | Circadian Rhythm Signaling | 5.41E-01 | 3.03E-02 |  | PER2 |
| 5 | MIF-mediated Glucocorticoid Regulation | 5.41E-01 | 3.03E-02 |  | PLA2G10 |
| 5 | NGF Signaling | 5.3E-01 | 1.89E-02 |  | SMPD3,MAPK10 |
| 5 | Role of Tissue Factor in Cancer | 5.24E-01 | 1.87E-02 |  | CXCL1,GNA14 |
| 5 | RAR Activation | 5.24E-01 | 1.6E-02 |  | DHRS9,MAPK10,RBP2 |
| 5 | Glucocorticoid Receptor Signaling | 5.18E-01 | 1.47E-02 |  | MAPK10,SLPI,NFAT5,SELE |
| 5 | Fc Epsilon RI Signaling | 5.13E-01 | 1.83E-02 |  | PLA2G10,MAPK10 |
| 5 | Complement System | 5.09E-01 | 2.78E-02 |  | C6 |
| 5 | CD28 Signaling in T Helper Cells | 4.92E-01 | 1.77E-02 |  | MAPK10,NFAT5 |
| 5 | Inhibition of Matrix Metalloproteases | 4.9E-01 | 2.63E-02 |  | TIMP4 |
| 5 | Type II Diabetes Mellitus Signaling | 4.87E-01 | 1.75E-02 |  | SMPD3,MAPK10 |
| 5 | Sperm Motility | 4.87E-01 | 1.75E-02 |  | PLA2G10,RARRES3 |
| 5 | Netrin Signaling | 4.81E-01 | 2.56E-02 |  | NFAT5 |
| 5 | HMGB1 Signaling | 4.68E-01 | 1.69E-02 |  | MAPK10,SELE |
| 5 | FcγRIIB Signaling in B Lymphocytes | 4.63E-01 | 2.44E-02 |  | MAPK10 |
| 5 | Role of IL-17F in Allergic Inflammatory Airway Diseases | 4.63E-01 | 2.44E-02 |  | CXCL1 |
| 5 | Dermatan Sulfate Biosynthesis (Late Stages) | 4.55E-01 | 2.38E-02 |  | HS3ST1 |
| 5 | LPS/IL-1 Mediated Inhibition of RXR Function | 4.46E-01 | 1.44E-02 |  | GSTA3,ALDH3B1,HS3ST1 |
| 5 | Hematopoiesis from Pluripotent Stem Cells | 4.39E-01 | 2.27E-02 |  | IL7 |
| 5 | Chondroitin Sulfate Biosynthesis (Late Stages) | 4.39E-01 | 2.27E-02 |  | HS3ST1 |
| 5 | FXR/RXR Activation | 4.36E-01 | 1.6E-02 |  | FOXA1,MAPK10 |
| 5 | Role of Oct4 in Mammalian Embryonic Stem Cell Pluripotency | 4.31E-01 | 2.22E-02 |  | FOXA1 |
| 5 | GNRH Signaling | 4.27E-01 | 1.57E-02 |  | MAPK10,GNA14 |
| 5 | nNOS Signaling in Neurons | 4.23E-01 | 2.17E-02 |  | CAPN9 |
| 5 | Human Embryonic Stem Cell Pluripotency | 4.11E-01 | 1.53E-02 |  | BMP15,NTF4 |
| 5 | Heparan Sulfate Biosynthesis (Late Stages) | 4.09E-01 | 2.08E-02 |  | HS3ST1 |
| 5 | Amyloid Processing | 3.95E-01 | 2E-02 |  | CAPN9 |
| 5 | CD27 Signaling in Lymphocytes | 3.89E-01 | 1.96E-02 |  | MAPK10 |
| 5 | Chondroitin Sulfate Biosynthesis | 3.82E-01 | 1.92E-02 |  | HS3ST1 |
| 5 | UVB-Induced MAPK Signaling | 3.76E-01 | 1.89E-02 |  | MAPK10 |
| 5 | Lymphotoxin β Receptor Signaling | 3.7E-01 | 1.85E-02 |  | CXCL1 |
| 5 | OX40 Signaling Pathway | 3.7E-01 | 1.85E-02 |  | MAPK10 |
| 5 | Heparan Sulfate Biosynthesis | 3.64E-01 | 1.82E-02 |  | HS3ST1 |
| 5 | Dermatan Sulfate Biosynthesis | 3.64E-01 | 1.82E-02 |  | HS3ST1 |
| 5 | Regulation of Cellular Mechanics by Calpain Protease | 3.64E-01 | 1.82E-02 |  | CAPN9 |
| 5 | Wnt/Ca+ pathway | 3.64E-01 | 1.82E-02 |  | NFAT5 |
| 5 | ErbB2-ErbB3 Signaling | 3.58E-01 | 1.79E-02 |  | NRG4 |
| 5 | Glioma Invasiveness Signaling | 3.53E-01 | 1.75E-02 |  | TIMP4 |
| 5 | Myc Mediated Apoptosis Signaling | 3.47E-01 | 1.72E-02 |  | MAPK10 |
| 5 | ErbB4 Signaling | 3.47E-01 | 1.72E-02 |  | NRG4 |
| 5 | Induction of Apoptosis by HIV1 | 3.42E-01 | 1.69E-02 |  | MAPK10 |
| 5 | ATM Signaling | 3.42E-01 | 1.69E-02 |  | MAPK10 |
| 5 | CXCR4 Signaling | 3.39E-01 | 1.32E-02 |  | MAPK10,GNA14 |
| 5 | Aldosterone Signaling in Epithelial Cells | 3.39E-01 | 1.32E-02 |  | DNAJB13,DNAJC28 |
| 5 | Activation of IRF by Cytosolic Pattern Recognition Receptors | 3.36E-01 | 1.67E-02 |  | MAPK10 |
| 5 | Role of JAK1 and JAK3 in γc Cytokine Signaling | 3.36E-01 | 1.67E-02 |  | IL7 |
| 5 | PCP pathway | 3.26E-01 | 1.61E-02 |  | MAPK10 |
| 5 | Tec Kinase Signaling | 3.23E-01 | 1.28E-02 |  | MAPK10,GNA14 |
| 5 | Mitotic Roles of Polo-Like Kinase | 3.21E-01 | 1.59E-02 |  | PLK3 |
| 5 | CD40 Signaling | 3.16E-01 | 1.56E-02 |  | MAPK10 |
| 5 | Protein Ubiquitination Pathway | 3.16E-01 | 1.18E-02 |  | DNAJB13,DNAJC28,USP2 |
| 5 | Xenobiotic Metabolism Signaling | 3.12E-01 | 1.17E-02 |  | GSTA3,ALDH3B1,HS3ST1 |
| 5 | GABA Receptor Signaling | 3.07E-01 | 1.52E-02 |  | GABRP |
| 5 | CCR5 Signaling in Macrophages | 3.02E-01 | 1.49E-02 |  | MAPK10 |
| 5 | Neurotrophin/TRK Signaling | 3.02E-01 | 1.49E-02 |  | NTF4 |
| 5 | GDNF Family Ligand-Receptor Interactions | 2.98E-01 | 1.47E-02 |  | MAPK10 |
| 5 | Granulocyte Adhesion and Diapedesis | 2.97E-01 | 1.21E-02 |  | CXCL1,SELE |
| 5 | Role of NFAT in Regulation of the Immune Response | 2.94E-01 | 1.2E-02 |  | GNA14,NFAT5 |
| 5 | Basal Cell Carcinoma Signaling | 2.94E-01 | 1.45E-02 |  | BMP15 |
| 5 | GPCR-Mediated Integration of Enteroendocrine Signaling Exemplified by an L Cell | 2.89E-01 | 1.43E-02 |  | GNA14 |
| 5 | LPS-stimulated MAPK Signaling | 2.77E-01 | 1.37E-02 |  | MAPK10 |
| 5 | Ephrin B Signaling | 2.77E-01 | 1.37E-02 |  | GNA14 |
| 5 | STAT3 Pathway | 2.77E-01 | 1.37E-02 |  | MAPK10 |
| 5 | IL-4 Signaling | 2.77E-01 | 1.37E-02 |  | NFAT5 |
| 5 | Agranulocyte Adhesion and Diapedesis | 2.7E-01 | 1.14E-02 |  | CXCL1,SELE |
| 5 | VEGF Family Ligand-Receptor Interactions | 2.65E-01 | 1.32E-02 |  | PLA2G10 |
| 5 | VDR/RXR Activation | 2.62E-01 | 1.3E-02 |  | CYP24A1 |
| 5 | Reelin Signaling in Neurons | 2.54E-01 | 1.27E-02 |  | MAPK10 |
| 5 | IL-8 Signaling | 2.51E-01 | 1.09E-02 |  | MAPK10,CXCL1 |
| 5 | Ceramide Signaling | 2.51E-01 | 1.25E-02 |  | SMPD3 |
| 5 | Role of Macrophages, Fibroblasts and Endothelial Cells in Rheumatoid Arthritis | 2.48E-01 | 1.05E-02 |  | NFAT5,SELE,IL7 |
| 5 | ERK/MAPK Signaling | 2.44E-01 | 1.08E-02 |  | PLA2G10,ELF3 |
| 5 | GPCR-Mediated Nutrient Sensing in Enteroendocrine Cells | 2.4E-01 | 1.2E-02 |  | GNA14 |
| 5 | Melanocyte Development and Pigmentation Signaling | 2.37E-01 | 1.19E-02 |  | TYR |
| 5 | Neuregulin Signaling | 2.31E-01 | 1.16E-02 |  | NRG4 |
| 5 | FAK Signaling | 2.31E-01 | 1.16E-02 |  | CAPN9 |
| 5 | RANK Signaling in Osteoclasts | 2.31E-01 | 1.16E-02 |  | MAPK10 |
| 5 | Leukocyte Extravasation Signaling | 2.29E-01 | 1.04E-02 |  | MAPK10,TIMP4 |
| 5 | G Beta Gamma Signaling | 2.25E-01 | 1.14E-02 |  | GNA14 |
| 5 | PAK Signaling | 2.25E-01 | 1.14E-02 |  | MAPK10 |
| 5 | Apoptosis Signaling | 2.25E-01 | 1.14E-02 |  | CAPN9 |
| 5 | Factors Promoting Cardiogenesis in Vertebrates | 2.22E-01 | 1.12E-02 |  | BMP15 |
| 5 | Salvage Pathways of Pyrimidine Ribonucleotides | 2.16E-01 | 1.1E-02 |  | AK7 |
| 5 | SAPK/JNK Signaling | 2.1E-01 | 1.08E-02 |  | MAPK10 |
| 5 | Telomerase Signaling | 2.02E-01 | 1.04E-02 |  | ELF3 |
| 5 | T Cell Receptor Signaling | 2.02E-01 | 1.04E-02 |  | NFAT5 |
| 5 | Amyotrophic Lateral Sclerosis Signaling | 1.99E-01 | 1.03E-02 |  | CAPN9 |
| 6 | Cell Cycle: G2/M DNA Damage Checkpoint Regulation | 6.5E00 | 1.84E-01 | -0.447 | YWHAQ,YWHAG,AURKA,SKP2,PRKDC,CKS2,CCNB2,YWHAB,CHEK1 |
| 6 | Protein Ubiquitination Pathway | 4.5E00 | 6.3E-02 |  | UCHL1,HSP90B1,CDC20,PSMD11,DNAJC19,PSMC2,DNAJC21,PSMA7,UBE2V2,HSPA5,SKP2,DNAJB11,PSMD14,PSMD8,PSMB2,PSMD2 |
| 6 | tRNA Charging | 3.14E00 | 1.32E-01 |  | EPRS,AARS,TARS,MARS,DARS |
| 6 | Mitotic Roles of Polo-Like Kinase | 2.9E00 | 9.52E-02 |  | RAD21,KIF23,HSP90B1,CDC20,PTTG1,CCNB2 |
| 6 | dTMP De Novo Biosynthesis | 2.46E00 | 4E-01 |  | SHMT2,TYMS |
| 6 | Granzyme A Signaling | 2.42E00 | 1.76E-01 |  | HIST1H1C,HMGB2,H1F0 |
| 6 | Granulocyte Adhesion and Diapedesis | 2.35E00 | 5.45E-02 |  | CXCL9,CCL22,MMP11,CXCL10,ITGB1,MMP12,MSN,MMP1,MMP14 |
| 6 | Inhibition of Matrix Metalloproteases | 2.24E00 | 1.05E-01 |  | MMP11,MMP12,MMP1,MMP14 |
| 6 | Agranulocyte Adhesion and Diapedesis | 2.19E00 | 5.14E-02 |  | CXCL9,CCL22,MMP11,CXCL10,ITGB1,MMP12,MSN,MMP1,MMP14 |
| 6 | Virus Entry via Endocytic Pathways | 2.15E00 | 6.74E-02 |  | FYN,PIK3CA,ITGB1,CLTC,AP2M1,TFRC |
| 6 | Superpathway of Serine and Glycine Biosynthesis I | 2.15E00 | 2.86E-01 |  | PSAT1,SHMT2 |
| 6 | p53 Signaling | 1.95E00 | 6.12E-02 | -0.447 | PIK3CA,HIF1A,CDK2,SERPINE2,PRKDC,CHEK1 |
| 6 | Caveolar-mediated Endocytosis Signaling | 1.94E00 | 7.04E-02 |  | FYN,ITGAV,COPB2,ITGB1,COPG1 |
| 6 | Pathogenesis of Multiple Sclerosis | 1.92E00 | 2.22E-01 |  | CXCL9,CXCL10 |
| 6 | Calcium Transport I | 1.92E00 | 2.22E-01 |  | ATP2B3,ATP2C1 |
| 6 | Folate Transformations I | 1.92E00 | 2.22E-01 |  | SHMT2,MTHFD2 |
| 6 | HIF1α Signaling | 1.91E00 | 6E-02 |  | MMP11,PIK3CA,HIF1A,MMP12,MMP1,MMP14 |
| 6 | Rac Signaling | 1.85E00 | 5.83E-02 | 2.449 | PAK2,CFL1,PIK3CA,ITGB1,CYBB,ACTR3 |
| 6 | Superpathway of Cholesterol Biosynthesis | 1.85E00 | 1.11E-01 |  | HMGCS1,ACAT2,SC5D |
| 6 | Ketogenesis | 1.83E00 | 2E-01 |  | HMGCS1,ACAT2 |
| 6 | Oleate Biosynthesis II (Animals) | 1.83E00 | 2E-01 |  | SCD,FADS2 |
| 6 | B Cell Development | 1.8E00 | 1.07E-01 |  | IGKC,IL7R,PTPRC |
| 6 | Role of Tissue Factor in Cancer | 1.78E00 | 5.61E-02 |  | FYN,CFL1,PIK3CA,ITGAV,ITGB1,MMP1 |
| 6 | Semaphorin Signaling in Neurons | 1.77E00 | 7.69E-02 |  | PAK2,FYN,CFL1,ITGB1 |
| 6 | Mevalonate Pathway I | 1.68E00 | 1.67E-01 |  | HMGCS1,ACAT2 |
| 6 | Myc Mediated Apoptosis Signaling | 1.61E00 | 6.9E-02 |  | YWHAQ,PIK3CA,YWHAG,YWHAB |
| 6 | Clathrin-mediated Endocytosis Signaling | 1.6E00 | 4.35E-02 |  | RAB7A,PIK3CA,ITGB1,CLTC,PCYOX1,AP2M1,ACTR3,TFRC |
| 6 | phagosome maturation | 1.59E00 | 5.08E-02 |  | TUBB2A,PRDX6,RAB7A,YKT6,LAMP2,CYBB |
| 6 | Aldosterone Signaling in Epithelial Cells | 1.59E00 | 4.64E-02 |  | DNAJC19,DNAJC21,ITPR2,PIK3CA,HSP90B1,HSPA5,DNAJB11 |
| 6 | ERK/MAPK Signaling | 1.58E00 | 4.3E-02 | 2.236 | YWHAQ,PAK2,FYN,PIK3CA,YWHAG,STAT1,ITGB1,YWHAB |
| 6 | Oncostatin M Signaling | 1.58E00 | 8.82E-02 |  | CHI3L1,STAT1,MMP1 |
| 6 | Role of JAK1 and JAK3 in γc Cytokine Signaling | 1.56E00 | 6.67E-02 |  | PIK3CA,STAT1,IL7R,IL2RA |
| 6 | PI3K/AKT Signaling | 1.55E00 | 4.96E-02 | 2.449 | YWHAQ,PIK3CA,YWHAG,ITGB1,HSP90B1,YWHAB |
| 6 | Leukocyte Extravasation Signaling | 1.5E00 | 4.15E-02 | 2.646 | MMP11,PIK3CA,ITGB1,MMP12,MSN,CYBB,MMP1,MMP14 |
| 6 | RAN Signaling | 1.44E00 | 1.25E-01 |  | XPO1,KPNA4 |
| 6 | Superpathway of Geranylgeranyldiphosphate Biosynthesis I (via Mevalonate) | 1.44E00 | 1.25E-01 |  | HMGCS1,ACAT2 |
| 6 | Guanine and Guanosine Salvage I | 1.42E00 | 5E-01 |  | HPRT1 |
| 6 | Putrescine Biosynthesis III | 1.42E00 | 5E-01 |  | ODC1 |
| 6 | Glycine Biosynthesis I | 1.42E00 | 5E-01 |  | SHMT2 |
| 6 | Glutamine Degradation I | 1.42E00 | 5E-01 |  | GLS |
| 6 | Paxillin Signaling | 1.4E00 | 5.1E-02 | 1.000 | PAK2,PIK3CA,ITGAV,PTPN12,ITGB1 |
| 6 | Actin Cytoskeleton Signaling | 1.31E00 | 3.81E-02 |  | PAK2,CFL1,PIK3CA,ITGB1,TRIO,MSN,ACTR3,TMSB10/TMSB4X |
| 6 | Tryptophan Degradation III (Eukaryotic) | 1.3E00 | 1.05E-01 |  | TDO2,ACAT2 |
| 6 | DNA damage-induced 14-3-3σ Signaling | 1.3E00 | 1.05E-01 |  | CDK2,CCNB2 |
| 6 | Ascorbate Recycling (Cytosolic) | 1.25E00 | 3.33E-01 |  | GSTO1 |
| 6 | Primary Immunodeficiency Signaling | 1.24E00 | 6.52E-02 |  | IGKC,IL7R,PTPRC |
| 6 | Endoplasmic Reticulum Stress Pathway | 1.22E00 | 9.52E-02 |  | HSP90B1,HSPA5 |
| 6 | Role of BRCA1 in DNA Damage Response | 1.21E00 | 5.13E-02 |  | RBBP8,STAT1,HLTF,CHEK1 |
| 6 | Ephrin A Signaling | 1.2E00 | 6.25E-02 |  | FYN,CFL1,PIK3CA |
| 6 | CD28 Signaling in T Helper Cells | 1.19E00 | 4.42E-02 |  | ITPR2,FYN,PIK3CA,PTPRC,ACTR3 |
| 6 | 14-3-3-mediated Signaling | 1.15E00 | 4.31E-02 |  | TUBB2A,YWHAQ,PIK3CA,YWHAG,YWHAB |
| 6 | Arsenate Detoxification I (Glutaredoxin) | 1.13E00 | 2.5E-01 |  | GSTO1 |
| 6 | Eumelanin Biosynthesis | 1.13E00 | 2.5E-01 |  | MIF |
| 6 | Lipid Antigen Presentation by CD1 | 1.12E00 | 8.33E-02 |  | PSAP,AP2M1 |
| 6 | Estrogen-mediated S-phase Entry | 1.12E00 | 8.33E-02 |  | SKP2,CDK2 |
| 6 | Glycolysis I | 1.12E00 | 8.33E-02 |  | TPI1,PKM |
| 6 | HIPPO signaling | 1.1E00 | 4.71E-02 |  | YWHAQ,YWHAG,SKP2,YWHAB |
| 6 | Bladder Cancer Signaling | 1.09E00 | 4.65E-02 |  | MMP11,MMP12,MMP1,MMP14 |
| 6 | FAK Signaling | 1.09E00 | 4.65E-02 |  | PAK2,FYN,PIK3CA,ITGB1 |
| 6 | Regulation of Actin-based Motility by Rho | 1.07E00 | 4.6E-02 |  | PAK2,CFL1,ITGB1,ACTR3 |
| 6 | CTLA4 Signaling in Cytotoxic T Lymphocytes | 1.07E00 | 4.6E-02 |  | FYN,PIK3CA,CLTC,AP2M1 |
| 6 | Antiproliferative Role of TOB in T Cell Signaling | 1.06E00 | 7.69E-02 |  | SKP2,CDK2 |
| 6 | Role of CHK Proteins in Cell Cycle Checkpoint Control | 1.06E00 | 5.45E-02 |  | CDK2,CLSPN,CHEK1 |
| 6 | PAK Signaling | 1.06E00 | 4.55E-02 |  | PAK2,CFL1,PIK3CA,ITGB1 |
| 6 | EGF Signaling | 1.04E00 | 5.36E-02 |  | ITPR2,PIK3CA,STAT1 |
| 6 | Tetrahydrofolate Salvage from 5,10-methenyltetrahydrofolate | 1.04E00 | 2E-01 |  | MTHFD2 |
| 6 | Serine Biosynthesis | 1.04E00 | 2E-01 |  | PSAT1 |
| 6 | Folate Polyglutamylation | 1.04E00 | 2E-01 |  | SHMT2 |
| 6 | Cell Cycle Control of Chromosomal Replication | 1.03E00 | 7.41E-02 |  | CDK2,MCM3 |
| 6 | Systemic Lupus Erythematosus Signaling | 9.93E-01 | 3.38E-02 |  | IGKC,LSM2,PIK3CA,LSM14A,EFTUD2,PTPRC,FCGR3A/FCGR3B |
| 6 | ATM Signaling | 9.9E-01 | 5.08E-02 |  | CDK2,CCNB2,CHEK1 |
| 6 | Adenine and Adenosine Salvage III | 9.63E-01 | 1.67E-01 |  | HPRT1 |
| 6 | EIF2 Signaling | 9.53E-01 | 3.49E-02 |  | EIF2S3,PIK3CA,EIF3H,RPL28,RPL39L,EIF4A2 |
| 6 | Ephrin Receptor Signaling | 9.53E-01 | 3.49E-02 |  | PAK2,MAP4K4,FYN,CFL1,ITGB1,ACTR3 |
| 6 | IGF-1 Signaling | 9.43E-01 | 4.12E-02 |  | YWHAQ,PIK3CA,YWHAG,YWHAB |
| 6 | Aryl Hydrocarbon Receptor Signaling | 9.36E-01 | 3.7E-02 | 1.000 | GSTO1,HSP90B1,CDK2,NRIP1,CHEK1 |
| 6 | ERK5 Signaling | 9.26E-01 | 4.76E-02 |  | YWHAQ,YWHAG,YWHAB |
| 6 | Hypoxia Signaling in the Cardiovascular System | 9.26E-01 | 4.76E-02 |  | HSP90B1,UBE2V2,HIF1A |
| 6 | NRF2-mediated Oxidative Stress Response | 9.1E-01 | 3.39E-02 |  | DNAJC19,DNAJC21,GSTO1,PIK3CA,CCT7,DNAJB11 |
| 6 | Role of JAK2 in Hormone-like Cytokine Signaling | 9.08E-01 | 6.25E-02 |  | STAT1,HLTF |
| 6 | Tryptophan Degradation to 2-amino-3-carboxymuconate Semialdehyde | 9E-01 | 1.43E-01 |  | TDO2 |
| 6 | Ketolysis | 9E-01 | 1.43E-01 |  | ACAT2 |
| 6 | Pregnenolone Biosynthesis | 9E-01 | 1.43E-01 |  | MICAL2 |
| 6 | Aspartate Degradation II | 9E-01 | 1.43E-01 |  | MDH2 |
| 6 | Remodeling of Epithelial Adherens Junctions | 8.82E-01 | 4.55E-02 |  | TUBB2A,RAB7A,ACTR3 |
| 6 | iCOS-iCOSL Signaling in T Helper Cells | 8.74E-01 | 3.88E-02 |  | ITPR2,PIK3CA,IL2RA,PTPRC |
| 6 | Regulation of eIF4 and p70S6K Signaling | 8.69E-01 | 3.52E-02 |  | EIF2S3,PIK3CA,ITGB1,EIF3H,EIF4A2 |
| 6 | Agrin Interactions at Neuromuscular Junction | 8.68E-01 | 4.48E-02 |  | PAK2,ITGB1,LAMC1 |
| 6 | IL-9 Signaling | 8.65E-01 | 5.88E-02 |  | PIK3CA,STAT1 |
| 6 | Oxidative Phosphorylation | 8.63E-01 | 3.85E-02 |  | COX6B1,COX7A2L,NDUFS6,NDUFB5 |
| 6 | Airway Pathology in Chronic Obstructive Pulmonary Disease | 8.46E-01 | 1.25E-01 |  | MMP1 |
| 6 | Sucrose Degradation V (Mammalian) | 8.46E-01 | 1.25E-01 |  | TPI1 |
| 6 | Phosphatidylethanolamine Biosynthesis II | 8.46E-01 | 1.25E-01 |  | EPT1 |
| 6 | Histidine Degradation III | 8.46E-01 | 1.25E-01 |  | MTHFD2 |
| 6 | Citrulline Biosynthesis | 8.46E-01 | 1.25E-01 |  | GLS |
| 6 | Salvage Pathways of Pyrimidine Deoxyribonucleotides | 8.46E-01 | 1.25E-01 |  | TK1 |
| 6 | Renal Cell Carcinoma Signaling | 8.41E-01 | 4.35E-02 |  | PAK2,PIK3CA,HIF1A |
| 6 | fMLP Signaling in Neutrophils | 8.31E-01 | 3.74E-02 | 2.000 | ITPR2,PIK3CA,CYBB,ACTR3 |
| 6 | Molecular Mechanisms of Cancer | 8.26E-01 | 2.79E-02 |  | PAK2,FYN,PIK3CA,AURKA,ITGB1,HIF1A,CDK2,PRKDC,FZD7,CHEK1 |
| 6 | Glucocorticoid Receptor Signaling | 8.25E-01 | 2.94E-02 |  | PIK3CA,STAT1,HSP90B1,HSPA5,HLTF,MMP1,FKBP4,NRIP1 |
| 6 | Natural Killer Cell Signaling | 8.21E-01 | 3.7E-02 |  | PAK2,FYN,PIK3CA,FCGR3A/FCGR3B |
| 6 | Renin-Angiotensin Signaling | 8.21E-01 | 3.7E-02 | 1.000 | ITPR2,PAK2,PIK3CA,STAT1 |
| 6 | Colorectal Cancer Metastasis Signaling | 8.15E-01 | 3.03E-02 | 2.646 | MMP11,PIK3CA,STAT1,MMP12,FZD7,MMP1,MMP14 |
| 6 | Small Cell Lung Cancer Signaling | 8.14E-01 | 4.23E-02 |  | PIK3CA,SKP2,CDK2 |
| 6 | Signaling by Rho Family GTPases | 8.01E-01 | 3E-02 | 2.449 | PAK2,CFL1,PIK3CA,ITGB1,MSN,CYBB,ACTR3 |
| 6 | NF-κB Activation by Viruses | 7.89E-01 | 4.11E-02 |  | PIK3CA,ITGAV,ITGB1 |
| 6 | Prolactin Signaling | 7.89E-01 | 4.11E-02 |  | FYN,PIK3CA,STAT1 |
| 6 | Integrin Signaling | 7.79E-01 | 3.09E-02 | 2.449 | PAK2,FYN,PIK3CA,ITGAV,ITGB1,ACTR3 |
| 6 | CCR3 Signaling in Eosinophils | 7.71E-01 | 3.54E-02 |  | ITPR2,PAK2,CFL1,PIK3CA |
| 6 | Histidine Degradation VI | 7.57E-01 | 1E-01 |  | MICAL2 |
| 6 | Glycine Betaine Degradation | 7.57E-01 | 1E-01 |  | SHMT2 |
| 6 | Germ Cell-Sertoli Cell Junction Signaling | 7.51E-01 | 3.21E-02 |  | TUBB2A,PAK2,CFL1,PIK3CA,ITGB1 |
| 6 | Tec Kinase Signaling | 7.51E-01 | 3.21E-02 | 2.236 | PAK2,FYN,PIK3CA,STAT1,ITGB1 |
| 6 | Cyclins and Cell Cycle Regulation | 7.42E-01 | 3.9E-02 |  | SKP2,CDK2,CCNB2 |
| 6 | p70S6K Signaling | 7.25E-01 | 3.39E-02 |  | YWHAQ,PIK3CA,YWHAG,YWHAB |
| 6 | Glutaryl-CoA Degradation | 7.2E-01 | 9.09E-02 |  | ACAT2 |
| 6 | Acyl-CoA Hydrolysis | 7.2E-01 | 9.09E-02 |  | PPT1 |
| 6 | Purine Nucleotides De Novo Biosynthesis II | 7.2E-01 | 9.09E-02 |  | GMPS |
| 6 | Reelin Signaling in Neurons | 7.19E-01 | 3.8E-02 |  | FYN,PIK3CA,ITGB1 |
| 6 | Axonal Guidance Signaling | 7.18E-01 | 2.58E-02 |  | TUBB2A,PAK2,FYN,CFL1,MMP11,PIK3CA,ITGB1,ADAM19,PSMD14,FZD7,ACTR3 |
| 6 | Prostate Cancer Signaling | 7.08E-01 | 3.75E-02 |  | PIK3CA,HSP90B1,CDK2 |
| 6 | Cleavage and Polyadenylation of Pre-mRNA | 6.86E-01 | 8.33E-02 |  | CPSF3 |
| 6 | PI3K Signaling in B Lymphocytes | 6.83E-01 | 3.25E-02 | 2.000 | ITPR2,FYN,PIK3CA,PTPRC |
| 6 | Role of NFAT in Regulation of the Immune Response | 6.77E-01 | 3.01E-02 |  | ITPR2,XPO1,FYN,PIK3CA,FCGR3A/FCGR3B |
| 6 | Role of Oct4 in Mammalian Embryonic Stem Cell Pluripotency | 6.75E-01 | 4.44E-02 |  | PHC3,SPP1 |
| 6 | TR/RXR Activation | 6.57E-01 | 3.53E-02 |  | TBL1XR1,PIK3CA,HIF1A |
| 6 | Dendritic Cell Maturation | 6.56E-01 | 2.96E-02 |  | IL32,PIK3CA,STAT1,FSCN1,FCGR3A/FCGR3B |
| 6 | Role of IL-17A in Psoriasis | 6.55E-01 | 7.69E-02 |  | S100A7 |
| 6 | NAD biosynthesis II (from tryptophan) | 6.55E-01 | 7.69E-02 |  | TDO2 |
| 6 | Ubiquinol-10 Biosynthesis (Eukaryotic) | 6.55E-01 | 7.69E-02 |  | MICAL2 |
| 6 | Cholesterol Biosynthesis I | 6.55E-01 | 7.69E-02 |  | SC5D |
| 6 | Cholesterol Biosynthesis II (via 24,25-dihydrolanosterol) | 6.55E-01 | 7.69E-02 |  | SC5D |
| 6 | Cholesterol Biosynthesis III (via Desmosterol) | 6.55E-01 | 7.69E-02 |  | SC5D |
| 6 | Estrogen Receptor Signaling | 6.5E-01 | 3.15E-02 |  | PHB2,MED10,PRKDC,NRIP1 |
| 6 | Role of Osteoblasts, Osteoclasts and Chondrocytes in Rheumatoid Arthritis | 6.49E-01 | 2.8E-02 |  | PIK3CA,ITGB1,SPP1,FZD7,MMP1,MMP14 |
| 6 | RhoGDI Signaling | 6.36E-01 | 2.91E-02 | -1.342 | PAK2,CFL1,ITGB1,MSN,ACTR3 |
| 6 | Cdc42 Signaling | 6.35E-01 | 3.1E-02 | 2.000 | PAK2,CFL1,ITGB1,ACTR3 |
| 6 | DNA Double-Strand Break Repair by Non-Homologous End Joining | 6.27E-01 | 7.14E-02 |  | PRKDC |
| 6 | Isoleucine Degradation I | 6.27E-01 | 7.14E-02 |  | ACAT2 |
| 6 | Superpathway of Citrulline Metabolism | 6.27E-01 | 7.14E-02 |  | GLS |
| 6 | Vitamin-C Transport | 6.27E-01 | 7.14E-02 |  | GSTO1 |
| 6 | CNTF Signaling | 6.21E-01 | 4.08E-02 |  | PIK3CA,STAT1 |
| 6 | IL-12 Signaling and Production in Macrophages | 6.2E-01 | 3.05E-02 |  | RAB7A,PIK3CA,STAT1,PCYOX1 |
| 6 | VEGF Signaling | 6.19E-01 | 3.37E-02 |  | EIF2S3,PIK3CA,HIF1A |
| 6 | PPAR Signaling | 6.1E-01 | 3.33E-02 |  | MAP4K4,HSP90B1,NRIP1 |
| 6 | Telomere Extension by Telomerase | 6.01E-01 | 6.67E-02 |  | HNRNPA1 |
| 6 | Leukotriene Biosynthesis | 6.01E-01 | 6.67E-02 |  | LTA4H |
| 6 | γ-glutamyl Cycle | 6.01E-01 | 6.67E-02 |  | GGCT |
| 6 | eNOS Signaling | 5.91E-01 | 2.96E-02 |  | ITPR2,PIK3CA,HSP90B1,HSPA5 |
| 6 | Fcγ Receptor-mediated Phagocytosis in Macrophages and Monocytes | 5.83E-01 | 3.23E-02 |  | FYN,ACTR3,FCGR3A/FCGR3B |
| 6 | ILK Signaling | 5.8E-01 | 2.76E-02 | 2.236 | CFL1,PIK3CA,ITGB1,HIF1A,TMSB10/TMSB4X |
| 6 | Granzyme B Signaling | 5.77E-01 | 6.25E-02 |  | PRKDC |
| 6 | Mismatch Repair in Eukaryotes | 5.77E-01 | 6.25E-02 |  | EXO1 |
| 6 | Parkinson's Signaling | 5.77E-01 | 6.25E-02 |  | UCHL1 |
| 6 | Unfolded protein response | 5.72E-01 | 3.77E-02 |  | HSP90B1,HSPA5 |
| 6 | IL-2 Signaling | 5.72E-01 | 3.77E-02 |  | PIK3CA,IL2RA |
| 6 | IL-8 Signaling | 5.68E-01 | 2.73E-02 | 2.236 | PAK2,MAP4K4,PIK3CA,ITGAV,CYBB |
| 6 | Nitric Oxide Signaling in the Cardiovascular System | 5.67E-01 | 3.16E-02 |  | ITPR2,PIK3CA,HSP90B1 |
| 6 | Role of IL-17A in Arthritis | 5.61E-01 | 3.7E-02 |  | PIK3CA,MMP1 |
| 6 | Telomerase Signaling | 5.58E-01 | 3.12E-02 |  | PIK3CA,HSP90B1,IL2RA |
| 6 | T Cell Receptor Signaling | 5.58E-01 | 3.12E-02 |  | FYN,PIK3CA,PTPRC |
| 6 | γ-linolenate Biosynthesis II (Animals) | 5.54E-01 | 5.88E-02 |  | FADS2 |
| 6 | Thrombopoietin Signaling | 5.5E-01 | 3.64E-02 |  | PIK3CA,STAT1 |
| 6 | Regulation of Cellular Mechanics by Calpain Protease | 5.5E-01 | 3.64E-02 |  | ITGB1,CDK2 |
| 6 | Actin Nucleation by ARP-WASP Complex | 5.39E-01 | 3.57E-02 |  | ITGB1,ACTR3 |
| 6 | Glutamate Receptor Signaling | 5.39E-01 | 3.57E-02 |  | GLS,SLC38A1 |
| 6 | Neuropathic Pain Signaling In Dorsal Horn Neurons | 5.35E-01 | 3.03E-02 |  | ITPR2,TACR1,PIK3CA |
| 6 | Glutathione Redox Reactions I | 5.34E-01 | 5.56E-02 |  | PRDX6 |
| 6 | Glioma Invasiveness Signaling | 5.29E-01 | 3.51E-02 |  | PIK3CA,ITGAV |
| 6 | Glioblastoma Multiforme Signaling | 5.24E-01 | 2.76E-02 | 2.000 | ITPR2,PIK3CA,CDK2,FZD7 |
| 6 | GADD45 Signaling | 5.14E-01 | 5.26E-02 |  | CDK2 |
| 6 | Role of Macrophages, Fibroblasts and Endothelial Cells in Rheumatoid Arthritis | 5.12E-01 | 2.44E-02 |  | IL32,PIK3CA,RYK,MIF,FZD7,MMP1,FCGR3A/FCGR3B |
| 6 | phagosome formation | 5.12E-01 | 2.94E-02 |  | PIK3CA,ITGB1,FCGR3A/FCGR3B |
| 6 | 3-phosphoinositide Biosynthesis | 5.06E-01 | 2.7E-02 |  | PIK3CA,PTPN12,HACD2,PTPRC |
| 6 | HGF Signaling | 4.97E-01 | 2.88E-02 |  | PIK3CA,ITGB1,CDK2 |
| 6 | Gap Junction Signaling | 4.89E-01 | 2.65E-02 |  | TUBB2A,ITPR2,PIK3CA,DBN1 |
| 6 | Pancreatic Adenocarcinoma Signaling | 4.83E-01 | 2.83E-02 |  | PIK3CA,STAT1,CDK2 |
| 6 | GM-CSF Signaling | 4.8E-01 | 3.23E-02 |  | PIK3CA,STAT1 |
| 6 | Eicosanoid Signaling | 4.8E-01 | 3.23E-02 |  | PRDX6,LTA4H |
| 6 | Pyrimidine Deoxyribonucleotides De Novo Biosynthesis I | 4.78E-01 | 4.76E-02 |  | TYMS |
| 6 | Pyridoxal 5'-phosphate Salvage Pathway | 4.71E-01 | 3.17E-02 |  | PAK2,CDK2 |
| 6 | Cell Cycle: G1/S Checkpoint Regulation | 4.71E-01 | 3.17E-02 |  | SKP2,CDK2 |
| 6 | Polyamine Regulation in Colon Cancer | 4.62E-01 | 4.55E-02 |  | ODC1 |
| 6 | Angiopoietin Signaling | 4.53E-01 | 3.08E-02 |  | PAK2,PIK3CA |
| 6 | Non-Small Cell Lung Cancer Signaling | 4.53E-01 | 3.08E-02 |  | ITPR2,PIK3CA |
| 6 | TCA Cycle II (Eukaryotic) | 4.47E-01 | 4.35E-02 |  | MDH2 |
| 6 | T Helper Cell Differentiation | 4.37E-01 | 2.99E-02 |  | STAT1,IL2RA |
| 6 | Role of MAPK Signaling in the Pathogenesis of Influenza | 4.37E-01 | 2.99E-02 |  | PRDX6,CXCL10 |
| 6 | IL-22 Signaling | 4.32E-01 | 4.17E-02 |  | STAT1 |
| 6 | Role of JAK1, JAK2 and TYK2 in Interferon Signaling | 4.32E-01 | 4.17E-02 |  | STAT1 |
| 6 | Triacylglycerol Degradation | 4.32E-01 | 4.17E-02 |  | PRDX6 |
| 6 | GDNF Family Ligand-Receptor Interactions | 4.29E-01 | 2.94E-02 |  | ITPR2,PIK3CA |
| 6 | Macropinocytosis Signaling | 4.29E-01 | 2.94E-02 |  | PIK3CA,ITGB1 |
| 6 | Growth Hormone Signaling | 4.21E-01 | 2.9E-02 |  | PIK3CA,STAT1 |
| 6 | IL-17A Signaling in Gastric Cells | 4.18E-01 | 4E-02 |  | CXCL10 |
| 6 | Role of JAK family kinases in IL-6-type Cytokine Signaling | 4.18E-01 | 4E-02 |  | STAT1 |
| 6 | Glutathione-mediated Detoxification | 4.18E-01 | 4E-02 |  | GSTO1 |
| 6 | Gluconeogenesis I | 4.18E-01 | 4E-02 |  | MDH2 |
| 6 | Mitochondrial Dysfunction | 4.14E-01 | 2.42E-02 |  | COX6B1,COX7A2L,NDUFS6,NDUFB5 |
| 6 | GPCR-Mediated Integration of Enteroendocrine Signaling Exemplified by an L Cell | 4.13E-01 | 2.86E-02 |  | ITPR2,GAL |
| 6 | IL-3 Signaling | 4.06E-01 | 2.82E-02 |  | PIK3CA,STAT1 |
| 6 | FLT3 Signaling in Hematopoietic Progenitor Cells | 4.06E-01 | 2.82E-02 |  | PIK3CA,STAT1 |
| 6 | Pyrimidine Ribonucleotides Interconversion | 4.05E-01 | 3.85E-02 |  | CTPS1 |
| 6 | IL-17 Signaling | 3.98E-01 | 2.78E-02 |  | PIK3CA,CXCL10 |
| 6 | JAK/Stat Signaling | 3.98E-01 | 2.78E-02 |  | PIK3CA,STAT1 |
| 6 | RhoA Signaling | 3.97E-01 | 2.5E-02 |  | CFL1,MSN,ACTR3 |
| 6 | IL-15 Production | 3.92E-01 | 3.7E-02 |  | STAT1 |
| 6 | Protein Kinase A Signaling | 3.92E-01 | 2.17E-02 |  | HIST1H1C,YWHAQ,ITPR2,YWHAG,PTPN12,PTPRC,YWHAB,H1F0 |
| 6 | Atherosclerosis Signaling | 3.91E-01 | 2.48E-02 |  | PRDX6,PCYOX1,MMP1 |
| 6 | D-myo-inositol (1,4,5,6)-Tetrakisphosphate Biosynthesis | 3.91E-01 | 2.48E-02 |  | PTPN12,HACD2,PTPRC |
| 6 | D-myo-inositol (3,4,5,6)-tetrakisphosphate Biosynthesis | 3.91E-01 | 2.48E-02 |  | PTPN12,HACD2,PTPRC |
| 6 | Pyrimidine Ribonucleotides De Novo Biosynthesis | 3.8E-01 | 3.57E-02 |  | CTPS1 |
| 6 | HER-2 Signaling in Breast Cancer | 3.7E-01 | 2.63E-02 |  | PIK3CA,ITGB1 |
| 6 | Role of p14/p19ARF in Tumor Suppression | 3.69E-01 | 3.45E-02 |  | PIK3CA |
| 6 | Huntington's Disease Signaling | 3.65E-01 | 2.21E-02 |  | PIK3CA,CLTC,HSPA5,YKT6,GLS |
| 6 | Hereditary Breast Cancer Signaling | 3.65E-01 | 2.38E-02 |  | PIK3CA,HLTF,CHEK1 |
| 6 | Adipogenesis pathway | 3.65E-01 | 2.38E-02 |  | TBL1XR1,HIF1A,FZD7 |
| 6 | VDR/RXR Activation | 3.64E-01 | 2.6E-02 |  | CXCL10,SPP1 |
| 6 | PDGF Signaling | 3.64E-01 | 2.6E-02 |  | PIK3CA,STAT1 |
| 6 | Production of Nitric Oxide and Reactive Oxygen Species in Macrophages | 3.52E-01 | 2.23E-02 | 2.000 | PIK3CA,STAT1,PCYOX1,CYBB |
| 6 | Ovarian Cancer Signaling | 3.45E-01 | 2.31E-02 |  | PIK3CA,FZD7,GJA1 |
| 6 | Human Embryonic Stem Cell Pluripotency | 3.4E-01 | 2.29E-02 |  | INHBA,PIK3CA,FZD7 |
| 6 | mTOR Signaling | 3.39E-01 | 2.2E-02 |  | PIK3CA,HIF1A,EIF3H,EIF4A2 |
| 6 | Inhibition of Angiogenesis by TSP1 | 3.37E-01 | 3.12E-02 |  | FYN |
| 6 | Superpathway of Inositol Phosphate Compounds | 3.28E-01 | 2.16E-02 |  | PIK3CA,PTPN12,HACD2,PTPRC |
| 6 | MIF-mediated Glucocorticoid Regulation | 3.28E-01 | 3.03E-02 |  | MIF |
| 6 | RAR Activation | 3.2E-01 | 2.14E-02 |  | PIK3CA,HLTF,MMP1,NRIP1 |
| 6 | Stearate Biosynthesis I (Animals) | 3.18E-01 | 2.94E-02 |  | PPT1 |
| 6 | Interferon Signaling | 3.18E-01 | 2.94E-02 |  | STAT1 |
| 6 | ErbB Signaling | 3.16E-01 | 2.35E-02 |  | PAK2,PIK3CA |
| 6 | D-myo-inositol-5-phosphate Metabolism | 3.13E-01 | 2.19E-02 |  | PTPN12,HACD2,PTPRC |
| 6 | Neuregulin Signaling | 3.1E-01 | 2.33E-02 |  | ITGB1,HSP90B1 |
| 6 | Cell Cycle Regulation by BTG Family Proteins | 3.1E-01 | 2.86E-02 |  | CDK2 |
| 6 | IL-17A Signaling in Fibroblasts | 3.1E-01 | 2.86E-02 |  | MMP1 |
| 6 | tRNA Splicing | 3.1E-01 | 2.86E-02 |  | TSEN15 |
| 6 | Breast Cancer Regulation by Stathmin1 | 3.09E-01 | 2.11E-02 |  | TUBB2A,ITPR2,PIK3CA,CDK2 |
| 6 | 3-phosphoinositide Degradation | 3.09E-01 | 2.17E-02 |  | PTPN12,HACD2,PTPRC |
| 6 | UVA-Induced MAPK Signaling | 3.05E-01 | 2.3E-02 |  | PIK3CA,STAT1 |
| 6 | autophagy | 3.01E-01 | 2.78E-02 |  | LAMP2 |
| 6 | Factors Promoting Cardiogenesis in Vertebrates | 2.94E-01 | 2.25E-02 |  | CDK2,FZD7 |
| 6 | Notch Signaling | 2.93E-01 | 2.7E-02 |  | HEY1 |
| 6 | Hepatic Fibrosis / Hepatic Stellate Cell Activation | 2.88E-01 | 2.04E-02 |  | CXCL9,COL4A1,STAT1,MMP1 |
| 6 | Salvage Pathways of Pyrimidine Ribonucleotides | 2.84E-01 | 2.2E-02 |  | PAK2,CDK2 |
| 6 | Docosahexaenoic Acid (DHA) Signaling | 2.77E-01 | 2.56E-02 |  | PIK3CA |
| 6 | Thyroid Cancer Signaling | 2.77E-01 | 2.56E-02 |  | CXCL10 |
| 6 | SAPK/JNK Signaling | 2.75E-01 | 2.15E-02 |  | MAP4K4,PIK3CA |
| 6 | Role of PKR in Interferon Induction and Antiviral Response | 2.7E-01 | 2.5E-02 |  | STAT1 |
| 6 | Mouse Embryonic Stem Cell Pluripotency | 2.7E-01 | 2.13E-02 |  | PIK3CA,FZD7 |
| 6 | Antioxidant Action of Vitamin C | 2.65E-01 | 2.11E-02 |  | PRDX6,GSTO1 |
| 6 | MIF Regulation of Innate Immunity | 2.63E-01 | 2.44E-02 |  | MIF |
| 6 | FcγRIIB Signaling in B Lymphocytes | 2.63E-01 | 2.44E-02 |  | PIK3CA |
| 6 | Mechanisms of Viral Exit from Host Cells | 2.63E-01 | 2.44E-02 |  | XPO1 |
| 6 | Role of Hypercytokinemia/hyperchemokinemia in the Pathogenesis of Influenza | 2.63E-01 | 2.44E-02 |  | CXCL10 |
| 6 | Role of IL-17F in Allergic Inflammatory Airway Diseases | 2.63E-01 | 2.44E-02 |  | CXCL10 |
| 6 | CXCR4 Signaling | 2.59E-01 | 1.99E-02 |  | ITPR2,PAK2,PIK3CA |
| 6 | Amyotrophic Lateral Sclerosis Signaling | 2.56E-01 | 2.06E-02 |  | PIK3CA,NEFL |
| 6 | Melanoma Signaling | 2.56E-01 | 2.38E-02 |  | PIK3CA |
| 6 | CDK5 Signaling | 2.52E-01 | 2.04E-02 |  | ITGB1,LAMC1 |
| 6 | iNOS Signaling | 2.49E-01 | 2.33E-02 |  | STAT1 |
| 6 | Hematopoiesis from Pluripotent Stem Cells | 2.43E-01 | 2.27E-02 |  | IGKC |
| 6 | MSP-RON Signaling Pathway | 2.31E-01 | 2.17E-02 |  | PIK3CA |
| 6 | TNFR1 Signaling | 2.25E-01 | 2.13E-02 |  | PAK2 |
| 6 | Heparan Sulfate Biosynthesis (Late Stages) | 2.2E-01 | 2.08E-02 |  | PRDX6 |
| 6 | CD27 Signaling in Lymphocytes | 2.04E-01 | 1.96E-02 |  | CD27 |
| 6 | Role of Cytokines in Mediating Communication between Immune Cells | 1.99E-01 | 1.92E-02 |  | IL32 |
| 6 | Endometrial Cancer Signaling | 1.99E-01 | 1.92E-02 |  | PIK3CA |
| 7 | Retinoate Biosynthesis I | 5.94E00 | 2.22E-01 |  | ADH7,ALDH1A1,ALDH1A3,RDH10 |
| 7 | Noradrenaline and Adrenaline Degradation | 3.95E00 | 1.43E-01 |  | ADH7,ALDH1A1,ALDH1A3 |
| 7 | Ethanol Degradation II | 3.83E00 | 1.3E-01 |  | ADH7,ALDH1A1,ALDH1A3 |
| 7 | RAR Activation | 3.24E00 | 3.36E-02 |  | ADH7,ALDH1A1,CRABP2,ALDH1A3,RDH10 |
| 7 | Serotonin Degradation | 3.2E00 | 8.11E-02 |  | ADH7,ALDH1A1,ALDH1A3 |
| 7 | Histamine Degradation | 3.05E00 | 2E-01 |  | ALDH1A1,ALDH1A3 |
| 7 | Fatty Acid α-oxidation | 2.88E00 | 1.67E-01 |  | ALDH1A1,ALDH1A3 |
| 7 | Tryptophan Degradation X (Mammalian, via Tryptamine) | 2.81E00 | 1.54E-01 |  | ALDH1A1,ALDH1A3 |
| 7 | Putrescine Degradation III | 2.81E00 | 1.54E-01 |  | ALDH1A1,ALDH1A3 |
| 7 | Oxidative Ethanol Degradation III | 2.75E00 | 1.43E-01 |  | ALDH1A1,ALDH1A3 |
| 7 | Ethanol Degradation IV | 2.68E00 | 1.33E-01 |  | ALDH1A1,ALDH1A3 |
| 7 | Dopamine Degradation | 2.58E00 | 1.18E-01 |  | ALDH1A1,ALDH1A3 |
| 7 | Glycerol Degradation I | 1.74E00 | 2.5E-01 |  | GK |
| 7 | LPS/IL-1 Mediated Inhibition of RXR Function | 1.57E00 | 2.11E-02 |  | IL18,ALDH1A1,ALDH1A3 |
| 7 | The Visual Cycle | 1.44E00 | 1.25E-01 |  | RDH10 |
| 7 | Retinol Biosynthesis | 1.18E00 | 6.67E-02 |  | RDH10 |
| 7 | Aryl Hydrocarbon Receptor Signaling | 1.16E00 | 2.11E-02 |  | ALDH1A1,ALDH1A3 |
| 7 | Graft-versus-Host Disease Signaling | 9.66E-01 | 4E-02 |  | IL18 |
| 7 | Role of Hypercytokinemia/hyperchemokinemia in the Pathogenesis of Influenza | 9.5E-01 | 3.85E-02 |  | IL18 |
| 7 | Acute Phase Response Signaling | 9.21E-01 | 1.53E-02 |  | IL18,CRABP2 |
| 7 | Role of Cytokines in Mediating Communication between Immune Cells | 9.06E-01 | 3.45E-02 |  | IL18 |
| 7 | Nicotine Degradation III | 8.92E-01 | 3.33E-02 |  | ADH7 |
| 7 | Nicotine Degradation II | 8.18E-01 | 2.78E-02 |  | ADH7 |
| 7 | T Helper Cell Differentiation | 7.87E-01 | 2.56E-02 |  | IL18 |
| 7 | Communication between Innate and Adaptive Immune Cells | 7.48E-01 | 2.33E-02 |  | IL18 |
| 7 | IL-10 Signaling | 7.39E-01 | 2.27E-02 |  | IL18 |
| 7 | Retinoic acid Mediated Apoptosis Signaling | 7.39E-01 | 2.27E-02 |  | CRABP2 |
| 7 | PXR/RXR Activation | 7.21E-01 | 2.17E-02 |  | ALDH1A1 |
| 7 | Role of PI3K/AKT Signaling in the Pathogenesis of Influenza | 7.13E-01 | 2.13E-02 |  | PLAC8 |
| 7 | TREM1 Signaling | 7.05E-01 | 2.08E-02 |  | IL18 |
| 7 | Altered T Cell and B Cell Signaling in Rheumatoid Arthritis | 6.89E-01 | 2E-02 |  | IL18 |
| 7 | Crosstalk between Dendritic Cells and Natural Killer Cells | 6.81E-01 | 1.96E-02 |  | IL18 |
| 7 | Toll-like Receptor Signaling | 6.81E-01 | 1.96E-02 |  | IL18 |
| 7 | VDR/RXR Activation | 6.67E-01 | 1.89E-02 |  | SERPINB1 |
| 7 | Xenobiotic Metabolism Signaling | 6.65E-01 | 1.05E-02 |  | ALDH1A1,ALDH1A3 |
| 7 | PCP pathway | 6.52E-01 | 1.82E-02 |  | JUNB |
| 7 | PPAR Signaling | 5.72E-01 | 1.47E-02 |  | IL18 |
| 7 | Cholecystokinin/Gastrin-mediated Signaling | 5.27E-01 | 1.3E-02 |  | IL18 |
| 7 | Atherosclerosis Signaling | 5.13E-01 | 1.25E-02 |  | IL18 |
| 7 | p38 MAPK Signaling | 5.13E-01 | 1.25E-02 |  | IL18 |
| 7 | Gustation Pathway | 5.04E-01 | 1.22E-02 |  | SCNN1B |
| 7 | LXR/RXR Activation | 4.95E-01 | 1.19E-02 |  | IL18 |
| 7 | Role of Pattern Recognition Receptors in Recognition of Bacteria and Viruses | 4.95E-01 | 1.19E-02 |  | IL18 |
| 7 | HMGB1 Signaling | 4.95E-01 | 1.19E-02 |  | IL18 |
| 7 | IL-6 Signaling | 4.83E-01 | 1.15E-02 |  | IL18 |
| 7 | FXR/RXR Activation | 4.79E-01 | 1.14E-02 |  | IL18 |
| 7 | IL-12 Signaling and Production in Macrophages | 4.38E-01 | 1.01E-02 |  | IL18 |
| 7 | eNOS Signaling | 4.38E-01 | 1.01E-02 |  | AQP3 |
| 7 | Insulin Receptor Signaling | 4.38E-01 | 1.01E-02 |  | SCNN1B |
| 7 | Hepatic Cholestasis | 4.31E-01 | 9.9E-03 |  | IL18 |
| 7 | Epithelial Adherens Junction Signaling | 4.11E-01 | 9.35E-03 |  | LMO7 |
| 7 | Agranulocyte Adhesion and Diapedesis | 4.05E-01 | 9.17E-03 |  | IL18 |
| 7 | Dendritic Cell Maturation | 3.99E-01 | 9.01E-03 |  | IL18 |
| 7 | Granulocyte Adhesion and Diapedesis | 3.99E-01 | 9.01E-03 |  | IL18 |
| 7 | Regulation of eIF4 and p70S6K Signaling | 3.84E-01 | 8.62E-03 |  | RPSA |
| 7 | Aldosterone Signaling in Epithelial Cells | 3.78E-01 | 8.47E-03 |  | SCNN1B |
| 7 | NF-κB Signaling | 3.57E-01 | 7.94E-03 |  | IL18 |
| 7 | NRF2-mediated Oxidative Stress Response | 3.47E-01 | 7.69E-03 |  | JUNB |
| 7 | PPARα/RXRα Activation | 3.45E-01 | 7.63E-03 |  | GK |
| 7 | Germ Cell-Sertoli Cell Junction Signaling | 3.4E-01 | 7.52E-03 |  | PLS1 |
| 7 | Systemic Lupus Erythematosus Signaling | 3.4E-01 | 7.52E-03 |  | IL18 |
| 7 | EIF2 Signaling | 3.28E-01 | 7.25E-03 |  | RPSA |
| 7 | Sertoli Cell-Sertoli Cell Junction Signaling | 3.24E-01 | 7.14E-03 |  | PLS1 |
| 7 | mTOR Signaling | 3.13E-01 | 6.9E-03 |  | RPSA |
| 7 | Role of Osteoblasts, Osteoclasts and Chondrocytes in Rheumatoid Arthritis | 2.83E-01 | 6.25E-03 |  | IL18 |
| 7 | Role of Macrophages, Fibroblasts and Endothelial Cells in Rheumatoid Arthritis | 1.97E-01 | 4.57E-03 |  | IL18 |
| 8 | Retinoic acid Mediated Apoptosis Signaling | 3.56E00 | 6.82E-02 |  | IFNA17,PARP9,PARP14 |
| 8 | UVA-Induced MAPK Signaling | 1.79E00 | 2.99E-02 |  | PARP9,PARP14 |
| 8 | Methylglyoxal Degradation III | 1.76E00 | 1.67E-01 |  | AKR1C1/AKR1C2 |
| 8 | Death Receptor Signaling | 1.73E00 | 2.78E-02 |  | PARP9,PARP14 |
| 8 | Bile Acid Biosynthesis, Neutral Pathway | 1.5E00 | 9.09E-02 |  | AKR1C1/AKR1C2 |
| 8 | Role of Lipids/Lipid Rafts in the Pathogenesis of Influenza | 1.4E00 | 7.14E-02 |  | IFNA17 |
| 8 | IL-22 Signaling | 1.32E00 | 5.88E-02 |  | SOCS3 |
| 8 | Role of JAK family kinases in IL-6-type Cytokine Signaling | 1.27E00 | 5.26E-02 |  | SOCS3 |
| 8 | Systemic Lupus Erythematosus Signaling | 1.24E00 | 1.5E-02 |  | IFNA17,LSM14B |
| 8 | IL-9 Signaling | 1.15E00 | 4E-02 |  | SOCS3 |
| 8 | Role of Hypercytokinemia/hyperchemokinemia in the Pathogenesis of Influenza | 1.14E00 | 3.85E-02 |  | IFNA17 |
| 8 | Role of JAK2 in Hormone-like Cytokine Signaling | 1.14E00 | 3.85E-02 |  | SOCS3 |
| 8 | Role of Cytokines in Mediating Communication between Immune Cells | 1.09E00 | 3.45E-02 |  | IFNA17 |
| 8 | Role of RIG1-like Receptors in Antiviral Innate Immunity | 1.02E00 | 2.94E-02 |  | IFNA17 |
| 8 | IL-10 Signaling | 9.19E-01 | 2.27E-02 |  | SOCS3 |
| 8 | Role of JAK1 and JAK3 in γc Cytokine Signaling | 9.01E-01 | 2.17E-02 |  | SOCS3 |
| 8 | Role of PI3K/AKT Signaling in the Pathogenesis of Influenza | 8.92E-01 | 2.13E-02 |  | IFNA17 |
| 8 | Role of Macrophages, Fibroblasts and Endothelial Cells in Rheumatoid Arthritis | 8.76E-01 | 9.13E-03 |  | PRSS1,SOCS3 |
| 8 | Activation of IRF by Cytosolic Pattern Recognition Receptors | 8.75E-01 | 2.04E-02 |  | IFNA17 |
| 8 | Growth Hormone Signaling | 8.75E-01 | 2.04E-02 |  | SOCS3 |
| 8 | Erythropoietin Signaling | 8.59E-01 | 1.96E-02 |  | SOCS3 |
| 8 | GABA Receptor Signaling | 8.51E-01 | 1.92E-02 |  | GABRE |
| 8 | JAK/Stat Signaling | 8.44E-01 | 1.89E-02 |  | SOCS3 |
| 8 | Leptin Signaling in Obesity | 8.36E-01 | 1.85E-02 |  | SOCS3 |
| 8 | CTLA4 Signaling in Cytotoxic T Lymphocytes | 8.07E-01 | 1.72E-02 |  | CLTC |
| 8 | Prolactin Signaling | 8.01E-01 | 1.69E-02 |  | SOCS3 |
| 8 | Role of Wnt/GSK-3β Signaling in the Pathogenesis of Influenza | 7.94E-01 | 1.67E-02 |  | IFNA17 |
| 8 | Virus Entry via Endocytic Pathways | 7.75E-01 | 1.59E-02 |  | CLTC |
| 8 | STAT3 Pathway | 7.75E-01 | 1.59E-02 |  | SOCS3 |
| 8 | Reelin Signaling in Neurons | 7.56E-01 | 1.52E-02 |  | LRP8 |
| 8 | TR/RXR Activation | 7.44E-01 | 1.47E-02 |  | AKR1C1/AKR1C2 |
| 8 | Type I Diabetes Mellitus Signaling | 7.01E-01 | 1.32E-02 |  | SOCS3 |
| 8 | IGF-1 Signaling | 6.91E-01 | 1.28E-02 |  | SOCS3 |
| 8 | Gustation Pathway | 6.71E-01 | 1.22E-02 |  | TAS2R31 |
| 8 | IL-6 Signaling | 6.49E-01 | 1.15E-02 |  | SOCS3 |
| 8 | D-myo-inositol (1,4,5,6)-Tetrakisphosphate Biosynthesis | 6.4E-01 | 1.12E-02 |  | SOCS3 |
| 8 | D-myo-inositol (3,4,5,6)-tetrakisphosphate Biosynthesis | 6.4E-01 | 1.12E-02 |  | SOCS3 |
| 8 | Type II Diabetes Mellitus Signaling | 6.32E-01 | 1.1E-02 |  | SOCS3 |
| 8 | IL-12 Signaling and Production in Macrophages | 6E-01 | 1.01E-02 |  | IFNA17 |
| 8 | Insulin Receptor Signaling | 6E-01 | 1.01E-02 |  | SOCS3 |
| 8 | Hepatic Cholestasis | 5.92E-01 | 9.9E-03 |  | ABCC1 |
| 8 | D-myo-inositol-5-phosphate Metabolism | 5.92E-01 | 9.9E-03 |  | SOCS3 |
| 8 | 3-phosphoinositide Degradation | 5.92E-01 | 9.9E-03 |  | SOCS3 |
| 8 | 3-phosphoinositide Biosynthesis | 5.64E-01 | 9.17E-03 |  | SOCS3 |
| 8 | Agranulocyte Adhesion and Diapedesis | 5.64E-01 | 9.17E-03 |  | XCL1 |
| 8 | Granulocyte Adhesion and Diapedesis | 5.57E-01 | 9.01E-03 |  | XCL1 |
| 8 | NRF2-mediated Oxidative Stress Response | 5E-01 | 7.69E-03 |  | ABCC1 |
| 8 | Acute Phase Response Signaling | 4.97E-01 | 7.63E-03 |  | SOCS3 |
| 8 | Superpathway of Inositol Phosphate Compounds | 4.81E-01 | 7.3E-03 |  | SOCS3 |
| 8 | EIF2 Signaling | 4.78E-01 | 7.25E-03 |  | RPL41 |
| 8 | Clathrin-mediated Endocytosis Signaling | 4.71E-01 | 7.09E-03 |  | CLTC |
| 8 | Hepatic Fibrosis / Hepatic Stellate Cell Activation | 4.66E-01 | 6.99E-03 |  | KLF6 |
| 8 | Huntington's Disease Signaling | 4.08E-01 | 5.92E-03 |  | CLTC |
| 8 | Axonal Guidance Signaling | 2.13E-01 | 3.12E-03 |  | ADAM23 |
| 9 | Spliceosomal Cycle | 2.29E00 | 5E-01 |  | LOC102724594/U2AF1 |
| 9 | Spermine and Spermidine Degradation I | 2.11E00 | 3.33E-01 |  | SAT1 |
| 9 | p53 Signaling | 1.79E00 | 2.67E-02 |  | SERPINB5,SFN |
| 9 | Role of IL-17A in Psoriasis | 1.59E00 | 1E-01 |  | S100A9 |
| 9 | DNA damage-induced 14-3-3σ Signaling | 1.51E00 | 8.33E-02 |  | SFN |
| 9 | Putrescine Degradation III | 1.48E00 | 7.69E-02 |  | SAT1 |
| 9 | Polyamine Regulation in Colon Cancer | 1.34E00 | 5.56E-02 |  | SAT1 |
| 9 | Thyroid Hormone Metabolism II (via Conjugation and/or Degradation) | 1.32E00 | 5.26E-02 |  | UGT1A9 (includes others) |
| 9 | Graft-versus-Host Disease Signaling | 1.2E00 | 4E-02 |  | IL1RN |
| 9 | Role of Hypercytokinemia/hyperchemokinemia in the Pathogenesis of Influenza | 1.18E00 | 3.85E-02 |  | IL1RN |
| 9 | Role of Cytokines in Mediating Communication between Immune Cells | 1.14E00 | 3.45E-02 |  | IL1RN |
| 9 | Notch Signaling | 1.14E00 | 3.45E-02 |  | JAG1 |
| 9 | Nicotine Degradation III | 1.12E00 | 3.33E-02 |  | UGT1A9 (includes others) |
| 9 | Netrin Signaling | 1.08E00 | 3.03E-02 |  | ABLIM1 |
| 9 | Melatonin Degradation I | 1.07E00 | 2.94E-02 |  | UGT1A9 (includes others) |
| 9 | Nicotine Degradation II | 1.05E00 | 2.78E-02 |  | UGT1A9 (includes others) |
| 9 | Superpathway of Melatonin Degradation | 1.05E00 | 2.78E-02 |  | UGT1A9 (includes others) |
| 9 | Serotonin Degradation | 1.04E00 | 2.7E-02 |  | UGT1A9 (includes others) |
| 9 | Cell Cycle: G2/M DNA Damage Checkpoint Regulation | 1.03E00 | 2.63E-02 |  | SFN |
| 9 | Communication between Innate and Adaptive Immune Cells | 9.75E-01 | 2.33E-02 |  | IL1RN |
| 9 | IL-10 Signaling | 9.66E-01 | 2.27E-02 |  | IL1RN |
| 9 | Myc Mediated Apoptosis Signaling | 9.57E-01 | 2.22E-02 |  | SFN |
| 9 | PXR/RXR Activation | 9.48E-01 | 2.17E-02 |  | UGT1A9 (includes others) |
| 9 | ERK5 Signaling | 9.39E-01 | 2.13E-02 |  | SFN |
| 9 | Acute Myeloid Leukemia Signaling | 9.14E-01 | 2E-02 |  | JUP |
| 9 | Altered T Cell and B Cell Signaling in Rheumatoid Arthritis | 9.14E-01 | 2E-02 |  | IL1RN |
| 9 | Toll-like Receptor Signaling | 9.05E-01 | 1.96E-02 |  | IL1RN |
| 9 | Hypoxia Signaling in the Cardiovascular System | 9.05E-01 | 1.96E-02 |  | UBE2D3 |
| 9 | TGF-β Signaling | 7.96E-01 | 1.49E-02 |  | RNF111 |
| 9 | PPAR Signaling | 7.9E-01 | 1.47E-02 |  | IL1RN |
| 9 | HIPPO signaling | 7.84E-01 | 1.45E-02 |  | SFN |
| 9 | VEGF Signaling | 7.78E-01 | 1.43E-02 |  | SFN |
| 9 | Corticotropin Releasing Hormone Signaling | 7.4E-01 | 1.3E-02 |  | IVL |
| 9 | Cholecystokinin/Gastrin-mediated Signaling | 7.4E-01 | 1.3E-02 |  | IL1RN |
| 9 | IGF-1 Signaling | 7.35E-01 | 1.28E-02 |  | SFN |
| 9 | Atherosclerosis Signaling | 7.25E-01 | 1.25E-02 |  | IL1RN |
| 9 | p38 MAPK Signaling | 7.25E-01 | 1.25E-02 |  | IL1RN |
| 9 | LXR/RXR Activation | 7.06E-01 | 1.19E-02 |  | IL1RN |
| 9 | IL-6 Signaling | 6.93E-01 | 1.15E-02 |  | IL1RN |
| 9 | FXR/RXR Activation | 6.88E-01 | 1.14E-02 |  | IL1RN |
| 9 | 14-3-3-mediated Signaling | 6.67E-01 | 1.08E-02 |  | SFN |
| 9 | p70S6K Signaling | 6.63E-01 | 1.06E-02 |  | SFN |
| 9 | Hereditary Breast Cancer Signaling | 6.55E-01 | 1.04E-02 |  | SFN |
| 9 | PI3K/AKT Signaling | 6.39E-01 | 1E-02 |  | SFN |
| 9 | Hepatic Cholestasis | 6.35E-01 | 9.9E-03 |  | IL1RN |
| 9 | Epithelial Adherens Junction Signaling | 6.13E-01 | 9.35E-03 |  | JUP |
| 9 | Agranulocyte Adhesion and Diapedesis | 6.06E-01 | 9.17E-03 |  | IL1RN |
| 9 | Dendritic Cell Maturation | 6E-01 | 9.01E-03 |  | IL1RN |
| 9 | Granulocyte Adhesion and Diapedesis | 6E-01 | 9.01E-03 |  | IL1RN |
| 9 | NF-κB Signaling | 5.52E-01 | 7.94E-03 |  | IL1RN |
| 9 | NRF2-mediated Oxidative Stress Response | 5.41E-01 | 7.69E-03 |  | CBR1 |
| 9 | ILK Signaling | 5.41E-01 | 7.69E-03 |  | DSP |
| 9 | Acute Phase Response Signaling | 5.38E-01 | 7.63E-03 |  | IL1RN |
| 9 | Germ Cell-Sertoli Cell Junction Signaling | 5.32E-01 | 7.52E-03 |  | JUP |
| 9 | Systemic Lupus Erythematosus Signaling | 5.32E-01 | 7.52E-03 |  | IL1RN |
| 9 | Sertoli Cell-Sertoli Cell Junction Signaling | 5.14E-01 | 7.14E-03 |  | JUP |
| 9 | Regulation of the Epithelial-Mesenchymal Transition Pathway | 5.11E-01 | 7.09E-03 |  | JAG1 |
| 9 | LPS/IL-1 Mediated Inhibition of RXR Function | 5.09E-01 | 7.04E-03 |  | IL1RN |
| 9 | Role of Osteoblasts, Osteoclasts and Chondrocytes in Rheumatoid Arthritis | 4.66E-01 | 6.25E-03 |  | IL1RN |
| 9 | Xenobiotic Metabolism Signaling | 4.05E-01 | 5.24E-03 |  | UGT1A9 (includes others) |
| 9 | Protein Ubiquitination Pathway | 3.88E-01 | 4.98E-03 |  | UBE2D3 |
| 9 | Glucocorticoid Receptor Signaling | 3.74E-01 | 4.76E-03 |  | IL1RN |
| 9 | Role of Macrophages, Fibroblasts and Endothelial Cells in Rheumatoid Arthritis | 3.6E-01 | 4.57E-03 |  | IL1RN |
| 9 | Protein Kinase A Signaling | 2.9E-01 | 3.65E-03 |  | SFN |
| 9 | Axonal Guidance Signaling | 2.44E-01 | 3.12E-03 |  | ABLIM1 |
| 10 | Complement System | 5.22E00 | 1.6E-01 |  | CD59,CD55,CFD,CFH |
| 10 | Inhibition of Matrix Metalloproteases | 2.19E00 | 8E-02 |  | A2M,MMP10 |
| 10 | Arsenate Detoxification I (Glutaredoxin) | 1.71E00 | 2.5E-01 |  | AS3MT |
| 10 | Retinoic acid Mediated Apoptosis Signaling | 1.71E00 | 4.55E-02 |  | CFLAR,PARP8 |
| 10 | Oleate Biosynthesis II (Animals) | 1.47E00 | 1.43E-01 |  | CYB5A |
| 10 | Death Receptor Signaling | 1.32E00 | 2.78E-02 |  | CFLAR,PARP8 |
| 10 | IGF-1 Signaling | 1.26E00 | 2.56E-02 |  | CYR61,CTGF |
| 10 | Role of Tissue Factor in Cancer | 1.22E00 | 2.44E-02 |  | CYR61,CTGF |
| 10 | γ-linolenate Biosynthesis II (Animals) | 1.21E00 | 7.69E-02 |  | CYB5A |
| 10 | Coagulation System | 8.79E-01 | 3.45E-02 |  | A2M |
| 10 | Phospholipases | 8.15E-01 | 2.94E-02 |  | PLA2G16 |
| 10 | Eicosanoid Signaling | 8.15E-01 | 2.94E-02 |  | PLA2G16 |
| 10 | Hepatic Fibrosis / Hepatic Stellate Cell Activation | 8.13E-01 | 1.4E-02 |  | A2M,CTGF |
| 10 | Role of Oct4 in Mammalian Embryonic Stem Cell Pluripotency | 7.92E-01 | 2.78E-02 |  | ETS2 |
| 10 | Integrin Signaling | 7.71E-01 | 1.32E-02 |  | TSPAN2,TSPAN6 |
| 10 | Role of MAPK Signaling in the Pathogenesis of Influenza | 6.87E-01 | 2.13E-02 |  | PLA2G16 |
| 10 | Growth Hormone Signaling | 6.71E-01 | 2.04E-02 |  | A2M |
| 10 | Neurotrophin/TRK Signaling | 6.63E-01 | 2E-02 |  | SPRY1 |
| 10 | Caveolar-mediated Endocytosis Signaling | 6.56E-01 | 1.96E-02 |  | CD55 |
| 10 | PEDF Signaling | 6.2E-01 | 1.79E-02 |  | CFLAR |
| 10 | Bladder Cancer Signaling | 6E-01 | 1.69E-02 |  | MMP10 |
| 10 | Ceramide Signaling | 5.94E-01 | 1.67E-02 |  | S1PR1 |
| 10 | Antioxidant Action of Vitamin C | 5.94E-01 | 1.67E-02 |  | PLA2G16 |
| 10 | Oxidative Phosphorylation | 5.82E-01 | 1.61E-02 |  | CYB5A |
| 10 | Virus Entry via Endocytic Pathways | 5.76E-01 | 1.59E-02 |  | CD55 |
| 10 | HIF1α Signaling | 5.53E-01 | 1.49E-02 |  | MMP10 |
| 10 | UVA-Induced MAPK Signaling | 5.53E-01 | 1.49E-02 |  | PARP8 |
| 10 | Nitric Oxide Signaling in the Cardiovascular System | 5.48E-01 | 1.47E-02 |  | PLN |
| 10 | Telomerase Signaling | 5.27E-01 | 1.39E-02 |  | ETS2 |
| 10 | Sperm Motility | 5.17E-01 | 1.35E-02 |  | PLA2G16 |
| 10 | Atherosclerosis Signaling | 4.89E-01 | 1.25E-02 |  | PLA2G16 |
| 10 | Sphingosine-1-phosphate Signaling | 4.85E-01 | 1.23E-02 |  | S1PR1 |
| 10 | HGF Signaling | 4.59E-01 | 1.15E-02 |  | ETS2 |
| 10 | IL-6 Signaling | 4.59E-01 | 1.15E-02 |  | A2M |
| 10 | Synaptic Long Term Depression | 4.44E-01 | 1.1E-02 |  | PLA2G16 |
| 10 | Gαi Signaling | 4.4E-01 | 1.09E-02 |  | S1PR1 |
| 10 | Cardiac β-adrenergic Signaling | 4.18E-01 | 1.02E-02 |  | PLN |
| 10 | Human Embryonic Stem Cell Pluripotency | 3.95E-01 | 9.52E-03 |  | S1PR1 |
| 10 | Mitochondrial Dysfunction | 3.83E-01 | 9.17E-03 |  | CYB5A |
| 10 | Agranulocyte Adhesion and Diapedesis | 3.83E-01 | 9.17E-03 |  | MMP10 |
| 10 | Granulocyte Adhesion and Diapedesis | 3.77E-01 | 9.01E-03 |  | MMP10 |
| 10 | Endothelin-1 Signaling | 3.71E-01 | 8.85E-03 |  | PLA2G16 |
| 10 | Axonal Guidance Signaling | 3.31E-01 | 6.23E-03 |  | MMP10,ADAM28 |
| 10 | Acute Phase Response Signaling | 3.24E-01 | 7.63E-03 |  | A2M |
| 10 | Germ Cell-Sertoli Cell Junction Signaling | 3.19E-01 | 7.52E-03 |  | A2M |
| 10 | Sertoli Cell-Sertoli Cell Junction Signaling | 3.03E-01 | 7.14E-03 |  | A2M |
| 10 | Leukocyte Extravasation Signaling | 3.01E-01 | 7.09E-03 |  | MMP10 |
| 10 | ERK/MAPK Signaling | 3.01E-01 | 7.09E-03 |  | ETS2 |
| 10 | cAMP-mediated signaling | 2.57E-01 | 6.1E-03 |  | S1PR1 |
| 10 | Huntington's Disease Signaling | 2.48E-01 | 5.92E-03 |  | IFT57 |
| 10 | Colorectal Cancer Metastasis Signaling | 2.31E-01 | 5.56E-03 |  | MMP10 |
| 10 | G-Protein Coupled Receptor Signaling | 2.12E-01 | 5.18E-03 |  | S1PR1 |
| 11 | Hepatic Fibrosis / Hepatic Stellate Cell Activation | 1.56E01 | 1.54E-01 |  | PDGFRB,COL10A1,MMP2,COL6A3,MMP9,COL3A1,IGFBP5,COL1A2,COL1A1,COL6A2,COL5A1,VCAM1,TIMP2,CXCL8,PDGFC,COL12A1,COL5A2,FN1,COL11A1,CCL2,COL4A2,TIMP1 |
| 11 | Atherosclerosis Signaling | 9.68E00 | 1.63E-01 |  | COL10A1,MMP9,COL3A1,COL1A2,COL1A1,VCAM1,CXCL8,PDGFC,LYZ,CXCR4,GLG1,CCL2,SERPINA1 |
| 11 | Inhibition of Matrix Metalloproteases | 7.18E00 | 2.8E-01 |  | MMP7,MMP2,MMP9,THBS2,ADAM12,TIMP1,TIMP2 |
| 11 | Leukocyte Extravasation Signaling | 4.99E00 | 7.8E-02 | 1.265 | MMP7,CXCR4,MMP2,MMP9,WIPF1,TIMP1,THY1,RAP1A,EDIL3,VCAM1,TIMP2 |
| 11 | Agranulocyte Adhesion and Diapedesis | 4.37E00 | 8.26E-02 |  | MMP7,CXCR4,GLG1,FN1,MMP2,MMP9,CCL2,VCAM1,CXCL8 |
| 11 | Granulocyte Adhesion and Diapedesis | 4.31E00 | 8.11E-02 |  | MMP7,CXCR4,GLG1,MMP2,MMP9,CCL2,THY1,VCAM1,CXCL8 |
| 11 | Airway Pathology in Chronic Obstructive Pulmonary Disease | 4.17E00 | 5E-01 |  | MMP2,MMP9,CXCL8 |
| 11 | IL-8 Signaling | 4.1E00 | 6.85E-02 | 1.000 | CCND2,MMP2,MMP9,GNB2L1,RHOB,PTGS2,GNG2,VCAM1,CXCL8,PDGFC |
| 11 | ILK Signaling | 3.78E00 | 6.92E-02 | 0.000 | VIM,FN1,MMP9,FLNA,RHOB,LEF1,PTGS2,LIMS1,PDGFC |
| 11 | Bladder Cancer Signaling | 3.57E00 | 1.02E-01 |  | MMP7,MMP2,MMP9,PA2G4,CXCL8,PDGFC |
| 11 | Intrinsic Prothrombin Activation Pathway | 3.51E00 | 1.82E-01 |  | COL10A1,COL3A1,COL1A2,COL1A1 |
| 11 | HIF1α Signaling | 3.27E00 | 8.96E-02 |  | MMP7,MMP2,MMP9,LDHA,PDGFC,LDHB |
| 11 | Glioma Invasiveness Signaling | 3.19E00 | 1.09E-01 | -0.447 | MMP2,MMP9,RHOB,TIMP1,TIMP2 |
| 11 | Pyruvate Fermentation to Lactate | 3.16E00 | 6.67E-01 |  | LDHA,LDHB |
| 11 | Ovarian Cancer Signaling | 2.98E00 | 6.73E-02 |  | MMP7,MMP2,MMP9,LEF1,PTGS2,PA2G4,PDGFC |
| 11 | Colorectal Cancer Metastasis Signaling | 2.75E00 | 5E-02 | 1.414 | MMP7,MMP2,MMP9,GNB2L1,RHOB,LEF1,PTGS2,GNG2,PDGFC |
| 11 | Protein Kinase A Signaling | 2.49E00 | 4.01E-02 |  | HIST1H1A,KDELR2,FLNA,GNB2L1,ATF1,LEF1,PTGS2,H3F3A/H3F3B,GNG2,RAP1A,ANAPC11 |
| 11 | Regulation of the Epithelial-Mesenchymal Transition Pathway | 2.23E00 | 4.96E-02 |  | PDGFRB,MMP2,MMP9,LEF1,ZEB1,ETS1,GAB1 |
| 11 | LXR/RXR Activation | 2.04E00 | 5.95E-02 | 1.342 | LYZ,MMP9,CCL2,PTGS2,SERPINA1 |
| 11 | IL-17 Signaling | 2.01E00 | 7.27E-02 |  | CCL2,PTGS2,TIMP1,CXCL8 |
| 11 | Axonal Guidance Signaling | 1.99E00 | 3.43E-02 |  | MMP7,TUBB4A,CXCR4,MMP2,MMP9,GNB2L1,ADAM12,WIPF1,GNG2,RAP1A,PDGFC |
| 11 | Renal Cell Carcinoma Signaling | 1.91E00 | 6.78E-02 |  | UBB,ETS1,GAB1,RAP1A |
| 11 | Ephrin B Signaling | 1.88E00 | 6.67E-02 |  | CXCR4,GNB2L1,CAP1,GNG2 |
| 11 | Lipoate Biosynthesis and Incorporation II | 1.82E00 | 1E00 |  | LIPT1 |
| 11 | Lipoate Salvage and Modification | 1.82E00 | 1E00 |  | LIPT1 |
| 11 | Ephrin Receptor Signaling | 1.72E00 | 4.35E-02 |  | CXCR4,GNB2L1,WIPF1,GNG2,RAP1A,PDGFC |
| 11 | Role of IL-17A in Arthritis | 1.67E00 | 7.69E-02 |  | CCL2,PTGS2,CXCL8 |
| 11 | Dendritic Cell Maturation | 1.56E00 | 4.5E-02 |  | CD83,COL10A1,COL3A1,COL1A2,COL1A1 |
| 11 | Diphthamide Biosynthesis | 1.52E00 | 5E-01 |  | EEF2 |
| 11 | Pancreatic Adenocarcinoma Signaling | 1.44E00 | 4.88E-02 |  | MMP9,PTGS2,PA2G4,PDGFC |
| 11 | TREM1 Signaling | 1.44E00 | 6.25E-02 |  | CD83,CCL2,CXCL8 |
| 11 | HMGB1 Signaling | 1.4E00 | 4.76E-02 | -1.000 | RHOB,CCL2,VCAM1,CXCL8 |
| 11 | Androgen Signaling | 1.37E00 | 4.65E-02 |  | CALR,POLR2H,GNB2L1,GNG2 |
| 11 | CD40 Signaling | 1.37E00 | 5.88E-02 |  | TNFAIP3,ATF1,PTGS2 |
| 11 | HGF Signaling | 1.36E00 | 4.6E-02 |  | PTGS2,ETS1,GAB1,RAP1A |
| 11 | G Protein Signaling Mediated by Tubby | 1.32E00 | 8.7E-02 |  | GNB2L1,GNG2 |
| 11 | RhoGDI Signaling | 1.32E00 | 3.88E-02 |  | GNB2L1,RHOB,GNG2,CDH11,ARHGDIB |
| 11 | Relaxin Signaling | 1.31E00 | 4.44E-02 |  | MMP9,GNB2L1,GNG2,RAP1A |
| 11 | Role of Macrophages, Fibroblasts and Endothelial Cells in Rheumatoid Arthritis | 1.29E00 | 3.2E-02 |  | FN1,CCL2,LEF1,SFRP2,VCAM1,CXCL8,PDGFC |
| 11 | Gαi Signaling | 1.28E00 | 4.35E-02 |  | GNB2L1,GNG2,RAP1A,NPR3 |
| 11 | Signaling by Rho Family GTPases | 1.27E00 | 3.39E-02 | 0.000 | VIM,GNB2L1,RHOB,WIPF1,GNG2,CDH11 |
| 11 | Complement System | 1.26E00 | 8E-02 |  | C1S,C1R |
| 11 | Role of Hypercytokinemia/hyperchemokinemia in the Pathogenesis of Influenza | 1.23E00 | 7.69E-02 |  | CCL2,CXCL8 |
| 11 | Zymosterol Biosynthesis | 1.22E00 | 2.5E-01 |  | MSMO1 |
| 11 | Clathrin-mediated Endocytosis Signaling | 1.19E00 | 3.55E-02 |  | LYZ,SNAP91,UBB,SERPINA1,PDGFC |
| 11 | Role of IL-17F in Allergic Inflammatory Airway Diseases | 1.17E00 | 7.14E-02 |  | CCL2,CXCL8 |
| 11 | Pentose Phosphate Pathway (Non-oxidative Branch) | 1.13E00 | 2E-01 |  | RPE |
| 11 | Leucine Degradation I | 1.13E00 | 2E-01 |  | BCAT1 |
| 11 | UDP-N-acetyl-D-glucosamine Biosynthesis II | 1.13E00 | 2E-01 |  | FMO3 |
| 11 | Ketolysis | 1.13E00 | 2E-01 |  | OXCT1 |
| 11 | Integrin Signaling | 1.08E00 | 3.29E-02 | 1.000 | TSPAN3,RHOB,WIPF1,LIMS1,RAP1A |
| 11 | Mitochondrial Dysfunction | 1.07E00 | 3.67E-02 |  | COX7B,GPX7,NDUFA4L2,COX6C |
| 11 | CXCR4 Signaling | 1.07E00 | 3.67E-02 |  | CXCR4,GNB2L1,RHOB,GNG2 |
| 11 | PPAR Signaling | 1.07E00 | 4.41E-02 |  | PDGFRB,PTGS2,PDGFC |
| 11 | Glioma Signaling | 1.03E00 | 4.23E-02 |  | PDGFRB,PA2G4,PDGFC |
| 11 | Calcium Signaling | 9.94E-01 | 3.45E-02 |  | CALR,TPM2,TPM1,RAP1A |
| 11 | Glucocorticoid Receptor Signaling | 9.89E-01 | 2.86E-02 |  | TAF13,POLR2H,CCL2,PTGS2,VCAM1,CXCL8 |
| 11 | Glioblastoma Multiforme Signaling | 9.64E-01 | 3.36E-02 | 1.000 | PDGFRB,RHOB,LEF1,PDGFC |
| 11 | Prostanoid Biosynthesis | 9.37E-01 | 1.25E-01 |  | PTGS2 |
| 11 | Pentose Phosphate Pathway | 9.37E-01 | 1.25E-01 |  | RPE |
| 11 | Assembly of RNA Polymerase II Complex | 9.25E-01 | 5.13E-02 |  | TAF13,POLR2H |
| 11 | phagosome maturation | 9.22E-01 | 3.8E-02 |  | CALR,TUBB4A,ATP6V1B2 |
| 11 | Sphingosine-1-phosphate Signaling | 8.98E-01 | 3.7E-02 |  | PDGFRB,RHOB,PDGFC |
| 11 | Gαs Signaling | 8.98E-01 | 3.7E-02 |  | GNB2L1,GNG2,RAP1A |
| 11 | Isoleucine Degradation I | 8.89E-01 | 1.11E-01 |  | BCAT1 |
| 11 | Cholesterol Biosynthesis I | 8.89E-01 | 1.11E-01 |  | MSMO1 |
| 11 | Cholesterol Biosynthesis II (via 24,25-dihydrolanosterol) | 8.89E-01 | 1.11E-01 |  | MSMO1 |
| 11 | Cholesterol Biosynthesis III (via Desmosterol) | 8.89E-01 | 1.11E-01 |  | MSMO1 |
| 11 | Antiproliferative Role of Somatostatin Receptor 2 | 8.72E-01 | 4.76E-02 |  | GNB2L1,GNG2 |
| 11 | Communication between Innate and Adaptive Immune Cells | 8.55E-01 | 4.65E-02 |  | CD83,CXCL8 |
| 11 | Acute Phase Response Signaling | 8.53E-01 | 3.05E-02 |  | FN1,SERPINA1,C1S,C1R |
| 11 | Role of IL-17A in Psoriasis | 8.46E-01 | 1E-01 |  | CXCL8 |
| 11 | Wnt/β-catenin Signaling | 8.2E-01 | 2.96E-02 |  | MMP7,LEF1,UBB,SFRP2 |
| 11 | CCR5 Signaling in Macrophages | 8.09E-01 | 4.35E-02 |  | GNB2L1,GNG2 |
| 11 | Differential Regulation of Cytokine Production in Macrophages and T Helper Cells by IL-17A and IL-17F | 8.08E-01 | 9.09E-02 |  | CCL2 |
| 11 | EIF2 Signaling | 7.96E-01 | 2.9E-02 |  | RPS23,RPL18,RPL30,RPS25 |
| 11 | Role of MAPK Signaling in the Pathogenesis of Influenza | 7.94E-01 | 4.26E-02 |  | CCL2,PTGS2 |
| 11 | Production of Nitric Oxide and Reactive Oxygen Species in Macrophages | 7.88E-01 | 2.88E-02 |  | LYZ,RHOB,SERPINA1,RAP1A |
| 11 | Actin Nucleation by ARP-WASP Complex | 7.8E-01 | 4.17E-02 |  | RHOB,WIPF1 |
| 11 | Lipid Antigen Presentation by CD1 | 7.73E-01 | 8.33E-02 |  | CALR |
| 11 | Fatty Acid α-oxidation | 7.73E-01 | 8.33E-02 |  | PTGS2 |
| 11 | ERK/MAPK Signaling | 7.73E-01 | 2.84E-02 | 1.000 | ATF1,H3F3A/H3F3B,ETS1,RAP1A |
| 11 | IL-15 Signaling | 7.66E-01 | 4.08E-02 |  | VCAM1,CXCL8 |
| 11 | GM-CSF Signaling | 7.66E-01 | 4.08E-02 |  | GNB2L1,ETS1 |
| 11 | Cell Cycle: G1/S Checkpoint Regulation | 7.52E-01 | 4E-02 |  | CCND2,PA2G4 |
| 11 | Hereditary Breast Cancer Signaling | 7.43E-01 | 3.12E-02 |  | POLR2H,UBB,NPM1 |
| 11 | mTOR Signaling | 7.43E-01 | 2.76E-02 |  | RPS23,RHOB,RPS25,PDGFC |
| 11 | Gluconeogenesis I | 7.42E-01 | 7.69E-02 |  | PGK1 |
| 11 | Caveolar-mediated Endocytosis Signaling | 7.39E-01 | 3.92E-02 |  | FLNA,COPZ1 |
| 11 | GDNF Family Ligand-Receptor Interactions | 7.39E-01 | 3.92E-02 |  | DOK3,GAB1 |
| 11 | Toll-like Receptor Signaling | 7.39E-01 | 3.92E-02 |  | TNFAIP3,UBB |
| 11 | Chemokine Signaling | 7.39E-01 | 3.92E-02 |  | CXCR4,CCL2 |
| 11 | GADD45 Signaling | 7.13E-01 | 7.14E-02 |  | CCND2 |
| 11 | Glutathione Redox Reactions I | 7.13E-01 | 7.14E-02 |  | GPX7 |
| 11 | Valine Degradation I | 7.13E-01 | 7.14E-02 |  | BCAT1 |
| 11 | PI3K/AKT Signaling | 7.08E-01 | 3E-02 |  | PTGS2,GAB1,LIMS1 |
| 11 | Small Cell Lung Cancer Signaling | 7.01E-01 | 3.7E-02 |  | PTGS2,PA2G4 |
| 11 | Estrogen Receptor Signaling | 6.91E-01 | 2.94E-02 |  | TAF13,POLR2H,H3F3A/H3F3B |
| 11 | Granzyme A Signaling | 6.86E-01 | 6.67E-02 |  | HIST1H1A |
| 11 | Differential Regulation of Cytokine Production in Intestinal Epithelial Cells by IL-17A and IL-17F | 6.86E-01 | 6.67E-02 |  | CCL2 |
| 11 | Glycolysis I | 6.86E-01 | 6.67E-02 |  | PGK1 |
| 11 | Human Embryonic Stem Cell Pluripotency | 6.66E-01 | 2.86E-02 |  | PDGFRB,LEF1,PDGFC |
| 11 | Epithelial Adherens Junction Signaling | 6.5E-01 | 2.8E-02 |  | TUBB4A,LEF1,RAP1A |
| 11 | Phospholipase C Signaling | 6.48E-01 | 2.52E-02 |  | GNB2L1,RHOB,GNG2,RAP1A |
| 11 | Gαq Signaling | 6.42E-01 | 2.78E-02 |  | GNB2L1,RHOB,GNG2 |
| 11 | Tec Kinase Signaling | 6.35E-01 | 2.75E-02 |  | GNB2L1,RHOB,GNG2 |
| 11 | Cyclins and Cell Cycle Regulation | 6.23E-01 | 3.28E-02 |  | CCND2,PA2G4 |
| 11 | PDGF Signaling | 6.23E-01 | 3.28E-02 |  | PDGFRB,PDGFC |
| 11 | MIF-mediated Glucocorticoid Regulation | 6.16E-01 | 5.56E-02 |  | PTGS2 |
| 11 | Superpathway of Cholesterol Biosynthesis | 6.16E-01 | 5.56E-02 |  | MSMO1 |
| 11 | Endoplasmic Reticulum Stress Pathway | 6.16E-01 | 5.56E-02 |  | CALR |
| 11 | Regulation of Actin-based Motility by Rho | 6.13E-01 | 3.23E-02 |  | RHOB,WIPF1 |
| 11 | Oxidative Phosphorylation | 6.13E-01 | 3.23E-02 |  | COX7B,COX6C |
| 11 | IL-17A Signaling in Gastric Cells | 5.96E-01 | 5.26E-02 |  | CXCL8 |
| 11 | Hematopoiesis from Pluripotent Stem Cells | 5.96E-01 | 5.26E-02 |  | CXCL8 |
| 11 | α-Adrenergic Signaling | 5.93E-01 | 3.12E-02 |  | GNB2L1,GNG2 |
| 11 | Prostate Cancer Signaling | 5.93E-01 | 3.12E-02 |  | LEF1,PA2G4 |
| 11 | Huntington's Disease Signaling | 5.89E-01 | 2.37E-02 |  | POLR2H,GNB2L1,UBB,GNG2 |
| 11 | Neuregulin Signaling | 5.83E-01 | 3.08E-02 |  | DCN,ERRFI1 |
| 11 | Cell Cycle Control of Chromosomal Replication | 5.77E-01 | 5E-02 |  | MCM6 |
| 11 | Role of p14/p19ARF in Tumor Suppression | 5.77E-01 | 5E-02 |  | NPM1 |
| 11 | G Beta Gamma Signaling | 5.74E-01 | 3.03E-02 |  | GNB2L1,GNG2 |
| 11 | Amyotrophic Lateral Sclerosis Signaling | 5.65E-01 | 2.99E-02 |  | SSR4,PDGFC |
| 11 | Antigen Presentation Pathway | 5.42E-01 | 4.55E-02 |  | CALR |
| 11 | CREB Signaling in Neurons | 5.32E-01 | 2.42E-02 |  | POLR2H,GNB2L1,GNG2 |
| 11 | PAK Signaling | 5.3E-01 | 2.82E-02 |  | PDGFRB,PDGFC |
| 11 | IL-1 Signaling | 5.3E-01 | 2.82E-02 |  | GNB2L1,GNG2 |
| 11 | MIF Regulation of Innate Immunity | 5.26E-01 | 4.35E-02 |  | PTGS2 |
| 11 | Molecular Mechanisms of Cancer | 5.22E-01 | 2.02E-02 |  | CCND2,RHOB,LEF1,PA2G4,GAB1,RAP1A |
| 11 | SAPK/JNK Signaling | 5.14E-01 | 2.74E-02 |  | GNG2,GAB1 |
| 11 | phagosome formation | 4.98E-01 | 2.67E-02 |  | FN1,RHOB |
| 11 | NRF2-mediated Oxidative Stress Response | 4.97E-01 | 2.31E-02 |  | UBB,UBE2K,ENC1 |
| 11 | TNFR2 Signaling | 4.96E-01 | 4E-02 |  | TNFAIP3 |
| 11 | IL-17A Signaling in Fibroblasts | 4.96E-01 | 4E-02 |  | CCL2 |
| 11 | Corticotropin Releasing Hormone Signaling | 4.83E-01 | 2.6E-02 |  | PTGS2,RAP1A |
| 11 | Cholecystokinin/Gastrin-mediated Signaling | 4.83E-01 | 2.6E-02 |  | RHOB,PTGS2 |
| 11 | Circadian Rhythm Signaling | 4.82E-01 | 3.85E-02 |  | BHLHE40 |
| 11 | Inhibition of Angiogenesis by TSP1 | 4.82E-01 | 3.85E-02 |  | MMP9 |
| 11 | Germ Cell-Sertoli Cell Junction Signaling | 4.8E-01 | 2.26E-02 |  | TUBB4A,RHOB,RAB8B |
| 11 | Thrombin Signaling | 4.69E-01 | 2.22E-02 |  | GNB2L1,RHOB,GNG2 |
| 11 | Thyroid Cancer Signaling | 4.69E-01 | 3.7E-02 |  | LEF1 |
| 11 | fMLP Signaling in Neutrophils | 4.69E-01 | 2.53E-02 |  | GNB2L1,GNG2 |
| 11 | p38 MAPK Signaling | 4.62E-01 | 2.5E-02 |  | ATF1,H3F3A/H3F3B |
| 11 | tRNA Charging | 4.56E-01 | 3.57E-02 |  | YARS |
| 11 | CCR3 Signaling in Eosinophils | 4.48E-01 | 2.44E-02 |  | GNB2L1,GNG2 |
| 11 | Coagulation System | 4.44E-01 | 3.45E-02 |  | SERPINA1 |
| 11 | Role of Cytokines in Mediating Communication between Immune Cells | 4.44E-01 | 3.45E-02 |  | CXCL8 |
| 11 | Neuroprotective Role of THOP1 in Alzheimer's Disease | 4.44E-01 | 3.45E-02 |  | MMP9 |
| 11 | Nucleotide Excision Repair Pathway | 4.44E-01 | 3.45E-02 |  | POLR2H |
| 11 | B Cell Receptor Signaling | 4.38E-01 | 2.13E-02 |  | ETS1,GAB1,RAP1A |
| 11 | MSP-RON Signaling Pathway | 4.32E-01 | 3.33E-02 |  | CCL2 |
| 11 | Breast Cancer Regulation by Stathmin1 | 4.19E-01 | 2.07E-02 |  | TUBB4A,GNB2L1,GNG2 |
| 11 | P2Y Purigenic Receptor Signaling Pathway | 4.16E-01 | 2.3E-02 |  | GNB2L1,GNG2 |
| 11 | NGF Signaling | 4.16E-01 | 2.3E-02 |  | GAB1,RAP1A |
| 11 | IL-6 Signaling | 4.16E-01 | 2.3E-02 |  | COL1A1,CXCL8 |
| 11 | D-myo-inositol (1,4,5,6)-Tetrakisphosphate Biosynthesis | 4.04E-01 | 2.25E-02 |  | WBP11,NUDT11 |
| 11 | D-myo-inositol (3,4,5,6)-tetrakisphosphate Biosynthesis | 4.04E-01 | 2.25E-02 |  | WBP11,NUDT11 |
| 11 | Eicosanoid Signaling | 3.9E-01 | 2.94E-02 |  | PTGS2 |
| 11 | Actin Cytoskeleton Signaling | 3.87E-01 | 1.97E-02 |  | FN1,FLNA,PDGFC |
| 11 | 14-3-3-mediated Signaling | 3.81E-01 | 2.15E-02 |  | VIM,TUBB4A |
| 11 | Heparan Sulfate Biosynthesis (Late Stages) | 3.71E-01 | 2.78E-02 |  | EXTL2 |
| 11 | Nicotine Degradation II | 3.71E-01 | 2.78E-02 |  | FMO3 |
| 11 | Cardiac β-adrenergic Signaling | 3.54E-01 | 2.04E-02 |  | GNB2L1,GNG2 |
| 11 | Role of Osteoblasts, Osteoclasts and Chondrocytes in Rheumatoid Arthritis | 3.54E-01 | 1.87E-02 |  | LEF1,SFRP2,COL1A1 |
| 11 | Endometrial Cancer Signaling | 3.54E-01 | 2.63E-02 |  | LEF1 |
| 11 | IL-12 Signaling and Production in Macrophages | 3.49E-01 | 2.02E-02 |  | LYZ,SERPINA1 |
| 11 | UVB-Induced MAPK Signaling | 3.45E-01 | 2.56E-02 |  | H3F3A/H3F3B |
| 11 | D-myo-inositol-5-phosphate Metabolism | 3.39E-01 | 1.98E-02 |  | WBP11,NUDT11 |
| 11 | 3-phosphoinositide Degradation | 3.39E-01 | 1.98E-02 |  | WBP11,NUDT11 |
| 11 | TNFR1 Signaling | 3.37E-01 | 2.5E-02 |  | TNFAIP3 |
| 11 | Glutamate Receptor Signaling | 3.37E-01 | 2.5E-02 |  | GNG2 |
| 11 | Cardiac Hypertrophy Signaling | 3.35E-01 | 1.82E-02 |  | GNB2L1,RHOB,GNG2 |
| 11 | Regulation of Cellular Mechanics by Calpain Protease | 3.3E-01 | 2.44E-02 |  | CAST |
| 11 | Lymphotoxin β Receptor Signaling | 3.22E-01 | 2.38E-02 |  | VCAM1 |
| 11 | Semaphorin Signaling in Neurons | 3.15E-01 | 2.33E-02 |  | RHOB |
| 11 | Heparan Sulfate Biosynthesis | 3.15E-01 | 2.33E-02 |  | EXTL2 |
| 11 | 3-phosphoinositide Biosynthesis | 3.03E-01 | 1.83E-02 |  | WBP11,NUDT11 |
| 11 | ATM Signaling | 3.01E-01 | 2.22E-02 |  | CBX5 |
| 11 | Non-Small Cell Lung Cancer Signaling | 2.88E-01 | 2.13E-02 |  | PA2G4 |
| 11 | ERK5 Signaling | 2.88E-01 | 2.13E-02 |  | GAB1 |
| 11 | Unfolded protein response | 2.88E-01 | 2.13E-02 |  | CALR |
| 11 | Endothelin-1 Signaling | 2.87E-01 | 1.77E-02 |  | PTGS2,GAB1 |
| 11 | Induction of Apoptosis by HIV1 | 2.82E-01 | 2.08E-02 |  | CXCR4 |
| 11 | Mitotic Roles of Polo-Like Kinase | 2.82E-01 | 2.08E-02 |  | ANAPC11 |
| 11 | Role of NFAT in Regulation of the Immune Response | 2.75E-01 | 1.72E-02 |  | GNB2L1,GNG2 |
| 11 | Regulation of eIF4 and p70S6K Signaling | 2.75E-01 | 1.72E-02 |  | RPS23,RPS25 |
| 11 | Acute Myeloid Leukemia Signaling | 2.7E-01 | 2E-02 |  | LEF1 |
| 11 | Neurotrophin/TRK Signaling | 2.7E-01 | 2E-02 |  | GAB1 |
| 11 | Macropinocytosis Signaling | 2.7E-01 | 2E-02 |  | PDGFC |
| 11 | Crosstalk between Dendritic Cells and Natural Killer Cells | 2.64E-01 | 1.96E-02 |  | CD83 |
| 11 | Remodeling of Epithelial Adherens Junctions | 2.64E-01 | 1.96E-02 |  | TUBB4A |
| 11 | Hypoxia Signaling in the Cardiovascular System | 2.64E-01 | 1.96E-02 |  | LDHA |
| 11 | GABA Receptor Signaling | 2.59E-01 | 1.92E-02 |  | UBB |
| 11 | VDR/RXR Activation | 2.53E-01 | 1.89E-02 |  | IGFBP5 |
| 11 | Basal Cell Carcinoma Signaling | 2.48E-01 | 1.85E-02 |  | LEF1 |
| 11 | HER-2 Signaling in Breast Cancer | 2.48E-01 | 1.85E-02 |  | MMP2 |
| 11 | Role of BRCA1 in DNA Damage Response | 2.43E-01 | 1.82E-02 |  | ATF1 |
| 11 | NF-κB Signaling | 2.4E-01 | 1.59E-02 |  | PDGFRB,TNFAIP3 |
| 11 | LPS-stimulated MAPK Signaling | 2.38E-01 | 1.79E-02 |  | ATF1 |
| 11 | PEDF Signaling | 2.38E-01 | 1.79E-02 |  | ZEB1 |
| 11 | Agrin Interactions at Neuromuscular Junction | 2.33E-01 | 1.75E-02 |  | LAMB1 |
| 11 | Role of Wnt/GSK-3β Signaling in the Pathogenesis of Influenza | 2.2E-01 | 1.67E-02 |  | LEF1 |
| 11 | BMP signaling pathway | 2.2E-01 | 1.67E-02 |  | MAGED1 |
| 11 | GPCR-Mediated Nutrient Sensing in Enteroendocrine Cells | 2.2E-01 | 1.67E-02 |  | GNG2 |
| 11 | Virus Entry via Endocytic Pathways | 2.07E-01 | 1.59E-02 |  | FLNA |
| 11 | STAT3 Pathway | 2.07E-01 | 1.59E-02 |  | PDGFRB |
| 12 | Remodeling of Epithelial Adherens Junctions | 5.46E00 | 1.37E-01 |  | ARPC2,ACTG1,ACTB,RALA,MAPRE1,TUBA4A,NME1 |
| 12 | GADD45 Signaling | 4.64E00 | 2.86E-01 |  | CDK4,PCNA,CDK1,CCNB1 |
| 12 | Mitotic Roles of Polo-Like Kinase | 4.51E00 | 1.25E-01 |  | ANAPC13,PRC1,HSP90AB1,KIF11,CDK1,CCNB1 |
| 12 | Mechanisms of Viral Exit from Host Cells | 3.39E00 | 1.43E-01 |  | SNF8,ACTG1,LMNB1,ACTB |
| 12 | Granzyme A Signaling | 3.08E00 | 2E-01 |  | HIST1H1E,NME1,SET |
| 12 | Pyrimidine Deoxyribonucleotides De Novo Biosynthesis I | 3.08E00 | 2E-01 |  | RRM2,RRM1,NME1 |
| 12 | Salvage Pathways of Pyrimidine Ribonucleotides | 2.88E00 | 7.81E-02 |  | CDK4,SGK1,TTK,NME1,CDK1 |
| 12 | Pentose Phosphate Pathway (Non-oxidative Branch) | 2.8E00 | 4E-01 |  | TKT,TALDO1 |
| 12 | Cell Cycle Control of Chromosomal Replication | 2.71E00 | 1.5E-01 |  | CDK4,MCM5,CDC6 |
| 12 | Epithelial Adherens Junction Signaling | 2.61E00 | 5.61E-02 |  | ARPC2,ACTG1,SNAI2,ACTB,TUBA4A,TGFBR1 |
| 12 | NAD Phosphorylation and Dephosphorylation | 2.49E00 | 2.86E-01 |  | ACP1,NADK2 |
| 12 | Pyridoxal 5'-phosphate Salvage Pathway | 2.46E00 | 8.16E-02 |  | CDK4,SGK1,TTK,CDK1 |
| 12 | Pentose Phosphate Pathway | 2.37E00 | 2.5E-01 |  | TKT,TALDO1 |
| 12 | RhoGDI Signaling | 2.21E00 | 4.65E-02 | -1.342 | ARPC2,ACTG1,ACTB,GDI2,WASF2,GNAI3 |
| 12 | DNA damage-induced 14-3-3σ Signaling | 2.01E00 | 1.67E-01 |  | CDK1,CCNB1 |
| 12 | Fcγ Receptor-mediated Phagocytosis in Macrophages and Monocytes | 2E00 | 6.06E-02 | 2.000 | ARPC2,ACTG1,ACTB,NCK1 |
| 12 | Gluconeogenesis I | 1.94E00 | 1.54E-01 |  | ENO1,MDH1 |
| 12 | Cell Cycle: G2/M DNA Damage Checkpoint Regulation | 1.91E00 | 7.89E-02 |  | TOP2A,CDK1,CCNB1 |
| 12 | RAN Signaling | 1.82E00 | 1.33E-01 |  | CSE1L,KPNB1 |
| 12 | p53 Signaling | 1.81E00 | 5.33E-02 |  | CDK4,SNAI2,BIRC5,PCNA |
| 12 | Pancreatic Adenocarcinoma Signaling | 1.69E00 | 4.88E-02 |  | CDK4,RALA,BIRC5,TGFBR1 |
| 12 | Tight Junction Signaling | 1.68E00 | 4.03E-02 |  | CDK4,ACTG1,ACTB,TGFBR1,NUDT21 |
| 12 | Estrogen-mediated S-phase Entry | 1.67E00 | 1.11E-01 |  | CDK4,CDK1 |
| 12 | RhoA Signaling | 1.62E00 | 4.65E-02 | 2.000 | ARPC2,ACTG1,ACTB,ANLN |
| 12 | Protein Kinase A Signaling | 1.62E00 | 2.92E-02 |  | HIST1H3C,PPP1CC,ANAPC13,ACP1,HIST1H1E,CDKN3,TGFBR1,GNAI3 |
| 12 | NRF2-mediated Oxidative Stress Response | 1.6E00 | 3.85E-02 |  | DNAJC13,ACTG1,ACTB,MGST2,STIP1 |
| 12 | Uridine-5'-phosphate Biosynthesis | 1.6E00 | 5E-01 |  | UMPS |
| 12 | Crosstalk between Dendritic Cells and Natural Killer Cells | 1.57E00 | 5.88E-02 |  | ACTG1,ACTB,TNFSF10 |
| 12 | Pyrimidine Ribonucleotides De Novo Biosynthesis | 1.5E00 | 9.09E-02 |  | UMPS,NME1 |
| 12 | Sonic Hedgehog Signaling | 1.5E00 | 9.09E-02 |  | CDK1,CCNB1 |
| 12 | Hereditary Breast Cancer Signaling | 1.47E00 | 4.17E-02 |  | CDK4,ACTB,CDK1,CCNB1 |
| 12 | Clathrin-mediated Endocytosis Signaling | 1.47E00 | 3.55E-02 |  | ARPC2,PICALM,ACTG1,ACTB,CSNK2A2 |
| 12 | Aspartate Degradation II | 1.42E00 | 3.33E-01 |  | MDH1 |
| 12 | Breast Cancer Regulation by Stathmin1 | 1.42E00 | 3.45E-02 |  | PPP1CC,TUBA4A,STMN1,GNAI3,CDK1 |
| 12 | Cyclins and Cell Cycle Regulation | 1.37E00 | 4.92E-02 |  | CDK4,CDK1,CCNB1 |
| 12 | Integrin Signaling | 1.35E00 | 3.29E-02 | 2.236 | ARPC2,ACTG1,ACTB,RALA,NCK1 |
| 12 | Tec Kinase Signaling | 1.3E00 | 3.67E-02 |  | ACTG1,ACTB,TNFSF10,GNAI3 |
| 12 | MSP-RON Signaling Pathway | 1.26E00 | 6.67E-02 |  | ACTG1,ACTB |
| 12 | Gap Junction Signaling | 1.25E00 | 3.54E-02 |  | ACTG1,ACTB,TUBA4A,GNAI3 |
| 12 | HIPPO signaling | 1.24E00 | 4.35E-02 |  | PPP1CC,FRMD6,STK4 |
| 12 | Leucine Degradation I | 1.21E00 | 2E-01 |  | MCCC1 |
| 12 | Death Receptor Signaling | 1.19E00 | 4.17E-02 |  | ACTG1,ACTB,TNFSF10 |
| 12 | Signaling by Rho Family GTPases | 1.12E00 | 2.82E-02 | 1.000 | ARPC2,ACTG1,ACTB,STMN1,GNAI3 |
| 12 | Oleate Biosynthesis II (Animals) | 1.07E00 | 1.43E-01 |  | FADS1 |
| 12 | Paxillin Signaling | 1.05E00 | 3.61E-02 |  | ACTG1,ACTB,NCK1 |
| 12 | Germ Cell-Sertoli Cell Junction Signaling | 1.05E00 | 3.01E-02 |  | ACTG1,ACTB,TUBA4A,TGFBR1 |
| 12 | Role of CHK Proteins in Cell Cycle Checkpoint Control | 1.04E00 | 5E-02 |  | PCNA,CDK1 |
| 12 | Regulation of Cellular Mechanics by Calpain Protease | 1.02E00 | 4.88E-02 |  | CDK4,CDK1 |
| 12 | Leukotriene Biosynthesis | 1.01E00 | 1.25E-01 |  | MGST2 |
| 12 | Ephrin Receptor Signaling | 1E00 | 2.9E-02 |  | ARPC2,ACP1,NCK1,GNAI3 |
| 12 | D-myo-inositol (1,4,5,6)-Tetrakisphosphate Biosynthesis | 9.8E-01 | 3.37E-02 |  | PPP1CC,ACP1,SET |
| 12 | D-myo-inositol (3,4,5,6)-tetrakisphosphate Biosynthesis | 9.8E-01 | 3.37E-02 |  | PPP1CC,ACP1,SET |
| 12 | BER pathway | 9.63E-01 | 1.11E-01 |  | PCNA |
| 12 | ATM Signaling | 9.51E-01 | 4.44E-02 |  | CDK1,CCNB1 |
| 12 | Non-Small Cell Lung Cancer Signaling | 9.2E-01 | 4.26E-02 |  | CDK4,STK4 |
| 12 | Cleavage and Polyadenylation of Pre-mRNA | 9.2E-01 | 1E-01 |  | NUDT21 |
| 12 | Granzyme B Signaling | 9.2E-01 | 1E-01 |  | LMNB1 |
| 12 | Mismatch Repair in Eukaryotes | 9.2E-01 | 1E-01 |  | PCNA |
| 12 | Chondroitin Sulfate Degradation (Metazoa) | 9.2E-01 | 1E-01 |  | GM2A |
| 12 | Aryl Hydrocarbon Receptor Signaling | 9.18E-01 | 3.16E-02 |  | CDK4,MGST2,HSP90AB1 |
| 12 | Actin Nucleation by ARP-WASP Complex | 9.05E-01 | 4.17E-02 |  | ARPC2,NCK1 |
| 12 | Angiopoietin Signaling | 8.9E-01 | 4.08E-02 |  | BIRC5,NCK1 |
| 12 | Actin Cytoskeleton Signaling | 8.89E-01 | 2.63E-02 |  | ARPC2,ACTG1,ACTB,WASF2 |
| 12 | Dermatan Sulfate Degradation (Metazoa) | 8.81E-01 | 9.09E-02 |  | GM2A |
| 12 | Insulin Receptor Signaling | 8.79E-01 | 3.03E-02 |  | PPP1CC,SGK1,NCK1 |
| 12 | Caveolar-mediated Endocytosis Signaling | 8.62E-01 | 3.92E-02 |  | ACTG1,ACTB |
| 12 | D-myo-inositol-5-phosphate Metabolism | 8.6E-01 | 2.97E-02 |  | PPP1CC,ACP1,SET |
| 12 | 3-phosphoinositide Degradation | 8.6E-01 | 2.97E-02 |  | PPP1CC,ACP1,SET |
| 12 | γ-linolenate Biosynthesis II (Animals) | 8.14E-01 | 7.69E-02 |  | FADS1 |
| 12 | NAD Salvage Pathway II | 8.14E-01 | 7.69E-02 |  | ACP1 |
| 12 | 3-phosphoinositide Biosynthesis | 7.9E-01 | 2.75E-02 |  | PPP1CC,ACP1,SET |
| 12 | Agranulocyte Adhesion and Diapedesis | 7.9E-01 | 2.75E-02 |  | ACTG1,ACTB,GNAI3 |
| 12 | Agrin Interactions at Neuromuscular Junction | 7.85E-01 | 3.51E-02 |  | ACTG1,ACTB |
| 12 | Glutathione Redox Reactions I | 7.84E-01 | 7.14E-02 |  | MGST2 |
| 12 | Glycolysis I | 7.57E-01 | 6.67E-02 |  | ENO1 |
| 12 | Ephrin B Signaling | 7.5E-01 | 3.33E-02 |  | ACP1,GNAI3 |
| 12 | PDGF Signaling | 7.39E-01 | 3.28E-02 |  | ACP1,CSNK2A2 |
| 12 | Glutathione-mediated Detoxification | 7.32E-01 | 6.25E-02 |  | MGST2 |
| 12 | TCA Cycle II (Eukaryotic) | 7.32E-01 | 6.25E-02 |  | MDH1 |
| 12 | Regulation of Actin-based Motility by Rho | 7.29E-01 | 3.23E-02 |  | ARPC2,ACTB |
| 12 | Aldosterone Signaling in Epithelial Cells | 7.2E-01 | 2.54E-02 |  | DNAJC13,SGK1,HSP90AB1 |
| 12 | Virus Entry via Endocytic Pathways | 7.18E-01 | 3.17E-02 |  | ACTG1,ACTB |
| 12 | Regulation of IL-2 Expression in Activated and Anergic T Lymphocytes | 7.18E-01 | 3.17E-02 |  | MALT1,TGFBR1 |
| 12 | FAK Signaling | 6.68E-01 | 2.94E-02 |  | ACTG1,ACTB |
| 12 | DNA Methylation and Transcriptional Repression Signaling | 6.65E-01 | 5.26E-02 |  | RBBP7 |
| 12 | NF-κB Signaling | 6.64E-01 | 2.38E-02 |  | MALT1,CSNK2A2,TGFBR1 |
| 12 | VEGF Signaling | 6.5E-01 | 2.86E-02 |  | ACTG1,ACTB |
| 12 | Pyrimidine Ribonucleotides Interconversion | 6.45E-01 | 5E-02 |  | NME1 |
| 12 | Chronic Myeloid Leukemia Signaling | 6.41E-01 | 2.82E-02 |  | CDK4,TGFBR1 |
| 12 | ILK Signaling | 6.38E-01 | 2.31E-02 |  | ACTG1,SNAI2,ACTB |
| 12 | PPARα/RXRα Activation | 6.32E-01 | 2.29E-02 |  | TGFBR1,HSP90AB1,CKAP5 |
| 12 | Factors Promoting Cardiogenesis in Vertebrates | 6.32E-01 | 2.78E-02 |  | TGFBR1,CDC6 |
| 12 | Antiproliferative Role of TOB in T Cell Signaling | 6.27E-01 | 4.76E-02 |  | TGFBR1 |
| 12 | Superpathway of Inositol Phosphate Compounds | 5.95E-01 | 2.19E-02 |  | PPP1CC,ACP1,SET |
| 12 | Protein Ubiquitination Pathway | 5.95E-01 | 1.99E-02 |  | DNAJC13,PSMC5,USP13,HSP90AB1 |
| 12 | Cellular Effects of Sildenafil (Viagra) | 5.9E-01 | 2.6E-02 |  | ACTG1,ACTB |
| 12 | EIF2 Signaling | 5.9E-01 | 2.17E-02 |  | PPP1CC,RPL8,RPL22 |
| 12 | Sertoli Cell-Sertoli Cell Junction Signaling | 5.78E-01 | 2.14E-02 |  | ACTG1,ACTB,TUBA4A |
| 12 | fMLP Signaling in Neutrophils | 5.74E-01 | 2.53E-02 |  | ARPC2,GNAI3 |
| 12 | Leukocyte Extravasation Signaling | 5.73E-01 | 2.13E-02 |  | ACTG1,ACTB,GNAI3 |
| 12 | p38 MAPK Signaling | 5.67E-01 | 2.5E-02 |  | HIST1H3C,TGFBR1 |
| 12 | Glucocorticoid Receptor Signaling | 5.54E-01 | 1.9E-02 |  | SGK1,ACTB,TGFBR1,HSP90AB1 |
| 12 | Inhibition of Angiogenesis by TSP1 | 5.47E-01 | 3.85E-02 |  | TGFBR1 |
| 12 | CD28 Signaling in T Helper Cells | 5.37E-01 | 2.38E-02 |  | ARPC2,MALT1 |
| 12 | Cdc42 Signaling | 5.3E-01 | 2.35E-02 |  | ARPC2,RALA |
| 12 | RAR Activation | 5.3E-01 | 2.01E-02 |  | ACTB,CSNK2A2,PSMC5 |
| 12 | Cell Cycle Regulation by BTG Family Proteins | 5.2E-01 | 3.57E-02 |  | CDK4 |
| 12 | tRNA Charging | 5.2E-01 | 3.57E-02 |  | IARS |
| 12 | Transcriptional Regulatory Network in Embryonic Stem Cells | 5.08E-01 | 3.45E-02 |  | SET |
| 12 | Melanoma Signaling | 4.95E-01 | 3.33E-02 |  | CDK4 |
| 12 | Gαi Signaling | 4.84E-01 | 2.17E-02 |  | RALA,GNAI3 |
| 12 | Dermatan Sulfate Biosynthesis (Late Stages) | 4.73E-01 | 3.12E-02 |  | HS2ST1 |
| 12 | Netrin Signaling | 4.62E-01 | 3.03E-02 |  | NCK1 |
| 12 | PTEN Signaling | 4.55E-01 | 2.06E-02 |  | CSNK2A2,TGFBR1 |
| 12 | Chondroitin Sulfate Biosynthesis (Late Stages) | 4.41E-01 | 2.86E-02 |  | HS2ST1 |
| 12 | Heparan Sulfate Biosynthesis (Late Stages) | 4.32E-01 | 2.78E-02 |  | HS2ST1 |
| 12 | Amyloid Processing | 4.13E-01 | 2.63E-02 |  | CSNK2A2 |
| 12 | T Helper Cell Differentiation | 4.05E-01 | 2.56E-02 |  | TGFBR1 |
| 12 | UVB-Induced MAPK Signaling | 4.05E-01 | 2.56E-02 |  | HIST1H3C |
| 12 | IL-2 Signaling | 3.96E-01 | 2.5E-02 |  | CSNK2A2 |
| 12 | Heparan Sulfate Biosynthesis | 3.72E-01 | 2.33E-02 |  | HS2ST1 |
| 12 | Chondroitin Sulfate Biosynthesis | 3.72E-01 | 2.33E-02 |  | HS2ST1 |
| 12 | Retinoic acid Mediated Apoptosis Signaling | 3.65E-01 | 2.27E-02 |  | TNFSF10 |
| 12 | EGF Signaling | 3.65E-01 | 2.27E-02 |  | CSNK2A2 |
| 12 | GPCR-Mediated Integration of Enteroendocrine Signaling Exemplified by an L Cell | 3.65E-01 | 2.27E-02 |  | GNAI3 |
| 12 | Dermatan Sulfate Biosynthesis | 3.58E-01 | 2.22E-02 |  | HS2ST1 |
| 12 | Xenobiotic Metabolism Signaling | 3.56E-01 | 1.57E-02 |  | HS2ST1,MGST2,HSP90AB1 |
| 12 | Dopamine-DARPP32 Feedback in cAMP Signaling | 3.52E-01 | 1.69E-02 |  | PPP1CC,GNAI3 |
| 12 | CCR5 Signaling in Macrophages | 3.51E-01 | 2.17E-02 |  | GNAI3 |
| 12 | ERK5 Signaling | 3.44E-01 | 2.13E-02 |  | SGK1 |
| 12 | Role of PI3K/AKT Signaling in the Pathogenesis of Influenza | 3.44E-01 | 2.13E-02 |  | GNAI3 |
| 12 | Cell Cycle: G1/S Checkpoint Regulation | 3.24E-01 | 2E-02 |  | CDK4 |
| 12 | Melatonin Signaling | 3.18E-01 | 1.96E-02 |  | GNAI3 |
| 12 | GDNF Family Ligand-Receptor Interactions | 3.18E-01 | 1.96E-02 |  | NCK1 |
| 12 | Chemokine Signaling | 3.18E-01 | 1.96E-02 |  | GNAI3 |
| 12 | Hypoxia Signaling in the Cardiovascular System | 3.18E-01 | 1.96E-02 |  | HSP90AB1 |
| 12 | VDR/RXR Activation | 3.06E-01 | 1.89E-02 |  | PSMC5 |
| 12 | Dopamine Receptor Signaling | 3.06E-01 | 1.89E-02 |  | PPP1CC |
| 12 | Small Cell Lung Cancer Signaling | 3.01E-01 | 1.85E-02 |  | CDK4 |
| 12 | Role of BRCA1 in DNA Damage Response | 2.95E-01 | 1.82E-02 |  | ACTB |
| 12 | Role of NFAT in Cardiac Hypertrophy | 2.95E-01 | 1.5E-02 |  | TGFBR1,GNAI3 |
| 12 | PEDF Signaling | 2.9E-01 | 1.79E-02 |  | WASF2 |
| 12 | Wnt/β-catenin Signaling | 2.88E-01 | 1.48E-02 |  | CSNK2A2,TGFBR1 |
| 12 | Molecular Mechanisms of Cancer | 2.79E-01 | 1.35E-02 |  | CDK4,RALA,TGFBR1,GNAI3 |
| 12 | Bladder Cancer Signaling | 2.75E-01 | 1.69E-02 |  | CDK4 |
| 12 | GPCR-Mediated Nutrient Sensing in Enteroendocrine Cells | 2.7E-01 | 1.67E-02 |  | GNAI3 |
| 12 | Regulation of the Epithelial-Mesenchymal Transition Pathway | 2.69E-01 | 1.42E-02 |  | SNAI2,TGFBR1 |
| 12 | ERK/MAPK Signaling | 2.69E-01 | 1.42E-02 |  | HIST1H3C,PPP1CC |
| 12 | LPS/IL-1 Mediated Inhibition of RXR Function | 2.66E-01 | 1.41E-02 |  | HS2ST1,MGST2 |
| 12 | Hepatic Fibrosis / Hepatic Stellate Cell Activation | 2.62E-01 | 1.4E-02 |  | TNFSF10,TGFBR1 |
| 12 | STAT3 Pathway | 2.56E-01 | 1.59E-02 |  | TGFBR1 |
| 12 | α-Adrenergic Signaling | 2.51E-01 | 1.56E-02 |  | GNAI3 |
| 12 | Prostate Cancer Signaling | 2.51E-01 | 1.56E-02 |  | HSP90AB1 |
| 12 | Neuregulin Signaling | 2.47E-01 | 1.54E-02 |  | HSP90AB1 |
| 12 | G Beta Gamma Signaling | 2.43E-01 | 1.52E-02 |  | GNAI3 |
| 12 | ErbB Signaling | 2.43E-01 | 1.52E-02 |  | NCK1 |
| 12 | TGF-β Signaling | 2.39E-01 | 1.49E-02 |  | TGFBR1 |
| 12 | Apoptosis Signaling | 2.39E-01 | 1.49E-02 |  | CDK1 |
| 12 | TR/RXR Activation | 2.35E-01 | 1.47E-02 |  | ENO1 |
| 12 | Nitric Oxide Signaling in the Cardiovascular System | 2.35E-01 | 1.47E-02 |  | HSP90AB1 |
| 12 | PPAR Signaling | 2.35E-01 | 1.47E-02 |  | HSP90AB1 |
| 12 | Glioma Signaling | 2.23E-01 | 1.41E-02 |  | CDK4 |
| 12 | PAK Signaling | 2.23E-01 | 1.41E-02 |  | NCK1 |
| 12 | IL-1 Signaling | 2.23E-01 | 1.41E-02 |  | GNAI3 |
| 12 | T Cell Receptor Signaling | 2.23E-01 | 1.41E-02 |  | MALT1 |
| 12 | Telomerase Signaling | 2.19E-01 | 1.39E-02 |  | HSP90AB1 |
| 12 | Natural Killer Cell Signaling | 2.12E-01 | 1.35E-02 |  | NCK1 |
| 12 | CDK5 Signaling | 2.09E-01 | 1.33E-02 |  | PPP1CC |
| 12 | Corticotropin Releasing Hormone Signaling | 2.02E-01 | 1.3E-02 |  | GNAI3 |
| 12 | IGF-1 Signaling | 1.98E-01 | 1.28E-02 |  | CSNK2A2 |

Additional file 11. Table S11. Differentially expressed lncRNA in ISCC and PSCC.

|  |  | **ISCC versus paired controls** | | **PSCC versus paired controls** | |
| --- | --- | --- | --- | --- | --- |
| **Gene symbol** | **Transcript type** | **Fold change** | **P-value** | **Fold change** | **P-value** |
| C10orf111 | antisense | -1.91601 | 3.05E-06 | -1.23712 | 0.02100211 |
| CASC2 | antisense | -1.80748 | 4.53E-06 |  |  |
| PLAC4 | antisense | -2.01144 | 1.13E-05 | -1.18481 | 0.04303672 |
| MIR22HG | lincRNA | -1.88185 | 3.04E-05 |  |  |
| DICER1-AS1 | antisense | -1.56198 | 0.000251882 | -1.17687 | 0.01332322 |
| GLIS3-AS1 | antisense | -1.83222 | 0.000463057 |  |  |
| PARD6G-AS1 | antisense | 1.49788 | 0.003536741 | 1.2665 | 0.03786947 |
| FER1L6-AS1 | antisense | -1.54552 | 0.004000612 |  |  |
| ZNF295-AS1 | lincRNA | -1.87102 | 0.004086718 | -1.20157 | 0.02646281 |
| LINC00173 | lincRNA | 1.65147 | 0.004518188 |  |  |
| MLLT4-AS1 | lincRNA | -1.34646 | 0.004712316 |  |  |
| C22orf24 | antisense | -1.52668 | 0.004812967 |  |  |
| RHPN1-AS1 | antisense | -1.30193 | 0.01094157 |  |  |
| LINC00308 | lincRNA | -1.31196 | 0.01128151 | -1.18131 | 0.02126401 |
| LINC00479 | lincRNA | -1.2925 | 0.01433035 |  |  |
| DIO3OS | lincRNA | -1.3312 | 0.01646057 | -1.27936 | 0.02388915 |
| MIR31HG | sense_overlapping | -1.32368 | 0.01820413 |  |  |
| RUSC1-AS1 | antisense | -1.35851 | 0.01886138 | -1.20258 | 0.01302492 |
| C1orf229 | lincRNA | 1.23737 | 0.02268396 |  |  |
| CROCCP2 | lincRNA | -1.241 | 0.02734764 | -1.46455 | 0.005892631 |
| C20orf197 | lincRNA | -1.19313 | 0.03050528 |  |  |
| LINC00189 | sense_overlapping | -1.4542 | 0.03194367 |  |  |
| JAKMIP2-AS1 | antisense | 1.42709 | 0.03324214 |  |  |
| LINC00311 | lincRNA | -1.24829 | 0.03343568 |  |  |
| SLC22A18AS | antisense | -1.20695 | 0.04312258 |  |  |
| C9orf62 | lincRNA | -1.21257 | 0.04352116 |  |  |
| GRIK1-AS1 | antisense | -1.33678 | 0.0447607 |  |  |
| LINC00482 | lincRNA | -1.23134 | 0.04841678 |  |  |
| DLEU2 | antisense | 1.37614 | 0.04853319 |  |  |
| RPL34-AS1 | lincRNA |  |  | -1.15335 | 0.0121407 |
| PCBP1-AS1 | lincRNA |  |  | -1.15117 | 0.03129921 |
| PCBP1-AS1 | antisense |  |  | -1.15117 | 0.03129921 |
| SND1-IT1 | lincRNA |  |  | 1.31032 | 0.01201417 |
| FAM27L | lincRNA |  |  | 1.47539 | 0.01327017 |
| C1RL-AS1 | antisense |  |  | -1.27081 | 0.02365462 |
| CYP1B1-AS1 | antisense |  |  | -1.15211 | 0.01917797 |
| HCG9 | lincRNA |  |  | -1.29523 | 0.01119898 |
| FER1L6-AS2 | antisense |  |  | -1.12057 | 0.04009566 |
| LINC00282 | lincRNA |  |  | -1.20182 | 0.02724167 |
| C5orf58 | antisense |  |  | 1.19662 | 0.006814322 |
